# Quantitative proteomics of *Sf*21 cells during Baculovirus infection reveals progressive host proteome changes and its regulation by viral miRNA

**DOI:** 10.1038/s41598-017-10787-z

**Published:** 2017-09-07

**Authors:** Nishtha Nayyar, Inderjeet Kaur, Pawan Malhotra, Raj K. Bhatnagar

**Affiliations:** 1 Insect Resistance Group, International Centre for Genetic Engineering and Biotechnology, Aruna Asaf Ali Marg, New Delhi, 110067 India; 2 Institute of Stem Cell Biology and Regenerative Medicine, National Centre for Biological Sciences, GKVK, Bellary Road, Bangalore, 560065 India; 3 Malaria Biology Group, International Centre for Genetic Engineering and Biotechnology, Aruna Asaf Ali Marg, New Delhi, 110067 India

## Abstract

System level knowledge of alterations in host is crucial to elucidate the molecular events of viral pathogenesis and to develop strategies to block viral establishment and amplification. Here, we applied quantitative proteomics approach to study global proteome changes in the host; *Spodoptera frugiperda* upon infection by a baculovirus, *Spodoptera litura NPV* at two stages i.e. 12 h and 72 h post infection. At 12 hpi, >95% of host proteins remained stable, however at 72 hpi, 52% host proteins exhibited downregulation of 2-fold or more. Functional analysis revealed significant upregulation of transposition and proteasomal machinery while translation, transcription, protein export and oxidative phosphorylation pathways were adversely affected. An assessment of perturbed proteome after viral infection and viral miRNA expression led to the identification of 117 genes that are potential targets of 10 viral miRNAs. Using miRNA mimics, we confirmed the down regulation of 9 host genes. The results comprehensively show dynamics of host responses after viral infection.

## Introduction

Establishment and replication of viruses in their host is dependent upon the interplay between viral and host factors where the latter largely outnumber their viral counterparts. A systemic analysis of temporal changes in the expression of host components can reveal the sequential impact of viral factors upon host physiology, yielding critical viral pathogenesis mechanisms and cues to alleviate them. In recent years, a combination of transcriptomics, proteomics and other high-throughput technologies have allowed us to gain important insights into virus-host interactions at the molecular level^1, 2^. Also, the data suggests that besides some specific pathways, different viruses target common pathways and machineries, which forms the basis for the discovery of broad range antiviral inhibitors or drugs^3, 4^. Our study explores the sequential effect of baculoviral infection on the proteome of Lepidopteran cell line.

Baculoviruses are natural pathogens of over 600 species of insects, predominant of which are Lepidopterans and are frequently employed in a wide range of biotechnological applications. Due to their inability to replicate in mammalian cells, these are being used as successful vectors for the expression of thousands of proteins and are also being studied as potential vectors for gene therapy^5^. Certain baculoviruses are used in agriculture and forestry as viable alternatives to chemical insecticides in insect pest control^6, 7^. Baculoviruses comprise of a dsDNA genome and replicate in nucleus of insect cells. The life cycle is broadly divided in three phases; an early phase(0–6 hpi) where actin-based motility drives the virus in host nucleus and early viral proteins are transcribed using host RNA polymerase; a late phase(6–24 hpi) marked by viral DNA replication, viral RNA polymerase driven transcription and release of budded virions from cell envelope; and a very late phase (>24 hpi) where virus forms occlusion bodies in the nucleus of infected cell^8, 9^. Carsten *et al*. (1979) first reported that baculoviral infection leads to shutoff of host protein synthesis upon 18 h of *Autographa californica* multinucleopolyhedrovirus (*Ac*MNPV) infection^10^. Subsequently, shutoff of host mRNA expression was also noticed between 12–18 h of infection^11^. High-throughput transcriptomic studies performed at different stages of infection have identified differential regulation of several host pathways like stress response, heat shock response, metabolism, protein expression, ER trafficking etc^12, 13^. But till date, there have been very few studies, which have systematically investigated the effect of baculoviral infection on the host proteome. Carinhas *et al*. (2011) and Yu *et al*. (2015) have studied the differential proteome of *S. frugiperda* cells after baculovirus infection at 6 hpi and 12 hpi intervals and identified differential regulation of 648 and 413 proteins respectively^14, 15^. Recently, Xing *et al*. (2017) have carried an integrated transcriptomic and proteomic analysis in infected fat body of *Helicoverpa armigera* and identified differential regulation of ~450 proteins, in particular those involved in cell metabolism^16^.

In this report, we describe comprehensive proteome profile of Lepidopteran cell line *Sf21*, identifying 5915 host proteins upon infection with a wild type baculovirus, *Spodoptera litura* NPV (*Splt*NPV) at two time-points i.e 12 h and 72 h post infection. Further, we carefully analysed the impact of viral infection on different biological pathways. The proteomic changes post infection were studied in context to gene regulation by baculoviral miRNAs. Nine of the predicted viral miRNA targets were validated by transfecting viral miRNA mimics in *Sf21* cells. The present work thus describes a comprehensive analysis of proteome of *Sf21* cells at early and late stages of *Splt*NPV infection and the regulatory mechanisms activated upon *Splt*NPV infection.

## Results

### *Splt*NPV infection and quantitative proteomics of infected *Sf21* cells

**Figure 1:**
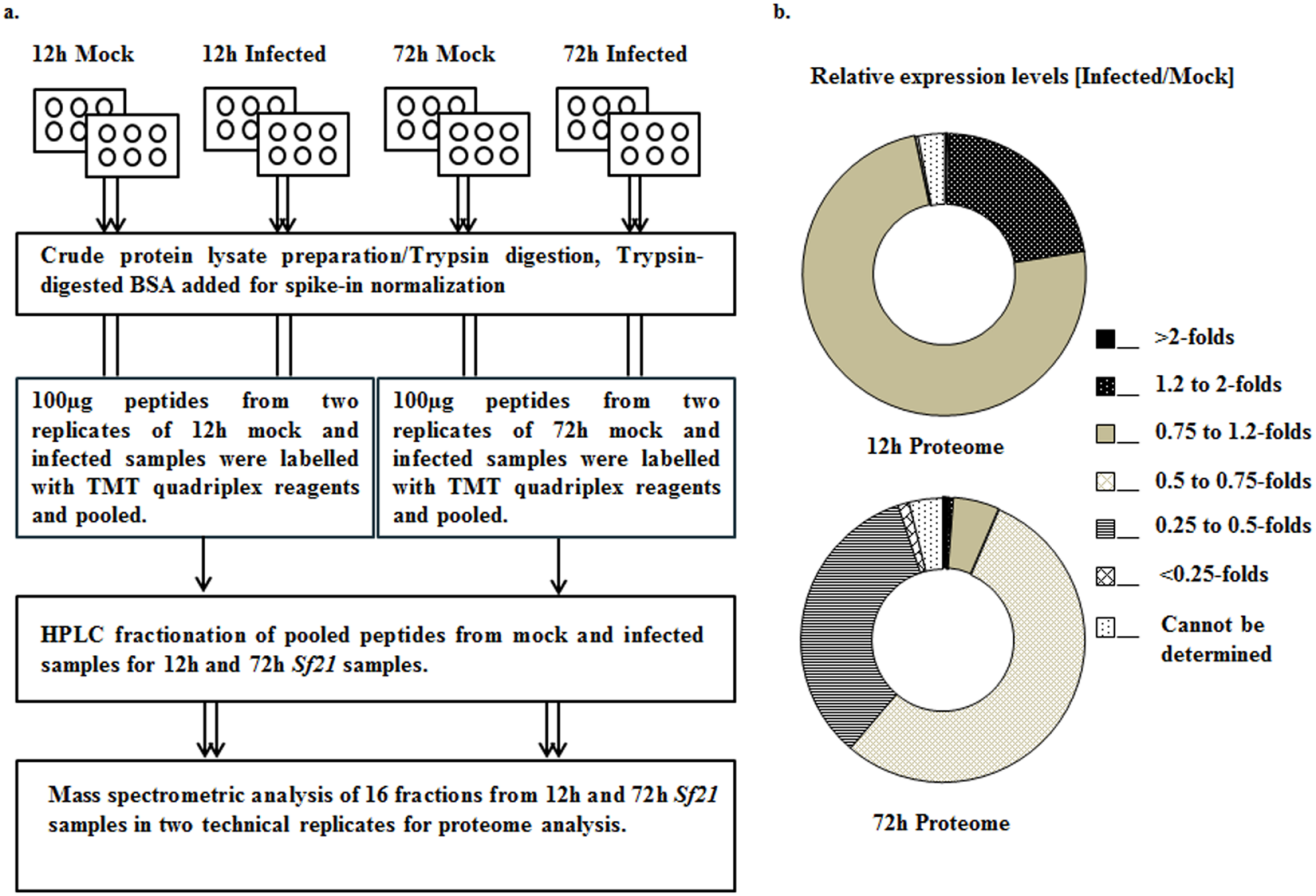
(**a**) Overview of the steps followed for global proteome analysis of *Splt*NPV infected *Sf21* cells. (**b**) Differential regulation of *Sf21* proteins at 12 h and 72 h post *Splt*NPV infection. The proteins are divided in seven categories based upon their relative expression in Infected/Mock samples and their distribution across the total proteome has been depicted.

To gain insights into baculoviral pathogenic mechanisms, *Sf21* cells were infected with wild-type *Splt*NPV at two different time intervals of infection i.e 12 hpi representing the late phase of infection and associated with viral replication and budded virion formation; and 72 hpi that represents the very late phase of viral infection characterized by occluded virion formation. The time intervals thus chosen were expected to yield dynamic changes in host protein expression as per the transcriptomic studies. *Sf21* cells were infected with *Splt*NPV at a low MOI to permit viral establishment as well as to avoid considerable cell lysis. Viral infection of insect cells was confirmed by the enlargement of cells as well as nuclei and appearance of occlusion bodies. At 12 hpi and 72 hpi, mock-infected and *Splt*NPV-infected *Sf21* cells in two biological replicates each were harvested and crude protein extract was prepared. Equal amount of lysate from each sample was subjected to trypsin digestion and the resultant peptides were labelled using different reporter ions of a quadruplex TMT labelling kit. The labelled peptide mixtures of mock and infected cells from two biological replicates were pooled together for 12 hpi and 72 hpi samples. The samples were fractionated using HILIC chromatography and sixteen fractions thus obtained were analysed using Orbitrap LC-MS/MS in two technical replicates each. The mass spectra obtained from all the samples were combined to analyse the global proteome at each time interval. A diagrammatic representation of the experimental setup is shown in Fig. 1a. The spectra were mapped onto predicted proteins from recently reported *Sf21* genome assembly^17^. A total of 4733 protein groups with 15980 peptides and 3914 protein groups with 11222 peptides were identified at 5% FDR from 12 hpi and 72 hpi respectively. At early stage, 3116 proteins were found to be common between two technical replicates while 2580 proteins were common at late stage. In all, this accounts for nearly 40% of the predicted proteome of *S. frugiperda*. Additionally, 101 *Splt*NPV proteins out of a total of 141 viral proteins were identified, with 61 proteins commonly present at both the time intervals. Six proteins were specific to the early stage, while thirty four of them were specifically found at late stages of infection including the well characterized very late phase proteins like Polyhedrin and P10. A list of all *Sf21* and *Splt*NPV proteins identified from the protein pool at both time intervals is provided in Supplementary Tables S1 and S2 respectively. Table 1 lists the total number of peptides/proteins identified upon mass spectrometric analysis of protein lysates from 12 h and 72 h mock/infected *Sf21* cells.

**Table 1:** Statistics of data obtained from LC-MS/MS analysis of mock-infected and *Splt*NPV infected *Sf21* cells at 12 h and 72 h of infection.

12 h Mock/Infected *Sf21* cell sample (Two biological replicates)		72 h Mock/Infected *Sf21* cell sample (Two biological replicates)	
Technical Replicate1		Technical Replicate1	
No. of protein groups	4068	No. of protein groups	3179
No. of peptides	15391	No. of peptides	10469
Technical Replicate2		Technical Replicate2	
No. of protein groups	4015	No. of protein groups	3342
No. of peptides	15980	No. of peptides	11222

Trypsin digested peptides were labelled with TMT-quadriplex reagents and subjected to LC-MS/MS.

### Quantitative proteomics reveals differential host proteome regulation at different time intervals of *Splt*NPV infection

As expected, the overall pattern of host protein regulation was markedly distinct at the two time intervals tested. In case a stringent criterion of upto two-fold regulation is considered, the levels of nearly 95% of the proteins remained unaltered after 12 h of infection, while 0.5% of them were upregulated. Only 0.02% of the proteins were found to be downregulated at this stage. In comparison, at late stages of viral infection >50% of the host proteins were downregulated, whereas 43% of them remain unchanged. It is worthwhile to mention here that the expression of >90% of the host proteins at this stage showed marked reduction of upto 0.75-folds with respect to mock infected samples. 5% of the proteins exhibited a downregulation of three-fold or higher after 72 h of infection. In contrast, the amount of upregulated proteins was very less i.e. 0.8% of the total host proteome. Figure 1b depicts the global pattern of differential regulation of *Sf21* proteins at 12 h and 72 h of *Splt*NPV infection. Expression levels of all detected host proteins with respect to mock infection at 12 hpi and 72 hpi are provided in Supplementary Table S1.

Despite the protein shutdown at late stages, 0.5% and 0.8% of the total proteome exhibited induced expression during early and late stages of baculoviral infection in respective order. A Pao retrotransposon peptidase family protein was found to be highly upregulated at both 12 hpi and 72 hpi time intervals; it showed 20-fold and 3-fold higher expression in infected cells at respective time intervals. At 72 h interval, Ubiquitin protein ligase E3B- like protein was the most over-expressed protein showing an activation of 22-folds in infected cells. A Sentrin/Sumo-specific protease Senp7 was also up-regulated by 8-folds. Certain other ubiquitination proteins like UBA domain-containing protein 1, DDB1- and CUL4-associated factor 7-like protein were slightly induced upon 12 h of *Splt*NPV infection. Immune defence-related proteins like antimicrobial protein 6Tox precursor, Hemocyte protein-glutamine gamma-glutamyltransferase-like, RNAi factors like maternal protein Tudor-like, R2D2 protein were up-regulated at 12 h of infection. Chitinase-precursor was up-regulated by 1.8-folds and 6.8-folds at 12 h and 72 h post infection time intervals. Ecdysis triggering hormone receptor isoform B was found to be overexpressed by 5-folds in 72 h of infection, while Ecdysone 20-hydroxylase showed 1.7-fold higher expression at 12 hpi. A vast majority of proteins at early stages of infection showed no significant down-regulation in expression after *Splt*NPV infection. A clear exception was hypothetical protein LOC100167034, which showed two-fold down-regulation in its expression in infected cells. Expression of only 0.4% of the total proteins was reduced by 0.75-folds or higher at this stage which included proteins like cytokinesis regulators, ATP binding cassette, kinesin and ferritin among others. Quant spectra of certain representative proteins at different time intervals of infection are shown in Supplementary Figure S1.

### Functional annotation of *Splt*NPV infected *Sf21* proteome

**Figure 2:**
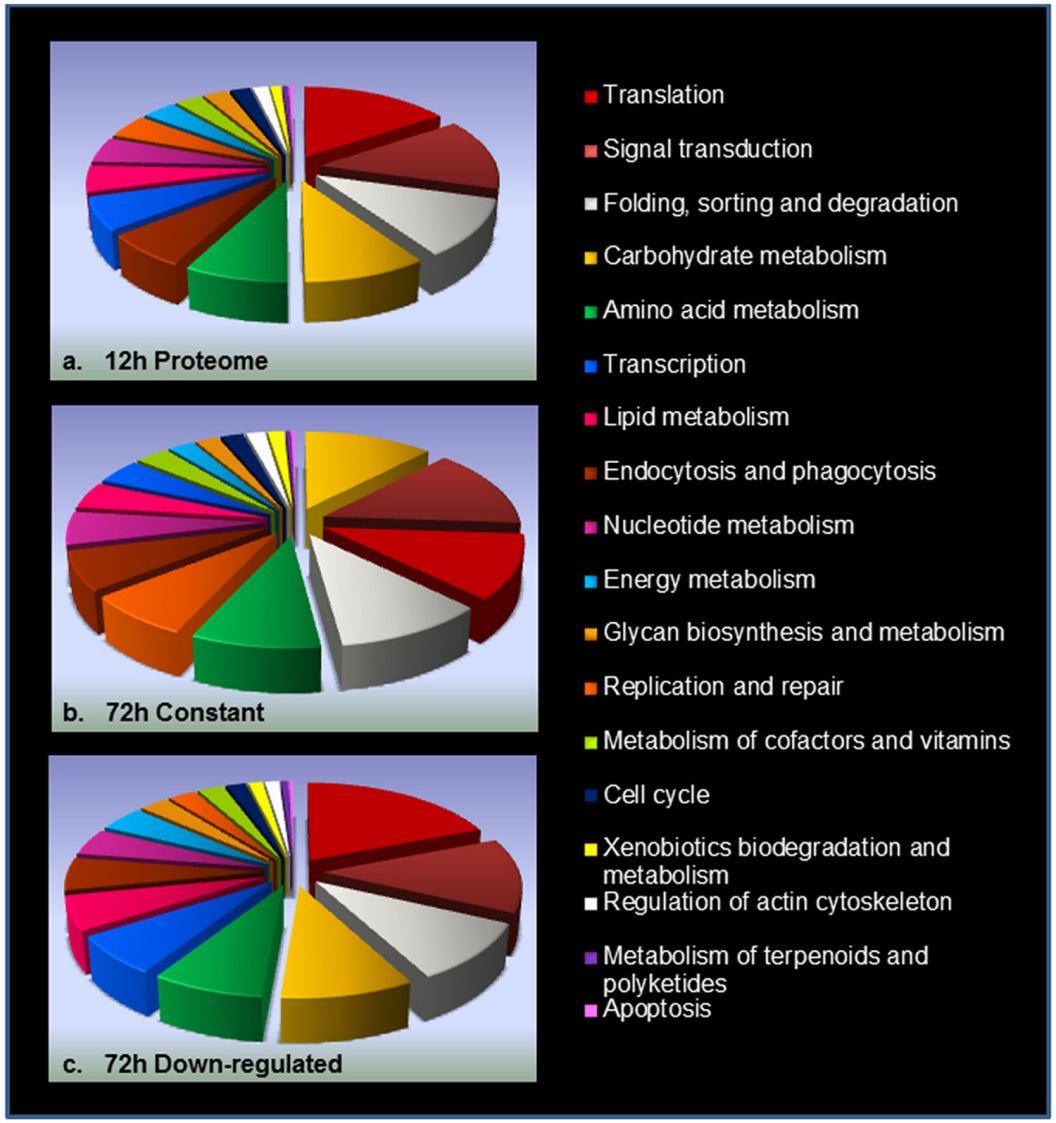
KEGG pathway distribution of proteins obtained upon LC-MS/MS analysis of *Sf21* cells upon *Splt*NPV infection. (**a**) 12 h proteome, (**b**) 72 h constant proteome, (**c**) 72 h down-regulated proteome. Proteins exhibiting at least two-fold reduction in their expression were considered down-regulated. Relative abundance of 18 most abundant KEGG pathways is portrayed in each category.

Functional annotation of *Sf21* proteome was performed using KEGG Automation Annotation Server (KAAS) that compares the proteins on the basis of their BLAST homology with KEGG genes database. KEGG pathways and Orthologues could be assigned to nearly 65% of the detected *Sf21* proteins. 3094 and 2573 proteins were respectively annotated in 12 h and 72 h proteome assembly. KEGG pathway distribution of the 12 h proteome is shown in Fig. 2a. The largest proportion of the proteins detected at 12 h interval belonged to translational machinery (15.6%), followed by signal transduction mechanisms (13.67%), protein folding, sorting and degradation (11%), carbohydrate metabolism (9.5%) and amino acid metabolism (8%).

To get a better idea of impact on host physiological pathways after infection, we divided the 72 h proteome in two broad categories, ones displaying more than two-fold reduction in their expression represented the down-regulated category while the ones displaying relative expression values between 0.5-folds to 1.5-folds represented the relatively constant category. It is to note that the constant category does include vast majority of proteins which show down-regulation upon infection but we included them in a separate category since their relative suppression was lower than the down-regulated category. We wanted to study the distribution of KEGG pathways across the two categories and find if there was a differential impact of viral infection on specific KEGG pathways. At 72 h of infection, we did observe substantial differences across BRITE hierarchy distribution between the two categories. The pathways which were most afflicted upon *Splt*NPV infection were those involved in translational machinery, energy and lipid metabolism, transcription while DNA replication and repair, carbohydrate metabolism remained relatively unaltered throughout the course of infection. KEGG pathway distribution of host proteome at 72 hpi across both the categories is shown in Fig. 2b,c.

**Figure 3:**
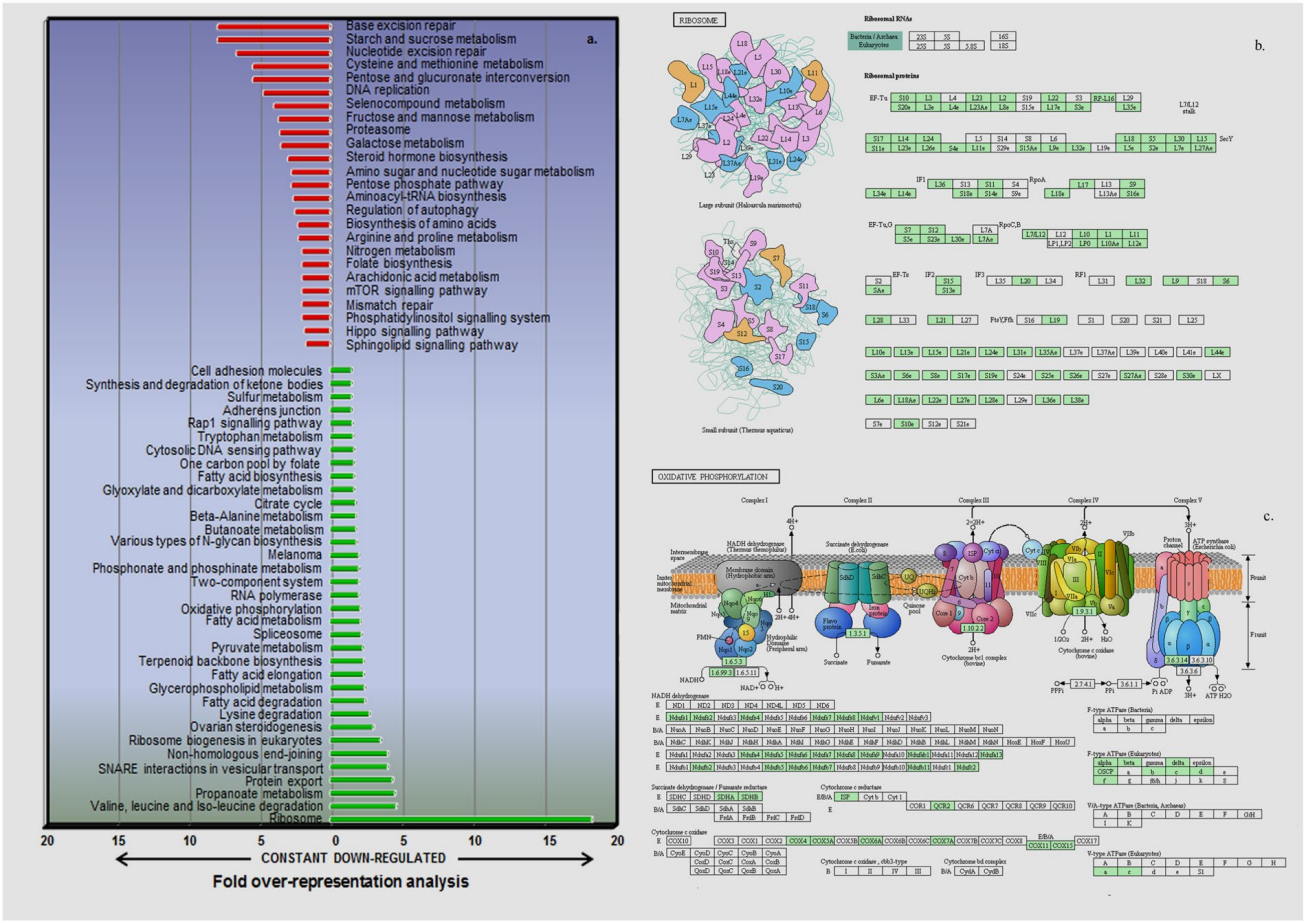
Specific inhibition of host physiology after *Splt*NPV infection. (**a**) KEGG pathway enrichment across constant and down-regulated *Sf21* proteins at late stages of viral infection. Ribosomal proteins were over-represented in down-regulated category by 18-folds in comparison to the unchanged group while Base-excision repair proteins were 5-times more prevalent in constant category. Depiction of down-regulated components of (**b**) Ribosomal machinery, (**c**) Oxidative phosphorylation which represent highly afflicted KEGG pathways at late stages of *Splt*NPV infection. The proteins which were found to be down-regulated in TMT labelling analysis are shown in green. Pathway maps were generated using KAAS^58–61^.

Upon further inspection, it was observed that the proteins belonging to the ribosomal machinery underwent maximum repression showing 18-times higher representation in the down-regulated group in comparison to the constant one. Also, proteins involved in amino acid degradation, fatty acid metabolism, protein export, oxidative phosphorylation and splicing exhibited significant suppression. In comparison, proteins related to DNA replication and repair mechanisms were reasonably unaltered even after 72 h of infection and were five-fold enriched in the constant category. Other pathways showing relatively constant expression were Proteasomal machinery, starch and sucrose metabolism, steroid hormone biosynthesis, and pentose glucoronate interconversion pathway, indicating that these pathways show less repression in infected cells. A representation of all pathways along with their fold enrichment in down-regulated and constant category is provided in Fig. 3a and the down-regulated components of Ribosomal machinery and Oxidative phosphorylation pathways which had been highly afflicted upon *Splt*NPV infection are shown in Fig. 3b,c.

### Expression analysis of selected genes upon *Splt*NPV infection

**Figure 4:**
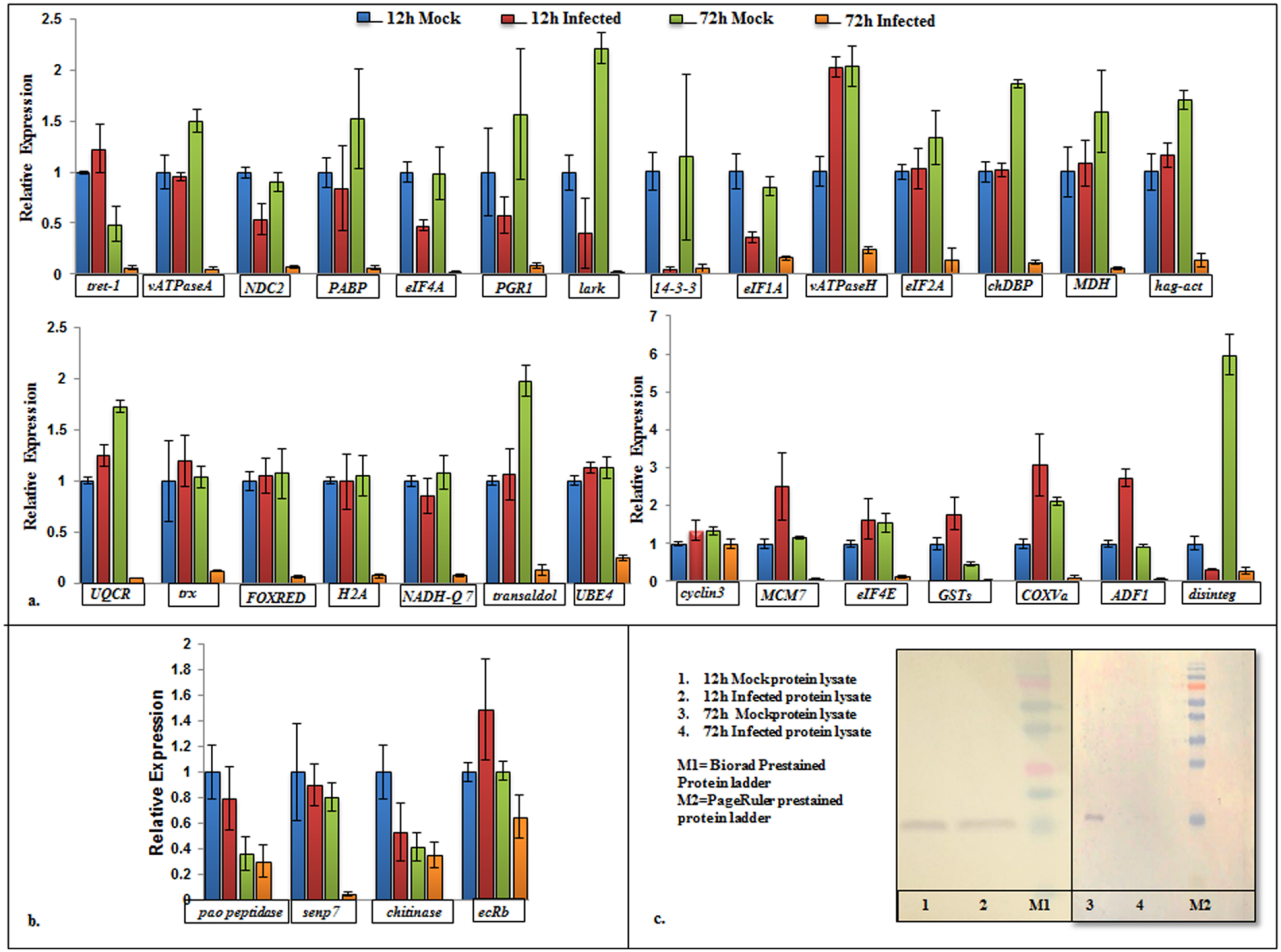
Comparative expression analysis of proteome data. (**a**) qPCR based assessment of gene expression in mock-infected and *Splt*NPV infected *Sf21* cells at 12 h and 72 h post infection. The tested *Sf21* genes were reportedly down-regulated at 72 h of infection in proteomics analysis. qPCR analysis confirms down-regulation of 27 out of 28 genes. [*tret-1*: *facilitated trehalose transporter*, *vATPaseA*: *V-type proton ATPase catalytic subunit A*, *NDC2*: *NADH dehydrogenase [ubiquinone] 1 subunit C2*, *PABP*: *poly(A)-binding protein*, *eIF4A*: *eukaryotic initiation factor 4* *A*, *PGR1*: *prostaglandin reductase 1*, *lark*: RNA binding protein Lark, *14-3-3*: *14-3-3 epsilon protein*, *eIF1A*: *eukaryotic translation initiation factor 1* *A*, *vATPaseH*: *V-type proton ATPase subunit H isoform 2*, *eIF2A*: *eukaryotic translation initiation factor 2* *A*, *chDBP*: *chromodomain helicase DNA binding protein*, *MDH*: *malate dehydrogenase*, *hag-act*: *heparan-alpha-glucosaminide N-acetyltransferase*, *UQCR*: *ubiquinol-cytochrome c reductase*, *trx*: *thioredoxin isoform* X*1*, *FOXRED*: *FAD-dependent oxidoreductase domain-containing protein 1*, *H2A*: *H2A histone family member V*, *NADH-Q7*: *NADH-ubiquinone oxidoreductase Fe-S protein 7*, *transaldol*: *transaldolase*, *UBE4*: *ubiquitin conjugation factor E4*, *MCM7*: *DNA replication licensing factor MCM7*, *eIF4E*: *eukaryotic translation initiation factor 4E*, *GSTs*: *glutathione S-transferase sigma 1*, *COXVa*: *cytochrome c oxidase subunit Va*, *ADF1*: *actin-depolymerizing factor 1*, *disinteg*: *disintegrin and metalloproteinase domain-containing protein* 12]; (**b**) Relative expression of four *Sf21* genes which were found to be up-regulated in proteomics analysis [*pao peptidase*: *pao retrotransposon peptidase family protein*, *senp7*: *sentrin/sumo-specific protease senp7*, *chitinase*: *chitinase precursor*, *ecRb*: *ecdysis triggering hormone receptor isoform B*] The genes show similar mRNA levels in both infected and mock-infected samples; (**c**) Comparative expression of *Sf21* protein Histone H3 in mock-infected and infected cells at 12 h (Lane1, 2) and 72 h (Lane 3, 4) post infection through western blot. Blots depicting expression at 12 h (Lane 1,2) and 72 h (Lane 3, 4) were processed separately. The figure shows no change in expression of Histone H3 at 12 h in mock and infected cells but a marked reduction in its expression at 72 h.p.i in *Splt*NPV infected cells v/s mock-infected cells.

qPCR analysis of thirty two genes was performed for comparative expression analysis of the proteomic data. Quantitative proteomics data suggested that at 12 h of infection, two of these proteins were up-regulated while rest thirty showed no change in protein expression level upon infection. At 72 h of infection, twenty eight of these genes were predicted to be down-regulated by proteomic analysis, while four were up-regulated. The results from qPCR analysis of all thirty two tested *Sf21* genes are shown in Fig. 4a,b. Upon qPCR analysis, it was observed that most of the genes showed expression patterns similar to the ones obtained in proteomic analysis, with a general trend of unchanged expression at 12 hpi and downregulation at 72 hpi. However, at 12 hpi, mRNA levels of some genes like *14-3-3 epsilon*, *lark*, translation initiation factors *eIF*1*a*, *eIF4a, V-type proton ATPase subunit H isoform 2*, MCM7, *cytochrome c oxidase subunit Va*, *glutathione S-transferase sigma* 1, *actin-depolymerizing factor 1* did not quite correlate with unchanged protein levels observed at the proteomic level. The differences observed at mRNA level of certain genes are not unexpected owing to variable protein half-lives and existence of post-transcriptional gene regulatory pathways in eukaryotic cells. Nevertheless, at 72 hpi there was a higher correlation between mRNA and protein levels since twenty seven out of twenty eight down-regulated proteins showed remarkably lower mRNA levels as well. Transcript levels of only one gene, *cyclin3*, did not show any decline as opposed to its protein levels and it is well established that cyclin proteins are generally regulated at proteomic level through ubiquitination^18^. For the genes which showed upregulated expression in our proteomic analysis, three out of the four genes tested displayed similar mRNA levels in the infected samples as the mock infected ones (Fig. 4b). We did not observe up-regulation at transcriptional level, but notably these were neither down-regulated as rest of the 27 genes. Together the results emphasize the significance of undertaking studies at the proteomic level.

As a representative of general expression trend of our quantitative proteomics analysis, we analysed the expression of *Sf*2*1* Histone H3 using western blot analysis. Proteomics analysis suggested that the levels of this protein were similar in uninfected and infected *Sf*21 cells at 12h of infection but were reduced to more than a half in infected cells at 72h of infection. Western blot analysis of the protein at both the time intervals confirmed these findings (Fig. 4c).

### Regulation of host proteome by *Splt*NPV miRNAs

We had previously identified and validated the expression of ten novel *Splt*NPV miRNAs encoded upon infection of *Sf2*1 cells and predicted their targets using RNAHybrid v2.0^19^. To understand the regulation of host proteome by *Splt*NPV miRNAs, we analysed the expression of their predicted targets in our analysis. 117 predicted targets of viral miRNAs displayed significant downregulation in proteomic analysis while 1 protein was found to be upregulated. A list of all the predicted *Splt*NPV miRNA targets showing differential regulation upon proteome analysis is provided in Supplementary Table S3 along with the observed changes in their levels after infection. Importantly, 28 of these 117 proteins also showed down regulation by qPCR (Fig. 4a). Notably, some of the down-regulated proteins were computationally targeted by more than one baculoviral miRNA. Examples of such proteins include V-type proton ATPase subunit H which was a predicted target of 11672_3p and 11698_3p; Prostaglandin reductase1-like was targeted by 11684_3p and 11701_5p; Cytochrome b5 by 11672_3p and 11660_3p; Translation initiation factors targeted by 11684_3p, 11694_5p, 11672_5p and 11660_3p; Constitutive coactivator of PPAR-gamma-like by 11684_3p and 11701_5p and few more. Additionally, some down-regulated proteins had conserved miRNA binding sites in a related organism *Bombyx mori* as well, indicating possibility of evolutionarily preserved role for viral miRNAs across baculoviral species. For instance, predicted target for 11684_5p is 14-3-3 epsilon protein and its target site is conserved in *B. mori* and *Sf21*. Similarly, binding sites of 11672_3p in RNA-binding protein Lark and Alpha-tocopherol transfer protein are preserved in both the insect species.

**Figure 5:**
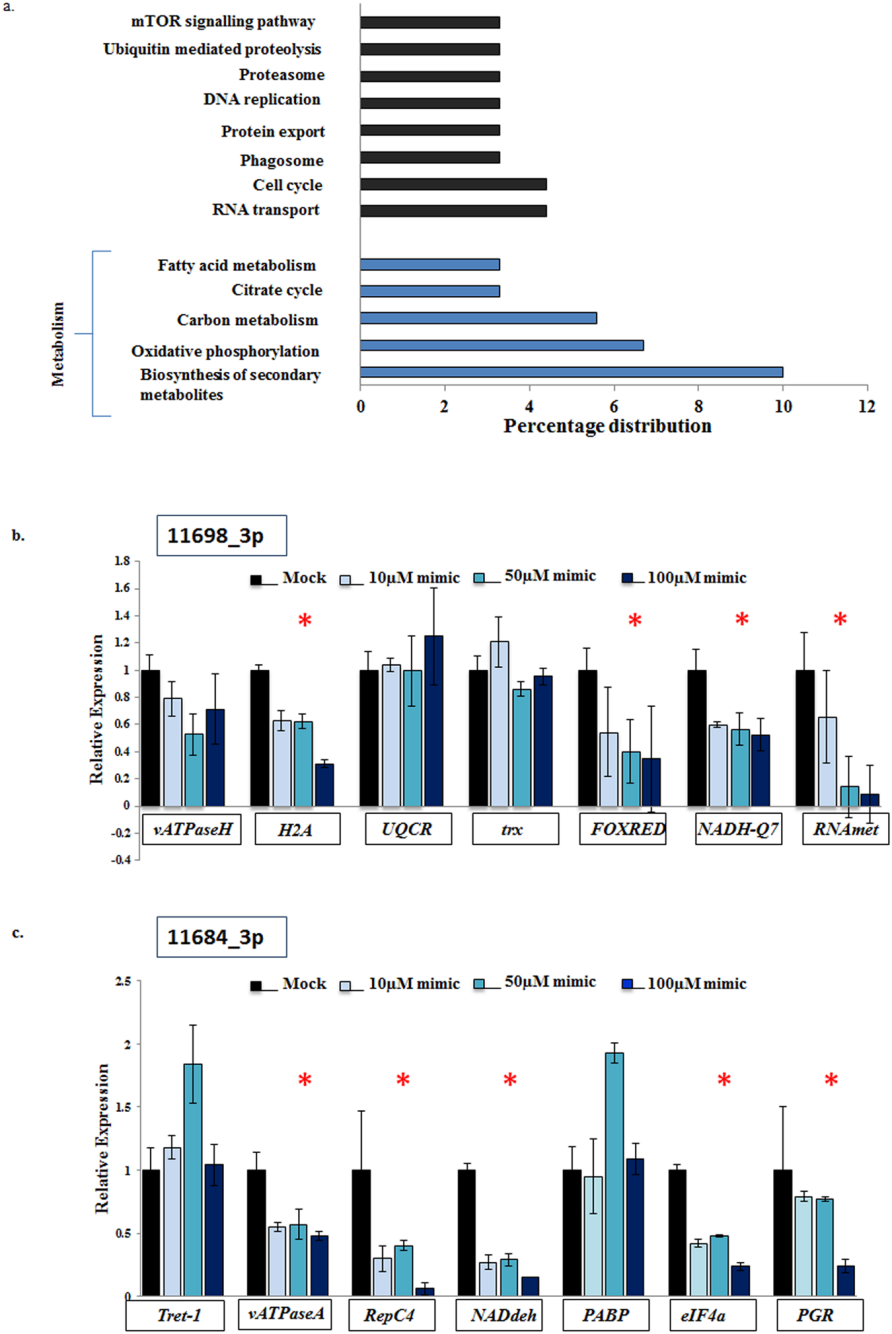
*Splt*NPV miRNAs and host proteome regulation. (**a**) KEGG pathway distribution of 89 predicted miRNA target genes which show profound down-regulation in proteomics analysis. Genes involved in secondary metabolite production and oxidative phosphorylation were enriched in this category. Relative transcript abundance of host genes upon transfection of 10 µM, 50 µM and 100 µM concentrations of miRNA mimics of *Splt*NPV miRNA (**b**) 11698_3p, and (**c**) 11684_3p. The genes marked with red asterisk showed progressively lower transcript levels with increasing concentration of miRNA mimics.

We carried out functional analysis of these 117 genes using KAAS and assigned KEGG orthologues to 89 of these genes. Most of the genes belonged to metabolic pathways (19%) majorly biosynthesis of secondary metabolites (10%) and oxidative phosphorylation (7%). Other targeted pathways were RNA transport, cell cycle, phagosome, protein export and DNA replication. Several KEGG pathways were targeted by multiple miRNAs. For instance, six viral miRNAs had binding sites for proteins involved in secondary metabolite biosynthesis while five of them had predicted targets in oxidative phosphorylation pathway. Figure 5a represents the distribution of most significantly targeted KEGG pathways after proteomic analysis. The analysis reflects upon the probable role of viral miRNAs in regulating metabolic pathways and cellular growth.

### Validation of *Splt*NPV miRNA targets in *Sf21* cells

To determine whether the host proteins which show profound changes upon *Splt*NPV infection are the targets of the viral miRNAs induced during the infection, we chose two viral miRNAs, 11684_3p and 11698_3p for experimental validation, since both of these miRNAs had the highest expression amongst other viral miRNAs (292231 RPM and 263RPM respectively) as observed by next generation sequencing analysis^19^. *Sf21* cells were transfected with increasing concentrations of miRNAs (10 µM, 50 µM, 100 µM) and transcript levels of the corresponding target genes were analysed with respect to mock infected and scrambled miRNA control. In case of 11684_3p, we observed progressive down-regulation in transcript levels of five out of seven genes upon transfection of increasing concentration of miRNA mimics, which includes genes encoding V-type proton ATPase catalytic subunit A-like (*vATPaseA*), replication factor C4 (*repC4*), NADH dehydrogenase [ubiquinone] 1 subunit C2-like (*ndc2*), eukaryotic initiation factor 4A-like isoform 1 (*eIF4A*) and prostaglandin reductase 1 (*pgr1*). After transfection of increasing concentrations of 11698_3p, we observed down-regulation in four out of seven genes namely H2A histone family member V (*H2A*), FAD-dependent oxidoreductase domain-containing protein 1-like (*FOXRED*), NADH-ubiquinone oxidoreductase Fe-S protein 7 (*NADH-Q*7) and RNA methyltransferase like 1 (*RNAmet*). For all these genes, transcript levels after transfection of 100 µM of negative miRNA mimic were comparable to those of mock transfection. Figure 5b,c represent the expression of all tested genes after administration of increasing concentration of both mimics.

To conclude, this study comprehensively analyses the proteomic changes in *Sf21* cells at early and late stages of baculoviral infection and identifies major cellular pathways perturbed upon infection. The work categorically demonstrates the role of baculoviral miRNAs in modulating specific host components and will pave way for future studies on baculoviral pathogenesis and miRNA studies.

## Discussion

Viral infections induce massive remodelling of cellular machinery in order to facilitate their own amplification. A systematic quantitative analysis of temporal changes in host factors throughout the course of productive infection can provide dynamic insights into the host-pathogen interactions and accelerate the discovery of antiviral drugs or inhibitor compounds. Although genomics and transcriptomics technologies have provided a lot of information in last few years on interplay of host-pathogen factors, still, proteome of an organism is quantitatively and qualitatively distinct. An integrated correlative assessment is required to understand the true metabolic state of the organism during pathogenesis. In the present study, we provide comprehensive data describing the temporal changes in the host proteome at two stages of infection i.e 12 hpi and 72 hpi. Simultaneously, we investigated the regulation of genes by viral microRNAs expressed during the course of infection.

Numerous transcriptomic studies have attempted to understand the differential regulation of host gene expression upon baculoviral infection at different stages^12, 13, 16, 20^. However, till date, very few proteomic studies have extensively investigated the host protein repertoire at different stages of infection. While most baculoviral proteomic studies done so far have focussed on viral protein expression^21, 22^, some others have limited their analysis till early stages of infection^14, 15^. We now report a comprehensive differential proteome analysis of *Lepidopteran* cell line; *Sf21* and the changes inflicted on it upon baculoviral infection at early and late stages of infection. A total of 5915 *Sf21* proteins were identified in this analysis, which is the largest Lepidopteran proteome identified so far. Using tandem mass tag labelling, the differential expression of 4709 and 3607 proteins at 12 hpi and 72 hpi was analysed. As discussed earlier, levels of majority of the *Sf21* proteins remained unchanged after 12 h of *Splt*NPV infection, while 94% of the proteins were downregulated by >0.75-folds at 72 hpi. The overall pattern of global down-regulation at late stages of infection conformed to the previous studies at protein and mRNA levels after baculoviral infection as did the levels of several proteins^10, 11, 13, 20^. Also, several viral induced phenomena were well reflected in our data. It has been reported that the replication of *Ac*MNPV in *Sf9* cells leads to activated DNA damage response, which is essential for viral replication^23^. Consistent with the observation, we found base excision repair, nucleotide excision repair and mismatch repair pathways showing unchanged/slightly changed expression even at very late stages of infection. Increased heat shock responses at early stages of infection has been documented in several studies and our analysis conformed to the same^12, 13, 24, 25^. On similar lines, activation of MAPK and phosphatidylinositol 3-kinase (PI3K)-Akt signalling at initial stages of infection is reportedly required for efficient *Bm*NPV replication^26, 27^. Correspondingly, we found increased expression of several proteins related to these pathways at 12 h of infection.

Several transposable elements including retroelements like Pao retrotransposon peptidase family protein, endonuclease/reverse transcriptase, *piggyBac* transposable element-derived protein 4 and enzymes like transposases were found to be significantly upregulated post infection. *PiggyBac* transposable elements were initially identified as insertion sequences in baculoviruses, but were later found to be encoded from the Lepidopteran genomes, after baculoviral infection^28, 29^. Transcripts derived from insect retroviruses, also termed as errantiviruses have previously been shown to be over-expressed in baculovirus infected hemocytes of *Heliothis virescens* larva as well as infected *H. zea* cells^20, 30^. Menzel and Rohrman were the first to speculate an increased transposition in baculovirus infected insect cells and since then several lines of investigation have provided credence to the hypothesis and our results resonate with the same^31, 32^. We had earlier reported significant reduction in piRNA population of *Sf21* cells after 12 h of *Splt*NPV infection^19^. This might be responsible for activated transposition since piRNAs are believed to play an important role in silencing of transposons^33^. However, it would be interesting to understand the advantages of piRNA suppression for baculoviral pathogenesis.

One of the most highly upregulated proteins in our analysis at 72 h of infection was ubiquitin-protein-ligase E3B-like protein. Earlier, Nguyen *et al*. (2013) have also reported E3 ubiquitin protein ligase to be amongst the top 5 upregulated transcripts during 24 and 48 hpi intervals^20^. E3 ubiquitin ligases bind to the protein substrates and act as a scaffold for binding of ubiquitin conjugating enzyme which then marks the protein for degradation^34, 35^. Certain RING finger proteins of baculoviruses have earlier been shown to demonstrate E3 ligase activities suggesting that these viruses might possess the ability to manipulate the specificity of ubiquitination^36^. Studies have also reported increased proteotoxicity, accumulation of ubiquitinated proteins and aggresomes during *Ac*MNPV infection. It was postulated that heat shock proteins fused with the aggresomes and were responsible for diminishing the observed proteotoxicity by lysosomal degradation^37, 38^. In tandem, our study observed increased or constant levels of several components of ubiquitin machinery, heat shock proteins as well as lysosomal proteins during both the stages of infection.

We also found the levels of actin polymerization proteins like Strumpellin and Formin to be induced at 72 h of *Splt*NPV infection. Strumpellin is a subunit of WASH complex, an endosomal Arp2/3 activator belonging to WASP family proteins, which is required for formation of branched actin networks^39, 40^, while Formin is a Rho GTPase which also promotes actin polymerization by associating with the barbed end of actin filaments^41^. Baculoviruses have been known to promote actin rearrangement and nucleation by virtue of its own proteins like Arif-1 and p78/83^42, 43^. Our data indicates active actin cytoskeletal dynamics at 72 h of infection, which might be helpful for viral egress at very late stages of infection.

Amongst the most highly afflicted processes after infection, translation ranked amongst the highest. Previously, transcriptional repression of several ribosomal proteins, EF-Tu, EF1d, eIF3-6, eIF3-2b, eIF1a and down-regulated protein levels of eIF5A and eIF4E have been observed^13, 44–46^. Expression of all these proteins as well as other proteins involved in translational mechanisms was found to be highly suppressed upon infection in our analysis at 72 h of infection. Besides, several ATP dependent RNA helicases, which play important roles in transcription regulation, splicing, ribosome biogenesis, mRNA export and other RNA metabolism pathways were found to be highly suppressed after viral infection at late stages^47^. The down-regulation of vesicular transport and protein export mechanisms observed in the study might have its implications in regulation of budded virus formation and transition to the occluded form.

Metabolic perturbation is a well-studied aspect of virus induced pathology and several viruses have been found to result in metabolic or mitochondrial dysfunction^48–50^. Recent study of Xing *et al*. (2017) has highlighted the impact of viral infection on metabolic genes/proteins^16^. Consistent to their study, we have also found significant down-regulation of citrate cycle and oxidative phosphorylation pathway associated proteins at 72 h of infection. Some components of glycolytic machinery like Phosphoenolpyruvate carboxykinase, Fructose bis-phosphate aldolase were also downregulated by >2-folds at late infection stages. Yet, other glycolytic components like Hexokinase, Fructose-1,6-bisphosphatase, Triosephosphate isomerase, Phosphoglyceromutase, Pyruvate kinase etc showed less suppression. Similarly, though propanoate metabolism represents one of the most down-regulated biological pathways, metabolism of sugars like sucrose, fructose, mannose and galactose were not much affected in our study. An analysis of *S. frugiperda* metabolic activity after baculovirus infection has earlier reported slightly elevated rates of sucrose and maltose uptake at 48–72 h of infection, providing significance to our analysis^51^. Notably, we also observed the levels of UDP-glucosyltransferases, UDP-glucose 4-epimerase, and some components of pentose and glucoronate-interconversions to be enriched in the constant category. In insects, conjugation of moulting hormone, ecdysteroid, with sugars by baculoviral enzyme, Ecdysteroid UDP-glucosyltransferase, is considered to prevent moulting and support viral amplification^52^. In *S. frugiperda* larvae, it was found that UDP-galactose and UDP-glucose were the most favoured substrates^53^. In this context, it would be interesting to find the significance of higher levels of galactose and UDP-sugar metabolism in infected insects.

Although the current study substantiates the global transcriptional and translational repression model, KEGG pathway analysis suggests significant differences in the extent of regulation of different pathways during the course of infection. It would thus be fascinating to understand the mechanisms controlling this specificity. One such proposed mechanism could be the selective ubiquitination mediated by E3 ligases as discussed before. Another mechanism capable of selective manipulation is overexpression of small regulatory RNAs such as miRNAs during the infection. We have previously demonstrated the expression of *Splt*NPV encoded miRNAs after 12 h of infection and identified their targets in *Sf21* cells^19^. Since the expression of viral miRNAs was coherent with the proposed initiation of protein shutdown, we were interested to find a correlation between both the studies. Upon comparison of predicted *Sf21* targets of validated *Splt*NPV miRNAs with the proteome data, it was observed that 117 predicted targets were downregulated in proteome analysis, while one protein was upregulated. qPCR analysis further confirmed the down-regulation of most of these genes. To know whether the viral miRNAs affected host gene expression, we analysed the effect of two viral miRNA mimics in *Sf21* cells. Nine of the host genes were found to be targeted by these viral miRNAs. Interestingly, both viral miRNAs were found to target enzymes in OXPHOS pathway like NADH-ubiquinone oxidoreductase Fe-S protein 7, FAD-dependent oxidoreductase and NADH dehydrogenase [ubiquinone] 1 subunit C2, thereby indicating that baculoviruses might be employing miRNAs to efficiently down-regulate OXPHOS pathway. In addition, 11684_3p was found to specifically inhibit Translation initiation factor 4a which is required to unwind mRNA secondary structure and to prepare them for binding to ribosomes for initiation of translation^54^. Besides these factors, replication factor C4, which is required for loading of proliferating cell nuclear antigen to DNA and facilitating the highly processive DNA replication, was also found to be targeted by miRNA 11684_3p^55^. Previously, *Heliothis virescens* ascovirus miRNA-1 has been reported to regulate transcriptional levels of DNA polymerase I and thus regulate its own replication and late stages of infection^56^. Here, we observe regulation of other replication proteins as well.

To conclude, the present work describes a comprehensive analysis of Lepidopteran insect proteome and its modulation following baculoviral infection. The work has revealed molecular basis for baculovirus-induced physiological phenomena and has led to the elucidation of novel aspects of baculoviral host interactions. The study thus provides a systemic analysis of proteome changes in *Sf21* cells at different stages of viral infection and role(s) of viral miRNAs in regulating the expression of host proteins. Overall, this study provide meaningful insights into the dynamics of host responses during viral infection as well as the conserved viral mechanisms in controlling host cellular machinery.

## Methods

### Cell Culture and *Splt*NPV infection

For *Splt*NPV infection, *Sf21* cells were maintained as a monolayer in serum-free TNM-FH insect medium at 27 °C. 1 × 10^7^ *Sf21* cells were infected with wild type *Splt*NPV (MOI = 2) for two hours in duplicates and harvested after 12 h and 72 h interval. Mock infected *Sf21* cells were used as a control and were also processed similarly in duplicates.

### Trypsin digestion, TMT-labelling and LC-MS/MS analysis

After 12 h and 72 h, the cells were harvested, lysed in lysis buffer (20 mM Tris-Cl, pH 8.0, 150 mM NaCl, 1 mM DTT, 0.015% Nonidet P-40 and 8 M Urea) and centrifuged at 13000 rpm for 30 min. The protein concentration in the supernatant was estimated using Bradford assay. The proteins were subjected to Trypsin digestion and subsequent TMT labelling as per the manufacturer’s protocol (Thermofisher Scientific Inc., USA) using 6plex-TMT labelling kit. Equal amount (100 µg) from each of the sample was reduced with DTT for 1 h at RT followed by alkylation using IAA for additional 1 h at RT. After reduction and alkylation, the protein samples were acetone precipitated and re-dissolved in 100 mM TEAB buffer. These proteins were then subjected to overnight trypsin digestion at 37 °C (Trypsin gold, Promega, USA). The peptides from control and infected cells (in duplicates each) from 12 hpi and 72 hpi were labelled individually with different labels for 1 h at RT. The labelling reaction was stopped by addition of hydroxylamine for 10 minutes at RT. The labelled peptides from control and infected cells were mixed together and vacuum dried. For internal experimental control, 20 fmol of BSA digest were spiked in each sample before labelling.

The mixed peptide pools from both the samples were re-suspended in 0.1% formic acid in 95% acetonitrile and fractionated into 15 fractions using HILIC chromatography. Each fraction was separately analysed in duplicates on LC-MS/MS. Tandem mass spectrometry experiments were performed using the Easy-nLC 1000 HPLC system (ThermoFisher Scientific, Waltham, MA) via nano-electrospray ion source connected to hybrid Orbitrap Velos Pro mass spectrometer (Thermo Fisher Scientific, Waltham, MA). The nano-LC was equipped with Acclaim^®^ PepMap100 C18 column (75 µm × 2 cm) pre-column packed with 3 μm C18 resin which was further connected to Acclaim^®^ PepMap100 C18 column (50 µm × 5 cm) analytical column (Dionex, USA) packed with 2 μm C18 beads.

Peptides were separated by a 120 min gradient of 5% buffer B to 90% buffer B (Buffer B 0.1% Formic Acid in 95% Acetonitrile; Buffer A: 0.1% Formic Acid in 5% Acetonitrile) with a flow-rate of 300 nl/min. Peptides eluting from the column were electro-sprayed directly into the Orbitrap velos MS with a spray voltage of 1.4 kV. Data acquisition was performed in a data-dependent mode to automatically switch between MS and MS2. Precursor ion spectra were acquired in Full-scan mode with a resolution of 60,000 in Orbitrap. Top 20 parent ions were sequentially isolated, fragmented using high-energy collision dissociation and acquired at a resolution of 7500. A dynamic exclusion of ions previously sequenced within 90 s was applied. All unassigned charge states and singly charged ions were excluded from sequencing. A minimum of 1000 counts was required for MS2 selection. Accurate mass measurements were enabled with the lock mass option on in both MS1 as well as MS2.

### Data analysis and functional annotation

The raw data were imported to Proteome Discoverer 1.4 (ThermoFisher, Waltham, MA, USA) and the proteins were identified by searching against the recently reported *Sf21* genome assembly proteome databases using SEQUEST algorithm^17^. The peptide matches were validated using Percolator at 5% FDR. The search parameters included a mass tolerance of 20 ppm for the precursors and 0.1 Da for fragmented ions. Upto 2 missed cleavages were allowed for trypsin specificity. Carbamidomethyl (C), Deamidation (NQ) and 6-plex TMT label (N-terminus and K) were set as variable modifications. The differential expression of proteins was calculated using reporter ion quantification approach available in Proteome Discoverer using default parameters. The mass spectrometry proteomics data were deposited to the ProteomeXchange Consortium via the PRIDE partner repository^57^.

Functional annotation of the identified proteins was performed using KEGG Automation Annotation Server (KAAS). Protein sequences were searched on the basis of their blast homology with KEGG genes database and KEGG orthologues, BRITE hierarchies and pathway maps were generated^58–61^.

### miRNA mimic transfection

*mir*Vana^TM^ miRNA mimics were ordered for *Splt*NPV miRNAs 11684_3p and 11698_3p (ThermoFisher Scientific). Both the mimics were transfected in 70–80% confluent *Sf*21 cells at three concentrations i.e 10 µM, 50 µM and 100 µM using recommended concentration of Cellfectin II reagent (ThermoFisher Scientific). The transfection was carried out at room temperature for four hours in serum-free TNM-FH medium (Sigma-Aldrich) with intermittent shaking. Scrambled miRNA mimic was also transfected at a concentration of 10 µM, 50 µM and 100 µM in a similar fashion. After four hours, the medium was replaced with 10% FBS containing TNM-FH medium and the cells were maintained for 48 h.

### qPCR validation and Western blot

To validate the proteome data, total RNA was extracted from mock infected and *Splt*NPV infected *Sf*21 cells at 12 h and 72 h of infection using TRIzol reagent (Invitrogen). This was followed by DNaseI digestion (Invitrogen) to remove genomic DNA contamination. One-step qPCR was used to determine the level of 32 selected *Sf*21 genes using Verso SYBR Green 1-Step qRT-PCR ROX mix kit (Thermo Scientific). A list of oligonucleotide primers used for this study is provided in Table S4. 100 ng of DNase I treated total RNA from 12 h and 72 h mock and *Splt*NPV infected *Sf*21 cells was used as a template. qPCR reaction was set up in triplicates for each gene with the following conditions: reverse transcription at 50 °C for 15 min, enzyme activation at 95 °C for 15 min, followed by 40 cycles of denaturation (95 °C)-15s, annealing (respective temperature)-30s and extension (72 °C)-30s. No Template control sample was run for each gene to ensure the fidelity of experiment. Relative expression levels of the mRNAs were calculated by normalizing against 28 S RNA levels using 2^−∆∆Ct^ method^62^.

To check relative expression of gene transcripts after administration of increasing concentration of miRNA mimics, total RNA of *Sf*21 cells was extracted after 48 h of transfection with 10 µM, 50 µM and 100 µM of both miRNA mimics. Total RNA was also extracted from 10 µM, 50 µM and 100 µM of scrambled miRNA control as well as mock transfection. After DNaseI digestion, one step qPCR was performed for selected transcripts as described before. A list of oligonucleotide primers used for this study is provided in Table S4. Normalization of expression was done using 28 S RNA.

Western blot was done according to the method described by Towbin *et al*. with minor modifications^63^. Mock-infected and *Splt*NPV infected *Sf21* cells were collected after 12 h and 72 h and lysed by sonication in the following buffer: 50 mM Tris-Cl (pH 7.5), 150 mM NaCl, 1X cOmplete Protease inhibitor cocktail (Roche). The lysate was centrifuged at 13000 rpm for 15 min and the supernatant was estimated for protein concentration using Bradford Assay. 50 µg of crude protein lysate was loaded for each sample along with PageRuler/Bio-Rad Pre-stained Protein Ladder for molecular weight determination and visualization of protein transfer onto the membrane. Mini Transblot Electrophoretic Cell apparatus (Bio-Rad) was used to transfer the proteins from gel onto nitrocellulose membrane. Electroblotting was performed in the presence of 1X Native PAGE buffer at a constant current of 170 mA for 1 hour. The membrane after transfer was rinsed briefly in 1X PBS and incubated in blocking solution (3% BSA in PBS buffer) for 1 h with gentle shaking at room temperature. The blocking solution was replaced with 1:2500 dilution of primary antibody solution [polyclonal Anti-Histone H3 raised in rabbit (Abcam), 1 mg/ml], and incubated for 1 h at room temperature with gentle shaking. Thereafter, the membrane was washed thrice with PBS buffer and 0.05% Tween-20 for 10 min each. After washing, alkaline-phosphatase conjugated secondary antibody solution (ThermoFisher Scientific, 0.6 mg/ml at 1:7500 dilution in PBS buffer) was added to the membrane and incubated for 1 h at room temperature with constant shaking. After that, membrane was washed thrice with PBS buffer and 0.05% Tween-20 for 10 min each. The protein-antibody complex was developed by adding 2–3 ml of Western Blue (Promega) stabilized substrate for alkaline phosphatase.

### Data availability

The mass spectrometry datasets generated during the current study have been deposited to the ProteomeXchange Consortium via the PRIDE partner repository with the dataset identifier PXD005870^57^.

## Electronic supplementary material

Supplementary Information
