# Supplementary material for: Quantitative proteomics of Sf21 cells during Baculovirus infection reveals progressive host proteome changes and its regulation by viral miRNA
Source: Sci Rep. 2017 Sep 7;7:10902. doi: 10.1038/s41598-017-10787-z (PMC5589936; doi:10.1038/s41598-017-10787-z)
Supplement: Supplementary file 1 — Supplementary Information [file 41598_2017_10787_MOESM1_ESM.doc]

**Quantitative proteomics of *Sf21* cells during Baculovirus infection reveals progressive host proteome changes and its regulation by viral miRNA.**

**Nishtha Nayyar, Inderjeet Kaur, Pawan Malhotra, Raj K. Bhatnagar**

Supplementary Table S1: A list of *Sf*21 proteins detected upon *Splt*NPV infection and their expression levels with respect to mock infection at 12h and 72h. The ratio denoted here is an average ratio of four replicates (two biological and two technical).

| **S.No** | **Protein Description** | **12hpi Avg Ratio [Infected/Mock]** | **72 hpi Avg ratio [Infected/Mock]** |
| --- | --- | --- | --- |
|  | PREDICTED: ubiquitin-protein ligase E3B-like - [512923562_XP_004930382.1] | #N/A | 22.895296 |
|  | PREDICTED: hypothetical protein LOC100166252 - [328703211_XP_001949790.2] | 3.144870 | 13.328633 |
|  | PREDICTED: tyrosine-protein phosphatase 69D-like isoform X1 - [498950434_XP_004523016.1] | #N/A | 12.251691 |
|  | PREDICTED: similar to transposase | 1.2873378 | 10.7381750 |
|  | PREDICTED: beta 1,3-galactosyl transferase | #N/A | 10.723587 |
|  | PREDICTED: uncharacterized protein LOC100888373 - [390332071_XP_003723412.1] | 1.11072827 | 8.9564447 |
|  | PREDICTED: leucine-rich repeats and immunoglobulin-like domains protein 3-like - [512893545_XP_004923208.1] | #N/A | 8.5534362 |
|  | PREDICTED: similar to sentrin/sumo-specific protease senp7 - [189237239_XP_971090.2] | 1.17476895 | 7.9679583 |
|  | Predicted: lactosylceramide 4-alpha-galactosyltransferase-like | #N/A | 7.4549502 |
|  | snap-scaffold3835_size39147-abinit-gene-0.3-mRNA-1 | #N/A | 7.0735387 |
|  | chitinase precursor - [169234932_NP_001108407.1] | #N/A | 6.7823058 |
|  | ecdysis triggering hormone receptor isoform B - [288558748_NP_001165737.1] | #N/A | 5.1047195 |
|  | PREDICTED: mediator of RNA polymerase II transcription subunit 27-like - [512896296_XP_004923877.1] | #N/A | 4.2971606 |
|  | cyclin L1 - [239835732_NP_001155189.1] | 1.23350683 | 4.1865651 |
|  | PREDICTED: uncharacterized protein LOC101740482 - [512889305_XP_004922243.1] | #N/A | 4.1198106 |
|  | PREDICTED: slit homolog 2 protein-like - [512886322_XP_004921739.1] | #N/A | 3.5403167 |
|  | PREDICTED: uncharacterized protein LOC101746397 - [512920801_XP_004929723.1] | #N/A | 3.3549365 |
|  | Pao retrotransposon peptidase family protein - [339234042_XP_003382138.1] | 19.575042 | 3.1842968 |
|  | PREDICTED: SAP30-binding protein-like - [512939751_XP_004934317.1] | 1.13078067 | 3.1268762 |
|  | PREDICTED: ionotropic glutamate receptor | #N/A | 2.9711609 |
|  | PREDICTED: carboxylesterase I | #N/A | 2.911735 |
|  | PREDICTED: laminin subunit beta-1-like - [449666107_XP_002168125.2] | 1.18281278 | 2.7905998 |
|  | PREDICTED: Wash complex subunit Strumpellin | 1.17552399 | 2.7125174 |
|  | PREDICTED: leucine-rich repeat protein lrrA-like - [512887823_XP_004921986.1] | #N/A | 2.6023705 |
|  | snap-scaffold2135_size34432-abinit-gene-0.1-mRNA-1 | #N/A | 2.3996433 |
|  | PREDICTED: uncharacterized protein LOC101741879 - [512923504_XP_004930368.1] | #N/A | 2.2221911 |
|  | snap-scaffold7631_size20398-abinit-gene-0.3-mRNA-1 | #N/A | 2.1580581 |
|  | PREDICTED: uncharacterized protein LOC101736405 - [512911048_XP_004927320.1] | #N/A | 2.0545342 |
|  | snap-scaffold3241_size48869-abinit-gene-0.6-mRNA-1 | #N/A | 2.0390689 |
|  | snap-scaffold4164_size25259-abinit-gene-0.2-mRNA-1 | 1.06998736 | 2.0306261 |
|  | PREDICTED: uncharacterized protein LOC101737844 - [512886606_XP_004921786.1] | #N/A | 2.0002948 |
|  | PREDICTED: zinc finger protein 268-like - [512897377_XP_004924137.1] | 1.06097939 | 1.8961437 |
|  | PREDICTED: glycosaminoglycan xylosylkinase-like - [512923151_XP_004930283.1] | #N/A | 1.8939282 |
|  | CRE-HID-1 protein - [308495231_XP_003109804.1] | #N/A | 1.8821093 |
|  | PREDICTED: GPI ethanolamine phosphate transferase 2-like - [512890317_XP_004922497.1] | 1.22228591 | 1.8493787 |
|  | snap-scaffold246_size74815-abinit-gene-0.15-mRNA-1 | #N/A | 1.7761501 |
|  | PREDICTED: formin like protein | #N/A | 1.7578312 |
|  | PREDICTED: GTP-binding protein 5-like - [512897595_XP_004924191.1] | #N/A | 1.6441993 |
|  | PREDICTED: uncharacterized protein LOC101741629 - [512910118_XP_004927089.1] | #N/A | 1.5085013 |
|  | sorbitol dehydrogenase - [112983008_NP_001037592.1] | 1.09122087 | 1.5059454 |
|  | snap-scaffold168_size160893-abinit-gene-0.15-mRNA-1 | #N/A | 1.4699514 |
|  | PREDICTED: thiamin pyrophosphokinase 1-like - [512927042_XP_004931235.1] | #N/A | 1.421646 |
|  | PREDICTED: zinc finger protein jing | #N/A | 1.4093401 |
|  | PREDICTED: ribonuclease Oy-like - [512919647_XP_004929436.1] | 1.13189391 | 1.3965588 |
|  | snap-scaffold5970_size23108-abinit-gene-0.0-mRNA-1 | 1.35788101 | 1.3908503 |
|  | PREDICTED: uncharacterized protein LOC100902345 - [391332386_XP_003740616.1] | 1.12741917 | 1.3829479 |
|  | PREDICTED: cryptochrome-1-like isoform X1 - [498980192_XP_004529289.1] | 2.1783757 | 1.3194158 |
|  | snap-scaffold9702_size13818-abinit-gene-0.3-mRNA-1 | 1.17438578 | 1.2800016 |
|  | general control of amino acid synthesis-like protein - [114053285_NP_001040126.1] | 1.23923641 | 1.2289556 |
|  | PREDICTED: ubiquitin-like protein 7-like - [512920365_XP_004929615.1] | 1.17079507 | 1.1784409 |
|  | PREDICTED: neuroligin-2-like - [512896307_XP_004923879.1] | #N/A | 1.1688515 |
|  | PREDICTED: probable nuclear transport factor 2-like isoform X1 - [512893707_XP_004923247.1] | 1.07000205 | 1.1653771 |
|  | PREDICTED: hypothetical protein LOC100634292 - [340384903_XP_003390950.1] | #N/A | 1.1518369 |
|  | PREDICTED: uncharacterized protein LOC101746638 - [512929167_XP_004931757.1] | 1.1118295 | 1.1378788 |
|  | snap-scaffold13349_size1781-abinit-gene-0.0-mRNA-1 | #N/A | 1.1341264 |
|  | PREDICTED: myelin gene regulatory factor | #N/A | 1.1284333 |
|  | PREDICTED: LOW QUALITY PROTEIN: 5'-AMP-activated protein kinase subunit gamma-like - [512926113_XP_004931004.1] | #N/A | 1.1244362 |
|  | CRE-PGP-3 protein - [308494977_XP_003109677.1] | #N/A | 1.1184717 |
|  | PREDICTED: serine-rich adhesin for platelets-like isoform X1 - [498952359_XP_004523327.1] | 1.23914975 | 1.1001915 |
|  | PREDICTED: RNA helicase like protein tudor domain containing | 1.18268821 | 1.098 |
|  | glutathione peroxidase - [114051564_NP_001040104.1] | 1.02058727 | 1.0878017 |
|  | heat shock protein hsp20.8 - [148298693_NP_001091794.1] | 1.44461961 | 1.0741987 |
|  | PREDICTED: uncharacterized protein LOC101739789 - [512916356_XP_004928621.1] | #N/A | 1.0568531 |
|  | PREDICTED: translation initiation factor eIF-2B subunit epsilon-like isoform X1 - [512913273_XP_004927856.1] | 1.13488966 | 1.0491686 |
|  | PREDICTED: recombination repair protein | 1.13483177 | 1.049 |
|  | annexin B13 - [112983539_NP_001036881.1] | 1.12414978 | 1.0474497 |
|  | PREDICTED: ubiquitin carboxyl terminal hydrolase | #N/A | 1.0419749 |
|  | PREDICTED: protein odr-4 homolog - [512929757_XP_004931902.1] | 1.13850582 | 1.0278952 |
|  | PREDICTED: serine/threonine-protein kinase N-like - [512932289_XP_004932509.1] | #N/A | 1.0249872 |
|  | PREDICTED: signal transducer and activator of transcription | 1.23281977 | 1.022 |
|  | PREDICTED: DNA repair protein xrcc4-like - [512887263_XP_004921894.1] | #N/A | 1.0208847 |
|  | aspartylglucosaminidase - [112982715_NP_001037686.1] | 1.02479082 | 1.017817 |
|  | PREDICTED: uncharacterized protein LOC101739665 - [512887213_XP_004921885.1] | #N/A | 1.0132748 |
|  | PREDICTED: slowpoke-binding protein-like - [512898675_XP_004924455.1] | #N/A | 1.0129461 |
|  | PREDICTED: catenin delta-2-like - [512909191_XP_004926861.1] | 1.2063784 | 1.0051602 |
|  | PREDICTED: niemann-Pick C1 protein-like - [383847243_XP_003699264.1] | 1.12246736 | 0.9975094 |
|  | PREDICTED: UDP-N-acetylglucosamine transferase subunit ALG13 homolog - [512902912_XP_004925490.1] | 1.17400735 | 0.994416 |
|  | PREDICTED: transforming growth factor beta-1-induced transcript 1 protein-like isoform X1 - [512902451_XP_004925379.1] | 1.30576584 | 0.9879801 |
|  | PREDICTED: uncharacterized threonine-rich GPI-anchored glycoprotein PJ4664.02 - [512933086_XP_004932704.1] | 1.22874877 | 0.9804286 |
|  | PREDICTED: uncharacterized protein C4orf29 homolog - [512918153_XP_004929062.1] | #N/A | 0.9788204 |
|  | PREDICTED: protein lethal(2)essential for life-like - [512937030_XP_004933665.1] | 1.16420563 | 0.9760055 |
|  | cAMP responsive element binding protein - [114051788_NP_001040181.1] | 1.50357 | 0.9744848 |
|  | PREDICTED: prenylated Rab acceptor protein 1-like isoform X3 - [512899382_XP_004924630.1] | #N/A | 0.974052 |
|  | PREDICTED: synaptic vesicle 2-related protein-like - [512908135_XP_004926607.1] | #N/A | 0.9688096 |
|  | PREDICTED: sentrin-specific protease 8-like - [512929296_XP_004931786.1] | #N/A | 0.967028 |
|  | PREDICTED: GPI inositol deacylase | 2.26821687 | 0.967 |
|  | snap-scaffold1742_size39014-abinit-gene-0.4-mRNA-1 | #N/A | 0.9662138 |
|  | PREDICTED: apolipoprotein D-like - [512909266_XP_004926879.1] | 1.10034065 | 0.9630391 |
|  | PREDICTED: ATPase family AAA domain-containing protein 2B-like - [512929443_XP_004931822.1] | 1.13209396 | 0.9599943 |
|  | DNA topoisomerase 2 - [112983266_NP_001037009.1] | 1.3089446 | 0.9502572 |
|  | PREDICTED: dihydrofolate reductase-like - [512912124_XP_004927579.1] | 1.07353957 | 0.9497 |
|  | PREDICTED: fascin 1/2 | 1.09712975 | 0.9488636 |
|  | PREDICTED: derlin-2-like - [512930786_XP_004932146.1] | 1.28981706 | 0.9484925 |
|  | PREDICTED: unc-112-related protein-like isoform 2 - [380026011_XP_003696755.1] | 1.22180324 | 0.9471683 |
|  | PREDICTED: WD repeat-containing protein 26-like - [512909528_XP_004926943.1] | 1.11830215 | 0.9435422 |
|  | PREDICTED: aminoacylase-1-like - [512907910_XP_004926560.1] | 1.1635137 | 0.9431918 |
|  | snap-scaffold3373_size25960-abinit-gene-0.8-mRNA-1 | #N/A | 0.941824 |
|  | PREDICTED: restin homolog - [512924098_XP_004930515.1] | 1.21666364 | 0.937079 |
|  | ubiquitin-related modifier 1 homolog - [114052050_NP_001040521.1] | 1.24601469 | 0.9299248 |
|  | heat shock protein hsp 19.9 - [112983420_NP_001036984.1] | 1.24148681 | 0.9255979 |
|  | PREDICTED: UDP-glucose 6-dehydrogenase-like - [512901472_XP_004925143.1] | 1.51743951 | 0.9186663 |
|  | Hypothetical protein CBG18156 - [268574422_XP_002642188.1] | 1.25587068 | 0.9163502 |
|  | PREDICTED: U2 small nuclear ribonucleoprotein auxiliary factor 35 kDa subunit-related protein 2-like - [512919437_XP_004929383.1] | 1.18089558 | 0.9145962 |
|  | PREDICTED: solute carrier family 5 | #N/A | 0.9131672 |
|  | PREDICTED: dynein intermediate chain 3, ciliary-like - [512932099_XP_004932462.1] | #N/A | 0.9110479 |
|  | PREDICTED: similar to pol polyprotein - [189242191_XP_001810599.1] | 1.50297727 | 0.9109119 |
|  | PREDICTED: putative riboflavin kinase-like - [512907423_XP_004926437.1] | 1.12459866 | 0.909993 |
|  | PREDICTED: transcription initiation factor TFIIE subunit alpha | 1.20927683 | 0.9092499 |
|  | PREDICTED: L-xylulose reductase-like isoform X1 - [512905210_XP_004925887.1] | 1.1473691 | 0.9073554 |
|  | PREDICTED: putative sulfiredoxin-like - [512916586_XP_004928676.1] | 1.08229571 | 0.8996544 |
|  | putative CBS domain pair - [339242935_XP_003377393.1] | 1.34396169 | 0.8992806 |
|  | PREDICTED: general transcription factor IIH subunit 3-like - [512910757_XP_004927249.1] | 1.13275466 | 0.898017 |
|  | PREDICTED: heat shock protein Hsp-16.1/Hsp-16.11-like - [512911176_XP_004927349.1] | #N/A | 0.8957499 |
|  | PREDICTED: uridine phosphorylase 1-like isoform X4 - [512903030_XP_004925519.1] | 0.96774714 | 0.8949285 |
|  | PREDICTED: peptidyl-prolyl cis-trans isomerase FKBP2-like - [512927214_XP_004931279.1] | 1.12589631 | 0.8923837 |
|  | heat shock protein hsp 19.9 - [112983420_NP_001036984.1] | 1.22364377 | 0.8881786 |
|  | exosome complex exonuclease Rrp40 - [237648986_NP_001153670.1] | 1.05471498 | 0.888073 |
|  | PREDICTED: RE1-silencing transcription factor-like - [512915784_XP_004928474.1] | 1.22814706 | 0.8877641 |
|  | PREDICTED: vam6/Vps39-like protein-like - [512914991_XP_004928279.1] | 1.18622112 | 0.8877301 |
|  | autophagy related protein Atg8 - [114052412_NP_001040244.1] | 1.13937136 | 0.8876188 |
|  | PREDICTED: origin recognition complex subunit 4-like - [512935725_XP_004933344.1] | 1.15380184 | 0.8872177 |
|  | autophagy related protein Atg12-like protein - [215820608_NP_001135963.1] | #N/A | 0.8862366 |
|  | PREDICTED: replication factor C subunit 1-like - [512892886_XP_004923043.1] | 1.27655569 | 0.8840669 |
|  | partner of Y14 and mago - [114053219_NP_001040288.1] | 1.12115993 | 0.8801458 |
|  | snap-scaffold1665_size66225-abinit-gene-0.14-mRNA-1 | #N/A | 0.8778919 |
|  | PREDICTED: uncharacterized protein LOC101738067 - [512890764_XP_004922569.1] | 1.57249117 | 0.8768171 |
|  | PREDICTED: LOW QUALITY PROTEIN: transcription initiation factor TFIID subunit 11-like - [512929198_XP_004931764.1] | 1.27069843 | 0.8737951 |
|  | PREDICTED: RNA-directed DNA polymerase from mobile element jockey-like - [328707593_XP_003243439.1] | #N/A | 0.872304 |
|  | snap-scaffold2822_size34332-abinit-gene-0.5-mRNA-1 | #N/A | 0.8698837 |
|  | PREDICTED: transmembrane protein 129-like - [512907171_XP_004926377.1] | 0.92038835 | 0.8676317 |
|  | PREDICTED: spectrin beta chain, non-erythrocytic 5-like - [512901464_XP_004925141.1] | 1.1595186 | 0.8676175 |
|  | PREDICTED: alpha-tubulin N-acetyltransferase 1-like isoform X3 - [512933384_XP_004932778.1] | #N/A | 0.8675314 |
|  | replication protein A3 - [112983074_NP_001036940.1] | 1.19226802 | 0.8667074 |
|  | PREDICTED: similar to orf - [189238306_XP_001810526.1] | #N/A | 0.8648822 |
|  | PREDICTED: general transcription factor IIH subunit 2-like isoform X1 - [512930578_XP_004932094.1] | 1.22653095 | 0.8589289 |
|  | PREDICTED: similar to Drop CG1897-PA - [91091014_XP_975059.1] | #N/A | 0.8561434 |
|  | PREDICTED: BRCA2 | 1.1288422 | #N/A |
|  | snap-scaffold4806_size13493-abinit-gene-0.0-mRNA-1 | 0.90499299 | 0.8528242 |
|  | PREDICTED: glucose-6-phosphate 1-epimerase-like, partial - [512922052_XP_004930025.1] | #N/A | 0.8513084 |
|  | myosin light polypeptide 9 isoform B - [160333484_NP_001103768.1] | 1.11529281 | 0.8483462 |
|  | PREDICTED: aldose reductase-like - [512936939_XP_004933642.1] | 1.17621036 | 0.8477172 |
|  | PREDICTED: deoxyuridine 5'-triphosphate nucleotidohydrolase-like - [512927369_XP_004931317.1] | 1.12023226 | 0.8465406 |
|  | lysophospholipase - [114052571_NP_001040255.1] | 1.02799212 | 0.8456137 |
|  | PREDICTED: spectrin beta chain, non-erythrocytic 5-like - [512901464_XP_004925141.1] | 1.13129315 | 0.8446239 |
|  | Rad51 homolog - [112984536_NP_001037484.1] | #N/A | 0.8443133 |
|  | acyl-CoA oxidase - [170033157_XP_001844445.1] | #N/A | 0.8407253 |
|  | PREDICTED: gamma-tubulin complex component 4-like - [512936027_XP_004933418.1] | #N/A | 0.8393648 |
|  | PREDICTED: DNA excision repair protein ERCC-1-like isoform X1 - [512926643_XP_004931137.1] | #N/A | 0.8381678 |
|  | PREDICTED: uncharacterized protein LOC101736189 - [512917416_XP_004928881.1] | #N/A | 0.835931 |
|  | Annexin IX isoform A - [112983958_NP_001036841.1] | 1.02211271 | 0.8356912 |
|  | PREDICTED: myotubularin-related protein 8-like - [512891547_XP_004922711.1] | #N/A | 0.8336412 |
|  | endoplasmic reticulum protein - [114051848_NP_001040189.1] | 1.18247129 | 0.8326936 |
|  | PREDICTED: NECAP-like protein CG9132-like - [512893746_XP_004923256.1] | 1.14672935 | 0.8322894 |
|  | glutathione S-transferase sigma 2 - [160333678_NP_001036994.1] | 0.97839486 | 0.8317959 |
|  | PREDICTED: probable methylthioribulose-1-phosphate dehydratase-like - [512886096_XP_004921704.1] | #N/A | 0.8316652 |
|  | PREDICTED: 1-phosphatidylinositol-4-phosphate 5-kinase | 1.25080534 | 0.830813 |
|  | glutathione S-transferase delta 2 - [112983444_NP_001036974.1] | 1.57182542 | 0.8305581 |
|  | PREDICTED: diamine N-acetyltransferase | 1.11071439 | 0.8272891 |
|  | PREDICTED: replication factor C subunit 5 | 1.31497267 | 0.827 |
|  | replication protein A1 - [112983132_NP_001036938.1] | 1.22479174 | 0.8256003 |
|  | PREDICTED: serine/threonine-protein phosphatase 2A activator-like isoform X1 - [512892704_XP_004922997.1] | 1.15995126 | 0.8254641 |
|  | signal transducer and activator of transcription - [255652895_NP_001157388.1] | #N/A | 0.8214554 |
|  | PREDICTED: glucose-induced degradation protein 4 homolog isoform X2 - [512932297_XP_004932511.1] | 1.21291192 | 0.8204932 |
|  | centrin - [114051744_NP_001040177.1] | 1.18039793 | 0.8200451 |
|  | PREDICTED: protein ACN9 homolog, mitochondrial-like isoform X1 - [512933573_XP_004932824.1] | 1.25259353 | 0.817188 |
|  | PREDICTED: uncharacterized protein LOC101744002 - [512930364_XP_004932049.1] | 1.44256603 | 0.8170413 |
|  | PREDICTED: alpha-crystallin B chain-like - [512909203_XP_004926864.1] | 1.27567231 | 0.8160657 |
|  | PREDICTED: hypothetical protein LOC100571647 - [328711860_XP_003244662.1] | 1.26608188 | 0.8151368 |
|  | activator of 90 kDa heat shock protein ATPase homolog 1 - [112982695_NP_001036909.1] | 1.19413958 | 0.8148739 |
|  | PREDICTED: uncharacterized protein LOC101737962 - [512924066_XP_004930507.1] | 1.1515551 | 0.814633 |
|  | PREDICTED: replication factor C subunit 3/5 | 1.23784814 | 0.8131636 |
|  | 6-pyruvoyltetrahydropterin synthase - [114051562_NP_001040103.1] | 0.93814707 | 0.8125076 |
|  | PREDICTED: Golgi to ER traffic protein 4 homolog - [512905190_XP_004925882.1] | 1.34827695 | 0.8121709 |
|  | mRNA cap-binding protein eIF4E - [114051309_NP_001040379.1] | 1.19414955 | 0.8117153 |
|  | glutathione S-transferase-like - [242247543_NP_001156113.1] | 1.16480783 | 0.8115629 |
|  | RNA polymerase Rpb1 - [256083198_XP_002577836.1] | 1.19493851 | 0.8104458 |
|  | achintya - [237648964_NP_001153660.1] | #N/A | 0.8091001 |
|  | PREDICTED: cysteine protease ATG4B-like - [512918831_XP_004929228.1] | #N/A | 0.8080098 |
|  | PREDICTED: chromatin assembly factor 1 subunit B-like - [383847607_XP_003699444.1] | 1.85165767 | 0.8059839 |
|  | PREDICTED: E3 ubiquitin-protein ligase RNF13-like - [512892941_XP_004923057.1] | 0.96851232 | 0.8040343 |
|  | BWK-1-like protein - [114051135_NP_001040311.1] | 1.21865753 | 0.8034753 |
|  | PREDICTED: similar to DnaJ homolog subfamily A member 1 - [91077138_XP_971446.1] | 1.23003713 | 0.8003958 |
|  | phosphoserine aminotransferase 1 - [114052677_NP_001040269.1] | #N/A | 0.7997869 |
|  | PREDICTED: ornithine aminotransferase, mitochondrial-like isoform X1 - [524868667_XP_005091134.1] | 1.21157313 | 0.7989673 |
|  | PREDICTED: 39S ribosomal protein L13, mitochondrial-like - [512889959_XP_004922416.1] | 1.1249018 | 0.7983741 |
|  | snap-scaffold4691_size11851-processed-gene-0.1-mRNA-1 | | 0.7977637 |
|  | PREDICTED: DNA primase small subunit-like - [512893742_XP_004923255.1] | #N/A | 0.7972257 |
|  | PREDICTED: sequestosome-1-like isoform X1 - [512931618_XP_004932348.1] | 1.53595971 | 0.7965538 |
|  | PREDICTED: LOW QUALITY PROTEIN: ribosomal protein S6 kinase beta-2-like - [498965741_XP_004525646.1] | 1.10553816 | 0.7961415 |
|  | PREDICTED: ester hydrolase C11orf54 homolog - [512885211_XP_004921557.1] | #N/A | 0.7961366 |
|  | PREDICTED: regucalcin-like isoform X1 - [512924953_XP_004930719.1] | 1.05091409 | 0.7949143 |
|  | PREDICTED: spermidine synthase-like isoform X1 - [512899761_XP_004924726.1] | 1.11313098 | 0.7946463 |
|  | PREDICTED: uncharacterized protein KIAA1841-like - [512917857_XP_004928990.1] | #N/A | 0.7946183 |
|  | PREDICTED: probable 2-oxoglutarate dehydrogenase E1 component DHKTD1 homolog, mitochondrial-like isoform X1 - [512894908_XP_004923544.1] | #N/A | 0.7930784 |
|  | PREDICTED: uncharacterized protein LOC101747151 - [512936588_XP_004933554.1] | 1.25395527 | 0.7929024 |
|  | thiol peroxiredoxin - [112982996_NP_001037083.1] | 1.15676809 | 0.791843 |
|  | snap-scaffold10945_size5422-processed-gene-0.0-mRNA-1 | #N/A | 0.7886213 |
|  | PREDICTED: spectrin beta chain, non-erythrocytic 5-like - [512901464_XP_004925141.1] | 1.1632047 | 0.7885351 |
|  | PREDICTED: growth factor receptor-binding protein 2 | 1.18787386 | 0.7881774 |
|  | glutathione S-transferase sigma 2 - [160333678_NP_001036994.1] | 0.98262938 | 0.7860076 |
|  | PREDICTED: uncharacterized protein LOC101736189 - [512917416_XP_004928881.1] | 1.30367061 | 0.7858809 |
|  | PREDICTED: talin-1-like - [328785261_XP_391944.4] | #N/A | 0.7855101 |
|  | PREDICTED: LOW QUALITY PROTEIN: putative transcription factor capicua-like - [512927783_XP_004931421.1] | #N/A | 0.7845198 |
|  | actin related protein 2/3 complex subunit 4 - [114052038_NP_001040449.1] | 1.07238732 | 0.7829255 |
|  | PREDICTED: replication factor C subunit 1-like - [512892886_XP_004923043.1] | 1.37245 | 0.7820836 |
|  | 40S ribosomal protein S3a - [157135190_XP_001656565.1] | #N/A | 0.781648 |
|  | PREDICTED: transcription initiation factor TFIID subunit 8-like - [512914943_XP_004928267.1] | 1.12038461 | 0.7801615 |
|  | PREDICTED: spectrin beta chain, non-erythrocytic 5-like - [512901464_XP_004925141.1] | 1.20329452 | 0.780109 |
|  | PREDICTED: DNA damage-binding protein 1-like - [512897190_XP_004924093.1] | 1.23046938 | 0.7800142 |
|  | PREDICTED: ankyrin-3-like - [345490474_XP_003426386.1] | 1.1775838 | 0.779787 |
|  | C. briggsae CBR-RPB-2 protein - [268574492_XP_002642223.1] | 1.1980449 | 0.7777027 |
|  | PREDICTED: spectrin beta chain, non-erythrocytic 5-like - [512901464_XP_004925141.1] | 1.18553316 | 0.7770135 |
|  | apoptosis-linked protein 2 - [114051846_NP_001040187.1] | 1.19170539 | 0.7759681 |
|  | snap-scaffold5054_size8063-abinit-gene-0.0-mRNA-1 | #N/A | 0.7749174 |
|  | PREDICTED: mothers against decapentaplegic homolog 4-like - [512927799_XP_004931425.1] | #N/A | 0.7725224 |
|  | PREDICTED: pleiotropic regulator 1 | 1.10250151 | 0.772319 |
|  | PREDICTED: trinucleotide repeat-containing gene 6B protein-like - [512888089_XP_004922030.1] | 1.1577235 | 0.7720446 |
|  | GA19530 - [198454358_XP_001359561.2] | 1.16886708 | 0.7708166 |
|  | PREDICTED: metallophosphoesterase 1-like - [156543679_XP_001605368.1] | 1.11448727 | 0.7702978 |
|  | PREDICTED: uncharacterized protein LOC101735359 - [512936592_XP_004933555.1] | 1.18249991 | 0.769845 |
|  | PREDICTED: peptidyl-prolyl cis-trans isomerase H-like - [512923824_XP_004930446.1] | 1.147867 | 0.7688879 |
|  | nuclear migration protein nudC - [114052643_NP_001040262.1] | 1.11788369 | 0.76794 |
|  | PREDICTED: vacuolar protein sorting associated protein 25 | 1.08933409 | 0.7675966 |
|  | PREDICTED: retinal dehydrogenase 1-like - [512926556_XP_004931115.1] | 1.04751012 | 0.7649028 |
|  | polychaetoid, isoform K - [161078104_NP_001097712.1] | 1.14812257 | 0.7645666 |
|  | PREDICTED: sodium/potassium-transporting ATPase subunit beta-2-like - [512918498_XP_004929149.1] | 1.1064164 | 0.7637433 |
|  | adenylate cyclase - [114052224_NP_001040230.1] | 1.10351688 | 0.7629868 |
|  | HSP90 cochaperone CDC37 homologue - [112983392_NP_001036991.1] | 1.16624189 | 0.7629075 |
|  | PREDICTED: cyclin-G-associated kinase-like - [512910428_XP_004927169.1] | 1.13206438 | 0.7623918 |
|  | PREDICTED: mitotic checkpoint protein BUB3-like - [512911375_XP_004927396.1] | 1.20430662 | 0.7608495 |
|  | PREDICTED: luciferin 4-monooxygenase-like - [512917289_XP_004928851.1] | #N/A | 0.7602605 |
|  | loquacious - [304307737_NP_001182008.1] | 1.10921694 | 0.7598164 |
|  | PREDICTED: E3 ubiquitin-protein ligase SIAH1 | 1.16222156 | 0.7593248 |
|  | protein phsophatase-2a - [157105387_XP_001648846.1] | 1.1948128 | 0.7590945 |
|  | PREDICTED: solute carrier family 39 (zinc transporter), member 13 | #N/A | 0.7580563 |
|  | PREDICTED: mediator of RNA polymerase II transcription subunit 15-like - [512919288_XP_004929338.1] | #N/A | 0.7573527 |
|  | alpha-esterase 45 - [162462783_NP_001104822.1] | #N/A | 0.7569939 |
|  | thymidylate synthase isoform 1 - [114053279_NP_001040281.1] | 1.15168873 | 0.7568262 |
|  | sorbitol dehydrogenase - [112983744_NP_001037311.1] | 1.24627769 | 0.7565076 |
|  | CRE-FAH-1 protein - [308511411_XP_003117888.1] | 1.01175622 | 0.7564863 |
|  | glutaminyl-tRNA synthetase - [170577575_XP_001894060.1] | 1.11335139 | 0.755839 |
|  | signal recognition particle 14 kDa protein-like protein - [148298791_NP_001091775.1] | 1.14371804 | 0.7556346 |
|  | snap-scaffold3674_size30976-abinit-gene-0.4-mRNA-1 | #N/A | 0.7550174 |
|  | PREDICTED: 26S protease regulatory subunit 7-like isoform X1 - [512896702_XP_004923974.1] | 1.22489776 | 0.7549374 |
|  | translation initiation factor 5A - [112982832_NP_001037538.1] | 1.08369034 | 0.7538109 |
|  | 5'-methylthioadenosine phosphorylase - [114052284_NP_001040514.1] | #N/A | 0.7518217 |
|  | PREDICTED: myosin-10-like - [512918179_XP_004929069.1] | #N/A | 0.7517689 |
|  | PREDICTED: uncharacterized protein LOC101737962 - [512924066_XP_004930507.1] | 1.22237096 | 0.7516443 |
|  | phosphatidylethanolamine binding protein isoform 2 - [153792114_NP_001093267.1] | 1.09281015 | 0.7512879 |
|  | ubiquitin conjugating enzyme E2 - [114052140_NP_001040223.1] | #N/A | 0.7504665 |
|  | PREDICTED: WD repeat and FYVE domain-containing protein 2-like isoform X1 - [512925650_XP_004930891.1] | #N/A | 0.750047 |
|  | snap-scaffold1668_size88367-abinit-gene-0.7-mRNA-1 | #N/A | 0.7494904 |
|  | translationally-controlled tumor protein homolog - [112982880_NP_001037572.1] | 1.12994356 | 0.7492131 |
|  | PREDICTED: HD domain-containing protein 2-like isoform X1 - [512922362_XP_004930102.1] | 1.09749973 | 0.7482087 |
|  | thioredoxin family Trp26 - [114052156_NP_001040222.1] | 1.15604265 | 0.7481814 |
|  | snap-scaffold1574_size45437-abinit-gene-0.6-mRNA-1 | #N/A | 0.7478699 |
|  | PREDICTED: MOB kinase activator-like 1-like - [512908089_XP_004926597.1] | #N/A | 0.7467506 |
|  | glutathione S-transferase epsilon 3 - [169234690_NP_001108466.1] | #N/A | 0.7458494 |
|  | PREDICTED: NEDD8-activating enzyme E1 catalytic subunit-like - [328779827_XP_623205.2] | 1.20298606 | 0.745627 |
|  | hydroxypyruvate isomerase - [114052328_NP_001040471.1] | 1.12211267 | 0.7455573 |
|  | DNA-directed RNA polymerase II polypeptide - [114051193_NP_001040388.1] | 1.13667849 | 0.7454555 |
|  | PREDICTED: hsp70-binding protein 1-like - [512917809_XP_004928978.1] | 1.22855446 | 0.745186 |
|  | PREDICTED: bifunctional glutamate/proline--tRNA ligase-like - [391341315_XP_003744976.1] | 1.16268245 | 0.7447106 |
|  | DNA-damage inducible protein - [114051417_NP_001040314.1] | 1.23834495 | 0.7446083 |
|  | superoxide dismutase [Cu-Zn] - [112982998_NP_001037084.1] | 1.10793687 | 0.7433697 |
|  | PREDICTED: uncharacterized protein LOC101736189 - [512917416_XP_004928881.1] | 1.15749632 | 0.7429566 |
|  | PREDICTED: microtubule-associated protein futsch-like - [512914323_XP_004928113.1] |  | 0.7429536 |
|  | PREDICTED: GTP-binding protein SAR1b-like isoform X1 - [512926764_XP_004931167.1] | 1.19079139 | 0.7422457 |
|  | DNA excision repair protein ERCC-3 | 1.35360662 | 0.7422112 |
|  | PREDICTED: CDK-activating kinase assembly factor MAT1-like - [512894527_XP_004923452.1] | #N/A | 0.7421752 |
|  | PREDICTED: DNA ligase 3 - [383847368_XP_003699326.1] | #N/A | 0.7420278 |
|  | PREDICTED: conserved oligomeric Golgi complex subunit 6-like - [512926431_XP_004931084.1] | 1.09736387 | 0.7418973 |
|  | S-phase kinase-associated protein - [114052370_NP_001040518.1] | 1.1530407 | 0.7416755 |
|  | cAMP-dependent protein kinase R2 - [162462811_NP_001104823.1] | #N/A | 0.7401868 |
|  | predicted protein - [156376445_XP_001630371.1] | 1.08604532 | 0.7400952 |
|  | PREDICTED: trans-Golgi network integral membrane protein 2-like - [512924524_XP_004930616.1] | #N/A | 0.7388449 |
|  | PREDICTED: LOW QUALITY PROTEIN: WD repeat-containing protein 7-like - [512935826_XP_004933369.1] | #N/A | 0.7382408 |
|  | DnaJ (Hsp40) homolog 9 - [114051830_NP_001040185.1] | 1.3340573 | 0.737669 |
|  | GE17320 - [195479496_XP_002100908.1] | 1.21134131 | 0.7374388 |
|  | PREDICTED: uncharacterized protein LOC101737617 - [512891577_XP_004922719.1] | #N/A | 0.7362569 |
|  | ubiquitin-conjugating enzyme E2L - [112983864_NP_001037390.1] | 1.14427711 | 0.7359859 |
|  | PREDICTED: DNA primase large subunit-like - [512918775_XP_004929214.1] | 1.28900411 | 0.7347843 |
|  | PREDICTED: exocyst complex component 7-like - [512907245_XP_004926394.1] | 1.12994573 | 0.7343089 |
|  | PREDICTED: uncharacterized protein LOC101738789 - [512896542_XP_004923935.1] | #N/A | 0.7343016 |
|  | ubiquitin-conjugating enzyme E2 - [114050835_NP_001040407.1] | 1.09072463 | 0.7339119 |
|  | PREDICTED: uncharacterized protein LOC101741417 - [512889331_XP_004922250.1] | 1.16878437 | 0.7335272 |
|  | proteasome zeta subunit - [114050993_NP_001040146.1] | 1.17427089 | 0.7328114 |
|  | PREDICTED: uncharacterized protein LOC101747094 - [512935811_XP_004933365.1] | #N/A | 0.7319192 |
|  | cellular retinoic acid binding protein - [112983600_NP_001037364.1] | 1.12602668 | 0.7318385 |
|  | AGAP000320-PB - [347963597_XP_003436968.1] | 1.13089494 | 0.7313532 |
|  | PREDICTED: bifunctional aminoacyl-tRNA synthetase-like, partial - [345492707_XP_001599828.2] | 1.15910136 | 0.7312639 |
|  | DnaJ (Hsp40) homolog 10 - [255652891_NP_001157386.1] | 1.19507994 | 0.7312593 |
|  | PREDICTED: thioredoxin-2 isoform 1 - [328784195_XP_003250408.1] | 1.13691963 | 0.7309932 |
|  | PREDICTED: 85/88 kDa calcium-independent phospholipase A2-like - [512928483_XP_004931589.1] | 1.15202049 | 0.7309739 |
|  | PREDICTED: leucyl-tRNA synthetase, cytoplasmic-like - [328714927_XP_001944432.2] | 1.17682639 | 0.7305713 |
|  | PREDICTED: acylphosphatase-2-like isoform X1 - [512933137_XP_004932716.1] | 1.1808879 | 0.7305442 |
|  | serine protease inhibitor 5 precursor - [112984548_NP_001037205.1] | #N/A | 0.7294232 |
|  | PREDICTED: spermine oxidase-like - [512920843_XP_004929734.1] | #N/A | 0.7293567 |
|  | mitochondrial ATP synthase coupling factor 6 - [148298673_NP_001091814.1] | 1.0665689 | 0.7290402 |
|  | caspase-1 - [112983104_NP_001037050.1] | 1.11960036 | 0.7288162 |
|  | AGAP010957-PA, partial - [158287848_XP_309749.4] | 1.10463847 | 0.7285661 |
|  | PREDICTED: gelsolin-like - [512889118_XP_004922200.1] | 1.10631397 | 0.7274907 |
|  | annexin isoform 2 - [148298814_NP_001091759.1] | 1.18836403 | 0.7269009 |
|  | PREDICTED: s-adenosylmethionine synthase-like isoform 2 - [340729976_XP_003403268.1] | #N/A | 0.7266688 |
|  | PREDICTED: nicalin-like - [512936212_XP_004933463.1] | 1.10758876 | 0.7261714 |
|  | PREDICTED: sorting and assembly machinery component 50 homolog A-like - [512925090_XP_004930753.1] | 1.13816037 | 0.7261426 |
|  | proteasome beta 3 subunit - [114052162_NP_001040460.1] | 1.12809383 | 0.7247316 |
|  | GH15895 - [195012013_XP_001983431.1] | #N/A | 0.7245082 |
|  | GA26189 - [198455115_XP_002138008.1] | 1.19527966 | 0.7243614 |
|  | eukaryotic peptide chain release factor subunit 1 - [339240611_XP_003376231.1] | 1.17303836 | 0.7242511 |
|  | PREDICTED: 4,5-DOPA dioxygenase extradiol-like protein-like - [512907709_XP_004926510.1] | 1.17000006 | 0.7236321 |
|  | DNA polymerase delta catalytic subunit - [256087234_XP_002579779.1] | 1.15699792 | 0.7234126 |
|  | nuclear migration protein nudC - [114052643_NP_001040262.1] | 1.10331579 | 0.7226774 |
|  | high mobility group protein A-like - [240848689_NP_001155402.1] | 1.28354539 | 0.7221528 |
|  | PREDICTED: S-formylglutathione hydrolase-like isoform X1 - [512915980_XP_004928520.1] | 1.07290917 | 0.7219893 |
|  | eukaryotic translation initiation factor 4H - [112983124_NP_001037660.1] | 1.12589491 | 0.7211668 |
|  | PREDICTED: cytochrome P450 6B1-like - [512892967_XP_004923064.1] | #N/A | 0.720953 |
|  | PREDICTED: similar to zinc finger protein - [189239068_XP_971434.2] | 1.20933022 | 0.7207766 |
|  | GF14731 - [194759206_XP_001961840.1] | 1.06224165 | 0.7202565 |
|  | PREDICTED: similar to chromodomain helicase-DNA-binding protein 3 - [189240851_XP_001812556.1] | #N/A | 0.7201869 |
|  | PREDICTED: uncharacterized protein LOC101746228 - [512889447_XP_004922280.1] | 1.15369429 | 0.7200149 |
|  | PREDICTED: probable phosphorylase b kinase regulatory subunit beta-like - [512926363_XP_004931067.1] | #N/A | 0.7192171 |
|  | AGAP009944-PA - [158298940_XP_319075.3] | 1.07318149 | 0.7185344 |
|  | PREDICTED: myotrophin-like - [512929361_XP_004931801.1] | 1.1125571 | 0.7185332 |
|  | PREDICTED: ribonuclease H2 subunit B-like - [512899279_XP_004924604.1] | 1.18198015 | 0.718215 |
|  | atypical protein kinase C - [112982924_NP_001036923.1] | 1.23360879 | 0.717916 |
|  | PREDICTED: leucine--tRNA ligase, cytoplasmic-like - [512912899_XP_004927767.1] | 1.18772856 | 0.71702 |
|  | PREDICTED: solute carrier family 12 member 8-like isoform X1 - [499008770_XP_004536402.1] | #N/A | 0.7168499 |
|  | PREDICTED: hypothetical protein LOC100574695 - [328698037_XP_003240520.1] | #N/A | 0.7167423 |
|  | PREDICTED: tumor protein D54-like isoform X3 - [512922426_XP_004930117.1] | 1.22461627 | 0.7166157 |
|  | PREDICTED: probable UDP-glucose 4-epimerase-like isoform 1 - [156539983_XP_001602357.1] | 1.1421893 | 0.7162696 |
|  | PREDICTED: protein KBP homolog - [512919683_XP_004929445.1] | 1.24289454 | 0.7159591 |
|  | PREDICTED: adenylate kinase isoenzyme 6 homolog - [512922044_XP_004930023.1] | 0.85121531 | 0.7155377 |
|  | PREDICTED: similar to dolichyl-phosphate mannosyltransferase - [91086609_XP_973998.1] | 1.06556751 | 0.7155377 |
|  | PREDICTED: titin-like - [512888968_XP_004922174.1] | 1.22517548 | 0.7153314 |
|  | PREDICTED: glycerol kinase-like isoform X1 - [498959495_XP_004524486.1] | 1.15313047 | 0.715153 |
|  | PREDICTED: DNA polymerase delta subunit 2-like isoform X1 - [512927887_XP_004931447.1] | #N/A | 0.7148803 |
|  | histone 1 - [170058994_XP_001865168.1] | 1.22031084 | 0.7144014 |
|  | PREDICTED: cysteine and histidine-rich domain-containing protein-like - [512919933_XP_004929505.1] | 1.25678732 | 0.7143981 |
|  | pyruvate dehydrogenase - [153792309_NP_001093304.1] | 1.15686845 | 0.7137522 |
|  | PREDICTED: BRCA1-associated RING domain protein 1-like - [383860223_XP_003705590.1] | #N/A | 0.7137052 |
|  | PREDICTED: arginine-glutamic acid dipeptide repeats protein-like isoform X1 - [512920505_XP_004929649.1] | 1.20961259 | 0.7132888 |
|  | PREDICTED: NADPH-dependent diflavin oxidoreductase 1-like - [512934404_XP_004933024.1] | #N/A | 0.7129872 |
|  | PREDICTED: probable aminoacyl tRNA synthase complex-interacting multifunctional protein 2-like isoform X4 - [512900292_XP_004924856.1] | 1.11782805 | 0.7124189 |
|  | PREDICTED: armadillo repeat-containing protein 8-like - [512928824_XP_004931674.1] | 1.11043399 | 0.7118846 |
|  | PREDICTED: TPPP family protein CG4893-like isoform X1 - [512928131_XP_004931507.1] | 1.11793977 | 0.7114132 |
|  | PREDICTED: polypeptide N-acetylgalactosaminyltransferase 5-like - [512929100_XP_004931741.1] | #N/A | 0.7107068 |
|  | eukaryotic translation initiation factor 2 subunit 2 - [112982928_NP_001037678.1] | 1.17061752 | 0.7104938 |
|  | PREDICTED: proteasome subunit beta type-6-like - [512892807_XP_004923023.1] | 1.1433058 | 0.7101189 |
|  | PREDICTED: LIN1-like protein-like - [512923254_XP_004930309.1] | 1.19891903 | 0.7100478 |
|  | PREDICTED: phosphomannomutase 1-like - [512887983_XP_004922013.1] | 1.13236912 | 0.7099746 |
|  | PREDICTED: armadillo repeat-containing protein 6 homolog - [512894597_XP_004923468.1] | 1.1598482 | 0.7092704 |
|  | GJ16193 - [195401074_XP_002059139.1] | 1.17698014 | 0.7091967 |
|  | clip domain serine protease 11 precursor - [112983100_NP_001037053.1] | 1.22684657 | 0.7090494 |
|  | GK17499 - [195428605_XP_002062362.1] | 1.09863374 | 0.7090417 |
|  | PREDICTED: geranylgeranyl pyrophosphate synthase-like isoform X1 - [512926822_XP_004931181.1] | 1.16400099 | 0.708994 |
|  | PREDICTED: BRCA1-A complex subunit BRE-like isoform X1 - [512907463_XP_004926449.1] | 1.11273185 | 0.708828 |
|  | PREDICTED: uncharacterized protein LOC101737289 - [512896823_XP_004924003.1] | #N/A | 0.7088216 |
|  | PREDICTED: isoleucyl-tRNA synthetase, cytoplasmic-like - [156549425_XP_001602982.1] | 1.22245107 | 0.708411 |
|  | snap-scaffold229_size256595-abinit-gene-0.11-mRNA-1 | #N/A | 0.7082965 |
|  | UDP-glucosyltransferase precursor - [306518648_NP_001182386.1] | 1.19387267 | 0.7082101 |
|  | PREDICTED: myotubularin-related protein 3-like - [512891891_XP_004922795.1] | 0.9371248 | 0.7073328 |
|  | atypical protein kinase C - [112982924_NP_001036923.1] | #N/A | 0.7069521 |
|  | PREDICTED: charged multivesicular body protein 4b-like - [512894601_XP_004923469.1] | 1.14289055 | 0.7069483 |
|  | PREDICTED: prefoldin subunit 5-like - [383851510_XP_003701275.1] | 1.16881649 | 0.7067724 |
|  | PREDICTED: peptidyl-prolyl cis-trans isomerase-like isoform 2 - [156536889_XP_001607048.1] | 1.11940915 | 0.7066807 |
|  | PREDICTED: proteasome-associated protein ECM29 homolog - [512910450_XP_004927174.1] | #N/A | 0.7066152 |
|  | PREDICTED: similar to predicted protein - [91079660_XP_966451.1] | 1.17425327 | 0.706574 |
|  | PREDICTED: Golgi reassembly-stacking protein 2-like - [512900244_XP_004924844.1] | 1.16522401 | 0.7065098 |
|  | acidic nucleoplasmic DNA-binding protein 1 - [217330566_NP_001136085.1] | #N/A | 0.7060064 |
|  | PREDICTED: hexokinase type 2-like isoform X2 - [512932872_XP_004932651.1] | #N/A | 0.7056551 |
|  | 6-phosphofructo-2-kinase/fructose-2,6-bisphosphatase short form - [157134604_XP_001663325.1] | 1.04800361 | 0.7053466 |
|  | PREDICTED: lambda-crystallin homolog isoform X1 - [512929917_XP_004931941.1] | 1.08821179 | 0.7052105 |
|  | ubiquitin conjugating enzyme E2 - [114051115_NP_001040393.1] | 1.04023218 | 0.7050145 |
|  | PREDICTED: LOW QUALITY PROTEIN: liver carboxylesterase-like - [512902952_XP_004925500.1] | 1.03868916 | 0.7048819 |
|  | PREDICTED: HIRA-interacting protein 3-like - [512891851_XP_004922785.1] | 1.14923135 | 0.7045839 |
|  | PREDICTED: tudor domain-containing protein 7-like - [512923292_XP_004930319.1] | #N/A | 0.7038448 |
|  | N-acetyltransferase - [114051023_NP_001040325.1] | 1.17876379 | 0.7037732 |
|  | PREDICTED: ethanolaminephosphotransferase 1-like - [512890487_XP_004922523.1] | #N/A | 0.7037407 |
|  | PREDICTED: ER membrane protein complex subunit 2-like - [512901995_XP_004925266.1] | 1.21851234 | 0.7035326 |
|  | PREDICTED: 28 kDa heat- and acid-stable phosphoprotein-like - [512909959_XP_004927049.1] | 1.29265428 | 0.7032919 |
|  | PREDICTED: dynein heavy chain, cytoplasmic-like - [391335980_XP_003742362.1] | 1.23479334 | 0.7029577 |
|  | PREDICTED: RWD domain-containing protein 2A-like - [512913329_XP_004927870.1] | 1.17379557 | 0.7027275 |
|  | PREDICTED: dymeclin-like - [512935562_XP_004933304.1] | 1.11905095 | 0.7019671 |
|  | GA15320 - [198471247_XP_001355553.2] | 1.21917888 | 0.7008904 |
|  | snap-scaffold1700_size205459-processed-gene-0.1-mRNA-1 | #N/A | 0.7005699 |
|  | PREDICTED: LOW QUALITY PROTEIN: esterase B1-like - [512907828_XP_004926539.1] | #N/A | 0.7000722 |
|  | PREDICTED: LOW QUALITY PROTEIN: phosphopantothenate--cysteine ligase-like - [512902122_XP_004925297.1] | 1.14481718 | 0.6999071 |
|  | PREDICTED: fatty-acid amide hydrolase 2-like isoform X2 - [512896660_XP_004923964.1] | 1.1254503 | 0.6996621 |
|  | replication protein A2 - [112983110_NP_001036939.1] | #N/A | 0.6995468 |
|  | PREDICTED: B-cell receptor-associated protein 31-like isoform X1 - [512895314_XP_004923642.1] | 1.25919667 | 0.6994898 |
|  | AGAP002521-PA - [347968005_XP_312416.4] | 1.11362548 | 0.6991224 |
|  | PREDICTED: putative glycogen [starch] synthase-like - [512925031_XP_004930739.1] | 1.35790278 | 0.6988706 |
|  | PREDICTED: uncharacterized protein LOC101735373 - [512902459_XP_004925381.1] | #N/A | 0.6987816 |
|  | PREDICTED: peroxisomal acyl-coenzyme A oxidase 3-like - [512924002_XP_004930491.1] | 1.16751491 | 0.6987765 |
|  | PREDICTED: V-type proton ATPase subunit H isoform 2 - [345485000_XP_003425170.1] | 1.17679989 | 0.6984689 |
|  | PREDICTED: dynactin subunit 1-like - [512907697_XP_004926507.1] | 1.24502407 | 0.6980116 |
|  | C. briggsae CBR-DHS-15 protein, partial - [268565163_XP_002647282.1] | 1.1066254 | 0.6977377 |
|  | PREDICTED: signal-induced proliferation-associated 1-like protein 2-like isoform X1 - [524874009_XP_005093746.1] | 1.11082037 | 0.6973748 |
|  | snap-scaffold120_size213325-abinit-gene-1.5-mRNA-1 | #N/A | 0.6968375 |
|  | PREDICTED: N-acetylgalactosaminyltransferase 7-like isoform X1 - [524898638_XP_005105765.1] | 1.1581474 | 0.6967002 |
|  | elongation factor 1 delta - [112983886_NP_001036853.1] | 1.19446093 | 0.6963623 |
|  | snap-scaffold331_size168604-abinit-gene-0.28-mRNA-1 | #N/A | 0.6962972 |
|  | PREDICTED: reticulocalbin-2-like - [512939713_XP_004934308.1] | #N/A | 0.6961607 |
|  | PREDICTED: IST1 homolog - [512930120_XP_004931988.1] | 1.1851361 | 0.6961414 |
|  | roadblock domain-containing protein 3 - [114050829_NP_001040324.1] | #N/A | 0.6955351 |
|  | PREDICTED: cysteine sulfinic acid decarboxylase-like - [512933911_XP_004932908.1] | #N/A | 0.6954262 |
|  | arginyl aa-tRNA synthetase protein 1, isoform b - [170584080_XP_001896849.1] | 1.15948372 | 0.6951323 |
|  | PREDICTED: similar to AGAP006340-PC - [189237679_XP_968527.2] | #N/A | 0.6947295 |
|  | PREDICTED: uncharacterized protein LOC101737115 - [512917794_XP_004928975.1] | #N/A | 0.6944086 |
|  | PREDICTED: uncharacterized protein CG4449-like - [512935641_XP_004933323.1] | 1.14188121 | 0.6942911 |
|  | PREDICTED: rho GTPase-activating protein 190 - [328792264_XP_624122.3] | 1.20339031 | 0.6936913 |
|  | PREDICTED: uncharacterized protein LOC101737920 - [512921994_XP_004930011.1] | 0.96486834 | 0.6935671 |
|  | PREDICTED: phosphoglycolate phosphatase-like - [512928127_XP_004931506.1] | 1.19813378 | 0.6933934 |
|  | PREDICTED: methylosome protein 50-like - [512900383_XP_004924878.1] | 1.07290952 | 0.69326 |
|  | coiled-coil domain containing 25 protein - [114050747_NP_001040106.1] | 0.88068311 | 0.6928351 |
|  | snap-scaffold4002_size63275-abinit-gene-0.9-mRNA-1 | #N/A | 0.6925458 |
|  | PREDICTED: flavin reductase (NADPH)-like isoform X1 - [512903927_XP_004925737.1] | 1.15948657 | 0.6916166 |
|  | PREDICTED: gamma-glutamylcyclotransferase-like - [512922278_XP_004930081.1] | 1.15606135 | 0.6914614 |
|  | RNA helicase-like protein - [112983270_NP_001037005.1] | #N/A | 0.6903849 |
|  | PREDICTED: putative ATP-dependent RNA helicase me31b-like - [512903199_XP_004925561.1] | 1.24187947 | 0.6902979 |
|  | PREDICTED: inositol 1,4,5-trisphosphate receptor-like - [512895244_XP_004923625.1] | #N/A | 0.6902375 |
|  | ribonucleoside-diphosphate reductase large subunit - [170038940_XP_001847305.1] | 1.19095352 | 0.690209 |
|  | PREDICTED: liprin-beta-1-like - [512913009_XP_004927795.1] | #N/A | 0.689726 |
|  | PREDICTED: palmitoyltransferase ZDHHC3-like isoform X1 - [512921291_XP_004929841.1] | #N/A | 0.6895273 |
|  | PREDICTED: uncharacterized protein LOC101743001, partial - [512935195_XP_004933215.1] | 1.27203401 | 0.6891317 |
|  | PREDICTED: activating signal cointegrator 1-like - [512890142_XP_004922465.1] | 1.19508397 | 0.6887326 |
|  | PREDICTED: rho GTPase-activating protein 8-like - [512933849_XP_004932893.1] | #N/A | 0.6887175 |
|  | PREDICTED: similar to myosin VA (heavy polypeptide 12, myoxin) - [91087053_XP_974649.1] | 1.14674239 | 0.6884979 |
|  | ATP binding protein - [114051584_NP_001040309.1] | #N/A | 0.6884074 |
|  | signal peptidase 18 kDa subunit - [114052797_NP_001040280.1] | 1.27569298 | 0.6883491 |
|  | PREDICTED: glutamate--cysteine ligase regulatory subunit-like - [512890896_XP_004922592.1] | #N/A | 0.6881895 |
|  | PREDICTED: phosphoserine phosphatase-like isoform X1 - [512911229_XP_004927361.1] | 1.2815471 | 0.6877041 |
|  | Hsc70/Hsp90-organizing protein HOP - [112983280_NP_001036957.1] | 1.15535547 | 0.6876209 |
|  | maker-scaffold7114_size20040-snap-gene-0.4-mRNA-1 | 1.18559689 | 0.6873364 |
|  | AGAP009944-PA - [158298940_XP_319075.3] | 1.10504974 | 0.6872066 |
|  | PREDICTED: WW domain-binding protein 2-like - [512934101_XP_004932952.1] | 1.28353491 | 0.6870799 |
|  | PREDICTED: N-terminal kinase-like protein-like - [512887068_XP_004921861.1] | #N/A | 0.6869893 |
|  | PREDICTED: LOW QUALITY PROTEIN: protein furry-like - [380018403_XP_003693118.1] | #N/A | 0.6865708 |
|  | PREDICTED: protein FAM86A-like - [512923775_XP_004930434.1] | #N/A | 0.686204 |
|  | PREDICTED: DCN1-like protein 4-like isoform X1 - [512902826_XP_004925470.1] | #N/A | 0.6860431 |
|  | ubiquinol-cytochrome C reductase complex 14kD subunit - [113866028_NP_001038957.1] | 1.14527921 | 0.6858903 |
|  | PREDICTED: V-type proton ATPase 116 kDa subunit a isoform 1-like - [499000245_XP_004534298.1] | #N/A | 0.6856535 |
|  | PREDICTED: actin-interacting protein 1-like isoform 2 - [340715670_XP_003396332.1] | 1.16102423 | 0.6845995 |
|  | PREDICTED: ras suppressor protein 1-like isoform X1 - [512892297_XP_004922897.1] | 1.17899417 | 0.6845488 |
|  | PREDICTED: uncharacterized protein LOC101738618 - [512918149_XP_004929061.1] | 1.1335056 | 0.684251 |
|  | PREDICTED: receptor-type tyrosine-protein phosphatase delta-like - [340380969_XP_003388994.1] | 1.08273815 | 0.6839557 |
|  | PREDICTED: similar to protease, reverse transcriptase, ribonuclease H, integrase - [189234033_XP_001807972.1] | 1.08176707 | 0.6833538 |
|  | PREDICTED: rab proteins geranylgeranyltransferase component A 2-like - [512912915_XP_004927771.1] | #N/A | 0.6831153 |
|  | PREDICTED: ER membrane protein complex subunit 8/9 homolog - [512928594_XP_004931616.1] | 1.18865367 | 0.682707 |
|  | PREDICTED: 8-oxo-dGDP phosphatase NUDT18-like - [512932745_XP_004932622.1] | #N/A | 0.6824858 |
|  | PREDICTED: lysine--tRNA ligase-like isoform X1 - [512926020_XP_004930980.1] | 1.11948042 | 0.6822709 |
|  | maker-scaffold572_size97023-snap-gene-0.19-mRNA-1 | 1.13704283 | 0.6822481 |
|  | PREDICTED: uncharacterized protein LOC101737964 - [512927607_XP_004931377.1] | #N/A | 0.6821549 |
|  | PREDICTED: band 4.1-like protein 5-like isoform X2 - [498983113_XP_004530031.1] | #N/A | 0.682055 |
|  | PREDICTED: triosephosphate isomerase-like - [340376355_XP_003386698.1] | 1.15211631 | 0.6814235 |
|  | PREDICTED: phosphoglucomutase | 1.16655448 | 0.6811085 |
|  | PREDICTED: mitochondrial import receptor subunit TOM20 homolog - [512893172_XP_004923115.1] | 1.16312237 | 0.6809856 |
|  | PREDICTED: ubiquitin conjugation factor E4 A-like - [512931356_XP_004932282.1] | 1.25581615 | 0.6809701 |
|  | NADP-dependent oxidoreductase - [148298833_NP_001091765.1] | #N/A | 0.6807149 |
|  | PREDICTED: ADP-ribosylation factor-like protein 1-like - [512912779_XP_004927739.1] | 1.16401316 | 0.6806625 |
|  | snap-scaffold4476_size9160-abinit-gene-0.2-mRNA-1 | #N/A | 0.6804472 |
|  | mitochondrial intermembrane space translocase subunit Tim10 - [160333493_NP_001037412.2] | 1.121467 | 0.6803005 |
|  | PREDICTED: telomerase-binding protein EST1A-like - [512900419_XP_004924887.1] | 1.21643665 | 0.6802322 |
|  | PREDICTED: prostaglandin reductase 1-like - [512931094_XP_004932223.1] | 1.1542025 | 0.680094 |
|  | PREDICTED: cell division cycle and apoptosis regulator protein 1-like - [383863264_XP_003707101.1] | 1.16019091 | 0.6800814 |
|  | thymosin isoform 1 - [114052645_NP_001040486.1] | 1.03003767 | 0.680032 |
|  | PREDICTED: uncharacterized protein LOC101738528 - [512903997_XP_004925754.1] | #N/A | 0.6799253 |
|  | snap-scaffold288_size219777-abinit-gene-0.5-mRNA-1 | #N/A | 0.6797206 |
|  | PREDICTED: uncharacterized protein LOC101459168 - [498972498_XP_004527327.1] | 1.06840629 | 0.6797189 |
|  | PREDICTED: acyl-CoA synthetase family member 3, mitochondrial-like - [512910844_XP_004927270.1] | 1.09386465 | 0.6794659 |
|  | PREDICTED: LOW QUALITY PROTEIN: nipped-B-like protein A-like - [512900427_XP_004924889.1] | #N/A | 0.6794145 |
|  | AGAP004852-PB - [158293046_XP_001688561.1] | 1.17763665 | 0.6793943 |
|  | AGAP001606-PA - [118794464_XP_321495.3] | 1.15435608 | 0.6793316 |
|  | snap-scaffold423_size138491-abinit-gene-0.9-mRNA-1 | #N/A | 0.6791732 |
|  | PREDICTED: uncharacterized protein LOC101739352 - [512895193_XP_004923612.1] | 1.06339267 | 0.679109 |
|  | PREDICTED: E3 ubiquitin-protein ligase RNF181-like - [512924430_XP_004930593.1] | #N/A | 0.6782842 |
|  | PREDICTED: lysosomal Pro-X carboxypeptidase-like isoform X1 - [512906728_XP_004926267.1] | 1.12108228 | 0.6781649 |
|  | UDP-glucosyltransferase precursor - [306518648_NP_001182386.1] | 1.16714975 | 0.6781474 |
|  | alpha-esterase 19 isoform 1 precursor - [174840656_NP_001116501.1] | #N/A | 0.6779795 |
|  | PREDICTED: uncharacterized protein LOC101737762 isoform X3 - [512892930_XP_004923054.1] | #N/A | 0.6777459 |
|  | PREDICTED: hypothetical protein LOC100743558 - [350416090_XP_003490837.1] | 1.12219954 | 0.6776943 |
|  | hypothetical protein TRIADDRAFT_24562 - [196003934_XP_002111834.1] | 1.06278185 | 0.6771984 |
|  | PREDICTED: uncharacterized protein LOC101737962 - [512924066_XP_004930507.1] | 1.2300816 | 0.6770672 |
|  | AGAP010935-PA - [158287863_XP_309759.4] | #N/A | 0.6769805 |
|  | PREDICTED: conserved oligomeric Golgi complex subunit 8-like - [512930691_XP_004932122.1] | #N/A | 0.6768158 |
|  | PREDICTED: maternal protein tudor-like - [512928832_XP_004931676.1] | 1.13169247 | 0.6767762 |
|  | nucleotide excision repair protein - [298160921_NP_001177140.1] | 1.25945382 | 0.6765997 |
|  | PREDICTED: 15-hydroxyprostaglandin dehydrogenase [NAD(+)]-like - [512885775_XP_004921652.1] | 1.10061974 | 0.6765013 |
|  | GJ17643 - [195387860_XP_002052610.1] | #N/A | 0.6764704 |
|  | PREDICTED: alpha-mannosidase 2-like - [449665416_XP_002161447.2] | #N/A | 0.6759769 |
|  | adaptin - [114052214_NP_001040229.1] | 1.24472755 | 0.6759444 |
|  | PREDICTED: similar to CG1440 CG1440-PC - [189238112_XP_001814047.1] | #N/A | 0.6757955 |
|  | PREDICTED: similar to eukaryotic translation initiation factor 4 gamma, 2 - [189236794_XP_969772.2] | 1.21620183 | 0.6757534 |
|  | PREDICTED: LOW QUALITY PROTEIN: dynein heavy chain 8, axonemal-like - [512923223_XP_004930301.1] | #N/A | 0.6756205 |
|  | PREDICTED: uncharacterized protein LOC100898095, partial - [391325678_XP_003737356.1] | #N/A | 0.6749246 |
|  | PREDICTED: tryptophan--tRNA ligase, cytoplasmic-like - [512886734_XP_004921808.1] | 1.16888009 | 0.6747513 |
|  | PREDICTED: peroxisomal biogenesis factor 19-like - [512905612_XP_004925988.1] | #N/A | 0.6745655 |
|  | PREDICTED: RNA polymerase I-specific transcription initiation factor RRN3-like isoform X1 - [512923547_XP_004930379.1] | 1.16570692 | 0.6745456 |
|  | PREDICTED: protein kinase C iota type isoform 1 - [328777457_XP_397273.4] | 1.07154876 | 0.6741888 |
|  | cleavage and polyadenylation specific factor 5 - [114051528_NP_001040354.1] | 1.07366881 | 0.6737715 |
|  | PREDICTED: pantothenate kinase 1-like - [512911588_XP_004927450.1] | #N/A | 0.6737283 |
|  | PREDICTED: similar to seryl-tRNA synthetase - [91086675_XP_968460.1] | 1.16792383 | 0.6734432 |
|  | endothelial-monocyte activating polypeptide II - [114053109_NP_001040121.1] | 1.12358478 | 0.673042 |
|  | GK22419 - [195449892_XP_002072272.1] | 1.14914933 | 0.6729529 |
|  | PREDICTED: protein phosphatase methylesterase 1-like - [512910238_XP_004927119.1] | 1.16336711 | 0.6724868 |
|  | AGAP002344-PA - [347967663_XP_312621.4] | #N/A | 0.672471 |
|  | PREDICTED: uncharacterized protein LOC100903675 - [391326025_XP_003737526.1] | #N/A | 0.6724549 |
|  | PREDICTED: conserved oligomeric Golgi complex subunit 5-like - [512901116_XP_004925056.1] | #N/A | 0.6721147 |
|  | PREDICTED: glutamyl aminopeptidase-like - [380020925_XP_003694326.1] | #N/A | 0.6720843 |
|  | Hypothetical protein CBG01975 - [268577581_XP_002643773.1] | 1.01889776 | 0.6717512 |
|  | PREDICTED: peflin-like isoform X4 - [512893561_XP_004923212.1] | 1.13184081 | 0.6717309 |
|  | snap-scaffold6169_size6379-abinit-gene-0.1-mRNA-1 | #N/A | 0.6715177 |
|  | PREDICTED: LOW QUALITY PROTEIN: liprin-alpha-1-like - [512925321_XP_004930810.1] | #N/A | 0.6714254 |
|  | PREDICTED: bleomycin hydrolase-like isoform 2 - [345490081_XP_003426293.1] | 1.21726087 | 0.6712977 |
|  | PREDICTED: probable protein phosphatase CG10417-like - [512923848_XP_004930452.1] | 1.16417201 | 0.6709443 |
|  | vacuolar ATP synthase subunit G - [114053231_NP_001040287.1] | 1.18414429 | 0.670744 |
|  | PREDICTED: inositol monophosphatase 1-like - [512887951_XP_004922007.1] | 1.08819545 | 0.6703493 |
|  | snap-scaffold2648_size84043-processed-gene-0.12-mRNA-1 | 1.07527798 | 0.6702778 |
|  | elongation factor 1-beta' - [112982743_NP_001037556.1] | 1.11821919 | 0.6702636 |
|  | vacuolar ATP synthase subunit B, brain isoform - [339245355_XP_003378603.1] | 1.16253686 | 0.6699091 |
|  | GA21003 - [125808950_XP_001360932.1] | 1.19585129 | 0.6696074 |
|  | PREDICTED: histone acetyltransferase type B catalytic subunit-like - [512927408_XP_004931327.1] | 1.14086053 | 0.6695043 |
|  | PREDICTED: arrestin domain-containing protein 4-like - [512902720_XP_004925443.1] | 1.10836997 | 0.6691427 |
|  | hypothetical protein AaeL_AAEL002349 - [157128258_XP_001661369.1] | 1.2136783 | 0.6691309 |
|  | snap-scaffold4664_size29437-abinit-gene-0.0-mRNA-1 | #N/A | 0.6691064 |
|  | PREDICTED: AP-1 complex subunit gamma-1 - [383850090_XP_003700650.1] | 1.20604419 | 0.6688461 |
|  | Arp2/3 complex subunit - [115292423_NP_001041679.1] | 1.23184194 | 0.6688434 |
|  | PREDICTED: N(6)-adenine-specific DNA methyltransferase 2-like isoform X1 - [512903901_XP_004925730.1] | 1.24423455 | 0.6687631 |
|  | PREDICTED: bolA-like protein DDB_G0274169-like isoform X2 - [512892261_XP_004922888.1] | 0.97442353 | 0.6684727 |
|  | pyridoxine 5'-phosphate oxidase - [112984194_NP_001037442.1] | 1.14470214 | 0.6684476 |
|  | PREDICTED: lanC-like protein 3-like isoform X1 - [512910721_XP_004927240.1] | 1.1746613 | 0.6683184 |
|  | PREDICTED: inositol 1,4,5-trisphosphate receptor-like - [512895244_XP_004923625.1] | 1.39722493 | 0.6683147 |
|  | PREDICTED: SWI/SNF-related matrix-associated actin-dependent regulator of chromatin subfamily A containing DEAD/H box 1 homolog - [512895326_XP_004923645.1] | #N/A | 0.6683043 |
|  | PREDICTED: methenyltetrahydrofolate synthase domain-containing protein-like - [512888352_XP_004922073.1] | 1.16209695 | 0.6681951 |
|  | CRE-GSPD-1 protein - [308491588_XP_003107985.1] | 1.1285635 | 0.6679297 |
|  | PREDICTED: ATP-binding cassette sub-family G member 1-like - [512919814_XP_004929476.1] | #N/A | 0.6678816 |
|  | PREDICTED: D-aspartate oxidase-like - [512939316_XP_004934211.1] | 1.07940906 | 0.6678552 |
|  | PREDICTED: aspartate aminotransferase, cytoplasmic-like isoform X1 - [512892870_XP_004923039.1] | 1.15976255 | 0.6677324 |
|  | PREDICTED: V-type proton ATPase subunit D 1-like - [498954026_XP_004523596.1] | 1.17839265 | 0.6675263 |
|  | PREDICTED: uncharacterized protein LOC101744553 - [512905640_XP_004925995.1] | 1.22904533 | 0.6674544 |
|  | PREDICTED: methionine--tRNA ligase, cytoplasmic-like - [512900847_XP_004924991.1] | 1.15755009 | 0.6673083 |
|  | adenosine kinase - [114051596_NP_001040165.1] | 1.18899704 | 0.6672069 |
|  | PREDICTED: tryptophan--tRNA ligase, cytoplasmic-like - [512886734_XP_004921808.1] | 1.13166211 | 0.6669265 |
|  | PREDICTED: probable alpha-aspartyl dipeptidase-like - [512925795_XP_004930926.1] | #N/A | 0.6667967 |
|  | PREDICTED: uncharacterized protein LOC101736596 - [512936630_XP_004933564.1] | 1.12592503 | 0.6666291 |
|  | peptidyl-prolyl cis-trans isomerase - [114051057_NP_001040140.1] | 1.16772871 | 0.6664314 |
|  | PREDICTED: huntingtin-like - [512888716_XP_004922132.1] | #N/A | 0.6662939 |
|  | PREDICTED: translin-associated protein X-like - [512911399_XP_004927402.1] | 1.37767762 | 0.6660571 |
|  | proteasome beta-subunit - [114052034_NP_001040204.1] | 1.16695052 | 0.6659652 |
|  | PREDICTED: T-complex protein 1 subunit epsilon-like - [512935385_XP_004933262.1] | 1.13167125 | 0.6659151 |
|  | PREDICTED: tripeptidyl-peptidase 2-like isoform 2 - [345495666_XP_003427548.1] | 1.23419013 | 0.6657784 |
|  | PREDICTED: phosphoacetylglucosamine mutase-like - [512934601_XP_004933071.1] | 1.24871354 | 0.6653727 |
|  | nucleoplasmin isoform 2 - [114052609_NP_001040261.1] | 1.07799826 | 0.6652494 |
|  | PREDICTED: biotin--protein ligase-like - [512927751_XP_004931413.1] | #N/A | 0.6651726 |
|  | PREDICTED: protein Mo25-like - [498959928_XP_004524554.1] | 1.11312537 | 0.6646133 |
|  | PREDICTED: transportin-1 isoform 2 - [391342940_XP_003745773.1] | #N/A | 0.6643444 |
|  | PREDICTED: cytosolic non-specific dipeptidase-like - [512915932_XP_004928508.1] | #N/A | 0.6642265 |
|  | PREDICTED: tubulin-folding cofactor B-like isoform X1 - [512902838_XP_004925473.1] | 1.12051336 | 0.6641366 |
|  | PREDICTED: protein diaphanous-like - [512925238_XP_004930790.1] | #N/A | 0.663979 |
|  | GF11862 - [194755026_XP_001959793.1] | #N/A | 0.6638333 |
|  | PREDICTED: LOW QUALITY PROTEIN: pre-mRNA-processing factor 6-like - [512929066_XP_004931733.1] | #N/A | 0.6632576 |
|  | PREDICTED: putative deoxyribose-phosphate aldolase-like - [512907512_XP_004926461.1] | 1.10952458 | 0.663238 |
|  | proteasome alpha 3 subunit - [114051245_NP_001040387.1] | 1.15374866 | 0.6632084 |
|  | PREDICTED: LOW QUALITY PROTEIN: TAF5-like RNA polymerase II p300/CBP-associated factor-associated factor 65 kDa subunit 5L-like - [512924701_XP_004930659.1] | 1.10232322 | 0.6630961 |
|  | PREDICTED: acylamino-acid-releasing enzyme-like isoform X1 - [512887894_XP_004921997.1] | 1.12266823 | 0.6629182 |
|  | PREDICTED: LOW QUALITY PROTEIN: estrogen sulfotransferase-like - [512932611_XP_004932588.1] | 1.32022689 | 0.662697 |
|  | PREDICTED: 26S proteasome non-ATPase regulatory subunit 8-like - [512903376_XP_004925604.1] | 1.18627293 | 0.6625922 |
|  | PREDICTED: N(G),N(G)-dimethylarginine dimethylaminohydrolase 1-like - [512921214_XP_004929823.1] | 1.20422294 | 0.6625543 |
|  | PREDICTED: proteasomal ATPase-associated factor 1-like - [512922207_XP_004930064.1] | #N/A | 0.6625316 |
|  | PREDICTED: zinc finger protein 845-like - [512937449_XP_004933769.1] | #N/A | 0.6622926 |
|  | hypothetical protein - [256083090_XP_002577783.1] | 1.22214897 | 0.6620821 |
|  | GF20988 - [194763661_XP_001963951.1] | 1.19465639 | 0.6618639 |
|  | PREDICTED: phospholipase A-2-activating protein-like - [383864085_XP_003707510.1] | 1.22313335 | 0.661821 |
|  | GK10953 - [195445998_XP_002070580.1] | #N/A | 0.6617814 |
|  | PREDICTED: mitochondrial sodium/hydrogen exchanger 9B2-like - [512919784_XP_004929469.1] | 1.00979587 | 0.6617012 |
|  | PREDICTED: heat shock 70 kDa protein 4L-like - [340711022_XP_003394081.1] | 1.15101535 | 0.6614792 |
|  | snap-scaffold2945_size65876-abinit-gene-0.8-mRNA-1 | #N/A | 0.6613157 |
|  | ribosomal protein S12 - [112982671_NP_001037568.1] | 1.11400056 | 0.6612164 |
|  | PREDICTED: pre-mRNA-processing factor 19-like isoform 2 - [193657225_XP_001948045.1] | #N/A | 0.6608639 |
|  | PREDICTED: transcriptional activator protein Pur-alpha-like - [512894825_XP_004923523.1] | 1.1799645 | 0.6608271 |
|  | PREDICTED: prostaglandin reductase 1-like - [512932417_XP_004932539.1] | 1.12796361 | 0.6607942 |
|  | nuclear excision repair protein rad23 - [283945482_NP_001164652.1] | 1.14274794 | 0.6603798 |
|  | PREDICTED: protein ELYS-like - [512933957_XP_004932919.1] | #N/A | 0.6603456 |
|  | PREDICTED: DNA-directed RNA polymerase III subunit RPC3-like isoform X1 - [512902589_XP_004925413.1] | 1.11179646 | 0.66033 |
|  | PREDICTED: isocitrate dehydrogenase 1 (NADP+), soluble-like - [291233358_XP_002736618.1] | 1.13290139 | 0.660322 |
|  | PREDICTED: phosphoglucomutase-2-like - [512919651_XP_004929437.1] | 1.16736238 | 0.6601303 |
|  | PREDICTED: LOW QUALITY PROTEIN: protein transport protein Sec24C-like - [512908267_XP_004926639.1] | #N/A | 0.6599612 |
|  | PREDICTED: histone-lysine N-methyltransferase eggless-like, partial - [512925530_XP_004930862.1] | 1.42065126 | 0.6598674 |
|  | PREDICTED: ubiquitin fusion degradation protein 1 homolog - [512917250_XP_004928842.1] | #N/A | 0.659854 |
|  | PREDICTED: uncharacterized protein C6orf203-like - [512924588_XP_004930631.1] | 1.09390486 | 0.6598539 |
|  | PREDICTED: serine/threonine-protein kinase GA29083-like - [512916901_XP_004928754.1] | #N/A | 0.6597259 |
|  | PREDICTED: phosphorylated adapter RNA export protein-like - [512890074_XP_004922448.1] | 1.16944626 | 0.6594902 |
|  | PREDICTED: hypothetical protein LOC100649135 - [340715393_XP_003396199.1] | #N/A | 0.659388 |
|  | casein kinase 2 beta subunit - [112983394_NP_001036989.1] | 1.1444244 | 0.65938 |
|  | Sr protein - [114050755_NP_001040152.1] | 1.20518163 | 0.6593264 |
|  | PREDICTED: torsin-like protein-like isoform X1 - [512916293_XP_004928599.1] | 1.17086025 | 0.6593136 |
|  | PREDICTED: uncharacterized protein LOC101736086 - [512895634_XP_004923720.1] | 0.99826986 | 0.6593116 |
|  | PREDICTED: similar to CG3430 CG3430-PA - [91078452_XP_967242.1] | 1.1415583 | 0.6590849 |
|  | PREDICTED: huntingtin-interacting protein K-like - [512896894_XP_004924020.1] | 1.06650033 | 0.659079 |
|  | ubiquitin ligase - [256077780_XP_002575178.1] | 1.15553249 | 0.6590397 |
|  | snap-scaffold745_size200421-abinit-gene-1.3-mRNA-1 | #N/A | 0.65902 |
|  | Mn superoxide dismutase - [112983802_NP_001037299.1] | 1.15699253 | 0.6590125 |
|  | glutaredoxin - [114052446_NP_001040246.1] | 1.11384993 | 0.6589138 |
|  | 26S proteasome non-ATPase regulatory subunit 14 - [114052633_NP_001040263.1] | 1.16422903 | 0.6588441 |
|  | predicted protein - [156390342_XP_001635230.1] | 1.21784698 | 0.6586125 |
|  | PREDICTED: sorting nexin-12-like isoform X1 - [512924855_XP_004930695.1] | 1.10331656 | 0.6583914 |
|  | PREDICTED: omega-amidase NIT2-like isoform X2 - [512901075_XP_004925046.1] | #N/A | 0.6583908 |
|  | tropomyosin-2 isoform 3 - [160333857_NP_001103783.1] | 1.12284924 | 0.6583891 |
|  | PREDICTED: T-complex protein 11-like protein 1-like - [512891136_XP_004922632.1] | #N/A | 0.6582259 |
|  | PREDICTED: D-aspartate oxidase-like - [512939316_XP_004934211.1] | 1.14917942 | 0.6581659 |
|  | PREDICTED: actin-interacting protein 1-like - [391339363_XP_003744021.1] | 1.13136306 | 0.6581253 |
|  | BAG domain-containing protein Samui - [112983960_NP_001036843.1] | 1.17995503 | 0.6581178 |
|  | PREDICTED: GTP-binding protein 128up-like isoform X1 - [512897332_XP_004924127.1] | 1.16725505 | 0.6573293 |
|  | PREDICTED: ancient ubiquitous protein 1-like - [512934192_XP_004932974.1] | 1.28868683 | 0.6573155 |
|  | PREDICTED: LOW QUALITY PROTEIN: protein FAM76A-like - [512897014_XP_004924049.1] | 1.15162991 | 0.657214 |
|  | glutathione S-transferase omega 1 - [114052242_NP_001040131.1] | 1.09604145 | 0.6566937 |
|  | PREDICTED: hexosaminidase D-like - [512888598_XP_004922112.1] | 1.13087473 | 0.6566817 |
|  | PREDICTED: similar to CG8080 CG8080-PA, partial - [91086081_XP_974309.1] | 1.19367132 | 0.6565474 |
|  | Mod(mdg4)-heS00531 - [163838692_NP_001106229.1] | 1.27070884 | 0.6565021 |
|  | PREDICTED: myrosinase 1-like isoform X2 - [512906269_XP_004926154.1] | 1.09192724 | 0.6564751 |
|  | PREDICTED: phosphatidylglycerophosphatase and protein-tyrosine phosphatase 1-like isoform X1 - [512894325_XP_004923402.1] | 1.10633023 | 0.6559908 |
|  | PREDICTED: zygotic DNA replication licensing factor mcm6-B-like - [340380288_XP_003388655.1] | 1.12598475 | 0.6554881 |
|  | PREDICTED: serine hydrolase-like protein 2-like - [512907619_XP_004926488.1] | 1.16551308 | 0.6554733 |
|  | PREDICTED: pyruvate kinase isoform 1 - [345489900_XP_001600651.2] | 1.15644141 | 0.6550466 |
|  | proliferating cell nuclear antigen - [112984050_NP_001036825.1] | 1.13149846 | 0.6550258 |
|  | PREDICTED: serine/threonine-protein kinase PAK 1-like isoform X1 - [512899491_XP_004924658.1] | #N/A | 0.6548106 |
|  | AGAP001043-PA - [347965016_XP_309245.5] | 1.04810948 | 0.6547828 |
|  | PREDICTED: GMP synthase [glutamine-hydrolyzing]-like - [524907909_XP_005109065.1] | 1.16052426 | 0.6547411 |
|  | PREDICTED: maternal protein tudor-like - [512928832_XP_004931676.1] | 1.11345731 | 0.6546992 |
|  | PREDICTED: LOW QUALITY PROTEIN: serine/arginine-rich splicing factor 1A-like - [512917969_XP_004929016.1] | 1.23103499 | 0.6544432 |
|  | PREDICTED: regulator of chromosome condensation-like - [512893358_XP_004923161.1] | 1.17190269 | 0.6542246 |
|  | zinc finger protein - [157138655_XP_001657329.1] | #N/A | 0.6541939 |
|  | PREDICTED: threonine--tRNA ligase, cytoplasmic-like isoform X2 - [512936200_XP_004933460.1] | 1.26174478 | 0.6541377 |
|  | GH11436 - [195034592_XP_001988931.1] | 1.19373235 | 0.6541228 |
|  | PREDICTED: fatty acid synthase-like - [512891939_XP_004922807.1] | #N/A | 0.6540967 |
|  | PREDICTED: sorting nexin-6-like isoform X1 - [512925207_XP_004930783.1] | 1.14263311 | 0.6540757 |
|  | PREDICTED: proteasome subunit beta type-2-like isoform X1 - [512914947_XP_004928268.1] | 1.16345863 | 0.6538057 |
|  | PREDICTED: acylamino-acid-releasing enzyme-like - [328786429_XP_001123355.2] | 1.18848681 | 0.6537889 |
|  | GI17019 - [195114386_XP_002001748.1] | 1.10890118 | 0.6537288 |
|  | PREDICTED: tudor and KH domain-containing protein-like - [512906954_XP_004926323.1] | 1.14948143 | 0.6536273 |
|  | GJ15472 - [195402935_XP_002060055.1] | 1.24453987 | 0.6536113 |
|  | PREDICTED: proteasomal ubiquitin receptor ADRM1 homolog - [193683329_XP_001949000.1] | 1.19705597 | 0.6533447 |
|  | PREDICTED: broad-complex core protein isoforms 1/2/3/4/5-like isoform X1 - [512926194_XP_004931024.1] | 1.15694961 | 0.6532423 |
|  | GD13184 - [195588000_XP_002083749.1] | 1.12545924 | 0.6526732 |
|  | 26s proteasome regulatory particle subunit - [256070245_XP_002571453.1] | 1.20187507 | 0.6526503 |
|  | glutamate cysteine ligase - [157117953_XP_001653117.1] | #N/A | 0.6523909 |
|  | AGAP002597-PA - [347968149_XP_312339.5] | 1.0447061 | 0.6519732 |
|  | PREDICTED: UDP-N-acetylglucosamine--dolichyl-phosphate N-acetylglucosaminephosphotransferase-like isoform X1 - [512903140_XP_004925546.1] | 1.27732761 | 0.6519719 |
|  | PREDICTED: dedicator of cytokinesis protein 11 isoform 1 - [328717510_XP_001946761.2] | #N/A | 0.6518275 |
|  | PREDICTED: serine/arginine repetitive matrix protein 1-like isoform X1 - [512907014_XP_004926338.1] | 1.17035621 | 0.6512879 |
|  | PREDICTED: probable glutamate--tRNA ligase, mitochondrial-like - [512892956_XP_004923061.1] | #N/A | 0.6509435 |
|  | PREDICTED: uncharacterized protein LOC100879429 - [383862945_XP_003706943.1] | #N/A | 0.6508967 |
|  | PREDICTED: maestro heat-like repeat-containing protein family member 1-like - [512919303_XP_004929342.1] | 1.1466911 | 0.6508246 |
|  | GJ21556 - [195381295_XP_002049388.1] | 1.21650792 | 0.650677 |
|  | PREDICTED: similar to AGAP009743-PA - [189240495_XP_968692.2] | 1.13833075 | 0.6506567 |
|  | C. briggsae CBR-ARX-1 protein - [268565923_XP_002639586.1] | 1.17118331 | 0.6506448 |
|  | snap-scaffold3358_size79210-abinit-gene-0.23-mRNA-1 | #N/A | 0.6503092 |
|  | PREDICTED: ubiquitin-conjugating enzyme E2 variant 2-like - [512928183_XP_004931520.1] | 1.03805886 | 0.6502819 |
|  | PREDICTED: nischarin-like - [383855512_XP_003703254.1] | #N/A | 0.6502675 |
|  | maker-scaffold4689_size36696-snap-gene-0.5-mRNA-1 | 1.00954402 | 0.650234 |
|  | nucleosome assembly protein isoform 1 - [290560655_NP_001166838.1] | 1.10288918 | 0.6501034 |
|  | PREDICTED: myc box-dependent-interacting protein 1-like - [512892438_XP_004922931.1] | 1.36422851 | 0.6501003 |
|  | phenylalanyl-tRNA synthetase subunit beta - [256088450_XP_002580348.1] | 1.1500951 | 0.6500894 |
|  | PREDICTED: glutaredoxin 3-like - [512924346_XP_004930573.1] | 1.11647424 | 0.6500668 |
|  | ATP synthase gamma-related - [256084407_XP_002578421.1] | 1.15916173 | 0.6500383 |
|  | PREDICTED: LOW QUALITY PROTEIN: GPI mannosyltransferase 3-like - [512895955_XP_004923797.1] | 1.15551233 | 0.6498375 |
|  | cystathionine beta-lyase - [157104407_XP_001648395.1] | 1.15163909 | 0.6497204 |
|  | vacuolar protein sorting-associated protein VTA1 homolog - [114050853_NP_001040410.1] | 1.18604797 | 0.6496504 |
|  | sex combs on midleg - [319803031_NP_001188365.1] | 1.19221552 | 0.6496381 |
|  | snap-scaffold3456_size45251-abinit-gene-0.0-mRNA-1 | 1.27438552 | 0.649562 |
|  | PREDICTED: monocarboxylate transporter 4-like - [512923062_XP_004930261.1] | #N/A | 0.6494898 |
|  | PREDICTED: programmed cell death protein 2-like - [512929730_XP_004931895.1] | 1.20647813 | 0.6493556 |
|  | PREDICTED: AP-3 complex subunit sigma-2-like - [512935621_XP_004933318.1] | #N/A | 0.6491084 |
|  | PREDICTED: adenylate cyclase type 10-like - [512890345_XP_004922501.1] | #N/A | 0.6490357 |
|  | PREDICTED: uncharacterized protein LOC101746153 - [512921082_XP_004929794.1] | 1.25447865 | 0.6487697 |
|  | DNA replication licensing factor MCM3 - [256077653_XP_002575116.1] | 1.1099282 | 0.6486074 |
|  | PREDICTED: LOW QUALITY PROTEIN: bromodomain adjacent to zinc finger domain protein 1A-like - [512904705_XP_004925762.1] | 1.17595579 | 0.6485123 |
|  | PREDICTED: protein CutA homolog - [512926846_XP_004931187.1] | 1.19147822 | 0.6484002 |
|  | PREDICTED: drebrin-like protein-like - [512934261_XP_004932992.1] | #N/A | 0.6480457 |
|  | PREDICTED: cyclin-H-like - [512904801_XP_004925786.1] | 1.14264379 | 0.6479835 |
|  | hypothetical protein - [256083090_XP_002577783.1] | 1.21322674 | 0.6479359 |
|  | vacuolar ATP synthase subunit E - [114052088_NP_001040451.1] | 1.14691533 | 0.6478815 |
|  | PREDICTED: PCI domain-containing protein 2 homolog - [512937136_XP_004933693.1] | 1.14610536 | 0.6477337 |
|  | trafficking protein particle complex subunit 3 - [114051652_NP_001040170.1] | 1.08083351 | 0.6476863 |
|  | PREDICTED: pre-mRNA-splicing factor ATP-dependent RNA helicase PRP16-like - [498972606_XP_004527354.1] | 1.21649724 | 0.647557 |
|  | legumaturain - [114053035_NP_001040501.1] | #N/A | 0.6474949 |
|  | hypothetical protein CRE_13033 - [308466794_XP_003095648.1] | #N/A | 0.6474399 |
|  | CMGC/CK2 protein kinase - [312071564_XP_003138666.1] | 1.19497751 | 0.6473124 |
|  | GTP binding protein - [114053313_NP_001040339.1] | 1.15297688 | 0.6473098 |
|  | PREDICTED: serine/threonine-protein kinase tricorner-like isoform X2 - [512900656_XP_004924943.1] | #N/A | 0.6472296 |
|  | GI20156 - [195120732_XP_002004875.1] | 1.21292866 | 0.6468666 |
|  | PREDICTED: RNA-binding protein 5-like isoform X1 - [512898263_XP_004924357.1] | 1.30076994 | 0.6467975 |
|  | PREDICTED: protein ROP-like isoform X1 - [512916317_XP_004928610.1] | 1.48686856 | 0.6467036 |
|  | PREDICTED: tubulin-specific chaperone C-like - [512919659_XP_004929439.1] | 1.22710839 | 0.6465991 |
|  | PREDICTED: protein maelstrom homolog - [512915400_XP_004928380.1] | #N/A | 0.646536 |
|  | PREDICTED: nardilysin-like - [350424656_XP_003493869.1] | #N/A | 0.6463178 |
|  | PREDICTED: 60S acidic ribosomal protein P1-like - [340730119_XP_003403334.1] | 1.12440022 | 0.6463043 |
|  | PREDICTED: atlastin-like - [512903840_XP_004925716.1] | 1.13589453 | 0.6462889 |
|  | PREDICTED: LOW QUALITY PROTEIN: ubiquitin thioesterase otubain-like - [512919495_XP_004929397.1] | 1.10634187 | 0.6460171 |
|  | PREDICTED: probable ATP-dependent RNA helicase DDX46-like isoform X1 - [512929039_XP_004931727.1] | #N/A | 0.6459273 |
|  | PREDICTED: similar to egalitarian CG4051-PA - [91084227_XP_969046.1] | #N/A | 0.6457186 |
|  | PREDICTED: nuclear pore complex protein Nup50-like - [512914004_XP_004928033.1] | 1.16545178 | 0.6452688 |
|  | PREDICTED: dynein heavy chain 6, axonemal-like - [328713942_XP_003245215.1] | #N/A | 0.6451423 |
|  | PREDICTED: similar to microtubule associated protein xmap215 - [189234292_XP_970495.2] | 1.16954789 | 0.6447453 |
|  | GK17594 - [195428978_XP_002062541.1] | #N/A | 0.6446491 |
|  | GTP cyclohydrolase I isoform A - [290560920_NP_001166803.1] | #N/A | 0.6444623 |
|  | PREDICTED: uncharacterized protein LOC101736820 - [512929817_XP_004931916.1] | 1.09465614 | 0.644282 |
|  | Probable ATP-dependent RNA helicase A - [170587044_XP_001898289.1] | 1.12474225 | 0.6441518 |
|  | PREDICTED: NADH dehydrogenase (ubiquinone) complex I, assembly factor 6-like - [512926012_XP_004930978.1] | #N/A | 0.6439418 |
|  | PREDICTED: dihydropteridine reductase-like - [512890095_XP_004922453.1] | 1.19408817 | 0.643915 |
|  | phosphoglyceromutase - [112982822_NP_001037540.1] | 1.18407956 | 0.643692 |
|  | PREDICTED: copia protein-like - [328698057_XP_003240529.1] | #N/A | 0.6432893 |
|  | PREDICTED: uncharacterized protein LOC101742343 - [512907269_XP_004926400.1] | #N/A | 0.6432198 |
|  | PREDICTED: partitioning defective protein 6-like - [512890499_XP_004922525.1] | 1.09678731 | 0.6431479 |
|  | PREDICTED: nudix hydrolase 8-like - [512905194_XP_004925883.1] | 1.14941271 | 0.6430588 |
|  | PREDICTED: cytoplasmic dynein 1 light intermediate chain 2-like, partial - [512918594_XP_004929172.1] | 1.24477549 | 0.6430154 |
|  | PREDICTED: phosphoribosylformylglycinamidine synthase-like - [390366538_XP_781955.3] | 1.12923329 | 0.6429621 |
|  | PREDICTED: ATP-citrate synthase-like - [383865717_XP_003708319.1] | 1.1610944 | 0.6429611 |
|  | PREDICTED: ataxin-2-like protein-like - [512918291_XP_004929097.1] | 1.18858791 | 0.6428879 |
|  | hypothetical protein CRE_07953 - [308450405_XP_003088287.1] | 1.17286254 | 0.6426582 |
|  | p23-like protein - [112983282_NP_001036958.1] | 1.190953 | 0.642636 |
|  | Zinc knuckle family protein - [170586914_XP_001898224.1] | 1.05166344 | 0.6424822 |
|  | PREDICTED: VID27-like protein-like - [512928051_XP_004931487.1] | 1.16223555 | 0.6424114 |
|  | PREDICTED: NAD(P)H-hydrate epimerase-like isoform X1 - [512898061_XP_004924306.1] | 1.06328734 | 0.6422102 |
|  | PREDICTED: peroxisomal targeting signal 1 receptor-like - [512923652_XP_004930404.1] | 1.1317789 | 0.6422014 |
|  | PREDICTED: uncharacterized protein LOC101739845 isoform X1 - [512931715_XP_004932371.1] | 1.18670069 | 0.6420481 |
|  | probable elongator complex protein 3 - [339238525_XP_003380817.1] | 1.20997818 | 0.641761 |
|  | snap-scaffold1194_size49489-abinit-gene-0.10-mRNA-1 | 1.1172292 | 0.6413804 |
|  | PREDICTED: COP9 signalosome complex subunit 8-like isoform X1 - [512926116_XP_004931005.1] | 1.06336506 | 0.6412536 |
|  | PREDICTED: RING finger and CHY zinc finger domain-containing protein 1-like - [512897761_XP_004924232.1] | #N/A | 0.6410854 |
|  | vacuolar ATPase subunit C - [114050729_NP_001040138.1] | 1.12801882 | 0.641028 |
|  | PREDICTED: 26S protease regulatory subunit 7-like isoform X1 - [512896702_XP_004923974.1] | 1.17881785 | 0.6409918 |
|  | PREDICTED: AT-rich interactive domain-containing protein 2-like - [512891588_XP_004922722.1] | #N/A | 0.6409221 |
|  | PREDICTED: protein MEMO1-like - [512889313_XP_004922245.1] | 1.08594465 | 0.6407139 |
|  | PREDICTED: phosphatidylinositol transfer protein alpha isoform-like - [512917375_XP_004928871.1] | 1.20747925 | 0.6403852 |
|  | PREDICTED: GDP-L-fucose synthase-like - [512937333_XP_004933742.1] | 1.11215658 | 0.6402744 |
|  | uncharacterized protein LOC778505 - [148298707_NP_001091800.1] | 1.19007999 | 0.6402529 |
|  | thioredoxin - [114052058_NP_001040348.1] | 1.15912256 | 0.6401149 |
|  | PREDICTED: serine/threonine-protein kinase OSR1-like - [391334187_XP_003741489.1] | 1.11558527 | 0.6399506 |
|  | pyridoxal kinase - [112984192_NP_001037440.1] | #N/A | 0.6399382 |
|  | ubiquitin-conjugating enzyme E2M - [114052396_NP_001040241.1] | 1.04838058 | 0.6399173 |
|  | PREDICTED: G-protein-signaling modulator 2-like - [512900692_XP_004924952.1] | 1.17991153 | 0.6397426 |
|  | AGAP010711-PA - [31204951_XP_311424.1] | 1.13569818 | 0.6397205 |
|  | PREDICTED: squamous cell carcinoma antigen recognized by T-cells 3-like - [512917400_XP_004928877.1] | 1.10869772 | 0.6394432 |
|  | PREDICTED: dedicator of cytokinesis protein 1-like - [512888695_XP_004922128.1] | #N/A | 0.6393611 |
|  | phosphotriesterase-like protein - [312261191_NP_001185957.1] | 1.10956072 | 0.6391982 |
|  | PREDICTED: proteasome subunit beta type-4-like - [512935468_XP_004933282.1] | 1.14343443 | 0.6391729 |
|  | GH22287 - [195062839_XP_001996263.1] | 1.24315589 | 0.6388695 |
|  | PREDICTED: golgi-specific brefeldin A-resistance guanine nucleotide exchange factor 1-like isoform 1 - [390353526_XP_003728128.1] | #N/A | 0.6386532 |
|  | PREDICTED: protein PRRC1-like - [512889974_XP_004922420.1] | 1.21361264 | 0.6386235 |
|  | GH23147 - [195058262_XP_001995418.1] | 1.17376746 | 0.6385089 |
|  | PREDICTED: nucleobindin-2-like isoform X2 - [512921230_XP_004929827.1] | 1.23776914 | 0.638488 |
|  | PREDICTED: serine-rich adhesin for platelets-like - [498950614_XP_004523045.1] | #N/A | 0.6384485 |
|  | PREDICTED: inositol 1,4,5-trisphosphate receptor-like - [512895244_XP_004923625.1] | 1.23960691 | 0.6382328 |
|  | Pao retrotransposon peptidase superfamily - [339257282_XP_003370011.1] | #N/A | 0.638197 |
|  | p38 map kinase - [112983386_NP_001036996.1] | 1.14286589 | 0.637969 |
|  | CG10413 - [19921484_NP_609887.1] | 1.0784856 | 0.6379011 |
|  | PREDICTED: prostaglandin reductase 1-like - [512932417_XP_004932539.1] | #N/A | 0.6377821 |
|  | PREDICTED: probable phospholipid-transporting ATPase IA-like - [345496886_XP_003427844.1] | #N/A | 0.6375814 |
|  | hypothetical protein CRE_08117 - [308494260_XP_003109319.1] | 1.14734199 | 0.6374663 |
|  | PREDICTED: cytoplasmic phosphatidylinositol transfer protein 1-like - [512930778_XP_004932144.1] | 1.14228845 | 0.6373756 |
|  | PREDICTED: uncharacterized protein LOC101737963 - [512924599_XP_004930634.1] | 1.17520824 | 0.6371176 |
|  | CRE-CCT-8 protein - [308461629_XP_003093105.1] | 1.10889163 | 0.6370866 |
|  | PREDICTED: proteasome assembly chaperone 2-like - [512927377_XP_004931319.1] | 1.17465651 | 0.6370757 |
|  | C. briggsae CBR-NRS-1 protein - [268562070_XP_002646595.1] | 1.20758525 | 0.6370402 |
|  | nuclear factor NF-kappa-B p110 subunit isoform 2 - [157412326_NP_001098704.1] | 1.21970119 | 0.6368661 |
|  | sec13-like protein - [114051650_NP_001040420.1] | 1.18215292 | 0.6368059 |
|  | AGAP012155-PA - [158300462_XP_320377.4] | 1.28489824 | 0.6362538 |
|  | acid phosphatase isoenzyme - [114051630_NP_001040167.1] | #N/A | 0.636235 |
|  | PREDICTED: uncharacterized protein LOC101739024 - [512907437_XP_004926441.1] | 1.16645738 | 0.6361989 |
|  | snap-scaffold9161_size8884-abinit-gene-0.0-mRNA-1 | #N/A | 0.6360904 |
|  | ATPase family protein - [339240921_XP_003376386.1] | 1.11614882 | 0.6357677 |
|  | PREDICTED: alcohol dehydrogenase class-3-like - [499011058_XP_004536957.1] | #N/A | 0.6356949 |
|  | snap-scaffold11319_size5567-abinit-gene-0.0-mRNA-1 | 1.21409633 | 0.6356155 |
|  | PREDICTED: chitinase domain-containing protein 1-like - [512929135_XP_004931749.1] | #N/A | 0.6356145 |
|  | PREDICTED: YTH domain family protein 3-like isoform X1 - [512899283_XP_004924605.1] | 1.18449447 | 0.6354806 |
|  | cytosolic juvenile hormone binding protein 36 kDa subunit - [112983082_NP_001037668.1] | 1.14838287 | 0.6354247 |
|  | PREDICTED: similar to snRNA-activating protein complex subunit 3 (SNAPc subunit 3) (Small nuclear RNA-activating complex polypeptide 3) (snRNA-activating protein complex 50 kDa subunit) (SNAPc 50 kDa subunit) (Proximal sequence element-binding transcription f - [91076534_XP_973776.1] | #N/A | 0.635046 |
|  | PREDICTED: TAR DNA-binding protein 43-like - [512900157_XP_004924823.1] | #N/A | 0.6349641 |
|  | PREDICTED: pyridoxal-dependent decarboxylase domain-containing protein 1-like - [512908447_XP_004926684.1] | 1.10926084 | 0.634605 |
|  | PREDICTED: aldose reductase-like isoform X1 - [512901366_XP_004925118.1] | 1.09305176 | 0.6346026 |
|  | PREDICTED: V-type proton ATPase catalytic subunit A-like - [383848807_XP_003700039.1] | 1.15124764 | 0.634367 |
|  | PREDICTED: 3-ketodihydrosphingosine reductase-like - [512932805_XP_004932635.1] | 1.15782062 | 0.6341804 |
|  | antennal-enriched UDP-glycosyltransferase precursor - [114051706_NP_001040425.1] | 1.07203453 | 0.6338149 |
|  | PREDICTED: nuclear pore complex protein Nup107-like - [512902916_XP_004925491.1] | 1.17179375 | 0.6337776 |
|  | potassium channel regulator - [157116994_XP_001652924.1] | 1.1562635 | 0.6337411 |
|  | PREDICTED: importin-11 - [193591743_XP_001945969.1] | 1.28632162 | 0.6333322 |
|  | phenol UDP-glucosyltransferase precursor - [112983138_NP_001037040.1] | #N/A | 0.6332769 |
|  | CRE-RPT-4 protein - [308470578_XP_003097522.1] | 1.2238022 | 0.6331936 |
|  | dynein molecular motor protein light chain 1 - [153791737_NP_001093289.1] | 1.21691626 | 0.6331709 |
|  | PREDICTED: tubulin--tyrosine ligase-like protein 12-like isoform X1 - [499013415_XP_004537531.1] | 1.12555219 | 0.6330889 |
|  | PREDICTED: neuferricin homolog - [512923675_XP_004930410.1] | 1.34089218 | 0.6329416 |
|  | PREDICTED: protein THEM6-like - [512929810_XP_004931915.1] | #N/A | 0.6328868 |
|  | PREDICTED: calcium homeostasis endoplasmic reticulum protein-like isoform X1 - [512930031_XP_004931968.1] | #N/A | 0.6328823 |
|  | PREDICTED: thyroid receptor-interacting protein 11-like - [512895859_XP_004923773.1] | 1.15565076 | 0.6328538 |
|  | PREDICTED: ATPase WRNIP1-like isoform 1 - [328792628_XP_392908.4] | 1.18469859 | 0.6328344 |
|  | PREDICTED: protein transport protein SFT2-like - [512937991_XP_004933898.1] | 1.14803648 | 0.6327284 |
|  | PREDICTED: guanine nucleotide-binding protein-like 1-like - [512889744_XP_004922361.1] | 1.12898349 | 0.6326926 |
|  | hypothetical protein AaeL_AAEL003882 - [157138597_XP_001664271.1] | 1.22827561 | 0.6325832 |
|  | PREDICTED: uncharacterized protein LOC101743665 - [512892743_XP_004923007.1] | 1.28445506 | 0.6324138 |
|  | PREDICTED: vacuolar fusion protein MON1 homolog A-like - [512936822_XP_004933613.1] | 1.12485974 | 0.6322671 |
|  | proteasome beta subunit - [151301141_NP_001093092.1] | 1.16313641 | 0.6321969 |
|  | PREDICTED: trans-1,2-dihydrobenzene-1,2-diol dehydrogenase-like isoform X1 - [512922290_XP_004930084.1] | 1.15886618 | 0.6320838 |
|  | GD25552 - [195583880_XP_002081744.1] | 1.21305819 | 0.6318686 |
|  | PREDICTED: LOW QUALITY PROTEIN: serine palmitoyltransferase 2-like - [512909511_XP_004926938.1] | #N/A | 0.631388 |
|  | PREDICTED: serine/threonine-protein phosphatase 6 regulatory subunit 3-like - [345497186_XP_001599593.2] | 0.99849277 | 0.6313218 |
|  | pelota - [157117107_XP_001658703.1] | 1.14504639 | 0.6312034 |
|  | PREDICTED: ubiquitin-like modifier-activating enzyme ATG7 - [390340346_XP_792811.3] | #N/A | 0.6311635 |
|  | PREDICTED: probable dynactin subunit 2-like - [512895322_XP_004923644.1] | 1.25546806 | 0.631127 |
|  | PREDICTED: zinc finger CCCH domain-containing protein 15 homolog isoform X1 - [512928574_XP_004931611.1] | 1.19068482 | 0.6311269 |
|  | GE16527 - [195470138_XP_002087365.1] | 1.127888 | 0.6309332 |
|  | PREDICTED: uncharacterized protein LOC101240437 - [449672876_XP_004207813.1] | #N/A | 0.6308757 |
|  | PREDICTED: RNA-binding protein 26-like - [512925443_XP_004930841.1] | 1.23886086 | 0.6308487 |
|  | PREDICTED: probable pseudouridine-5'-monophosphatase-like isoform X1 - [512927444_XP_004931336.1] | 1.09900368 | 0.6308434 |
|  | PREDICTED: uncharacterized protein LOC101737914 isoform X1 - [512915341_XP_004928366.1] | #N/A | 0.6307986 |
|  | PREDICTED: UTP--glucose-1-phosphate uridylyltransferase | 1.18305889 | 0.630709 |
|  | PREDICTED: uncharacterized protein LOC101744068 - [512896172_XP_004923847.1] | #N/A | 0.6306612 |
|  | PREDICTED: oligoribonuclease, mitochondrial-like isoform X3 - [512933121_XP_004932712.1] | 1.13379537 | 0.6303731 |
|  | PREDICTED: protein ELYS-like - [512933957_XP_004932919.1] | 1.25888205 | 0.630349 |
|  | PREDICTED: myb-like protein X-like - [512934661_XP_004933086.1] | 1.05298215 | 0.6300088 |
|  | PREDICTED: inositol 1,4,5-trisphosphate receptor-like - [512895244_XP_004923625.1] | #N/A | 0.6299955 |
|  | PREDICTED: cold shock domain-containing protein CG9705-like - [512931232_XP_004932251.1] | 1.09424922 | 0.6298567 |
|  | PREDICTED: ataxin-10-like - [512895935_XP_004923792.1] | #N/A | 0.6297638 |
|  | AGAP000776-PA - [347964482_XP_311315.5] | 1.09249316 | 0.6295411 |
|  | PREDICTED: zinc metalloproteinase nas-4-like - [512916893_XP_004928752.1] | #N/A | 0.6292168 |
|  | PREDICTED: vacuolar fusion protein CCZ1 homolog - [512930906_XP_004932176.1] | 1.18347661 | 0.6291914 |
|  | PREDICTED: hypothetical protein LOC100645109 - [340729865_XP_003403215.1] | 1.18691474 | 0.6288606 |
|  | PREDICTED: ubiquitin-conjugating enzyme E2 G2-like - [340728561_XP_003402589.1] | 1.16786241 | 0.628754 |
|  | PREDICTED: trifunctional purine biosynthetic protein adenosine-3-like - [512901569_XP_004925167.1] | 1.03379666 | 0.6287381 |
|  | PREDICTED: protein phosphatase inhibitor 2-like - [512937418_XP_004933761.1] | 1.15169854 | 0.628491 |
|  | heat shock protein hsp20.1 - [112983134_NP_001036941.1] | #N/A | 0.6282422 |
|  | PREDICTED: alpha-glucosidase | #N/A | 0.6282402 |
|  | predicted protein - [156371244_XP_001628675.1] | #N/A | 0.628231 |
|  | C. briggsae CBR-XRN-1 protein, partial - [268533038_XP_002631647.1] | 1.19165834 | 0.6280648 |
|  | predicted protein - [156386530_XP_001633965.1] | 1.21565449 | 0.6280611 |
|  | glutathione S-transferase unclassified 2 - [114052286_NP_001040130.1] | 1.15270119 | 0.6279506 |
|  | PREDICTED: chaoptin-like - [512932583_XP_004932581.1] | #N/A | 0.6279406 |
|  | PREDICTED: similar to CG8486 CG8486-PC - [189237536_XP_001813934.1] | #N/A | 0.6278875 |
|  | heat shock protein hsp21.4 - [112983414_NP_001036985.1] | 1.11162645 | 0.6278535 |
|  | PREDICTED: LOW QUALITY PROTEIN: COP9 signalosome complex subunit 4-like - [512928984_XP_004931714.1] | 1.13456083 | 0.6277832 |
|  | PREDICTED: probable ATP-dependent RNA helicase DDX20-like - [512928197_XP_004931522.1] | #N/A | 0.6277784 |
|  | PREDICTED: condensin complex subunit 3-like - [512917412_XP_004928880.1] | 1.14020986 | 0.6276359 |
|  | PREDICTED: uncharacterized protein LOC101736400 - [512907678_XP_004926502.1] | 1.01530228 | 0.627533 |
|  | PREDICTED: proteasome-associated protein ECM29 homolog - [512910450_XP_004927174.1] | 1.20710979 | 0.627397 |
|  | PREDICTED: anoctamin-1-like - [328706743_XP_001944325.2] | #N/A | 0.6273445 |
|  | C. briggsae CBR-UBXN-1 protein - [268565293_XP_002639398.1] | 1.22288428 | 0.6273092 |
|  | PREDICTED: calcium/calmodulin-dependent protein kinase type 1-like - [391343809_XP_003746198.1] | 1.18295334 | 0.6271756 |
|  | eIF2B-alpha protein - [112983332_NP_001037648.1] | 1.10640042 | 0.6270856 |
|  | PREDICTED: similar to F-box and leucine-rich repeat protein 11 - [189238300_XP_970863.2] | 1.24175411 | 0.6270673 |
|  | PREDICTED: V-type proton ATPase subunit S1-like - [512912049_XP_004927561.1] | 1.18838546 | 0.6267924 |
|  | translin - [114053281_NP_001040282.1] | 1.16331372 | 0.6267366 |
|  | cytochrome P450 9a20 - [134254438_NP_001077079.1] | 0.99543811 | 0.6267177 |
|  | PREDICTED: mucolipin-3-like - [512933889_XP_004932903.1] | #N/A | 0.626696 |
|  | PREDICTED: protein FAM136A-like - [512900787_XP_004924976.1] | 1.11520258 | 0.6266585 |
|  | PREDICTED: uncharacterized protein LOC101735506 - [512904883_XP_004925807.1] | #N/A | 0.6264525 |
|  | PREDICTED: rap1 GTPase-GDP dissociation stimulator 1-A-like - [512899916_XP_004924764.1] | 1.19599035 | 0.6264317 |
|  | PREDICTED: uncharacterized protein YJR142W-like - [512907472_XP_004926451.1] | 1.17121384 | 0.6264257 |
|  | PREDICTED: ankyrin repeat domain-containing protein 13B-like isoform X1 - [524872976_XP_005093242.1] | #N/A | 0.6262523 |
|  | PREDICTED: exocyst complex component 7-like - [512907245_XP_004926394.1] | 1.18204373 | 0.6259811 |
|  | PREDICTED: microtubule-associated protein RP/EB family member 1-like isoform 2 - [345484834_XP_001600969.2] | 1.12207393 | 0.6258164 |
|  | PREDICTED: probable exonuclease mut-7 homolog - [328776161_XP_391887.3] | #N/A | 0.6256878 |
|  | AGAP003725-PB - [347970483_XP_003436586.1] | 1.13218895 | 0.6256195 |
|  | PREDICTED: uncharacterized protein LOC101738134 - [512921346_XP_004929854.1] | #N/A | 0.6255991 |
|  | Protein SEC-23 - [17566436_NP_507877.1] | 1.17019064 | 0.6254423 |
|  | PREDICTED: transcription initiation factor IIA subunit 1-like - [512927883_XP_004931446.1] | 1.1121975 | 0.6251989 |
|  | PREDICTED: glycerophosphodiester phosphodiesterase 1-like - [512907592_XP_004926481.1] | 1.43353281 | 0.6251588 |
|  | DNA replication licensing factor MCM7 - [256076787_XP_002574691.1] | 1.09053 | 0.6244249 |
|  | ADP-ribosylation factor - [148298726_NP_001091755.1] | 1.15221347 | 0.6243068 |
|  | PREDICTED: nuclear cap-binding protein subunit 1-like - [328711986_XP_001947457.2] | 1.19327623 | 0.6242762 |
|  | PREDICTED: DNA polymerase epsilon subunit 3-like - [512893327_XP_004923153.1] | 1.14517801 | 0.6242559 |
|  | GJ16489 - [195397155_XP_002057194.1] | 1.16290542 | 0.6241434 |
|  | PREDICTED: ubiquitin-like modifier-activating enzyme 1-like - [498938566_XP_004520952.1] | 1.14715349 | 0.6240654 |
|  | PREDICTED: proteasome subunit alpha type-1-like - [512891246_XP_004922650.1] | 1.14906557 | 0.6240168 |
|  | PREDICTED: zinc finger BED domain-containing protein 4-like - [449662126_XP_004205480.1] | #N/A | 0.6237255 |
|  | PREDICTED: ubiquitin carboxyl-terminal hydrolase 20-like - [512890913_XP_004922594.1] | #N/A | 0.6234869 |
|  | PREDICTED: uncharacterized protein LOC101739024 - [512907437_XP_004926441.1] | #N/A | 0.6232948 |
|  | PREDICTED: E3 ubiquitin-protein ligase CHIP-like - [512932413_XP_004932538.1] | #N/A | 0.6232317 |
|  | PREDICTED: solute carrier family 2, facilitated glucose transporter member 1-like - [512894940_XP_004923552.1] | #N/A | 0.6230616 |
|  | UDP-glucosyltransferase precursor - [306518650_NP_001182387.1] | 1.12017439 | 0.6229836 |
|  | PREDICTED: carnitine O-palmitoyltransferase 2, mitochondrial-like - [193652791_XP_001944749.1] | 1.18851029 | 0.6229792 |
|  | glutathione S-transferase omega 2 - [112983952_NP_001037406.1] | 1.04736632 | 0.6229746 |
|  | PREDICTED: uncharacterized protein LOC101451129 - [498942037_XP_004521657.1] | #N/A | 0.6229579 |
|  | PREDICTED: STAM-binding protein-like A-like - [512926666_XP_004931143.1] | #N/A | 0.6229278 |
|  | PREDICTED: similar to transportin - [91080171_XP_970238.1] | 1.15304265 | 0.6227557 |
|  | predicted protein - [156405378_XP_001640709.1] | #N/A | 0.6226981 |
|  | PREDICTED: ribosome maturation protein SBDS-like - [512923664_XP_004930407.1] | 1.11533834 | 0.6226033 |
|  | PREDICTED: la protein homolog isoform X1 - [512918630_XP_004929181.1] | 1.09946638 | 0.6224946 |
|  | PREDICTED: protein ROP-like isoform X1 - [512916317_XP_004928610.1] | 1.4260068 | 0.6223755 |
|  | phosphorylase B kinase alpha kpb1 - [256080112_XP_002576327.1] | 1.12145568 | 0.6223494 |
|  | PREDICTED: dual specificity mitogen-activated protein kinase kinase 6-like isoform X3 - [512887768_XP_004921977.1] | 1.17272464 | 0.6222804 |
|  | hemolin-interacting protein - [112982986_NP_001037086.1] | #N/A | 0.6222604 |
|  | PREDICTED: uncharacterized protein PFB0145c-like - [512913374_XP_004927880.1] | 1.21307765 | 0.6222365 |
|  | elongation factor 1 gamma - [157128964_XP_001661570.1] | 1.15497264 | 0.6222098 |
|  | PREDICTED: carboxypeptidase A4-like - [512891959_XP_004922812.1] | 1.1541609 | 0.6222078 |
|  | PREDICTED: coiled-coil domain-containing protein 86-like - [512909320_XP_004926892.1] | 1.59139821 | 0.6218396 |
|  | AGAP000983-PA - [347964884_XP_560205.4] | #N/A | 0.621777 |
|  | PREDICTED: venom serine carboxypeptidase-like - [512917905_XP_004929002.1] | 1.10003693 | 0.621716 |
|  | PREDICTED: ero1-like protein-like - [350423512_XP_003493504.1] | 1.3175064 | 0.6212661 |
|  | PREDICTED: transcriptional activator cubitus interruptus - [512894849_XP_004923529.1] | #N/A | 0.6211815 |
|  | PREDICTED: dedicator of cytokinesis protein 1-like - [512888695_XP_004922128.1] | #N/A | 0.6211762 |
|  | sex combs on midleg - [319803031_NP_001188365.1] | 1.05457953 | 0.6211522 |
|  | GD10512 - [195581344_XP_002080494.1] | 1.12985917 | 0.6210866 |
|  | PREDICTED: uncharacterized protein LOC101736300, partial - [512937112_XP_004933687.1] | #N/A | 0.6210589 |
|  | PREDICTED: THAP domain-containing protein 4-like - [512937820_XP_004933858.1] | 1.11079333 | 0.6210376 |
|  | PREDICTED: LOW QUALITY PROTEIN: enhancer of mRNA-decapping protein 4-like - [512919240_XP_004929326.1] | 1.1417081 | 0.6210356 |
|  | PREDICTED: trafficking protein particle complex subunit 5-like - [512924895_XP_004930705.1] | 1.19545726 | 0.6208567 |
|  | PREDICTED: ATP-dependent RNA helicase Ddx1 isoform 1 - [48095590_XP_392325.1] | 1.2202439 | 0.6208132 |
|  | PREDICTED: vesicle-fusing ATPase 1 - [512930371_XP_004932051.1] | 1.14774678 | 0.6207144 |
|  | prohibitin protein WPH - [114053221_NP_001040289.1] | 1.1151479 | 0.6206911 |
|  | PREDICTED: protein diaphanous-like - [512925238_XP_004930790.1] | #N/A | 0.6204247 |
|  | PREDICTED: conserved oligomeric Golgi complex subunit 1-like - [512903511_XP_004925637.1] | 1.06867604 | 0.6203688 |
|  | moleskin - [17864392_NP_524780.1] | 1.19326515 | 0.6203673 |
|  | PREDICTED: UPF0587 protein GA18326-like - [512895939_XP_004923793.1] | 1.19073577 | 0.6202516 |
|  | PREDICTED: DNA-directed RNA polymerase III subunit RPC1-like - [512904699_XP_004925761.1] | #N/A | 0.6201106 |
|  | glutathione S-transferase sigma 1 - [112983028_NP_001037077.1] | 1.09016127 | 0.619898 |
|  | glycine--tRNA ligase - [114053191_NP_001040293.1] | 1.20603916 | 0.6197708 |
|  | PREDICTED: membrane-associated progesterone receptor component 1-like, partial - [512939785_XP_004934326.1] | #N/A | 0.6196354 |
|  | predicted protein - [156363719_XP_001626188.1] | 1.16205939 | 0.6194224 |
|  | PREDICTED: cyclin-dependent kinase 9-like - [72136390_XP_798269.1] | 1.03937202 | 0.6194102 |
|  | PREDICTED: speckle targeted PIP5K1A-regulated poly(A) polymerase-like isoform X1 - [512915301_XP_004928356.1] | 1.19685637 | 0.6193376 |
|  | PREDICTED: adenylyl cyclase-associated protein 1-like isoform X1 - [512922470_XP_004930126.1] | 1.13954497 | 0.6192342 |
|  | PREDICTED: dynein light chain 2, cytoplasmic-like isoform X2 - [512917957_XP_004929013.1] | 1.15571155 | 0.6192005 |
|  | PREDICTED: 60 kDa SS-A/Ro ribonucleoprotein-like - [512916324_XP_004928613.1] | 1.14493509 | 0.61903 |
|  | LRP16 protein - [114051600_NP_001040417.1] | 1.1847562 | 0.6189644 |
|  | PREDICTED: inositol hexakisphosphate and diphosphoinositol-pentakisphosphate kinase-like - [345492232_XP_001600241.2] | #N/A | 0.6189375 |
|  | PREDICTED: 26S proteasome non-ATPase regulatory subunit 3-like isoform X2 - [524902594_XP_005107539.1] | 1.1996421 | 0.6188685 |
|  | GI21325 - [195124865_XP_002006904.1] | 1.20610886 | 0.6188449 |
|  | AGAP006958-PA - [118778689_XP_308800.3] | 1.20985465 | 0.6186979 |
|  | PREDICTED: similar to eukaryotic translation initiation factor 4 gamma, 2 - [189236794_XP_969772.2] | 1.20563996 | 0.6186353 |
|  | PREDICTED: kinesin light chain-like isoform X3 - [512914417_XP_004928137.1] | #N/A | 0.618196 |
|  | PREDICTED: chromobox protein homolog 5-like - [512928095_XP_004931498.1] | 1.15048083 | 0.6181939 |
|  | chromobox-like protein 5 - [114052857_NP_001040539.1] | 1.17565979 | 0.6180602 |
|  | DNA replication licensing factor Mcm5-B - [339239301_XP_003381205.1] | #N/A | 0.6180548 |
|  | PREDICTED: putative proteasome inhibitor-like isoform X1 - [512922064_XP_004930028.1] | 1.14117301 | 0.617973 |
|  | GH24832 - [195048716_XP_001992582.1] | 1.1915523 | 0.6179498 |
|  | PREDICTED: glycerol-3-phosphate acyltransferase 1, mitochondrial-like, partial - [512934516_XP_004933052.1] | 1.22265865 | 0.6179179 |
|  | GL19290 - [195147348_XP_002014642.1] | 1.1554292 | 0.6177832 |
|  | PREDICTED: BRCA1-associated protein-like - [512891792_XP_004922770.1] | #N/A | 0.617755 |
|  | PREDICTED: proteasome-associated protein ECM29 homolog - [512910450_XP_004927174.1] | #N/A | 0.6177157 |
|  | GK10852 - [195446401_XP_002070763.1] | 1.12005983 | 0.6176354 |
|  | PREDICTED: uncharacterized protein LOC101740448 - [512899710_XP_004924713.1] | #N/A | 0.6174584 |
|  | PREDICTED: hypothetical protein LOC100679659 isoform 1 - [345481827_XP_003424464.1] | 0.83408349 | 0.6173442 |
|  | DNA supercoiling factor precursor - [112983659_NP_001037337.1] | 1.11094945 | 0.6173407 |
|  | GJ15201 - [195400905_XP_002059056.1] | 1.146549 | 0.6172676 |
|  | PREDICTED: cAMP-dependent protein kinase catalytic subunit alpha-like isoform 2 - [391340846_XP_003744746.1] | 1.27777288 | 0.6171348 |
|  | hypothetical protein TRIADDRAFT_63350 - [196008677_XP_002114204.1] | 1.16009441 | 0.6171302 |
|  | PREDICTED: guanine nucleotide-binding protein-like 1-like - [512889744_XP_004922361.1] | 1.14483745 | 0.6169783 |
|  | PREDICTED: AP-3 complex subunit mu-1-like - [340727932_XP_003402287.1] | 1.1249789 | 0.6169066 |
|  | PREDICTED: protein Smaug-like - [512905447_XP_004925946.1] | 1.15937754 | 0.6167256 |
|  | snap-scaffold11498_size2399-abinit-gene-0.0-mRNA-1 | 1.14719182 | 0.6164082 |
|  | PREDICTED: apoptosis inhibitor 5-like - [328780766_XP_624077.3] | 1.28693915 | 0.6162722 |
|  | PREDICTED: peroxisomal acyl-coenzyme A oxidase 3-like - [512924002_XP_004930491.1] | 1.16168363 | 0.6161939 |
|  | PREDICTED: cyclin-Y-like isoform X1 - [498983947_XP_004530244.1] | 1.24276095 | 0.6160917 |
|  | PREDICTED: LOW QUALITY PROTEIN: enhancer of mRNA-decapping protein 4-like - [512919240_XP_004929326.1] | 1.3112955 | 0.6160222 |
|  | PREDICTED: histidyl-tRNA synthetase, cytoplasmic-like isoform 1 - [350415904_XP_003490785.1] | 1.14531051 | 0.6160139 |
|  | PREDICTED: golgi-specific brefeldin A-resistance guanine nucleotide exchange factor 1-like - [512896744_XP_004923983.1] | 1.16316703 | 0.6159882 |
|  | eukaryotic translation initiation factor 3 subunit J - [112983090_NP_001037664.1] | 1.14809428 | 0.6159828 |
|  | proteasome 25 kDa subunit - [114052504_NP_001040344.1] | 1.20010738 | 0.6158864 |
|  | programmed cell death protein 5-like protein - [148298849_NP_001091796.1] | 1.1228913 | 0.6158474 |
|  | coatomer gamma subunit - [256088224_XP_002580249.1] | 1.1693675 | 0.6157538 |
|  | PREDICTED: gem-associated protein 5-like - [512914293_XP_004928106.1] | 1.12383048 | 0.6153662 |
|  | PREDICTED: protein zwilch-like - [512894900_XP_004923542.1] | 1.08163344 | 0.6152461 |
|  | ribosomal protein S6 kinase, 90 kDa - [321400071_NP_001189457.1] | 1.24660555 | 0.6152155 |
|  | PREDICTED: ubiquitin carboxyl-terminal hydrolase 7-like - [499004347_XP_004535310.1] | 1.15443928 | 0.6151848 |
|  | PREDICTED: isocitrate dehydrogenase [NAD] subunit beta, mitochondrial-like - [512910789_XP_004927257.1] | 1.17468401 | 0.6150501 |
|  | PREDICTED: coronin-7-like - [512896128_XP_004923836.1] | 1.18978794 | 0.6150483 |
|  | CRE-PKC-2 protein - [308489019_XP_003106703.1] | #N/A | 0.6150246 |
|  | PREDICTED: TBC1 domain family member 13-like - [512910498_XP_004927187.1] | 1.22167588 | 0.6149649 |
|  | PREDICTED: probable ubiquitin carboxyl-terminal hydrolase FAF-like - [512899674_XP_004924704.1] | 1.11413331 | 0.6148375 |
|  | PREDICTED: carbonyl reductase [NADPH] 1-like - [512926045_XP_004930987.1] | 1.15801434 | 0.6148102 |
|  | PREDICTED: protein FAM192A-like - [512911971_XP_004927542.1] | 1.07663542 | 0.6147888 |
|  | PREDICTED: ADP-ribosylation factor-like protein 2-like isoform X1 - [512903773_XP_004925700.1] | 0.93385066 | 0.6147574 |
|  | GK25787 - [195447902_XP_002071421.1] | 1.15555083 | 0.6147311 |
|  | PREDICTED: ras-related protein Rab-14-like isoform X1 - [512919109_XP_004929294.1] | 1.22874769 | 0.6147147 |
|  | fructose-1,6-bisphosphatase - [114051287_NP_001040381.1] | 1.19492565 | 0.614562 |
|  | PREDICTED: dynamin-1-like protein-like isoform X1 - [498987985_XP_004531240.1] | 1.11470725 | 0.6142851 |
|  | PREDICTED: mannose-6-phosphate isomerase-like - [512924300_XP_004930563.1] | 1.24992538 | 0.6140984 |
|  | PREDICTED: probable actin-related protein 2/3 complex subunit 2-like - [512928936_XP_004931702.1] | 1.13086642 | 0.6139178 |
|  | PREDICTED: LOW QUALITY PROTEIN: transcription initiation factor TFIID subunit 4-like - [512900696_XP_004924953.1] | 1.2171936 | 0.613894 |
|  | PREDICTED: eukaryotic translation elongation factor 1 epsilon-1-like isoform X1 - [512889832_XP_004922383.1] | 1.13556789 | 0.6138147 |
|  | PREDICTED: similar to carbohydrate kinase-like - [189241031_XP_971652.2] | 1.27642408 | 0.6137995 |
|  | PREDICTED: cytoskeleton-associated protein 5-like - [512910989_XP_004927306.1] | 1.12043443 | 0.6136332 |
|  | PREDICTED: nuclear pore complex protein Nup160 homolog - [512928719_XP_004931647.1] | 1.18366439 | 0.6136331 |
|  | dynein heavy chain, cytoplasmic - [339234557_XP_003378833.1] | 1.20746991 | 0.6135634 |
|  | PREDICTED: amine sulfotransferase-like - [512934587_XP_004933069.1] | 1.13584863 | 0.6134519 |
|  | hypothetical protein AaeL_AAEL003516 - [157136831_XP_001656929.1] | #N/A | 0.6134456 |
|  | PREDICTED: similar to selenophosphate synthetase - [91085589_XP_968685.1] | 1.10790718 | 0.6133155 |
|  | PREDICTED: probable Dol-P-Man:Man(7)GlcNAc(2)-PP-Dol alpha-1,6-mannosyltransferase-like - [512886951_XP_004921841.1] | #N/A | 0.6131132 |
|  | PREDICTED: SET-binding protein-like - [512915111_XP_004928309.1] | #N/A | 0.6130978 |
|  | CRE-APM-1 protein - [308500167_XP_003112269.1] | 1.13463629 | 0.6130129 |
|  | PREDICTED: protein ETHE1, mitochondrial-like - [512915215_XP_004928335.1] | #N/A | 0.6128284 |
|  | PREDICTED: trifunctional purine biosynthetic protein adenosine-3-like - [512901569_XP_004925167.1] | 1.12445523 | 0.6125372 |
|  | CLIP-associating protein - [170070561_XP_001869623.1] | 1.14179852 | 0.6123838 |
|  | tetraspanin E - [114050851_NP_001040157.1] | 1.43919583 | 0.6122486 |
|  | PREDICTED: sterol regulatory element-binding protein cleavage-activating protein-like - [512935122_XP_004933197.1] | 0.6121711 |  |
|  | actin - [157107715_XP_001649905.1] | #N/A | 0.6120214 |
|  | Mo-molybdopterin cofactor sulfurase - [114052577_NP_001040259.1] | 1.08783258 | 0.6117262 |
|  | PREDICTED: ubiquitin conjugation factor E4 B-like - [512928944_XP_004931704.1] | 1.11005224 | 0.6117046 |
|  | BCP inhibitor precursor - [112983070_NP_001037057.1] | 1.05565323 | 0.6114569 |
|  | PREDICTED: zinc finger BED domain-containing protein 1-like - [328698733_XP_003240718.1] | #N/A | 0.6113251 |
|  | YAP65-like protein - [182510216_NP_001116819.1] | 1.16896369 | 0.6110345 |
|  | PREDICTED: papilin-like isoform X6 - [498985734_XP_004530680.1] | #N/A | 0.6109524 |
|  | PREDICTED: ferrochelatase, mitochondrial-like - [350412282_XP_003489596.1] | 1.20586188 | 0.6107872 |
|  | 6-phosphofructokinase - [170045475_XP_001850333.1] | #N/A | 0.6107811 |
|  | PREDICTED: twitchin-like - [383852204_XP_003701618.1] | #N/A | 0.6107662 |
|  | PREDICTED: similar to high-affinity octopamine transporter protein - [91087161_XP_975356.1] | #N/A | 0.6107523 |
|  | PREDICTED: protein purity of essence-like - [512917049_XP_004928791.1] | #N/A | 0.6106522 |
|  | PREDICTED: acetyl-CoA acetyltransferase B, mitochondrial-like - [156544532_XP_001607694.1] | #N/A | 0.6104961 |
|  | PREDICTED: serine/threonine-protein phosphatase PP2A 65 kDa regulatory subunit-like isoform X1 - [498940697_XP_004521445.1] | 1.16355614 | 0.6103842 |
|  | PREDICTED: LOW QUALITY PROTEIN: transcription initiation factor TFIID subunit 4-like - [512900696_XP_004924953.1] | #N/A | 0.6103104 |
|  | PREDICTED: TBC1 domain family member 22B-like - [512917861_XP_004928991.1] | #N/A | 0.6101503 |
|  | saposin-related precursor - [112984026_NP_001036831.1] | 1.09938546 | 0.6096708 |
|  | PREDICTED: uncharacterized protein LOC101739056, partial - [512885082_XP_004921536.1] | #N/A | 0.6096477 |
|  | PREDICTED: calcyclin-binding protein-like - [512929353_XP_004931799.1] | 1.20691306 | 0.6094054 |
|  | PREDICTED: phytanoyl-CoA dioxygenase domain-containing protein 1-like - [512934973_XP_004933161.1] | 1.08351409 | 0.609319 |
|  | PREDICTED: phytanoyl-CoA dioxygenase domain-containing protein 1-like - [512934973_XP_004933161.1] | 1.04937859 | 0.609319 |
|  | signal recognition particle 19 kDa protein - [114051175_NP_001040389.1] | 1.12632691 | 0.6092894 |
|  | PREDICTED: ubiquitin carboxyl-terminal hydrolase 5-like - [524908048_XP_005109132.1] | 1.14690232 | 0.6090643 |
|  | PREDICTED: asparagiNyl tRNA Synthetase family member (nrs-2)-like - [291225664_XP_002732808.1] | 1.07489728 | 0.6089519 |
|  | Rootletin (Ciliary rootlet coiled-coil protein) - [256074955_XP_002573787.1] | 1.18156437 | 0.608757 |
|  | PREDICTED: similar to DNA replication licensing factor MCM4 - [189238875_XP_973671.2] | 1.15142044 | 0.6087016 |
|  | PREDICTED: multidrug resistance-associated protein 1-like - [512907928_XP_004926564.1] | #N/A | 0.6084429 |
|  | PREDICTED: uncharacterized protein LOC101459655 - [498972150_XP_004527241.1] | #N/A | 0.6083901 |
|  | PREDICTED: ribose-phosphate pyrophosphokinase 1-like isoform 1 - [156553413_XP_001599320.1] | 1.08799063 | 0.6079846 |
|  | PREDICTED: glutamyl-tRNA(Gln) amidotransferase subunit A homolog - [156551449_XP_001604590.1] | 1.14288494 | 0.607934 |
|  | PREDICTED: COP9 signalosome complex subunit 7b-like - [512897293_XP_004924118.1] | 1.14618316 | 0.6078698 |
|  | PREDICTED: uroporphyrinogen decarboxylase-like - [512913333_XP_004927871.1] | 1.16407681 | 0.60783 |
|  | PREDICTED: inositol-3-phosphate synthase-like - [512896736_XP_004923981.1] | 1.16512314 | 0.6076127 |
|  | PREDICTED: dmX-like protein 2-like - [524879263_XP_005096309.1] | 1.23313562 | 0.6073456 |
|  | PREDICTED: mitochondrial import inner membrane translocase subunit Tim22-like - [512891637_XP_004922732.1] | 1.13979284 | 0.6072046 |
|  | PREDICTED: protein CDV3 homolog - [512935741_XP_004933348.1] | 1.12889265 | 0.6072026 |
|  | PREDICTED: enolase-like isoform 1 - [193669445_XP_001948161.1] | 1.15758466 | 0.6071129 |
|  | PREDICTED: NSFL1 cofactor p47-like - [383852776_XP_003701901.1] | 1.12166933 | 0.6070459 |
|  | PREDICTED: nucleoporin NUP188 homolog - [512932038_XP_004932447.1] | 1.16918123 | 0.6067792 |
|  | PREDICTED: tyrosine-protein phosphatase non-receptor type 23-like - [383850014_XP_003700623.1] | #N/A | 0.6067162 |
|  | cytochrome P450 9a20 - [134254438_NP_001077079.1] | 1.08108496 | 0.6066383 |
|  | PREDICTED: uncharacterized protein LOC101746598 - [512936341_XP_004933495.1] | #N/A | 0.606629 |
|  | PREDICTED: LOW QUALITY PROTEIN: putative ATPase N2B-like - [512930204_XP_004932008.1] | 1.26199615 | 0.6065878 |
|  | PREDICTED: cAMP-dependent protein kinase type II regulatory subunit-like isoform 6 - [345492835_XP_003426938.1] | 1.18379421 | 0.6062659 |
|  | glycerol kinase-like protein - [290565764_NP_001166873.1] | 0.6061814 |  |
|  | PREDICTED: mitochondrial glutamate carrier 1-like - [512927479_XP_004931345.1] | 1.16589707 | 0.6061479 |
|  | PREDICTED: similar to sphingosine phosphate lyase isoform 1 - [91076782_XP_967792.1] | 1.12826643 | 0.6058837 |
|  | Protein CTPS-1 - [25148299_NP_507243.2] | 1.09360071 | 0.6058588 |
|  | PREDICTED: LOW QUALITY PROTEIN: AP-1 complex subunit beta-1-like - [512917216_XP_004928833.1] | 1.13413184 | 0.6058241 |
|  | PREDICTED: condensin complex subunit 2-like - [512928523_XP_004931599.1] | 1.12254606 | 0.6058184 |
|  | PREDICTED: LOW QUALITY PROTEIN: talin-1-like - [383857561_XP_003704273.1] | 1.24424549 | 0.605673 |
|  | coatomer protein complex subunit alpha - [289629216_NP_001166192.1] | 1.1852682 | 0.6055567 |
|  | PREDICTED: LOW QUALITY PROTEIN: nipped-B-like protein A-like - [512900427_XP_004924889.1] | #N/A | 0.6051262 |
|  | replication factor C (activator 1) 5 - [114050971_NP_001040148.1] | 1.1536211 | 0.6050804 |
|  | PREDICTED: poly(A) polymerase gamma-like isoform X1 - [512893293_XP_004923145.1] | 1.2623554 | 0.6049768 |
|  | nonclathrin coat protein zeta 1-COP - [114051996_NP_001040202.1] | 1.30624412 | 0.6049473 |
|  | PREDICTED: charged multivesicular body protein 2b-like isoform X1 - [512930562_XP_004932090.1] | 0.99363292 | 0.6049234 |
|  | PREDICTED: E3 ubiquitin-protein ligase BRE1-like isoform X1 - [512913355_XP_004927876.1] | 1.11387872 | 0.6048689 |
|  | PREDICTED: uncharacterized protein LOC101737178 - [512938120_XP_004933929.1] | 1.07388266 | 0.6047015 |
|  | PREDICTED: BRISC and BRCA1-A complex member 1-like isoform X1 - [512926690_XP_004931149.1] | 1.10558916 | 0.6046494 |
|  | PREDICTED: nuclear distribution protein nudE-like 1-B-like - [512909935_XP_004927043.1] | 1.10344008 | 0.604605 |
|  | signal sequence receptor beta subunit precursor - [114052941_NP_001040332.1] | 1.15836107 | 0.6045332 |
|  | PREDICTED: spermine oxidase-like - [512889701_XP_004922349.1] | 1.13067427 | 0.6042918 |
|  | FAM40A protein, partial - [312080732_XP_003142726.1] | 1.1889707 | 0.6039561 |
|  | PREDICTED: LOW QUALITY PROTEIN: probable tRNA (guanine(26)-N(2))-dimethyltransferase-like - [512901109_XP_004925054.1] | 1.20186072 | 0.6038419 |
|  | NADPH-specific isocitrate dehydrogenase - [151301209_NP_001093090.1] | 1.12699861 | 0.6037014 |
|  | GA10892 - [125776883_XP_001359425.1] | 1.20940364 | 0.6035143 |
|  | PREDICTED: inositol-3-phosphate synthase-like - [512896736_XP_004923981.1] | 1.14560643 | 0.6033473 |
|  | PREDICTED: glutathione synthetase-like isoform X1 - [512912406_XP_004927647.1] | 1.13294756 | 0.6033226 |
|  | PREDICTED: quinone oxidoreductase-like - [512887563_XP_004921945.1] | 1.12735919 | 0.6033189 |
|  | PREDICTED: sister chromatid cohesion protein DCC1-like - [512902317_XP_004925346.1] | 1.17727548 | 0.6032646 |
|  | GK22654 - [195449447_XP_002072079.1] | 1.1474604 | 0.6032192 |
|  | hypothetical protein AaeL_AAEL004332 - [157105573_XP_001648928.1] | #N/A | 0.603207 |
|  | GK25150 - [195447878_XP_002071411.1] | #N/A | 0.6030955 |
|  | PREDICTED: hypothetical protein LOC100647311 - [340722685_XP_003399734.1] | #N/A | 0.60308 |
|  | PREDICTED: exportin-7-like - [391331330_XP_003740102.1] | 1.10484199 | 0.6029447 |
|  | AGAP010313-PA, partial - [158300117_XP_320116.6] | #N/A | 0.6028827 |
|  | cytochrome P450 9a20 - [134254438_NP_001077079.1] | 0.9847455 | 0.6027782 |
|  | ribonuclease L inhibitor homolog - [112982681_NP_001036911.1] | 1.17075789 | 0.6026435 |
|  | PREDICTED: tubulin-specific chaperone A-like isoform X1 - [512901175_XP_004925071.1] | 1.06919163 | 0.6024813 |
|  | PREDICTED: probable isoaspartyl peptidase/L-asparaginase GA20639-like isoform X2 - [512932225_XP_004932493.1] | 1.15634429 | 0.6024354 |
|  | mitochondrial aldehyde dehydrogenase - [114052408_NP_001040475.1] | 1.11193613 | 0.6022105 |
|  | PREDICTED: FAD-linked sulfhydryl oxidase ALR-like - [512937050_XP_004933670.1] | #N/A | 0.6021541 |
|  | aspartate aminotransferase - [114053127_NP_001040337.1] | 1.15461032 | 0.6020507 |
|  | PREDICTED: LOW QUALITY PROTEIN: nipped-B-like protein-like - [340717550_XP_003397244.1] | 1.23809509 | 0.6018027 |
|  | snap-scaffold495_size381097-abinit-gene-0.3-mRNA-1 | #N/A | 0.6017373 |
|  | PREDICTED: long-chain fatty acid transport protein 4-like - [512918879_XP_004929240.1] | 1.1202442 | 0.601537 |
|  | preimplantation protein - [114050741_NP_001040402.1] | 1.18601285 | 0.6013871 |
|  | PREDICTED: multiple coagulation factor deficiency protein 2 homolog isoform X1 - [512889107_XP_004922198.1] | 1.19169815 | 0.6013814 |
|  | T-complex protein 1 subunit beta - [339241349_XP_003376600.1] | 1.17873626 | 0.601257 |
|  | PREDICTED: structural maintenance of chromosomes protein 4-like isoform X1 - [498960983_XP_004524728.1] | 1.11978806 | 0.60121 |
|  | PREDICTED: retinol dehydrogenase 13-like isoform X1 - [512892632_XP_004922979.1] | 0.94493047 | 0.6011555 |
|  | PREDICTED: ankyrin repeat and FYVE domain-containing protein 1-like - [328713406_XP_001945772.2] | 1.10395733 | 0.6008963 |
|  | PREDICTED: cyclin-G-associated kinase-like - [512903567_XP_004925651.1] | #N/A | 0.6007337 |
|  | PREDICTED: pleckstrin homology domain-containing family F member 2-like - [512889865_XP_004922392.1] | #N/A | 0.6007067 |
|  | PREDICTED: uncharacterized protein LOC101742439 - [512895532_XP_004923694.1] | #N/A | 0.6006512 |
|  | PREDICTED: protein SHQ1 homolog - [512888376_XP_004922077.1] | 1.11229339 | 0.6005788 |
|  | snap-scaffold3341_size32710-abinit-gene-0.3-mRNA-1 | 0.6004973 |  |
|  | PREDICTED: THUMP domain-containing protein 1 homolog - [512929897_XP_004931936.1] | 1.10798178 | 0.6004545 |
|  | PREDICTED: dehydrogenase/reductase SDR family protein 7-like - [512900213_XP_004924837.1] | 1.19339943 | 0.6003738 |
|  | PREDICTED: LOW QUALITY PROTEIN: bifunctional protein NCOAT-like - [512888763_XP_004922140.1] | 1.22860973 | 0.6003516 |
|  | PREDICTED: similar to AGAP007253-PA - [189238240_XP_972964.2] | #N/A | 0.6003213 |
|  | PREDICTED: phosphopantothenoylcysteine decarboxylase-like - [512895346_XP_004923650.1] | #N/A | 0.6002994 |
|  | PREDICTED: coatomer subunit beta-like isoform X1 - [524881016_XP_005097164.1] | 1.15141797 | 0.6002519 |
|  | PREDICTED: uncharacterized protein LOC101745488 isoform X1 - [512921571_XP_004929909.1] | #N/A | 0.6001428 |
|  | PREDICTED: nicalin-like - [512936212_XP_004933463.1] | 1.24999239 | 0.6000827 |
|  | PREDICTED: putative N-acetylglucosamine-6-phosphate deacetylase-like - [512913198_XP_004927838.1] | 1.05885653 | 0.5999978 |
|  | AP-2 complex subunit alpha-2 - [339245073_XP_003378462.1] | 1.18306838 | 0.5998968 |
|  | PREDICTED: uncharacterized protein LOC101745476 isoform X1 - [512909903_XP_004927035.1] | 1.01484648 | 0.5998843 |
|  | PREDICTED: LOW QUALITY PROTEIN: protein FAM76A-like - [512897014_XP_004924049.1] | 1.28384176 | 0.5997617 |
|  | PREDICTED: transcription elongation factor B polypeptide 2-like isoform X1 - [512901967_XP_004925259.1] | 1.10591165 | 0.5997525 |
|  | PREDICTED: uncharacterized protein LOC101738207 - [512903384_XP_004925606.1] | #N/A | 0.5996784 |
|  | CCT-5 protein - [312079735_XP_003142302.1] | 1.15144096 | 0.599646 |
|  | PREDICTED: guanine nucleotide exchange factor MSS4 homolog - [512911967_XP_004927541.1] | 1.12944214 | 0.5995084 |
|  | PREDICTED: putative leucine-rich repeat-containing protein DDB_G0290503-like - [512920314_XP_004929601.1] | 1.18723973 | 0.5994527 |
|  | PREDICTED: activating signal cointegrator 1 complex subunit 3-like - [390354247_XP_003728286.1] | 1.23154823 | 0.5993198 |
|  | PREDICTED: sideroflexin-2-like - [512924827_XP_004930688.1] | 1.1666665 | 0.5992753 |
|  | PREDICTED: 5'-3' exoribonuclease 2-like - [340375702_XP_003386373.1] | 1.16256159 | 0.599256 |
|  | PREDICTED: venom carboxylesterase-6-like - [512901695_XP_004925199.1] | 1.1117178 | 0.5992476 |
|  | PREDICTED: oxysterol-binding protein 1-like isoform 2 - [345494533_XP_003427315.1] | 1.12622913 | 0.5991956 |
|  | PREDICTED: ATP-citrate synthase-like, partial - [512938247_XP_004933961.1] | 1.13766999 | 0.5991332 |
|  | PREDICTED: uncharacterized protein LOC101742884 - [512905596_XP_004925984.1] | #N/A | 0.599129 |
|  | PREDICTED: SHC SH2 domain-binding protein 1 homolog B-like - [345479291_XP_001605544.2] | #N/A | 0.5989438 |
|  | ARP1 actin-related protein 1-like protein A - [114053021_NP_001040336.1] | 1.05354925 | 0.5988525 |
|  | PREDICTED: RAC serine/threonine-protein kinase - [512915270_XP_004928348.1] | #N/A | 0.5987093 |
|  | elongation factor 1-alpha - [339238085_XP_003380597.1] | 1.15207338 | 0.5987063 |
|  | PREDICTED: coiled-coil domain-containing protein 6-like isoform X1 - [512929714_XP_004931891.1] | 1.15602328 | 0.5986605 |
|  | PREDICTED: protein FAM160A1-like, partial - [512938309_XP_004933975.1] | #N/A | 0.5985392 |
|  | GI19719 - [195119065_XP_002004052.1] | 1.1162026 | 0.5984206 |
|  | PREDICTED: uncharacterized protein C45G9.7-like - [512922147_XP_004930049.1] | #N/A | 0.5983719 |
|  | PREDICTED: stromal membrane-associated protein 1-like - [512902305_XP_004925343.1] | 1.24188308 | 0.5983214 |
|  | PREDICTED: ubiquitin carboxyl-terminal hydrolase 14-like - [512910665_XP_004927226.1] | 1.09651275 | 0.5981867 |
|  | PREDICTED: uncharacterized protein LOC101744209 - [512891025_XP_004922613.1] | 1.12063812 | 0.5981103 |
|  | PREDICTED: protein purity of essence-like - [512917049_XP_004928791.1] | 1.18474009 | 0.5980638 |
|  | PREDICTED: tyrosine-protein kinase Fps85D-like - [512924253_XP_004930552.1] | #N/A | 0.5980487 |
|  | PREDICTED: reticulocyte-binding protein PFD0110w-like - [512928653_XP_004931631.1] | 1.14907993 | 0.59801 |
|  | PREDICTED: very-long-chain (3R)-3-hydroxyacyl-[acyl-carrier protein] dehydratase-like - [512920793_XP_004929721.1] | 1.11732666 | 0.5979138 |
|  | chaperonin containing t-complex protein 1 zeta subunit tcpz - [256071267_XP_002571962.1] | 1.12205891 | 0.5979056 |
|  | PREDICTED: exocyst complex component 6-like - [512932515_XP_004932564.1] | #N/A | 0.59788 |
|  | Histone H3c - [170053486_XP_001862696.1] | 1.24702731 | 0.5978172 |
|  | PREDICTED: uncharacterized protein LOC101743491 - [512895839_XP_004923768.1] | 1.12355594 | 0.5974472 |
|  | PREDICTED: septin-1-like isoform 2 - [345484753_XP_003425116.1] | 1.10898253 | 0.5972115 |
|  | PREDICTED: uncharacterized protein LOC101745942 - [512891086_XP_004922624.1] | 0.90863831 | 0.5971602 |
|  | PREDICTED: U4/U6.U5 tri-snRNP-associated protein 2-like - [512916383_XP_004928627.1] | #N/A | 0.597102 |
|  | snap-scaffold7256_size37059-abinit-gene-0.6-mRNA-1 | 1.25308567 | 0.5970736 |
|  | PREDICTED: proteasome subunit beta type-4-like - [512935468_XP_004933282.1] | 1.18673026 | 0.5968129 |
|  | DDRGK domain-containing protein 1 precursor - [225703086_NP_001139535.1] | 1.20669372 | 0.596637 |
|  | PREDICTED: cytosolic non-specific dipeptidase-like - [512915932_XP_004928508.1] | 1.15462528 | 0.5966289 |
|  | PREDICTED: LOW QUALITY PROTEIN: myotubularin-related protein 5-like - [512891523_XP_004922705.1] | 1.23385993 | 0.5966207 |
|  | coatomer protein complex subunit alpha - [289629216_NP_001166192.1] | 1.25719333 | 0.5965717 |
|  | vacuolar ATP synthase subunit F - [114052036_NP_001040448.1] | 1.27790013 | 0.5961871 |
|  | PREDICTED: N-myristoyltransferase 2-like - [291243083_XP_002741431.1] | 1.20704925 | 0.5961022 |
|  | PREDICTED: kinesin light chain-like isoform X1 - [512914409_XP_004928135.1] | 1.14904739 | 0.5960064 |
|  | PREDICTED: teneurin-m-like - [512913867_XP_004927998.1] | 0.96462757 | 0.5959764 |
|  | eukaryotic translation initiation factor 3 subunit K - [114053117_NP_001040534.1] | 1.16442593 | 0.5957996 |
|  | FK506-binding protein - [114051243_NP_001040382.1] | 1.00190133 | 0.5957557 |
|  | PREDICTED: neuroligin-4, X-linked-like - [512898742_XP_004924471.1] | 1.03664606 | 0.5955903 |
|  | PREDICTED: adenylate kinase isoenzyme 1-like isoform X2 - [512918578_XP_004929168.1] | 1.35544705 | 0.5955049 |
|  | PREDICTED: uncharacterized protein LOC100870087 - [380011487_XP_003689834.1] | 1.18198009 | 0.5953119 |
|  | PREDICTED: putative deoxyribonuclease TATDN1-like - [512936227_XP_004933466.1] | #N/A | 0.5951523 |
|  | PREDICTED: eukaryotic translation initiation factor 2-alpha kinase-like - [498927134_XP_004518064.1] | 1.2863669 | 0.595124 |
|  | mitochondrial thioredoxin 2 - [114053263_NP_001040283.1] | 1.15081597 | 0.5950916 |
|  | PREDICTED: helicase SKI2W-like - [512887417_XP_004921921.1] | 1.16724604 | 0.5949523 |
|  | PREDICTED: YEATS domain-containing protein 2-like - [512900908_XP_004925005.1] | #N/A | 0.5949331 |
|  | PREDICTED: leucine-rich repeat-containing protein 47-like - [512917837_XP_004928985.1] | 1.22743372 | 0.594833 |
|  | NADH dehydrogenase 1 alpha subcomplex subunit 5 - [153792038_NP_001093287.1] | #N/A | 0.5948145 |
|  | PREDICTED: MGC80816 protein-like - [291236799_XP_002738314.1] | 1.0838474 | 0.5947178 |
|  | PREDICTED: structural maintenance of chromosomes protein 3-like - [512886230_XP_004921724.1] | 1.18277816 | 0.5946733 |
|  | PREDICTED: nardilysin-like - [380020506_XP_003694124.1] | #N/A | 0.5946203 |
|  | GH11577 - [195035417_XP_001989174.1] | 1.18979196 | 0.5946102 |
|  | PREDICTED: probable phosphorylase b kinase regulatory subunit beta-like - [512926363_XP_004931067.1] | #N/A | 0.5943617 |
|  | uncharacterized protein LOC778477 - [148298784_NP_001091772.1] | #N/A | 0.5942571 |
|  | PREDICTED: cytoplasmic aconitate hydratase-like - [512888904_XP_004922163.1] | 1.14857972 | 0.5940087 |
|  | PREDICTED: uncharacterized protein LOC101736046 - [512907107_XP_004926361.1] | #N/A | 0.5940033 |
|  | PREDICTED: facilitated trehalose transporter Tret1-like - [512933881_XP_004932901.1] | 1.1004638 | 0.593857 |
|  | PREDICTED: intracellular protein transport protein USO1-like - [512916825_XP_004928735.1] | 1.29103514 | 0.593762 |
|  | cytochrome P450 6AB4 - [119226184_NP_001073135.1] | 1.13972153 | 0.5936648 |
|  | adenosine deaminase related growth factor precursor - [157412314_NP_001098698.1] | 1.11604559 | 0.5936192 |
|  | PREDICTED: WD repeat-containing protein 55 homolog isoform X1 - [512919121_XP_004929297.1] | 1.16323815 | 0.5935958 |
|  | PREDICTED: echinoderm microtubule-associated protein-like 1-like isoform 3 - [328713019_XP_003244975.1] | #N/A | 0.5935201 |
|  | PREDICTED: luciferin 4-monooxygenase-like - [383854344_XP_003702681.1] | 1.14422558 | 0.593506 |
|  | valine-tRNA ligase - [256089093_XP_002580651.1] | 1.15605351 | 0.5934341 |
|  | AGAP007332-PA, partial - [158285854_XP_308497.4] | 1.1456846 | 0.5933974 |
|  | PREDICTED: succinate dehydrogenase cytochrome b560 subunit, mitochondrial-like - [512933149_XP_004932719.1] | 1.13415145 | 0.5932986 |
|  | PREDICTED: aspartate--tRNA ligase, cytoplasmic-like - [512935617_XP_004933317.1] | 1.12066859 | 0.5932979 |
|  | PREDICTED: ribonucleoside-diphosphate reductase large subunit-like - [512903164_XP_004925552.1] | 1.16568075 | 0.5932146 |
|  | snap-scaffold2751_size97069-abinit-gene-0.4-mRNA-1 | 1.17722037 | 0.5931387 |
|  | alanyl-tRNA synthetase domain-containing protein 1 - [170060836_XP_001865977.1] | #N/A | 0.5930218 |
|  | hypothetical protein CRE_21279 - [308486001_XP_003105198.1] | 1.20552341 | 0.5929607 |
|  | PREDICTED: pre-mRNA-splicing factor 38B-like - [512916560_XP_004928669.1] | 1.23980415 | 0.5928597 |
|  | AMP-activated protein kinase beta subunit - [158186774_NP_001103403.1] | 1.02668402 | 0.5928593 |
|  | PREDICTED: uncharacterized protein LOC101740611 - [512932229_XP_004932494.1] | 1.08476046 | 0.592773 |
|  | multidrug resistance related protein 1 - [170574287_XP_001892748.1] | 1.06367964 | 0.5927633 |
|  | hypothetical protein - [170577165_XP_001893907.1] | 1.17010465 | 0.5927371 |
|  | PREDICTED: hypothetical protein LOC100651932 - [340723750_XP_003400252.1] | 1.26325536 | 0.5927265 |
|  | GL21279 - [195175634_XP_002028537.1] | 1.17319394 | 0.5927057 |
|  | PREDICTED: uncharacterized protein LOC101742849 - [512923532_XP_004930375.1] | 1.02789122 | 0.592618 |
|  | PREDICTED: vacuolar protein sorting-associated protein 16 homolog - [512924269_XP_004930556.1] | #N/A | 0.5926019 |
|  | PREDICTED: ADP-ribosylation factor 2-like - [512929175_XP_004931759.1] | 1.1875983 | 0.5925665 |
|  | PREDICTED: UHRF1-binding protein 1-like - [512891519_XP_004922704.1] | #N/A | 0.5925041 |
|  | PREDICTED: ubiquitin carboxyl-terminal hydrolase isozyme L5-like isoform X1 - [512934835_XP_004933128.1] | 1.1565613 | 0.5924732 |
|  | PREDICTED: pre-mRNA-splicing factor SYF1-like - [498936004_XP_004520278.1] | 1.1478538 | 0.5921762 |
|  | snap-scaffold5902_size19925-abinit-gene-0.1-mRNA-1 | #N/A | 0.5921499 |
|  | hexokinase - [157123146_XP_001660030.1] | 1.20781624 | 0.5919681 |
|  | PREDICTED: 26S protease regulatory subunit 6B-like - [524899180_XP_005106028.1] | 1.19689884 | 0.5918943 |
|  | eukaryotic translation initiation factor 6 - [114052170_NP_001040517.1] | 1.15393837 | 0.5918298 |
|  | PREDICTED: cytosolic non-specific dipeptidase-like - [512915932_XP_004928508.1] | 1.13923142 | 0.5917933 |
|  | PREDICTED: acyl-CoA synthetase family member 3, mitochondrial-like - [512910844_XP_004927270.1] | 1.02835716 | 0.591725 |
|  | PREDICTED: COP9 signalosome complex subunit 2-like isoform 2 - [383856487_XP_003703740.1] | 1.15490982 | 0.5916917 |
|  | PREDICTED: adenine phosphoribosyltransferase-like - [512936612_XP_004933560.1] | 1.15499657 | 0.5916875 |
|  | cytochrome P450 9a20 - [134254438_NP_001077079.1] | #N/A | 0.5915773 |
|  | PREDICTED: iron-sulfur assembly protein IscA-like 2, mitochondrial-like - [512929210_XP_004931767.1] | #N/A | 0.591483 |
|  | PREDICTED: beta carbonic anhydrase 1-like - [512892712_XP_004922999.1] | 1.27713892 | 0.5914265 |
|  | PREDICTED: mediator of RNA polymerase II transcription subunit 6-like - [512899005_XP_004924537.1] | 1.15414068 | 0.5914143 |
|  | PREDICTED: dnaJ homolog subfamily C member 2-like - [512906510_XP_004926214.1] | 1.12984947 | 0.5913995 |
|  | GI16597 - [195135443_XP_002012142.1] | 1.29078968 | 0.590993 |
|  | importin-beta 3 - [256076789_XP_002574692.1] | 1.1438273 | 0.5906369 |
|  | PREDICTED: uncharacterized protein R102.4-like - [512887961_XP_004922009.1] | 1.10514136 | 0.5906237 |
|  | PREDICTED: dnaJ homolog subfamily C member 17-like - [512915039_XP_004928291.1] | #N/A | 0.5906061 |
|  | PREDICTED: alcohol dehydrogenase [NADP(+)]-like - [512891681_XP_004922743.1] | 1.18584306 | 0.5905855 |
|  | PREDICTED: exosome complex component CSL4-like - [512934499_XP_004933048.1] | 0.91200896 | 0.5905619 |
|  | PREDICTED: histone deacetylase complex subunit SAP18-like - [512886396_XP_004921749.1] | 1.16624509 | 0.5905543 |
|  | PREDICTED: hypothetical protein LOC408314 isoform 2 - [66519484_XP_623559.1] | 1.20869663 | 0.5905219 |
|  | PREDICTED: membrane-associated protein Hem-like - [328718030_XP_003246364.1] | 1.18223493 | 0.5905067 |
|  | PREDICTED: protein vav-like - [512917129_XP_004928811.1] | #N/A | 0.5903762 |
|  | PREDICTED: nuclear cap-binding protein subunit 1-like - [512920777_XP_004929717.1] | 1.13093247 | 0.5902528 |
|  | bolA-like 3 - [114051307_NP_001040378.1] | 1.2907398 | 0.5901563 |
|  | PREDICTED: zinc finger BED domain-containing protein 4-like - [328724218_XP_001947526.2] | #N/A | 0.5901179 |
|  | RAN binding protein - [114051399_NP_001040369.1] | 1.12463395 | 0.5900489 |
|  | AGAP003018-PB - [347969072_XP_003436355.1] | #N/A | 0.5900387 |
|  | PREDICTED: synaptobrevin-like - [512913297_XP_004927862.1] | 1.1071273 | 0.589992 |
|  | PREDICTED: peroxisomal multifunctional enzyme type 2-like - [512935985_XP_004933409.1] | 1.10407712 | 0.5898801 |
|  | PREDICTED: oxysterol-binding protein-related protein 9-like - [512897885_XP_004924262.1] | 1.23640378 | 0.5898164 |
|  | PREDICTED: titin-like - [512905985_XP_004926083.1] | #N/A | 0.5895959 |
|  | PREDICTED: protein SEC13 homolog - [512894099_XP_004923349.1] | 1.16861361 | 0.58958 |
|  | PREDICTED: protein suppressor of sable-like - [512931360_XP_004932283.1] | 1.19652404 | 0.589482 |
|  | GE20372 - [195492746_XP_002094123.1] | 1.13529791 | 0.5894286 |
|  | PREDICTED: tetratricopeptide repeat protein 27-like - [512923512_XP_004930370.1] | 1.2329194 | 0.589363 |
|  | PREDICTED: protein phosphatase PP2A 55 kDa regulatory subunit-like isoform 1 - [340725467_XP_003401091.1] | 1.10568403 | 0.589301 |
|  | PREDICTED: N-alpha-acetyltransferase 15, NatA auxiliary subunit-like - [345496372_XP_001603208.2] | 1.1095226 | 0.5892484 |
|  | snap-scaffold4356_size35143-abinit-gene-0.3-mRNA-1 | #N/A | 0.5891554 |
|  | endoribonuclease Dicer - [170049284_XP_001855187.1] | 1.05100615 | 0.5891108 |
|  | PREDICTED: PR domain zinc finger protein 16-like - [512929523_XP_004931842.1] | 1.20720627 | 0.5890467 |
|  | ribonucleoside-diphosphate reductase small chain - [157126966_XP_001654749.1] | 1.17828723 | 0.5888152 |
|  | PREDICTED: tudor domain-containing protein 3-like - [512912895_XP_004927766.1] | 1.16337922 | 0.5886194 |
|  | PREDICTED: LOW QUALITY PROTEIN: rho GDP-dissociation inhibitor 1-like - [512888254_XP_004922056.1] | 1.14490157 | 0.588279 |
|  | c-Jun NH2-terminal kinase - [158186758_NP_001103396.1] | #N/A | 0.5881049 |
|  | SNF4/AMP-activated protein kinase gamma subunit - [187281646_NP_001119720.1] | #N/A | 0.5880373 |
|  | DnaJ (Hsp40) homolog 5 - [112983400_NP_001036990.1] | 1.37122222 | 0.5879836 |
|  | PREDICTED: lamin-C-like - [512922266_XP_004930078.1] | 1.14505427 | 0.5879817 |
|  | glycogen phosphorylase - [339246111_XP_003374689.1] | 1.11743376 | 0.5878951 |
|  | snap-scaffold7484_size16772-abinit-gene-0.3-mRNA-1 | #N/A | 0.587826 |
|  | PREDICTED: LOW QUALITY PROTEIN: pre-mRNA-processing factor 19-like - [524891985_XP_005102524.1] | 1.18164541 | 0.5877853 |
|  | PREDICTED: nuclear pore complex protein Nup85-like - [512910568_XP_004927204.1] | 1.22298461 | 0.587697 |
|  | CRE-CARS-1 protein - [308505978_XP_003115172.1] | 1.1685179 | 0.5876463 |
|  | PREDICTED: protein scarlet-like - [512931821_XP_004932395.1] | #N/A | 0.5875624 |
|  | mitochondrial assembly regulatory factor, isoform A - [20128953_NP_572320.1] | 1.19934934 | 0.5874775 |
|  | PREDICTED: LOW QUALITY PROTEIN: exocyst complex component 5-like - [380024884_XP_003696219.1] | 1.14532132 | 0.5874174 |
|  | tRNA (guanine-N(7)-)-methyltransferase - [114051962_NP_001040444.1] | #N/A | 0.5872448 |
|  | adducin - [157128354_XP_001661416.1] | 1.1713086 | 0.5871362 |
|  | PREDICTED: porphobilinogen deaminase-like - [512893168_XP_004923114.1] | 1.09251065 | 0.5871352 |
|  | PREDICTED: LOW QUALITY PROTEIN: ubiquitin carboxyl-terminal hydrolase 32-like, partial - [512891796_XP_004922771.1] | 1.24765405 | 0.5870265 |
|  | PREDICTED: ribosome biogenesis protein BRX1 homolog - [512907455_XP_004926446.1] | 1.16043518 | 0.5865263 |
|  | PREDICTED: probable ATP-dependent RNA helicase DHX36-like - [512929535_XP_004931845.1] | #N/A | 0.5865085 |
|  | PREDICTED: cell differentiation protein RCD1 homolog - [340723059_XP_003399915.1] | 1.19413947 | 0.5864608 |
|  | PREDICTED: ubiquitin carboxyl-terminal hydrolase 16-like - [512896752_XP_004923985.1] | #N/A | 0.5864539 |
|  | PREDICTED: protein-associating with the carboxyl-terminal domain of ezrin-like - [512916307_XP_004928606.1] | #N/A | 0.5864341 |
|  | PREDICTED: nuclear pore complex protein Nup98-Nup96-like - [512889721_XP_004922354.1] | 1.19477213 | 0.5862949 |
|  | aspartate aminotransferase - [114053127_NP_001040337.1] | 1.10181112 | 0.5861973 |
|  | PREDICTED: uncharacterized protein LOC101745471 - [512907599_XP_004926483.1] | #N/A | 0.5861201 |
|  | PREDICTED: glyoxylate reductase/hydroxypyruvate reductase-like isoform X1 - [512898611_XP_004924439.1] | #N/A | 0.5859249 |
|  | squid protein homologue - [112983696_NP_001037323.1] | 1.04812504 | 0.5858606 |
|  | ecdysteroid-phosphate phosphatase - [112982782_NP_001036900.1] | 1.09402506 | 0.5855197 |
|  | PREDICTED: 3'(2'),5'-bisphosphate nucleotidase 1-like isoform X2 - [512899033_XP_004924544.1] | 1.12702239 | 0.5855033 |
|  | PREDICTED: LOW QUALITY PROTEIN: centromere-associated protein E-like - [512914297_XP_004928107.1] | 1.16775005 | 0.5854664 |
|  | PREDICTED: UPF0585 protein CG18661-like isoform X1 - [512922596_XP_004930154.1] | 1.15129841 | 0.5853267 |
|  | WD40 protein - [114052198_NP_001040226.1] | 1.09235685 | 0.5851334 |
|  | PREDICTED: protein YIPF2-like isoform X1 - [512896043_XP_004923817.1] | 1.11161315 | 0.5849548 |
|  | cak1 - [157169491_XP_001657865.1] | 1.12714458 | 0.5849174 |
|  | PREDICTED: vacuolar protein-sorting-associated protein 36-like - [512913115_XP_004927818.1] | 1.09664737 | 0.5848306 |
|  | PREDICTED: similar to orf - [189242129_XP_001810711.1] | #N/A | 0.5848207 |
|  | SNF4/AMP-activated protein kinase gamma subunit - [187281646_NP_001119720.1] | 1.08173153 | 0.5847222 |
|  | DnaJ (Hsp40) homolog 1 - [255652879_NP_001157380.1] | 1.20376522 | 0.5845176 |
|  | PREDICTED: fragile X mental retardation syndrome-related protein 1 isoform 2 - [328783510_XP_394058.4] | 1.14425479 | 0.5844844 |
|  | vacuolar protein sorting-associated protein - [170049890_XP_001858599.1] | 1.2029715 | 0.5844171 |
|  | hypothetical protein Tsp_10268 - [339254572_XP_003372509.1] | 1.15944802 | 0.5843443 |
|  | PREDICTED: LOW QUALITY PROTEIN: N-alpha-acetyltransferase 15, NatA auxiliary subunit-like - [512891181_XP_004922640.1] | 1.15569032 | 0.5842179 |
|  | zinc finger protein - [112982721_NP_001037117.1] | 1.18957969 | 0.584123 |
|  | PREDICTED: ATP-dependent RNA helicase Ddx1-like - [512918187_XP_004929071.1] | 1.22888364 | 0.5840625 |
|  | PREDICTED: general vesicular transport factor p115-like - [156552740_XP_001599627.1] | 1.14552604 | 0.5840428 |
|  | PREDICTED: probable NADH dehydrogenase [ubiquinone] 1 alpha subcomplex subunit 12-like - [512900831_XP_004924987.1] | 1.11693652 | 0.5840383 |
|  | PREDICTED: uncharacterized protein LOC101742014 - [512905572_XP_004925978.1] | 1.11830834 | 0.5840271 |
|  | PREDICTED: rho GTPase-activating protein 17-like - [350407047_XP_003487967.1] | 1.18566002 | 0.5838141 |
|  | PREDICTED: glutamate-rich WD repeat-containing protein 1-like - [512934097_XP_004932951.1] | 1.16372471 | 0.583649 |
|  | PREDICTED: xanthine dehydrogenase-like - [512905502_XP_004925960.1] | #N/A | 0.5835506 |
|  | elongator complex protein 2 - [19921984_NP_610600.1] | 1.09951698 | 0.5831482 |
|  | tyrosyl-tRNA synthetase, cytoplasmic - [339234385_XP_003382309.1] | 1.1530385 | 0.5831199 |
|  | PREDICTED: THAP domain-containing protein 1 A-like isoform X1 - [512924903_XP_004930707.1] | 1.23655242 | 0.5830097 |
|  | prefoldin subunit 3 - [114052186_NP_001040458.1] | 1.13390374 | 0.5829577 |
|  | Fanconi anemia, complementation group D2 - [350536779_NP_001233147.1] | 1.16632166 | 0.5828758 |
|  | PREDICTED: dual 3',5'-cyclic-AMP and -GMP phosphodiesterase 11-like - [512929618_XP_004931866.1] | #N/A | 0.5828648 |
|  | PREDICTED: probable pseudouridine-5'-monophosphatase-like isoform X1 - [512927444_XP_004931336.1] | 1.04387652 | 0.5827857 |
|  | PREDICTED: ras-related protein Rab-30-like - [512895974_XP_004923802.1] | #N/A | 0.582686 |
|  | PREDICTED: calreticulin-like - [328720082_XP_001944055.2] | 1.15018951 | 0.5825307 |
|  | small GTP binding protein RAB5 - [112983262_NP_001037614.1] | #N/A | 0.5825115 |
|  | PREDICTED: transmembrane protein 179-like - [512903796_XP_004925705.1] | #N/A | 0.5825023 |
|  | ribosomal protein S7 - [112984058_NP_001037261.1] | 1.10114287 | 0.582306 |
|  | GA28294 - [198463345_XP_002135480.1] | 1.14622219 | 0.5820971 |
|  | PREDICTED: androgen-dependent TFPI-regulating protein-like - [512929254_XP_004931777.1] | 1.22190561 | 0.5820702 |
|  | PREDICTED: synaptojanin-1-like - [345491863_XP_001607775.2] | 1.23627148 | 0.5820358 |
|  | PREDICTED: origin recognition complex subunit 2-like - [512896914_XP_004924025.1] | #N/A | 0.5819772 |
|  | snap-scaffold5149_size13376-abinit-gene-0.3-mRNA-1 | #N/A | 0.5818211 |
|  | PREDICTED: LOW QUALITY PROTEIN: zinc finger HIT domain-containing protein 2-like - [512918223_XP_004929080.1] | #N/A | 0.5817482 |
|  | PREDICTED: cullin-4B-like - [110759504_XP_392800.3] | 1.23924104 | 0.5817218 |
|  | trafficking protein particle complex 1 - [114052112_NP_001040218.1] | 1.20306273 | 0.581328 |
|  | PREDICTED: puromycin-sensitive aminopeptidase-like - [512928179_XP_004931519.1] | 1.13755242 | 0.5813147 |
|  | snap-scaffold2873_size90719-abinit-gene-0.7-mRNA-1 | #N/A | 0.5812963 |
|  | GK13863 - [195451449_XP_002072925.1] | 1.1917825 | 0.5811873 |
|  | PREDICTED: transmembrane and coiled-coil domains protein 1-like - [512918996_XP_004929268.1] | 1.14537375 | 0.5810737 |
|  | maker-scaffold484_size156588-snap-gene-0.21-mRNA-1 | #N/A | 0.5809418 |
|  | PREDICTED: uncharacterized protein LOC101740287 - [512913613_XP_004927939.1] | 1.02955864 | 0.5809341 |
|  | PREDICTED: prostaglandin reductase 1-like - [512931102_XP_004932225.1] | 1.24372578 | 0.580921 |
|  | PREDICTED: mediator of RNA polymerase II transcription subunit 4-like - [512924508_XP_004930612.1] | 1.17374494 | 0.5807126 |
|  | PREDICTED: GPI transamidase component PIG-S-like - [512917782_XP_004928972.1] | 1.16601268 | 0.580662 |
|  | PREDICTED: neurabin-1-like - [512934121_XP_004932957.1] | #N/A | 0.5806563 |
|  | PREDICTED: allantoinase-like - [512900335_XP_004924866.1] | #N/A | 0.5804224 |
|  | PREDICTED: carbonyl reductase [NADPH] 1-like - [512908244_XP_004926634.1] | 1.10355424 | 0.5803517 |
|  | cAMP-dependent protein kinase R1 - [153791441_NP_001093295.1] | 1.16231143 | 0.5801999 |
|  | PREDICTED: carbonyl reductase [NADPH] 1-like - [512908244_XP_004926634.1] | 1.10195665 | 0.5800488 |
|  | PREDICTED: cullin-5-like - [498927451_XP_004518144.1] | 1.21087717 | 0.5800065 |
|  | PREDICTED: general vesicular transport factor p115-like - [512896784_XP_004923993.1] | 1.09627987 | 0.579883 |
|  | PREDICTED: acyl-CoA synthetase family member 3, mitochondrial-like - [512910844_XP_004927270.1] | 1.21629697 | 0.5798042 |
|  | PREDICTED: eukaryotic translation initiation factor 2-alpha kinase-like - [512910191_XP_004927107.1] | 1.18710888 | 0.5795988 |
|  | PREDICTED: exportin-2-like - [512910851_XP_004927272.1] | 1.15452066 | 0.5795203 |
|  | PREDICTED: succinyl-CoA ligase [ADP-forming] subunit beta, mitochondrial-like isoform X1 - [524909457_XP_005109775.1] | 1.17136885 | 0.5793893 |
|  | uncharacterized protein LOC101745924 - [525342815_NP_001266295.1] | 1.16443116 | 0.5793668 |
|  | PREDICTED: T-complex protein 1 subunit delta-like - [391332975_XP_003740901.1] | 1.14406343 | 0.5792817 |
|  | PREDICTED: protein purity of essence-like - [512917049_XP_004928791.1] | 1.1319883 | 0.5792433 |
|  | ELL complex EAP30 subunit - [114051099_NP_001040397.1] | #N/A | 0.5792018 |
|  | PREDICTED: kanadaptin-like - [512905564_XP_004925976.1] | #N/A | 0.5791815 |
|  | PREDICTED: inositol polyphosphate 5-phosphatase OCRL-1-like - [383858844_XP_003704909.1] | 1.20019723 | 0.5790369 |
|  | snap-scaffold13901_size3403-abinit-gene-0.0-mRNA-1 | #N/A | 0.5789994 |
|  | PREDICTED: nuclear export mediator factor NEMF homolog isoform X1 - [512903935_XP_004925739.1] | 0.96990527 | 0.578894 |
|  | PREDICTED: rho guanine nucleotide exchange factor 11-like - [512895827_XP_004923765.1] | 1.00814944 | 0.5788839 |
|  | PREDICTED: uncharacterized protein LOC101742736 - [512907820_XP_004926537.1] | #N/A | 0.5788392 |
|  | DJ-1 beta - [350534642_NP_001232899.1] | 1.13084378 | 0.5788032 |
|  | PREDICTED: U4/U6 small nuclear ribonucleoprotein Prp3-like isoform X1 - [512901346_XP_004925113.1] | 1.06131022 | 0.5787213 |
|  | PREDICTED: rho guanine nucleotide exchange factor 11-like - [328698761_XP_001945506.2] | 1.03773997 | 0.5785422 |
|  | PREDICTED: uncharacterized protein LOC101746353 isoform X2 - [512931018_XP_004932204.1] | #N/A | 0.5785089 |
|  | PREDICTED: 116 kDa U5 small nuclear ribonucleoprotein component-like isoform 4 - [340369212_XP_003383142.1] | 1.1514313 | 0.5785045 |
|  | protein ultraspiracle homolog - [112984304_NP_001037470.1] | 1.14117799 | 0.5783213 |
|  | PREDICTED: uncharacterized protein LOC101745092 - [512915852_XP_004928489.1] | #N/A | 0.5782345 |
|  | PREDICTED: CRAL-TRIO domain-containing protein C589.09, mitochondrial-like - [512913167_XP_004927831.1] | 1.11365604 | 0.5782225 |
|  | PREDICTED: constitutive coactivator of PPAR-gamma-like protein 1 homolog - [383851492_XP_003701266.1] | 1.1628117 | 0.5781988 |
|  | PREDICTED: protein FAM117B-like - [512930842_XP_004932160.1] | 1.00704875 | 0.5778419 |
|  | PREDICTED: malate synthase, glyoxysomal-like - [512892034_XP_004922830.1] | 1.12779255 | 0.5778155 |
|  | PREDICTED: Tat-binding protein-1-like - [291234599_XP_002737235.1] | 1.20755644 | 0.5777947 |
|  | PREDICTED: protein SMG9-like - [345492371_XP_001600565.2] | #N/A | 0.5777354 |
|  | PREDICTED: THO complex subunit 6-like - [512924038_XP_004930500.1] | 1.21032518 | 0.5776206 |
|  | PREDICTED: long-chain-fatty-acid--CoA ligase 1-like - [340723943_XP_003400346.1] | 1.3672693 | 0.5775488 |
|  | PREDICTED: uncharacterized protein LOC101742112 - [512919417_XP_004929373.1] | #N/A | 0.5774058 |
|  | GE21489 - [195491954_XP_002093786.1] | 1.13607935 | 0.5772658 |
|  | PREDICTED: vesicular integral-membrane protein VIP36-like - [512934571_XP_004933065.1] | 1.20572028 | 0.5771868 |
|  | serine/threonine protein kinase - [256077810_XP_002575193.1] | 1.19109404 | 0.5771517 |
|  | PREDICTED: mitogen-activated protein kinase kinase kinase kinase 4-like isoform X5 - [512915926_XP_004928507.1] | #N/A | 0.5771395 |
|  | dicer-2 - [302318908_NP_001180543.1] | 1.12563011 | 0.5770837 |
|  | PREDICTED: ATP-dependent RNA helicase DDX18-like - [512924772_XP_004930677.1] | #N/A | 0.5770834 |
|  | PREDICTED: maternal protein pumilio - [512893044_XP_004923083.1] | 1.06565152 | 0.5770311 |
|  | PREDICTED: tRNA-splicing ligase RtcB homolog - [512900228_XP_004924840.1] | 1.17165781 | 0.5768918 |
|  | PREDICTED: RING finger protein 121-like - [512911044_XP_004927319.1] | #N/A | 0.5768372 |
|  | PREDICTED: CDK5 regulatory subunit-associated protein 3-like - [512937764_XP_004933844.1] | 1.19411838 | 0.576627 |
|  | PREDICTED: alpha-mannosidase 2-like - [512887311_XP_004921902.1] | 1.20812555 | 0.576536 |
|  | PREDICTED: viral IAP-associated factor homolog - [512903856_XP_004925720.1] | 1.18775388 | 0.5765296 |
|  | transaldolase - [114052613_NP_001040544.1] | 1.11283346 | 0.5764567 |
|  | maker-scaffold4760_size30153-snap-gene-0.9-mRNA-1 | #N/A | 0.5761949 |
|  | eIF2B-gamma protein - [112983294_NP_001037654.1] | 1.86103315 | 0.5761738 |
|  | PREDICTED: putative succinate dehydrogenase [ubiquinone] cytochrome b small subunit, mitochondrial-like isoform X1 - [512894333_XP_004923404.1] | #N/A | 0.5761177 |
|  | CRM1 C family protein - [339235717_XP_003379413.1] | 1.17253075 | 0.5760592 |
|  | isocitrate dehydrogenase - [170033046_XP_001844390.1] | 1.1245722 | 0.576054 |
|  | PREDICTED: 26S protease regulatory subunit 8-like - [380029623_XP_003698467.1] | 1.18532901 | 0.5758459 |
|  | GK17139 - [195427287_XP_002061708.1] | 1.21079562 | 0.5758156 |
|  | PREDICTED: pyridine nucleotide-disulfide oxidoreductase domain-containing protein 1-like - [512913602_XP_004927937.1] | #N/A | 0.5757663 |
|  | PREDICTED: ribulose-phosphate 3-epimerase-like - [512926575_XP_004931120.1] | 1.09333659 | 0.5757596 |
|  | PREDICTED: E3 ubiquitin-protein ligase RBBP6-like - [512909915_XP_004927038.1] | 1.23066654 | 0.5757127 |
|  | PREDICTED: aprataxin and PNK-like factor-like isoform X1 - [524913425_XP_005111543.1] | 1.27677235 | 0.575458 |
|  | snap-scaffold28593_size453-abinit-gene-0.0-mRNA-1 | #N/A | 0.5753052 |
|  | PREDICTED: dolichyl-diphosphooligosaccharide--protein glycosyltransferase subunit STT3A-like - [512892902_XP_004923047.1] | 1.12035665 | 0.5752795 |
|  | eukaryotic translation initiation factor 1A - [151301107_NP_001093083.1] | 1.17476123 | 0.5749658 |
|  | phosphate transport protein - [114052589_NP_001040482.1] | 1.15886369 | 0.5748329 |
|  | snap-scaffold10535_size4564-abinit-gene-0.0-mRNA-1 | #N/A | 0.5748281 |
|  | aldehyde dehydrogenase isoform 2 - [114053233_NP_001040290.1] | 1.08915833 | 0.5747867 |
|  | AGAP012408-PA, partial - [158300145_XP_320147.4] | 1.14690932 | 0.5747497 |
|  | putative ATPase, AAA family - [339252154_XP_003371300.1] | 1.17518557 | 0.5745923 |
|  | GK22070 - [195436483_XP_002066197.1] | 1.16176043 | 0.5745778 |
|  | pyruvate dehydrogenase - [153792309_NP_001093304.1] | 1.16920341 | 0.5745193 |
|  | leucine zipper protein - [114050787_NP_001040153.1] | #N/A | 0.5745096 |
|  | GF16133 - [194746378_XP_001955657.1] | 1.13880986 | 0.5744349 |
|  | transmembrane emp24 protein transport domain containing 9 precursor - [114052711_NP_001040538.1] | 1.14348766 | 0.5744189 |
|  | NADH-ubiquinone oxidoreductase subunit B14.7 - [114051374_NP_001040317.1] | 1.14769998 | 0.5743665 |
|  | G protein-coupled receptor kinase 2 - [339240839_XP_003376345.1] | #N/A | 0.5743317 |
|  | PREDICTED: probable cysteine--tRNA ligase, mitochondrial-like - [512902881_XP_004925483.1] | 1.05067102 | 0.5742882 |
|  | PREDICTED: uridine phosphorylase 1-like isoform X1 - [512894460_XP_004923436.1] | 1.07587987 | 0.5742834 |
|  | PREDICTED: regulator of nonsense transcripts 1 homolog - [512920556_XP_004929661.1] | 1.08595052 | 0.5742666 |
|  | PREDICTED: L-galactose dehydrogenase-like isoform X1 - [512934934_XP_004933152.1] | 1.13297288 | 0.5741739 |
|  | PREDICTED: sorting nexin-13-like - [512893636_XP_004923230.1] | #N/A | 0.5741596 |
|  | PREDICTED: cytoplasmic FMR1-interacting protein-like - [156545874_XP_001606532.1] | #N/A | 0.5741345 |
|  | isocitrate dehydrogenase - [284813561_NP_001165386.1] | #N/A | 0.5741277 |
|  | PREDICTED: protein FAM172A-like - [512901091_XP_004925050.1] | 1.09279792 | 0.574106 |
|  | CRE-RME-8 protein - [308476906_XP_003100668.1] | 1.16555092 | 0.5740776 |
|  | AGAP003165-PA - [347969394_XP_312856.4] | 1.12359526 | 0.573977 |
|  | GL12714 - [195172386_XP_002026979.1] | #N/A | 0.5737137 |
|  | ATP-dependent RNA helicase vasa - [94400887_NP_001035345.1] | 1.10356533 | 0.5736924 |
|  | synaptobrevin - [112983402_NP_001037634.1] | 1.02967294 | 0.5735248 |
|  | transferrin precursor - [112983240_NP_001037014.1] | #N/A | 0.5735176 |
|  | PREDICTED: LOW QUALITY PROTEIN: translational activator GCN1-like - [512885819_XP_004921659.1] | 1.15139006 | 0.573458 |
|  | PREDICTED: SH3 domain-binding glutamic acid-rich protein homolog - [512907733_XP_004926516.1] | 1.12192897 | 0.5734221 |
|  | PREDICTED: t-complex protein 1 subunit eta-like - [193577789_XP_001948962.1] | #N/A | 0.5733758 |
|  | PREDICTED: hypothetical protein LOC100638572, partial - [340385252_XP_003391124.1] | #N/A | 0.5732687 |
|  | PREDICTED: heat shock 70 kDa protein 14-like - [512923792_XP_004930438.1] | 1.16556925 | 0.5731238 |
|  | PREDICTED: protein Spindly-like - [512899908_XP_004924762.1] | 1.03367143 | 0.5730485 |
|  | FK506-binding protein FKBP59 homologue - [112983388_NP_001036992.1] | 1.1308249 | 0.5730326 |
|  | death-related protein - [218505765_NP_001136228.1] | 1.16844255 | 0.5729874 |
|  | CRE-KLP-4 protein - [308512099_XP_003118232.1] | 1.10588642 | 0.572936 |
|  | coatomer protein complex subunit beta 2 - [290560891_NP_001166610.1] | 1.22711244 | 0.5729037 |
|  | PREDICTED: etoposide-induced protein 2.4 homolog - [512908922_XP_004926796.1] | 1.26480344 | 0.5726721 |
|  | PREDICTED: transcription elongation regulator 1-like, partial - [512936955_XP_004933646.1] | 1.26352606 | 0.5725947 |
|  | PREDICTED: cytochrome P450 9e2-like - [512908451_XP_004926685.1] | #N/A | 0.5724636 |
|  | PREDICTED: tRNA (adenine(58)-N(1))-methyltransferase catalytic subunit TRMT61A-like - [512927259_XP_004931289.1] | #N/A | 0.5722506 |
|  | microtubule associated-protein orbit - [157109069_XP_001650512.1] | 1.22781046 | 0.5722106 |
|  | PREDICTED: twinfilin-like - [512910078_XP_004927079.1] | 1.1241387 | 0.572095 |
|  | PREDICTED: pleckstrin homology domain-containing family F member 2-like - [512889865_XP_004922392.1] | 1.1988199 | 0.5720759 |
|  | coatomer protein complex subunit alpha - [289629216_NP_001166192.1] | 1.15673121 | 0.5720551 |
|  | PREDICTED: protein purity of essence-like - [512917049_XP_004928791.1] | #N/A | 0.5720279 |
|  | GH13636 - [195051433_XP_001993094.1] | 1.13869384 | 0.5719262 |
|  | PREDICTED: UPF0554 protein C2orf43 homolog - [512923092_XP_004930268.1] | #N/A | 0.5718605 |
|  | PREDICTED: coiled-coil domain-containing protein 96-like - [512905263_XP_004925900.1] | 1.17444486 | 0.5717491 |
|  | dipeptidyl-peptidase - [157109347_XP_001650630.1] | #N/A | 0.5717014 |
|  | PREDICTED: nudix hydrolase 8-like - [512905198_XP_004925884.1] | #N/A | 0.5715503 |
|  | small nuclear ribonucleoprotein Sm D1 - [114050791_NP_001040404.1] | 1.14383298 | 0.5714629 |
|  | PREDICTED: ankyrin repeat and FYVE domain-containing protein 1-like, partial - [512939839_XP_004934339.1] | 1.1152142 | 0.5714242 |
|  | PREDICTED: similar to Toll-interacting protein - [91081565_XP_975168.1] | #N/A | 0.5713298 |
|  | PREDICTED: KH domain-containing, RNA-binding, signal transduction-associated protein 3-like isoform X2 - [512906293_XP_004926160.1] | 1.14190458 | 0.571184 |
|  | snap-scaffold4974_size12883-abinit-gene-0.2-mRNA-1 | #N/A | 0.5711258 |
|  | PREDICTED: prolyl endopeptidase-like - [380014010_XP_003691037.1] | 1.10515398 | 0.5710506 |
|  | Elav protein - [195546808_NP_001091837.2] | 1.21432058 | 0.5710245 |
|  | PREDICTED: ATP-binding cassette sub-family B member 8, mitochondrial-like - [391326514_XP_003737759.1] | 1.09127464 | 0.5708101 |
|  | PREDICTED: DNA primase small subunit-like - [512893742_XP_004923255.1] | #N/A | 0.570775 |
|  | PREDICTED: spectrin beta chain-like - [512915638_XP_004928438.1] | #N/A | 0.5707694 |
|  | PREDICTED: protein NDUFAF4 homolog - [512890866_XP_004922587.1] | 1.18491922 | 0.570768 |
|  | PREDICTED: septin-11-like isoform X1 - [524893568_XP_005103293.1] | 1.08306682 | 0.5706739 |
|  | PREDICTED: helicase SKI2W-like - [512887417_XP_004921921.1] | 1.14461816 | 0.5706595 |
|  | PREDICTED: gamma-tubulin complex component 2-like - [524869423_XP_005091505.1] | 1.14624084 | 0.5704065 |
|  | PREDICTED: estradiol 17-beta-dehydrogenase 8-like isoform X1 - [512916427_XP_004928638.1] | 1.06803919 | 0.5702368 |
|  | DnaJ (Hsp40) homolog 6 - [255652885_NP_001157383.1] | 1.27006386 | 0.5702213 |
|  | GD13118 - [195588274_XP_002083883.1] | 1.00480236 | 0.5697673 |
|  | PREDICTED: peptidyl-prolyl cis-trans isomerase CWC27 homolog - [512935409_XP_004933268.1] | 0.99186713 | 0.5695851 |
|  | PREDICTED: trifunctional purine biosynthetic protein adenosine-3-like - [512901569_XP_004925167.1] | 1.09082093 | 0.5694905 |
|  | beta-tubulin - [112983456_NP_001036888.1] | 1.15121607 | 0.5693423 |
|  | PREDICTED: sorting nexin-2-like - [512889970_XP_004922419.1] | 1.11729223 | 0.569285 |
|  | PREDICTED: hypothetical protein LOC410867 - [328788856_XP_003251195.1] | #N/A | 0.5692747 |
|  | ADP-ribosylation factor-like protein - [114051594_NP_001040168.1] | 1.12052189 | 0.569249 |
|  | actin-depolymerizing factor 1 - [153792659_NP_001093278.1] | 1.14293851 | 0.5692066 |
|  | PREDICTED: acyl-CoA dehydrogenase family member 9, mitochondrial-like isoform X1 - [512906994_XP_004926333.1] | 1.12386192 | 0.5689538 |
|  | PREDICTED: dipeptidyl peptidase 3-like - [350417327_XP_003491368.1] | 1.15247712 | 0.5688119 |
|  | PREDICTED: RING finger and transmembrane domain-containing protein 2-like - [512895093_XP_004923590.1] | 1.16739032 | 0.5688095 |
|  | PREDICTED: 14-3-3 protein zeta-like - [345479702_XP_001600046.2] | 1.11438412 | 0.5686648 |
|  | dicer-2 - [302318908_NP_001180543.1] | 1.13432337 | 0.5685874 |
|  | UDP-glucose 4-epimerase - [157115992_XP_001652750.1] | 1.10003782 | 0.5685592 |
|  | dicer-2 - [302318908_NP_001180543.1] | 1.10259384 | 0.5685469 |
|  | LSM Sm-like protein family member - [114051632_NP_001040418.1] | #N/A | 0.5684005 |
|  | DCMP deaminase - [114052458_NP_001040508.1] | #N/A | 0.5683979 |
|  | H+ transporting ATPase V0 subunit D - [114051764_NP_001040429.1] | 1.15329856 | 0.5683259 |
|  | phosphoglucomutase - [157129577_XP_001661733.1] | #N/A | 0.5682138 |
|  | 14-3-3 epsilon protein - [148298752_NP_001091764.1] | 1.11989222 | 0.568179 |
|  | PREDICTED: spermatogenesis-associated protein 20-like - [328702149_XP_001952649.2] | 1.17245718 | 0.5680942 |
|  | PREDICTED: esterase FE4-like - [512899307_XP_004924611.1] | 1.10599669 | 0.5680005 |
|  | PREDICTED: protein piccolo-like - [512890952_XP_004922601.1] | 1.05303276 | 0.5679439 |
|  | PREDICTED: trafficking protein particle complex subunit 4-like - [512909469_XP_004926929.1] | 1.13745276 | 0.5678956 |
|  | PREDICTED: nucleoporin NDC1-like - [512924473_XP_004930604.1] | 1.16179349 | 0.5678196 |
|  | PREDICTED: DNA damage-binding protein 1-like - [512897190_XP_004924093.1] | #N/A | 0.5678117 |
|  | snap-scaffold9_size109563-abinit-gene-0.4-mRNA-1 | #N/A | 0.5677638 |
|  | protein tyrosine phosphatase - [350536615_NP_001232932.1] | 1.17682092 | 0.5677415 |
|  | PREDICTED: insulin-like growth factor 2 mRNA-binding protein 1-like isoform X4 - [512921567_XP_004929908.1] | 1.13653295 | 0.5676871 |
|  | PREDICTED: geranylgeranyl transferase type-2 subunit alpha-like - [512926202_XP_004931026.1] | #N/A | 0.5676346 |
|  | PREDICTED: SRR1-like protein-like - [512924819_XP_004930686.1] | 1.13757334 | 0.5674657 |
|  | PREDICTED: valine--tRNA ligase-like isoform X1 - [512895378_XP_004923658.1] | 1.18292967 | 0.5674124 |
|  | PREDICTED: sperm-associated antigen 7 homolog - [512897437_XP_004924152.1] | #N/A | 0.5673537 |
|  | PREDICTED: sulfhydryl oxidase 1-like - [512921696_XP_004929937.1] | #N/A | 0.5671674 |
|  | maker-scaffold2367_size29331-snap-gene-0.5-mRNA-1 | #N/A | 0.567002 |
|  | seven in absentia - [170060566_XP_001865860.1] | 1.15091692 | 0.5669345 |
|  | PREDICTED: nardilysin-like - [345478824_XP_001599332.2] | 1.26535027 | 0.5665648 |
|  | PREDICTED: uncharacterized protein LOC101746654, partial - [512895923_XP_004923789.1] | 1.2411401 | 0.56656 |
|  | 6-phosphogluconate dehydrogenase, decarboxylating - [170594611_XP_001902057.1] | 1.1494653 | 0.5665451 |
|  | casein kinase I alpha - [112983854_NP_001037287.1] | 1.15302891 | 0.5664619 |
|  | PREDICTED: uncharacterized protein LOC101745679 - [512917189_XP_004928826.1] | 1.14761028 | 0.5663591 |
|  | small GTP-binding protein - [112983246_NP_001037010.1] | 1.148715 | 0.5663506 |
|  | PREDICTED: RNA-binding protein 45-like - [383850196_XP_003700683.1] | 1.0275867 | 0.5663238 |
|  | ribosomal protein L19 - [112984306_NP_001037221.1] | 1.10073411 | 0.5662389 |
|  | short-chain dehydrogenease/reductase - [114050771_NP_001040154.1] | 1.1544981 | 0.5662309 |
|  | PREDICTED: TBC1 domain family member 9-like - [512893775_XP_004923262.1] | 1.61829394 | 0.5661375 |
|  | PREDICTED: similar to AGAP011535-PA - [91087169_XP_975384.1] | 1.14535021 | 0.5661282 |
|  | PREDICTED: U5 small nuclear ribonucleoprotein 200 kDa helicase-like - [524889162_XP_005101142.1] | 1.1246329 | 0.5661212 |
|  | PREDICTED: T-complex protein 1 subunit gamma-like - [512920757_XP_004929712.1] | 1.15667123 | 0.5660443 |
|  | AGAP007494-PA - [158285579_XP_308381.4] | 1.11637585 | 0.5658374 |
|  | PREDICTED: plasma membrane calcium-transporting ATPase 3-like isoform X4 - [512905930_XP_004926069.1] | 1.08261271 | 0.5657348 |
|  | PREDICTED: TAR DNA-binding protein 43-like - [512899479_XP_004924655.1] | 1.10687478 | 0.565674 |
|  | PREDICTED: MATH and LRR domain-containing protein PFE0570w-like - [512929539_XP_004931846.1] | #N/A | 0.5656148 |
|  | PREDICTED: elongation of very long chain fatty acids protein 6-like - [512900028_XP_004924792.1] | 1.16802748 | 0.5655238 |
|  | PREDICTED: LOW QUALITY PROTEIN: NEDD8-activating enzyme E1 regulatory subunit - [328791044_XP_003251507.1] | 1.17391022 | 0.5654483 |
|  | PREDICTED: d-3-phosphoglycerate dehydrogenase-like - [512934875_XP_004933138.1] | 1.13165066 | 0.5654324 |
|  | PREDICTED: ubiA prenyltransferase domain-containing protein 1 homolog - [512928940_XP_004931703.1] | #N/A | 0.5654297 |
|  | PREDICTED: zinc finger FYVE domain-containing protein 26-like - [512887359_XP_004921911.1] | #N/A | 0.5653495 |
|  | PREDICTED: phosphatidylinositol 4-phosphate 3-kinase C2 domain-containing subunit alpha-like - [512914269_XP_004928100.1] | 1.00295598 | 0.5652449 |
|  | PREDICTED: mitochondrial thiamine pyrophosphate carrier-like - [512933964_XP_004932920.1] | #N/A | 0.5652008 |
|  | PREDICTED: protein polybromo-1-like - [328719779_XP_001944619.2] | 1.21556578 | 0.5651231 |
|  | PREDICTED: solute carrier family 35 member F5-like, partial - [512890248_XP_004922485.1] | #N/A | 0.5650392 |
|  | PREDICTED: LOW QUALITY PROTEIN: oxysterol-binding protein 2-like - [512909332_XP_004926895.1] | #N/A | 0.5650297 |
|  | PREDICTED: RecQ protein-like (DNA helicase Q1-like)-like - [291228382_XP_002734149.1] | #N/A | 0.5649631 |
|  | PREDICTED: methionine aminopeptidase 2-like isoform X1 - [512907476_XP_004926452.1] | 1.14409025 | 0.5649465 |
|  | predicted protein - [156362593_XP_001625860.1] | 1.11148214 | 0.5649344 |
|  | PREDICTED: integrator complex subunit 3 homolog isoform X1 - [498957789_XP_004524209.1] | 1.18050888 | 0.5649131 |
|  | PREDICTED: exocyst complex component 2-like - [512893013_XP_004923076.1] | 1.07044836 | 0.5645325 |
|  | prefoldin beta subunit - [114051930_NP_001040192.1] | 1.05971866 | 0.5644297 |
|  | PREDICTED: uncharacterized protein LOC101737690 - [512916651_XP_004928691.1] | 1.20725925 | 0.5643577 |
|  | PREDICTED: elongation factor Tu GTP-binding domain-containing protein 1-like - [512920932_XP_004929757.1] | #N/A | 0.5642537 |
|  | glyoxylate reductase/hydroxypyruvate reductase - [114053007_NP_001040540.1] | 1.1007004 | 0.564132 |
|  | PREDICTED: hemocyte protein-glutamine gamma-glutamyltransferase-like - [512936208_XP_004933462.1] | 3.41560913 | 0.5641143 |
|  | Protein AHCY-1 - [17506425_NP_491955.1] | 1.13506235 | 0.5640522 |
|  | cytochrome P450, family 6, subfamily ab, polypeptide 5 - [160358393_NP_001104007.1] | 1.0866847 | 0.5640258 |
|  | PREDICTED: rac GTPase-activating protein 1-like isoform X1 - [512901613_XP_004925178.1] | 0.9811059 | 0.5640063 |
|  | PREDICTED: alpha-(1,6)-fucosyltransferase-like - [512914516_XP_004928161.1] | 1.08697472 | 0.5639492 |
|  | PREDICTED: LOW QUALITY PROTEIN: kinesin-like protein KIF13A-like - [512923019_XP_004930257.1] | #N/A | 0.5639046 |
|  | PREDICTED: F-box/WD repeat-containing protein 9-like isoform X1 - [512922171_XP_004930055.1] | 1.13056001 | 0.5637673 |
|  | PREDICTED: ADP-dependent glucokinase-like isoform X1 - [512914532_XP_004928165.1] | 1.07185007 | 0.5636828 |
|  | PREDICTED: aladin-like - [512896694_XP_004923972.1] | 1.11873376 | 0.5636005 |
|  | PREDICTED: phosphatidylinositol 4-phosphate 3-kinase C2 domain-containing subunit alpha-like - [512914269_XP_004928100.1] | #N/A | 0.5635951 |
|  | uridine 5'-monophosphate synthase - [114050831_NP_001040160.1] | 1.11661434 | 0.5634897 |
|  | coatomer beta subunit - [256073322_XP_002572980.1] | 1.18687375 | 0.5634516 |
|  | snap-scaffold656_size59057-abinit-gene-0.16-mRNA-1 | #N/A | 0.5634034 |
|  | PREDICTED: glutathione S-transferase D7-like, partial - [512924026_XP_004930497.1] | 1.14106954 | 0.5633925 |
|  | PREDICTED: hydroxyacyl-coenzyme A dehydrogenase, mitochondrial-like - [340368342_XP_003382711.1] | 1.10589771 | 0.5633461 |
|  | PREDICTED: uncharacterized protein LOC101740488 - [512894920_XP_004923547.1] | 1.12497571 | 0.5632766 |
|  | PREDICTED: eukaryotic translation initiation factor 3, subunit 6 48kDa-like - [291230151_XP_002735030.1] | 1.12764751 | 0.5631689 |
|  | PREDICTED: esterase FE4-like - [512899311_XP_004924612.1] | 1.08074994 | 0.5629961 |
|  | PREDICTED: eukaryotic translation initiation factor 5B-like isoform X2 - [512913656_XP_004927949.1] | 1.1752499 | 0.5629354 |
|  | AGAP002603-PA - [158290767_XP_312333.3] | 1.07059842 | 0.5628348 |
|  | GI12751 - [195125265_XP_002007102.1] | 1.16057352 | 0.5626111 |
|  | PREDICTED: exocyst complex component 3-like - [383855052_XP_003703033.1] | 1.2052972 | 0.5623731 |
|  | PREDICTED: protein lin-37 homolog isoform X1 - [512895409_XP_004923665.1] | #N/A | 0.5622848 |
|  | PREDICTED: tubulin alpha chain-like - [340725412_XP_003401064.1] | 1.14692764 | 0.5621357 |
|  | adapter-related protein complex 1 beta subunit - [256092820_XP_002582075.1] | 1.16512759 | 0.5621149 |
|  | H+ transporting ATP synthase gamma subunit - [114051740_NP_001040428.1] | 1.0977228 | 0.5620135 |
|  | DEAD box ATP-dependent RNA helicase - [157126319_XP_001654592.1] | 1.13786546 | 0.5619368 |
|  | PREDICTED: cohesin subunit SA-1-like - [512910140_XP_004927094.1] | 1.08841431 | 0.5615199 |
|  | mannose-P-dolichol utilization defect 1 protein - [158635927_NP_001040213.1] | 1.14919678 | 0.5615162 |
|  | interphase cyctoplasmic foci protein 45 - [114051932_NP_001040194.1] | 1.17018682 | 0.5615157 |
|  | AGAP003397-PA - [347969816_XP_311683.4] | 1.19561464 | 0.5614831 |
|  | PREDICTED: serine/threonine-protein kinase mTOR - [391340134_XP_003744400.1] | 1.26823286 | 0.5614678 |
|  | PREDICTED: selenoprotein O-like isoform 3 - [390367287_XP_786396.3] | 1.05266266 | 0.561454 |
|  | PREDICTED: hemicentin-2-like - [328698595_XP_003240678.1] | 1.04264313 | 0.5610618 |
|  | PREDICTED: SHC-transforming protein 1-like - [512932018_XP_004932442.1] | 1.12021262 | 0.5609615 |
|  | Protein GDI-1, isoform b - [115533048_NP_001041044.1] | 1.15350316 | 0.5609251 |
|  | PREDICTED: glycogen synthase kinase-3 beta isoform X3 - [512915019_XP_004928286.1] | 1.17906226 | 0.5606195 |
|  | PREDICTED: LOW QUALITY PROTEIN: MMS19 nucleotide excision repair protein homolog - [380024638_XP_003696100.1] | 1.08055533 | 0.56061 |
|  | protein kinase c inhibitor - [112983493_NP_001037526.1] | 1.11053016 | 0.5604916 |
|  | CRE-PAB-1 protein - [308499747_XP_003112059.1] | 1.19168174 | 0.5604315 |
|  | PREDICTED: sarcolemmal membrane-associated protein-like - [512931435_XP_004932302.1] | 1.1363123 | 0.5603695 |
|  | PREDICTED: UDP-xylose and UDP-N-acetylglucosamine transporter-like - [512901585_XP_004925171.1] | 1.12692426 | 0.560219 |
|  | PREDICTED: threonine--tRNA ligase, cytoplasmic-like isoform X2 - [512936200_XP_004933460.1] | 1.15899706 | 0.5602149 |
|  | transcription initiation factor IIA gamma chain - [114052188_NP_001040463.1] | 1.13940818 | 0.5600986 |
|  | PREDICTED: protein arginine N-methyltransferase 8-like - [512897194_XP_004924094.1] | 1.11770307 | 0.560097 |
|  | Myb transcription factor - [382546244_NP_001244265.1] | #N/A | 0.5600783 |
|  | NIF3-like protein - [148298856_NP_001091745.1] | 1.08912188 | 0.5600281 |
|  | PREDICTED: ribosome biogenesis protein TSR3 homolog - [512930109_XP_004931986.1] | #N/A | 0.5598332 |
|  | PREDICTED: transcription elongation factor SPT6-like - [512931471_XP_004932311.1] | #N/A | 0.5598092 |
|  | PREDICTED: ubiquilin-1-like isoform X2 - [512920622_XP_004929678.1] | 1.16605195 | 0.5597896 |
|  | PREDICTED: similar to predicted protein - [91078842_XP_971622.1] | 1.15384761 | 0.5597757 |
|  | PREDICTED: protein KTI12 homolog - [512935393_XP_004933264.1] | 1.2420349 | 0.5595846 |
|  | PREDICTED: spermine synthase-like - [512900064_XP_004924801.1] | 1.18007621 | 0.5595515 |
|  | PREDICTED: uncharacterized protein LOC101741771 - [512912602_XP_004927699.1] | #N/A | 0.5594764 |
|  | PREDICTED: similar to RHO guanyl-nucleotide exchange factor - [189238697_XP_967832.2] | #N/A | 0.5594335 |
|  | PREDICTED: FACT complex subunit Ssrp1 - [110766132_XP_001120264.1] | 1.15303079 | 0.5593921 |
|  | glycerol-3-phosphate dehydrogenase - [62526114_NP_001014994.1] | 1.12939928 | 0.5592273 |
|  | PREDICTED: probable GDP-fucose transporter-like isoform X1 - [512913771_XP_004927974.1] | 1.09093729 | 0.5592209 |
|  | PREDICTED: non-specific lipid-transfer protein-like - [345478896_XP_001607735.2] | 1.0944103 | 0.5592029 |
|  | PREDICTED: phosphoglycerate kinase isoform 1 - [110763826_XP_395047.3] | #N/A | 0.5591778 |
|  | PREDICTED: nodal modulator 3-like - [512911180_XP_004927350.1] | 1.13332245 | 0.5590649 |
|  | uncharacterized protein LOC100174822 - [195963351_NP_001124348.1] | 1.26599901 | 0.5590624 |
|  | PREDICTED: suppressor of G2 allele of SKP1 homolog - [512925205_XP_004930782.1] | 1.21456178 | 0.5590178 |
|  | PREDICTED: FK506-binding protein 2-like - [345497242_XP_001599993.2] | 1.08288474 | 0.5589198 |
|  | PREDICTED: nodal modulator 3-like - [512911180_XP_004927350.1] | 1.14882481 | 0.5588244 |
|  | PREDICTED: gonadal protein gdl-like - [512929985_XP_004931958.1] | 1.21191359 | 0.5584248 |
|  | PREDICTED: T-cell activation inhibitor, mitochondrial-like - [512919891_XP_004929495.1] | #N/A | 0.5583758 |
|  | PREDICTED: transmembrane protein 55B-like isoform X1 - [512928143_XP_004931510.1] | 1.11298733 | 0.5582664 |
|  | PREDICTED: G kinase-anchoring protein 1-like isoform X1 - [512920544_XP_004929658.1] | #N/A | 0.5581744 |
|  | GD21557 - [195575219_XP_002105577.1] | 1.12377924 | 0.5581104 |
|  | PREDICTED: polyubiquitin-like, partial - [340382024_XP_003389521.1] | 1.13440608 | 0.5581017 |
|  | PREDICTED: cytoplasmic aconitate hydratase-like - [512888904_XP_004922163.1] | 1.10363324 | 0.5580947 |
|  | ras-like protein 2 - [112983376_NP_001036993.1] | #N/A | 0.557988 |
|  | PREDICTED: tetratricopeptide repeat protein 1-like - [512911573_XP_004927446.1] | 1.16603611 | 0.5577082 |
|  | PREDICTED: alpha-actinin, sarcomeric-like isoform 1 - [340721945_XP_003399373.1] | 0.5575241 |  |
|  | UDP-galactose 4-epimerase - [114052166_NP_001040224.1] | 1.06920243 | 0.5574436 |
|  | PREDICTED: chromatin complexes subunit BAP18-like isoform X4 - [512935078_XP_004933186.1] | 1.14841854 | 0.5572241 |
|  | transcription factor-like protein - [114050713_NP_001040313.1] | 1.07505405 | 0.5569636 |
|  | PREDICTED: plasminogen activator inhibitor 1 RNA-binding protein-like - [512923489_XP_004930365.1] | 1.12937193 | 0.5569361 |
|  | importin subunit beta-1 - [339253254_XP_003371850.1] | 1.17138589 | 0.556933 |
|  | alpha spectrin, isoform B - [442629558_NP_001261286.1] | 1.17945798 | 0.556876 |
|  | PREDICTED: Pyruvate dehydrogenase [acetyl-transferring]-phosphatase 1-like isoform X1 - [512921513_XP_004929896.1] | 1.20722104 | 0.5568272 |
|  | PREDICTED: dolichyl-phosphate beta-glucosyltransferase-like - [512912864_XP_004927759.1] | 1.18417032 | 0.556662 |
|  | snap-scaffold4768_size20349-abinit-gene-0.4-mRNA-1 | #N/A | 0.556563 |
|  | brunelleschi - [24585462_NP_610044.2] | #N/A | 0.5565436 |
|  | PREDICTED: major facilitator superfamily domain-containing protein 10-like - [512932269_XP_004932504.1] | #N/A | 0.5564362 |
|  | ras-related GTP-binding protein Rab11 - [112983314_NP_001037618.1] | 1.17672057 | 0.5564238 |
|  | PREDICTED: peroxisomal membrane protein 11C-like - [512901934_XP_004925253.1] | #N/A | 0.5563398 |
|  | AGAP009439-PA, partial - [158284767_XP_307851.4] | 1.15777397 | 0.5562608 |
|  | vacuolar protein sorting (vps33) - [157137649_XP_001657113.1] | #N/A | 0.5562592 |
|  | proteasome subunit alpha type 6-A - [114052160_NP_001040459.1] | 1.16474529 | 0.5560355 |
|  | glutathione peroxidase - [112983348_NP_001036999.1] | 1.13602926 | 0.555986 |
|  | PREDICTED: integrator complex subunit 12-like - [383856036_XP_003703516.1] | 1.1410166 | 0.555924 |
|  | PREDICTED: COP9 signalosome complex subunit 3-like - [156537217_XP_001605022.1] | 1.17085533 | 0.5558019 |
|  | GJ23829 - [195389358_XP_002053344.1] | #N/A | 0.555745 |
|  | snap-scaffold3213_size88406-abinit-gene-0.6-mRNA-1 | #N/A | 0.5557168 |
|  | alanyl-tRNA synthetase - [256077790_XP_002575183.1] | 1.15128308 | 0.555715 |
|  | PREDICTED: mitochondrial cardiolipin hydrolase-like - [512916423_XP_004928637.1] | #N/A | 0.5556725 |
|  | PREDICTED: apoptosis-inducing factor 3-like - [512909328_XP_004926894.1] | 1.05827296 | 0.5554669 |
|  | GG22462 - [194883238_XP_001975710.1] | 1.08520881 | 0.5554577 |
|  | PREDICTED: LOW QUALITY PROTEIN: oxysterol-binding protein-related protein 8-like - [512889372_XP_004922262.1] | 1.22124407 | 0.5553566 |
|  | RPA43_N protein - [289629226_NP_001166197.1] | #N/A | 0.5553519 |
|  | PREDICTED: protein arginine N-methyltransferase 5-like isoform X1 - [512916114_XP_004928553.1] | 1.14767528 | 0.5552152 |
|  | PREDICTED: protein disulfide-isomerase A5-like isoform X1 - [512921261_XP_004929834.1] | 1.12484019 | 0.5551381 |
|  | GI10395 - [195112364_XP_002000744.1] | 1.20342987 | 0.5548697 |
|  | PREDICTED: cytoplasmic aconitate hydratase-like - [512888904_XP_004922163.1] | 1.12738541 | 0.5545818 |
|  | JAB-MPN domain protein - [223890174_NP_001138802.1] | 1.15513135 | 0.5545342 |
|  | muscle glycogen phosphorylase - [182509200_NP_001116811.1] | 1.13140006 | 0.554507 |
|  | snap-scaffold228_size128950-abinit-gene-0.25-mRNA-1 | 1.14720464 | 0.5544934 |
|  | PREDICTED: eukaryotic translation initiation factor 2A-like - [512899475_XP_004924654.1] | 1.14936679 | 0.5544503 |
|  | PREDICTED: phosphatidylinositol N-acetylglucosaminyltransferase subunit H-like - [115739596_XP_001177880.1] | #N/A | 0.5543319 |
|  | PREDICTED: pre-mRNA 3' end processing protein WDR33-like isoform X1 - [512929243_XP_004931774.1] | 1.20941194 | 0.5543073 |
|  | PREDICTED: neurochondrin homolog - [512887085_XP_004921864.1] | 1.14818205 | 0.554259 |
|  | PREDICTED: eukaryotic translation initiation factor 3 subunit A-like isoform X2 - [512917662_XP_004928942.1] | 1.13306077 | 0.5542506 |
|  | lola like, isoform A - [17865642_NP_524778.1] | 1.15468647 | 0.5541025 |
|  | PREDICTED: RPII140-upstream gene protein-like - [512910074_XP_004927078.1] | 1.21003644 | 0.5537834 |
|  | PREDICTED: probable nucleoporin Nup58-like - [512912068_XP_004927565.1] | 1.12822286 | 0.55369 |
|  | PREDICTED: kynurenine--oxoglutarate transaminase 3-like isoform X1 - [512916446_XP_004928642.1] | 1.08931201 | 0.5535931 |
|  | PREDICTED: Glutathione S-Transferase family member (gst-19)-like - [291227913_XP_002733919.1] | 1.10613527 | 0.5535344 |
|  | snap-scaffold7304_size18297-abinit-gene-0.2-mRNA-1 | 1.20199589 | 0.5534827 |
|  | PREDICTED: plastin-2-like - [512894777_XP_004923511.1] | 1.13347473 | 0.5534746 |
|  | PREDICTED: protein unc-45 homolog B-like isoform X1 - [524875757_XP_005094602.1] | 1.20343251 | 0.5534472 |
|  | PREDICTED: DEP domain-containing protein 5-like - [512893617_XP_004923226.1] | #N/A | 0.5533928 |
|  | PREDICTED: adenylosuccinate lyase-like - [193613382_XP_001945655.1] | 1.12896753 | 0.5533476 |
|  | PREDICTED: protein HGV2-like isoform X1 - [512932425_XP_004932541.1] | #N/A | 0.5531854 |
|  | PREDICTED: UDP-sugar transporter UST74c-like - [512933685_XP_004932852.1] | #N/A | 0.5531016 |
|  | PREDICTED: calcineurin-binding protein cabin-1-like - [512904887_XP_004925808.1] | 1.21896988 | 0.5530359 |
|  | PREDICTED: sphingosine-1-phosphate lyase-like - [512892522_XP_004922952.1] | 1.25395476 | 0.5527614 |
|  | PREDICTED: lysosomal acid phosphatase-like - [512892640_XP_004922981.1] | 1.19562196 | 0.5527457 |
|  | PREDICTED: eukaryotic translation initiation factor 3 subunit L-like isoform 2 - [115908481_XP_795841.2] | 1.17279967 | 0.552725 |
|  | PREDICTED: neutral alpha-glucosidase AB-like isoform X3 - [512893254_XP_004923136.1] | 1.15315226 | 0.5526938 |
|  | shibire, isoform N - [442616503_NP_001259588.1] | 1.18261569 | 0.552655 |
|  | PREDICTED: cytoplasmic aconitate hydratase-like - [512888904_XP_004922163.1] | 1.11070086 | 0.5526346 |
|  | conserved hypothetical protein - [170054825_XP_001863306.1] | 1.20067656 | 0.5525499 |
|  | PREDICTED: inositol hexakisphosphate and diphosphoinositol-pentakisphosphate kinase-like - [350424817_XP_003493922.1] | #N/A | 0.5525175 |
|  | Cmc1 protein - [226502030_NP_001140193.1] | #N/A | 0.5523212 |
|  | maker-scaffold5079_size12465-snap-gene-0.4-mRNA-1 | #N/A | 0.5522014 |
|  | PREDICTED: ATP-binding cassette sub-family D member 3-like - [512937461_XP_004933772.1] | 1.07644012 | 0.5521793 |
|  | PREDICTED: protein sax-3-like - [512914233_XP_004928091.1] | #N/A | 0.5521268 |
|  | SUMO-1 activating enzyme - [114052607_NP_001040485.1] | 1.15222627 | 0.5520803 |
|  | PREDICTED: E3 UFM1-protein ligase 1 homolog - [350412410_XP_003489635.1] | 1.1329184 | 0.551913 |
|  | PREDICTED: nuclear pore complex protein Nup205-like - [512916639_XP_004928688.1] | 1.12117667 | 0.5517135 |
|  | glutamate synthase - [339242467_XP_003377159.1] | 1.1224863 | 0.5516479 |
|  | PREDICTED: sulfatase-modifying factor 1-like - [512929965_XP_004931953.1] | 1.08714217 | 0.5515638 |
|  | PREDICTED: thioredoxin-related transmembrane protein 2 homolog - [512901322_XP_004925107.1] | 1.20699882 | 0.5513787 |
|  | CRE-PPTR-2 protein - [308476346_XP_003100389.1] | 1.16322834 | 0.5511227 |
|  | AGAP005627-PB - [118786767_XP_315641.3] | 1.12815934 | 0.5510189 |
|  | PREDICTED: abhydrolase domain-containing protein 16A-like - [512933669_XP_004932848.1] | 1.10285697 | 0.5509995 |
|  | cytidylate kinase - [114051530_NP_001040356.1] | 1.15428038 | 0.5509351 |
|  | PREDICTED: pre-mRNA 3'-end-processing factor FIP1-like isoform X1 - [512892735_XP_004923005.1] | 1.24185467 | 0.5509171 |
|  | mago nashi - [112983378_NP_001037630.1] | 1.23833216 | 0.5508896 |
|  | peroxisomal membrane protein PMP22 - [114053251_NP_001040524.1] | 1.15474907 | 0.5508349 |
|  | PREDICTED: transcription elongation factor SPT6-like - [512931471_XP_004932311.1] | 1.08549649 | 0.5508166 |
|  | GF16133 - [194746378_XP_001955657.1] | #N/A | 0.5506215 |
|  | PREDICTED: LOW QUALITY PROTEIN: myosin heavy chain, non-muscle-like - [512887957_XP_004922008.1] | 1.08174244 | 0.5504744 |
|  | PREDICTED: S1 RNA-binding domain-containing protein 1-like isoform X1 - [512923763_XP_004930431.1] | 1.18766157 | 0.5503919 |
|  | AGAP007623-PA - [158285331_XP_308248.4] | #N/A | 0.5503579 |
|  | PREDICTED: secretory carrier-associated membrane protein 1-like - [512908701_XP_004926746.1] | 1.19764179 | 0.550216 |
|  | PREDICTED: probable prefoldin subunit 4-like - [512928008_XP_004931476.1] | 1.1455614 | 0.550191 |
|  | PREDICTED: peroxisomal multifunctional enzyme type 2-like - [512935985_XP_004933409.1] | 1.10714109 | 0.5501687 |
|  | glycerol-3-phosphate dehydrogenase-1 - [112983418_NP_001036970.1] | #N/A | 0.5501173 |
|  | PREDICTED: sepiapterin reductase-like - [512898507_XP_004924416.1] | 1.19266576 | 0.5500478 |
|  | PREDICTED: protein phosphatase 1B-like isoform X1 - [512897166_XP_004924087.1] | 1.12747478 | 0.55 |
|  | PREDICTED: trifunctional enzyme subunit alpha, mitochondrial-like - [345495602_XP_001605350.2] | 1.1383012 | 0.5498958 |
|  | GF15487 - [194760505_XP_001962480.1] | #N/A | 0.5498565 |
|  | RP42 - [170586718_XP_001898126.1] | 1.11005258 | 0.5498483 |
|  | GG18891 - [194889931_XP_001977192.1] | 1.13490951 | 0.5497386 |
|  | PREDICTED: similar to vesicle associated protein, putative - [91087995_XP_973673.1] | 1.21547119 | 0.5495519 |
|  | PREDICTED: protein PXR1-like - [512910847_XP_004927271.1] | 1.28682394 | 0.549489 |
|  | PREDICTED: protein EFR3 homolog B-like isoform X1 - [524877100_XP_005095257.1] | #N/A | 0.5493323 |
|  | PREDICTED: AT-rich interactive domain-containing protein 2-like - [512891588_XP_004922722.1] | 1.14378993 | 0.5493035 |
|  | PREDICTED: serine--tRNA ligase, mitochondrial-like - [512930546_XP_004932086.1] | 1.09918943 | 0.5492889 |
|  | maker-scaffold33_size132850-snap-gene-0.25-mRNA-1 | 1.1694434 | 0.5492811 |
|  | PREDICTED: ankyrin repeat and MYND domain-containing protein 2-like - [512923566_XP_004930383.1] | 1.13700607 | 0.5491669 |
|  | PREDICTED: CCR4-NOT transcription complex subunit 11-like - [512909732_XP_004926993.1] | 1.14452612 | 0.5490994 |
|  | PREDICTED: glutathione S-transferase D7-like, partial - [512924026_XP_004930497.1] | 1.38920304 | 0.5490964 |
|  | PREDICTED: protein BCCIP homolog - [512934796_XP_004933119.1] | 1.16194285 | 0.5490738 |
|  | AGAP002895-PA - [347968780_XP_312015.5] | 1.14229772 | 0.5490633 |
|  | PREDICTED: ubiquitin-like with PHD and ring finger domains 1-like - [291242193_XP_002740994.1] | 1.15803087 | 0.5490574 |
|  | snap-scaffold5642_size52275-abinit-gene-0.0-mRNA-1 | #N/A | 0.5490207 |
|  | PREDICTED: glycine cleavage system H protein, mitochondrial-like isoform X1 - [512933861_XP_004932896.1] | 1.15940489 | 0.5489562 |
|  | PREDICTED: similar to rCG61344 - [189242212_XP_967555.2] | 1.14753435 | 0.5488019 |
|  | fatty acid transport protein - [197209926_NP_001127727.1] | 1.0719235 | 0.5486142 |
|  | GK19517 - [195429459_XP_002062776.1] | 1.16931721 | 0.5485483 |
|  | clathrin coat assembly protein AP50 - [170035332_XP_001845524.1] | 1.24420477 | 0.5482505 |
|  | PREDICTED: la-related protein 7-like - [512908957_XP_004926804.1] | 1.07194731 | 0.5480999 |
|  | PREDICTED: LOW QUALITY PROTEIN: protein 4.1 homolog - [512905888_XP_004926058.1] | 1.14075719 | 0.5480944 |
|  | PREDICTED: protein arginine N-methyltransferase 8-like - [512897194_XP_004924094.1] | 1.10433315 | 0.5480666 |
|  | putative signal recognition particle 68 kDa protein - [148298713_NP_001091777.1] | 1.1233228 | 0.5477508 |
|  | PREDICTED: zinc finger protein 813-like - [512925158_XP_004930770.1] | #N/A | 0.5476925 |
|  | AGAP009127-PA - [158299870_XP_553055.2] | 1.04227177 | 0.5476352 |
|  | PREDICTED: serine/threonine-protein phosphatase PP1-beta catalytic subunit-like isoform 2 - [345491368_XP_003426582.1] | 1.18048994 | 0.5475529 |
|  | PREDICTED: NADH dehydrogenase [ubiquinone] 1 beta subcomplex subunit 7-like - [512907496_XP_004926457.1] | 1.06069307 | 0.5474178 |
|  | PREDICTED: putative ATP-dependent Clp protease proteolytic subunit, mitochondrial-like - [512886601_XP_004921785.1] | 1.1609474 | 0.5473099 |
|  | PREDICTED: similar to AGAP006755-PA isoform 1 - [91078908_XP_967303.1] | #N/A | 0.547281 |
|  | PREDICTED: trichohyalin-like - [512919667_XP_004929441.1] | 1.03143992 | 0.5472396 |
|  | PREDICTED: LOW QUALITY PROTEIN: fumarate hydratase, mitochondrial-like - [512909069_XP_004926832.1] | 1.13116502 | 0.5471667 |
|  | PREDICTED: transcription termination factor 2-like - [512894452_XP_004923434.1] | 1.02614225 | 0.5470663 |
|  | PREDICTED: TIP41-like protein-like - [512914024_XP_004928038.1] | 1.08561849 | 0.5470182 |
|  | PREDICTED: programmed cell death 6-interacting protein-like - [512908162_XP_004926613.1] | 1.35619192 | 0.5469022 |
|  | snap-scaffold150_size368343-abinit-gene-2.18-mRNA-1 | 1.26866793 | 0.5468892 |
|  | PREDICTED: transient receptor potential channel pyrexia - [110755702_XP_001122445.1] | 0.5468595 |  |
|  | WW domain binding protein 4 - [114050857_NP_001040408.1] | 1.12919493 | 0.5467835 |
|  | PREDICTED: peroxiredoxin-4-like - [512926760_XP_004931166.1] | 1.1260171 | 0.5467798 |
|  | PREDICTED: uncharacterized protein CG16817-like isoform X1 - [512915880_XP_004928496.1] | 1.09405552 | 0.5467774 |
|  | snap-scaffold1497_size85809-abinit-gene-0.20-mRNA-1 | 1.19543749 | 0.5467433 |
|  | heat shock factor-d - [379699024_NP_001243985.1] | #N/A | 0.5466611 |
|  | PREDICTED: V-type proton ATPase 116 kDa subunit a isoform 1-like - [512927147_XP_004931262.1] | 1.17795894 | 0.5464808 |
|  | PREDICTED: uncharacterized protein LOC101738649 - [512893234_XP_004923131.1] | #N/A | 0.5464746 |
|  | PREDICTED: uncharacterized protein DDB_G0284459-like, partial - [512918988_XP_004929266.1] | 1.18353099 | 0.5463131 |
|  | PREDICTED: LOW QUALITY PROTEIN: proteasome activator complex subunit 3-like - [512931260_XP_004932258.1] | 1.15087072 | 0.5460318 |
|  | PREDICTED: 10 kDa heat shock protein, mitochondrial-like - [512934077_XP_004932946.1] | 1.06902747 | 0.5458371 |
|  | PREDICTED: erlin-2-like - [512928988_XP_004931715.1] | 1.14918572 | 0.5458222 |
|  | Probable 26S protease regulatory subunit 4 - [170594441_XP_001901972.1] | 1.18336829 | 0.5458119 |
|  | mitochondrial aldehyde dehydrogenase - [114052408_NP_001040475.1] | 1.12081494 | 0.5455675 |
|  | profilin - [112982865_NP_001037108.1] | 1.09064524 | 0.5453861 |
|  | vesicle amine transport protein - [153792203_NP_001093281.1] | 1.09521109 | 0.5453522 |
|  | cysteine-type peptidase - [114052835_NP_001040496.1] | 1.21752526 | 0.5452939 |
|  | PREDICTED: hydroxyacylglutathione hydrolase, mitochondrial-like - [512932433_XP_004932543.1] | 1.13332522 | 0.5452636 |
|  | PREDICTED: aldose 1-epimerase-like isoform X1 - [512891308_XP_004922660.1] | 1.11767014 | 0.5452447 |
|  | PREDICTED: calcium-binding mitochondrial carrier protein SCaMC-2-like isoform X2 - [512933657_XP_004932845.1] | #N/A | 0.5451274 |
|  | PREDICTED: spectrin beta chain-like isoform 2 - [328699230_XP_003240873.1] | 1.17262425 | 0.5450429 |
|  | PREDICTED: sulfide:quinone oxidoreductase, mitochondrial-like isoform X2 - [512932579_XP_004932580.1] | 1.03502189 | 0.5450269 |
|  | PREDICTED: developmentally-regulated GTP-binding protein 2-like - [449672355_XP_002154991.2] | 1.12998312 | 0.5447968 |
|  | snap-scaffold11144_size2552-abinit-gene-0.0-mRNA-1 | 1.14771362 | 0.5446949 |
|  | PREDICTED: putative polypeptide N-acetylgalactosaminyltransferase 9-like isoform X1 - [512897354_XP_004924132.1] | 1.13438614 | 0.5445884 |
|  | mitochondrial prohibitin complex protein 2 - [114051710_NP_001040326.1] | 1.10793745 | 0.5445414 |
|  | PREDICTED: REST corepressor 3 isoform 2 - [328785930_XP_392644.3] | 1.16034335 | 0.5444193 |
|  | PREDICTED: protein ERGIC-53-like - [512908888_XP_004926789.1] | 1.22200755 | 0.5441494 |
|  | PREDICTED: structural maintenance of chromosomes protein 5-like - [512894672_XP_004923486.1] | #N/A | 0.5441298 |
|  | PREDICTED: conserved oligomeric Golgi complex subunit 3-like - [512932058_XP_004932452.1] | 0.99906411 | 0.5440791 |
|  | ribosomal protein S10 - [112983505_NP_001037524.1] | 1.13065099 | 0.5440544 |
|  | GD11704 - [195585815_XP_002082674.1] | 1.13745924 | 0.5439675 |
|  | PREDICTED: protein polybromo-1-like - [391326863_XP_003737929.1] | 1.21044146 | 0.5439631 |
|  | PREDICTED: LOW QUALITY PROTEIN: supporter of activation of yellow protein-like - [512914733_XP_004928215.1] | 1.17204746 | 0.5438938 |
|  | PREDICTED: N-alpha-acetyltransferase 40-like - [512886985_XP_004921847.1] | 1.41074044 | 0.5436072 |
|  | PREDICTED: LOW QUALITY PROTEIN: spectrin alpha chain, non-erythrocytic 1-like - [449667307_XP_004206536.1] | 1.16818654 | 0.5435743 |
|  | PREDICTED: ran-binding protein 3-like - [512910458_XP_004927176.1] | 1.1228272 | 0.543573 |
|  | PREDICTED: putative U5 small nuclear ribonucleoprotein 200 kDa helicase-like - [512908573_XP_004926714.1] | 1.04309221 | 0.5434683 |
|  | Methylmalonyl-CoA carboxyltransferase 12S subunit, putative - [157138270_XP_001664206.1] | 1.06258626 | 0.5434074 |
|  | 60S ribosomal protein L5 - [112983276_NP_001037008.1] | 1.1270086 | 0.5432881 |
|  | GK20068, partial - [195432498_XP_002064260.1] | 1.15903395 | 0.5432066 |
|  | PREDICTED: conserved oligomeric Golgi complex subunit 3-like - [512932058_XP_004932452.1] | #N/A | 0.5432047 |
|  | PREDICTED: proteasome subunit beta type-5-like - [512890860_XP_004922586.1] | 1.17263304 | 0.5431294 |
|  | cell adhesion molecule - [170044415_XP_001849844.1] | #N/A | 0.5429954 |
|  | PREDICTED: probable cytosolic iron-sulfur protein assembly protein Ciao1-like - [512900379_XP_004924877.1] | 1.18945659 | 0.5429734 |
|  | GJ19364 - [195393580_XP_002055432.1] | 1.15609041 | 0.5429483 |
|  | glyoxylate reductase/hydroxypyruvate reductase - [114053007_NP_001040540.1] | 1.07881417 | 0.5428851 |
|  | PREDICTED: abhydrolase domain-containing protein 16A-like - [512933669_XP_004932848.1] | 1.16152786 | 0.5428204 |
|  | PREDICTED: negative elongation factor B-like - [512935973_XP_004933406.1] | 1.16256125 | 0.5423582 |
|  | short stop, isoform AC - [386767917_NP_001246312.1] | 1.07602197 | 0.5422917 |
|  | PREDICTED: l-2-hydroxyglutarate dehydrogenase, mitochondrial-like - [512919941_XP_004929507.1] | 1.11961649 | 0.5422785 |
|  | PREDICTED: protein OPI10 homolog isoform X1 - [512916773_XP_004928722.1] | 1.0458254 | 0.5422369 |
|  | PREDICTED: LDLR chaperone boca-like isoform X1 - [512930617_XP_004932103.1] | 1.11295647 | 0.5421925 |
|  | small GTP-binding protein Rab10 - [148298847_NP_001091822.1] | 1.2358763 | 0.5416951 |
|  | PREDICTED: eukaryotic initiation factor 4A-like isoform 1 - [350421455_XP_003492849.1] | 1.14840828 | 0.5415435 |
|  | cytosolic malate dehydrogenase - [114052561_NP_001040257.1] | 1.12890121 | 0.541376 |
|  | disulfide-isomerase A6 - [170041921_XP_001848695.1] | 1.17807705 | 0.5413372 |
|  | PREDICTED: 26S proteasome non-ATPase regulatory subunit 6-like - [512926210_XP_004931028.1] | 1.18948482 | 0.5412682 |
|  | nascent polypeptide associated complex protein alpha subunit - [114051461_NP_001040365.1] | 1.16233744 | 0.541125 |
|  | protein phosphatase 1 catalytic subunit - [114052575_NP_001040480.1] | 1.19501239 | 0.5409424 |
|  | PREDICTED: fatty acyl-CoA reductase 1-like - [512905704_XP_004926011.1] | #N/A | 0.5408933 |
|  | PREDICTED: protein Kr-h2-like - [512896054_XP_004923819.1] | 1.10176004 | 0.5407983 |
|  | PREDICTED: charged multivesicular body protein 6-A-like - [512932344_XP_004932523.1] | 1.10297108 | 0.5407334 |
|  | UDP-glycosyltransferase UGT41A1 precursor - [379699038_NP_001243993.1] | 1.1585895 | 0.540703 |
|  | PREDICTED: heterogeneous nuclear ribonucleoprotein K-like isoform 1 - [328702409_XP_001943097.2] | #N/A | 0.5406452 |
|  | PREDICTED: transducin beta-like protein 2-like - [512930727_XP_004932131.1] | #N/A | 0.540465 |
|  | PREDICTED: hypothetical protein LOC724922 - [328787693_XP_001120822.2] | 1.12167903 | 0.540307 |
|  | PREDICTED: AP-2 complex subunit mu-1-like isoform 1 - [156549242_XP_001606373.1] | 1.18663534 | 0.5402058 |
|  | PREDICTED: stress-induced-phosphoprotein 1-like - [512920560_XP_004929662.1] | 1.08695784 | 0.540099 |
|  | PREDICTED: myrosinase 1-like - [512896247_XP_004923866.1] | 1.25587059 | 0.5400442 |
|  | PREDICTED: hypothetical protein LOC100572873 - [328722409_XP_003247573.1] | 1.13691501 | 0.5398702 |
|  | PREDICTED: rab3 GTPase-activating protein catalytic subunit-like - [512925676_XP_004930897.1] | #N/A | 0.5398675 |
|  | PREDICTED: uncharacterized protein LOC101743552 - [512919145_XP_004929303.1] | 1.18052783 | 0.5397989 |
|  | PREDICTED: 3-ketoacyl-CoA thiolase, mitochondrial-like - [156546522_XP_001606212.1] | 1.14593843 | 0.5397982 |
|  | PREDICTED: putative inositol monophosphatase 3-like - [512897456_XP_004924157.1] | #N/A | 0.5395948 |
|  | PREDICTED: uncharacterized protein LOC101739294 - [512894385_XP_004923417.1] | #N/A | 0.5393964 |
|  | PREDICTED: histone-lysine N-methyltransferase PRDM9-like, partial - [390368084_XP_003731386.1] | #N/A | 0.5392704 |
|  | PREDICTED: ER membrane protein complex subunit 10-like isoform X1 - [512913442_XP_004927897.1] | 1.16176808 | 0.5391279 |
|  | maker-scaffold1473_size111687-snap-gene-0.27-mRNA-1 | 1.2074105 | 0.5390727 |
|  | PREDICTED: actin-like protein 6A-like - [512935663_XP_004933329.1] | 1.16878962 | 0.5387233 |
|  | geminin - [382546515_NP_001244268.1] | 1.1229504 | 0.5386834 |
|  | PREDICTED: iron/zinc purple acid phosphatase-like protein-like - [512915321_XP_004928361.1] | #N/A | 0.5386571 |
|  | PREDICTED: protein asunder-like, partial - [512912283_XP_004927617.1] | #N/A | 0.5384936 |
|  | PREDICTED: ubiquitin-conjugating enzyme E2-17 kDa-like isoform X1 - [512919737_XP_004929458.1] | 1.10752474 | 0.5384658 |
|  | PREDICTED: sel1 repeat-containing protein 1 homolog - [498954580_XP_004523688.1] | 1.18195487 | 0.5383986 |
|  | PREDICTED: delta-sarcoglycan-like - [512914135_XP_004928066.1] | #N/A | 0.5383951 |
|  | septin and tuftelin interacting protein - [148298831_NP_001091840.1] | 1.13009427 | 0.5382321 |
|  | PREDICTED: 5-formyltetrahydrofolate cyclo-ligase-like isoform X1 - [512933360_XP_004932772.1] | 1.15865391 | 0.5381231 |
|  | PREDICTED: mitochondrial import receptor subunit TOM22 homolog isoform X1 - [512931206_XP_004932244.1] | 1.16838061 | 0.5380804 |
|  | PREDICTED: dopamine N-acetyltransferase-like - [512915630_XP_004928436.1] | 1.08492015 | 0.5380234 |
|  | PREDICTED: 2-methoxy-6-polyprenyl-1,4-benzoquinol methylase, mitochondrial-like isoform X1 - [512917973_XP_004929017.1] | 1.18585875 | 0.5379397 |
|  | PREDICTED: titin-like - [512907721_XP_004926513.1] | 1.14997295 | 0.5378174 |
|  | PREDICTED: WD repeat-containing protein on Y chromosome-like - [512909473_XP_004926930.1] | #N/A | 0.5374642 |
|  | PREDICTED: WD repeat-containing protein 6-like - [512925333_XP_004930813.1] | #N/A | 0.537436 |
|  | GF11811 - [194755214_XP_001959887.1] | #N/A | 0.5373569 |
|  | 2-hydroxyphytanoyl-CoA lyase - [114051914_NP_001040193.1] | 1.06962308 | 0.5373311 |
|  | PREDICTED: LOW QUALITY PROTEIN: ruvB-like 2-like - [512889339_XP_004922252.1] | 1.14675179 | 0.5373283 |
|  | GJ24495 - [195391726_XP_002054511.1] | 1.10673913 | 0.5372864 |
|  | hypothetical protein AaeL_AAEL002910 - [157132830_XP_001662659.1] | 1.1361821 | 0.5372529 |
|  | PREDICTED: integrator complex subunit 5-like - [512898077_XP_004924310.1] | #N/A | 0.5371784 |
|  | predicted protein - [156389050_XP_001634805.1] | 1.14856232 | 0.5371296 |
|  | PREDICTED: 2',5'-phosphodiesterase 12-like - [512919043_XP_004929279.1] | #N/A | 0.5370434 |
|  | PREDICTED: probable bifunctional methylenetetrahydrofolate dehydrogenase/cyclohydrolase 2-like isoform X1 - [512908723_XP_004926751.1] | 1.11888082 | 0.5370097 |
|  | GK14704 - [195434757_XP_002065369.1] | #N/A | 0.5368975 |
|  | PREDICTED: soluble NSF attachment protein-like isoform X1 - [512893894_XP_004923294.1] | 1.03900814 | 0.5368161 |
|  | PREDICTED: putative fatty acyl-CoA reductase CG8306-like - [498924719_XP_004517505.1] | 1.12182088 | 0.5368033 |
|  | PREDICTED: poly(ADP-ribose) glycohydrolase-like - [512899809_XP_004924738.1] | 1.25708036 | 0.5366257 |
|  | PREDICTED: diphthine synthase-like isoform X1 - [512898926_XP_004924517.1] | 1.13880448 | 0.5365382 |
|  | PREDICTED: RNA polymerase-associated protein Rtf1-like - [512893366_XP_004923163.1] | #N/A | 0.5365278 |
|  | C. briggsae CBR-UNC-32 protein - [268574974_XP_002642466.1] | 1.14077741 | 0.5364699 |
|  | 3-hydroxyacyl-CoA dehydrogenase - [114050917_NP_001040414.1] | 1.03920596 | 0.5364121 |
|  | Yipf6 protein - [114051339_NP_001040374.1] | 1.24767391 | 0.5362871 |
|  | PREDICTED: hypothetical protein LOC552428 - [66506411_XP_624805.1] | 0.94762872 | 0.5361749 |
|  | PREDICTED: transmembrane protein 214-like isoform X1 - [512936231_XP_004933467.1] | 1.16267467 | 0.5361598 |
|  | PREDICTED: protein smg8-like - [512902075_XP_004925286.1] | 1.14427017 | 0.5360861 |
|  | PREDICTED: exocyst complex component 8-like - [512917404_XP_004928878.1] | 1.16926799 | 0.5360569 |
|  | PREDICTED: AP-3 complex subunit beta-2-like - [512901242_XP_004925087.1] | 1.15847325 | 0.5356645 |
|  | ADP/ATP translocase - [158631166_NP_001037072.1] | 1.13774112 | 0.5356477 |
|  | PREDICTED: translation initiation factor eIF-2B subunit delta-like - [512931876_XP_004932408.1] | 1.16394469 | 0.5356082 |
|  | PREDICTED: uncharacterized protein LOC101741453 isoform X1 - [512927349_XP_004931312.1] | 1.15462183 | 0.5354964 |
|  | PREDICTED: protein shuttle craft-like - [512888154_XP_004922042.1] | 1.26288847 | 0.5354562 |
|  | PREDICTED: THO complex subunit 3-like - [512934396_XP_004933022.1] | 1.13024547 | 0.5354425 |
|  | sensory neuron membrane protein 1 - [112984488_NP_001037186.1] | #N/A | 0.5353903 |
|  | PREDICTED: anaphase-promoting complex subunit 4-like - [512913325_XP_004927869.1] | #N/A | 0.5352943 |
|  | PREDICTED: signal recognition particle subunit SRP72-like - [512899857_XP_004924750.1] | 1.10388506 | 0.5349402 |
|  | PREDICTED: putative RNA-binding protein Luc7-like 2-like isoform X1 - [512909943_XP_004927045.1] | 1.14792352 | 0.5349248 |
|  | PREDICTED: LOW QUALITY PROTEIN: UPF0160 protein C27H6.8-like - [512904727_XP_004925768.1] | 1.11756314 | 0.5349239 |
|  | NIPSNAP protein - [114051027_NP_001040139.1] | 1.04651665 | 0.5345326 |
|  | PREDICTED: myosin-IA-like - [383863969_XP_003707452.1] | #N/A | 0.534489 |
|  | PREDICTED: grpE protein homolog, mitochondrial-like - [512907055_XP_004926348.1] | 1.1403963 | 0.5344464 |
|  | PREDICTED: copper chaperone for superoxide dismutase-like - [512888909_XP_004922164.1] | #N/A | 0.5344139 |
|  | PREDICTED: phagocyte signaling-impaired protein-like - [512918243_XP_004929085.1] | 1.17099398 | 0.5342837 |
|  | Hypothetical protein CBG17698 - [268552733_XP_002634349.1] | 1.09299954 | 0.5342517 |
|  | receptor for activated protein kinase C RACK 1 isoform 1 - [115345341_NP_001041703.1] | 1.09754548 | 0.5342301 |
|  | PREDICTED: NADP-dependent malic enzyme-like - [512903677_XP_004925678.1] | 1.14608871 | 0.5342227 |
|  | dna2/nam7 helicase family member - [256071194_XP_002571926.1] | 1.14403519 | 0.5340899 |
|  | PREDICTED: protein FAM73B-like - [512925811_XP_004930930.1] | 1.25603473 | 0.533989 |
|  | PREDICTED: endoplasmic reticulum resident protein 44-like isoform X1 - [498935116_XP_004520061.1] | 1.17872896 | 0.5339814 |
|  | PREDICTED: probable tyrosyl-DNA phosphodiesterase-like isoform X2 - [512901395_XP_004925125.1] | 1.09223529 | 0.5338592 |
|  | PREDICTED: similar to GA15808-PA - [91082623_XP_969114.1] | #N/A | 0.5338486 |
|  | PREDICTED: esterase FE4-like isoform X1 - [512910400_XP_004927162.1] | 1.1118112 | 0.5336278 |
|  | NIPSNAP protein - [114051027_NP_001040139.1] | 1.08002119 | 0.5336188 |
|  | PREDICTED: long-chain-fatty-acid--CoA ligase 4-like isoform 2 - [345489519_XP_003426154.1] | 1.12289594 | 0.5335898 |
|  | replication factor C4 - [114052591_NP_001040483.1] | 1.26169016 | 0.5334568 |
|  | PREDICTED: unconventional myosin-IXa-like - [512920992_XP_004929772.1] | 1.1167563 | 0.5333643 |
|  | serine/threonine protein kinase - [256084161_XP_002578300.1] | 1.14192101 | 0.533244 |
|  | PREDICTED: protein flightless-1-like - [512931216_XP_004932247.1] | #N/A | 0.5330691 |
|  | cuticular protein RR-2 motif 143 - [290560871_NP_001166630.1] | #N/A | 0.5329997 |
|  | PREDICTED: isovaleryl-CoA dehydrogenase, mitochondrial-like - [390363158_XP_796528.3] | 1.05839902 | 0.5329769 |
|  | PREDICTED: ATPase ASNA1 homolog - [512936051_XP_004933424.1] | 1.14765423 | 0.5329159 |
|  | PREDICTED: testis-expressed sequence 2 protein-like - [512911012_XP_004927311.1] | #N/A | 0.5328774 |
|  | Elongation factor Tu C-terminal domain containing protein - [170582798_XP_001896292.1] | 1.14879196 | 0.5327149 |
|  | snap-scaffold5025_size16535-abinit-gene-0.1-mRNA-1 | #N/A | 0.532624 |
|  | C. briggsae CBR-HIM-1 protein, partial - [268562094_XP_002646601.1] | 1.1590865 | 0.5325775 |
|  | ribosomal protein L35 - [112984164_NP_001037241.1] | 1.13977025 | 0.5325464 |
|  | PREDICTED: vacuolar protein sorting-associated protein 13C-like - [512903659_XP_004925674.1] | 1.13097053 | 0.532473 |
|  | PREDICTED: potassium/sodium hyperpolarization-activated cyclic nucleotide-gated channel 2-like - [383863027_XP_003706984.1] | #N/A | 0.5324424 |
|  | ribosomal protein S15A - [112982855_NP_001037570.1] | 1.13369117 | 0.5323769 |
|  | PREDICTED: probable cytosolic Fe-S cluster assembly factor AGAP009023-like - [328793099_XP_003251826.1] | 1.12744326 | 0.5322629 |
|  | snap-scaffold950_size50203-abinit-gene-0.5-mRNA-1 | #N/A | 0.5320302 |
|  | PREDICTED: dihydroorotate dehydrogenase (quinone), mitochondrial-like - [512897645_XP_004924203.1] | 1.15926777 | 0.5320227 |
|  | PREDICTED: similar to ATP-binding cassette transporter - [91084133_XP_969849.1] | 1.08771981 | 0.5320084 |
|  | stromal cell-derived factor 2 precursor - [114052765_NP_001040278.1] | 1.21371206 | 0.5318898 |
|  | PREDICTED: insulin-degrading enzyme-like - [512907043_XP_004926345.1] | 1.09182122 | 0.5318743 |
|  | PREDICTED: mitotic spindle assembly checkpoint protein MAD1-like - [512936023_XP_004933417.1] | 1.19027975 | 0.5318528 |
|  | PREDICTED: similar to kek1 - [91081765_XP_973226.1] | #N/A | 0.5318491 |
|  | hypothetical protein CRE_24262 - [308459496_XP_003092067.1] | 1.13496393 | 0.5316889 |
|  | GH21699 - [195028370_XP_001987049.1] | 1.17026726 | 0.5316309 |
|  | PREDICTED: putative tricarboxylate transport protein, mitochondrial-like - [512891685_XP_004922744.1] | 1.12885436 | 0.5315616 |
|  | PREDICTED: uncharacterized protein LOC101737777 isoform X1 - [512914623_XP_004928188.1] | 1.29741462 | 0.5314066 |
|  | PREDICTED: 7,8-dihydro-8-oxoguanine triphosphatase-like - [512910572_XP_004927205.1] | 1.0388405 | 0.5313646 |
|  | inosine triphosphatase - [114052993_NP_001040500.1] | 1.11575946 | 0.5311998 |
|  | PREDICTED: uncharacterized protein LOC101745200 - [512931519_XP_004932323.1] | 1.08805418 | 0.5310663 |
|  | PREDICTED: facilitated trehalose transporter Tret1-1-like - [512924528_XP_004930617.1] | #N/A | 0.5309207 |
|  | snap-scaffold1536_size103565-processed-gene-0.13-mRNA-1 | #N/A | 0.5308946 |
|  | PREDICTED: protein rogdi-like isoform X1 - [512893640_XP_004923231.1] | #N/A | 0.5307952 |
|  | PREDICTED: N-acetylserotonin O-methyltransferase-like protein-like - [512904960_XP_004925825.1] | 1.0623439 | 0.5307948 |
|  | multiple edematous wings, isoform C - [442616184_NP_001259504.1] | 1.0193306 | 0.5306596 |
|  | GJ13250 - [195376665_XP_002047113.1] | 1.1340606 | 0.5305098 |
|  | PREDICTED: U1 small nuclear ribonucleoprotein C-like - [512903627_XP_004925666.1] | 1.1129579 | 0.5304801 |
|  | PREDICTED: ribosome biogenesis protein WDR12 homolog - [512930703_XP_004932125.1] | 1.1902271 | 0.5304408 |
|  | PREDICTED: ras-related protein Rab-18-like - [512909155_XP_004926852.1] | 1.120803 | 0.5303569 |
|  | GF10367 - [194749817_XP_001957333.1] | 1.16935429 | 0.5302963 |
|  | PREDICTED: neurochondrin homolog - [512887085_XP_004921864.1] | #N/A | 0.5302623 |
|  | GF15360 - [194759999_XP_001962229.1] | 1.11529745 | 0.5302033 |
|  | PREDICTED: similar to CG1244 CG1244-PA - [91080107_XP_967072.1] | 1.17183403 | 0.5300836 |
|  | ribosomal protein S11 isoform 1 - [164420683_NP_001106708.1] | 1.12195094 | 0.5300673 |
|  | PREDICTED: glycine N-methyltransferase-like - [512907159_XP_004926374.1] | 1.02917016 | 0.5300589 |
|  | protein O-fucosyltransferase 2 precursor - [148298691_NP_001091743.1] | 1.15169559 | 0.5300473 |
|  | PREDICTED: solute carrier family 41 member 1-like - [512929104_XP_004931742.1] | #N/A | 0.5299075 |
|  | PREDICTED: negative elongation factor E-like - [512926156_XP_004931015.1] | 1.14850893 | 0.5298935 |
|  | PREDICTED: luc7-like protein 3-like isoform X3 - [512932356_XP_004932526.1] | 1.13638024 | 0.5297989 |
|  | snap-scaffold10633_size6929-processed-gene-0.0-mRNA-1 | #N/A | 0.529667 |
|  | PREDICTED: probable very-long-chain enoyl-CoA reductase art-1-like - [512922628_XP_004930162.1] | 1.15527115 | 0.5296126 |
|  | cathepsin D precursor - [112983576_NP_001037351.1] | 1.04482181 | 0.5296024 |
|  | GA15376 - [125986217_XP_001356872.1] | #N/A | 0.5294097 |
|  | cytochrome c oxidase subunit Va - [164448662_NP_001106743.1] | 1.10105736 | 0.5293918 |
|  | PREDICTED: uncharacterized protein C18orf8-like - [512897516_XP_004924172.1] | #N/A | 0.5293736 |
|  | PREDICTED: BRCA1-A complex subunit Abraxas-like - [512932864_XP_004932649.1] | #N/A | 0.5293159 |
|  | maker-scaffold3984_size35841-snap-gene-0.4-mRNA-1 | #N/A | 0.5292202 |
|  | PREDICTED: probable medium-chain specific acyl-CoA dehydrogenase, mitochondrial-like isoform X1 - [512921594_XP_004929914.1] | #N/A | 0.5291881 |
|  | PREDICTED: F-box/WD repeat-containing protein 1A-like - [512901433_XP_004925134.1] | 1.19624473 | 0.5291864 |
|  | PREDICTED: serine/threonine-protein kinase Genghis Khan-like - [512895919_XP_004923788.1] | 1.29331766 | 0.5291324 |
|  | PREDICTED: uncharacterized protein LOC101736728 - [512927207_XP_004931277.1] | #N/A | 0.5290943 |
|  | PREDICTED: phosphatidylinositol N-acetylglucosaminyltransferase subunit Q-like - [512909939_XP_004927044.1] | #N/A | 0.5289514 |
|  | chromosome associated protein D3 - [169234704_NP_001108473.1] | #N/A | 0.528868 |
|  | PREDICTED: similar to trifunctional enzyme beta subunit (tp-beta) - [91087131_XP_975238.1] | 1.13293695 | 0.5288657 |
|  | poly(A)-specific ribonuclease - [237649000_NP_001153677.1] | 1.16756936 | 0.5287538 |
|  | PREDICTED: pentatricopeptide repeat-containing protein 2, mitochondrial-like - [512889769_XP_004922368.1] | 1.11441068 | 0.528685 |
|  | PREDICTED: phosphatidylinositide phosphatase SAC1-A-like - [512906382_XP_004926182.1] | 1.14001824 | 0.5286722 |
|  | proteasome subunit beta 7 - [114053073_NP_001040536.1] | 1.13351231 | 0.5285444 |
|  | PREDICTED: similar to orf - [189242337_XP_001810078.1] | #N/A | 0.5283855 |
|  | glutathione S-transferase epsilon 6 - [169234688_NP_001108465.1] | 1.13547854 | 0.5281676 |
|  | golgi SNAP receptor complex member 2 - [114050903_NP_001040413.1] | #N/A | 0.5281064 |
|  | PREDICTED: CCR4-NOT transcription complex subunit 7-like - [512913385_XP_004927882.1] | #N/A | 0.5280358 |
|  | PREDICTED: N-alpha-acetyltransferase 35, NatC auxiliary subunit-like - [383859083_XP_003705027.1] | 1.15110635 | 0.5280343 |
|  | ras protein - [112983416_NP_001036972.1] | 1.21059182 | 0.5279734 |
|  | CG30069 - [116008309_NP_610937.4] | 1.15356279 | 0.5279409 |
|  | PREDICTED: ethanolaminephosphotransferase 1-like isoform X1 - [512890573_XP_004922536.1] | #N/A | 0.5278794 |
|  | multidrug resistance-associated protein 1 - [170037039_XP_001846368.1] | #N/A | 0.527742 |
|  | PREDICTED: similar to exportin - [189242397_XP_967037.2] | #N/A | 0.5275745 |
|  | PREDICTED: proline-rich protein PRCC-like - [512906652_XP_004926250.1] | #N/A | 0.5275119 |
|  | eukaryotic initiation factor 4B protein - [148298703_NP_001091774.1] | 1.15691235 | 0.5273147 |
|  | PREDICTED: BAG family molecular chaperone regulator 2-like isoform X1 - [512937248_XP_004933721.1] | 1.13359474 | 0.5272757 |
|  | hypothetical protein CRE_29747 - [308500051_XP_003112211.1] | #N/A | 0.5272225 |
|  | PREDICTED: solute carrier family 35 member F6-like isoform X1 - [512934985_XP_004933164.1] | 1.04678135 | 0.52711 |
|  | PREDICTED: uncharacterized protein LOC101740626 - [512891895_XP_004922796.1] | #N/A | 0.5269685 |
|  | AGAP003703-PA - [347970432_XP_313496.5] | 1.11105002 | 0.5269457 |
|  | PREDICTED: lactoylglutathione lyase-like isoform X1 - [512893382_XP_004923167.1] | 1.20840014 | 0.5267544 |
|  | PREDICTED: chromodomain-helicase-DNA-binding protein Mi-2 homolog - [345481883_XP_001605650.2] | #N/A | 0.5267178 |
|  | thioredoxin peroxidase - [114052210_NP_001040464.1] | 1.11269499 | 0.5265813 |
|  | caspase Nc - [306518668_NP_001182396.1] | 1.14211611 | 0.5264512 |
|  | ubiquitin and ribosomal protein S27a - [148298787_NP_001091826.1] | 1.13832323 | 0.5263713 |
|  | PREDICTED: LOW QUALITY PROTEIN: cell division cycle 5-like protein-like - [512926635_XP_004931135.1] | 1.17593079 | 0.5263636 |
|  | PREDICTED: dmX-like protein 2-like - [390340589_XP_790747.3] | 1.22627381 | 0.5263239 |
|  | GA25794 - [198474044_XP_002132614.1] | 1.11766746 | 0.5262342 |
|  | PREDICTED: similar to Macroglobulin complement-related CG7586-PA - [91085649_XP_970922.1] | 1.0193306 | 0.5261415 |
|  | deoxyhypusine synthase - [209571448_NP_001129357.1] | 0.99357648 | 0.5260679 |
|  | PREDICTED: actin-related protein 2/3 complex subunit 5-like protein-like - [512919157_XP_004929306.1] | 1.10798819 | 0.5260209 |
|  | PREDICTED: ATPase family AAA domain-containing protein 1-B-like isoform X1 - [512898461_XP_004924406.1] | 1.12194038 | 0.525919 |
|  | PREDICTED: uncharacterized protein LOC101741471 - [512887646_XP_004921957.1] | 1.2702907 | 0.525914 |
|  | GI21492 - [195132789_XP_002010825.1] | 1.15344027 | 0.5259077 |
|  | PREDICTED: LOW QUALITY PROTEIN: mitochondrial inner membrane protease ATP23 homolog - [512923778_XP_004930435.1] | 1.09912955 | 0.5257275 |
|  | PREDICTED: thioredoxin domain-containing protein 17-like - [512923536_XP_004930376.1] | 1.33178617 | 0.5257238 |
|  | snap-scaffold10483_size16291-abinit-gene-0.0-mRNA-1 | 1.20835446 | 0.525351 |
|  | glycerol-3-phosphate dehydrogenase isoform 1 - [260166539_NP_001091835.2] | 1.11150572 | 0.525344 |
|  | PREDICTED: proton-coupled amino acid transporter 4-like - [512935188_XP_004933213.1] | 1.08101577 | 0.5253411 |
|  | PREDICTED: LOW QUALITY PROTEIN: cullin-4B-like - [512896381_XP_004923897.1] | #N/A | 0.5253187 |
|  | PREDICTED: coronin-7-like isoform X1 - [498981006_XP_004529500.1] | 1.19589132 | 0.5252938 |
|  | GD12323 - [195591382_XP_002085420.1] | 1.09503313 | 0.5252936 |
|  | PREDICTED: dihydrolipoyllysine-residue acetyltransferase component of pyruvate dehydrogenase complex, mitochondrial-like - [350426198_XP_003494364.1] | 1.17888342 | 0.5252665 |
|  | death related ced-3/Nedd2-like protein - [168823407_NP_001108337.1] | 1.21359313 | 0.5252593 |
|  | exosc7 protein - [114052969_NP_001040497.1] | 1.13618989 | 0.5250957 |
|  | AGAP005906-PA - [158294976_XP_315936.4] | 0.5249471 |  |
|  | PREDICTED: similar to AGAP007474-PA - [189238749_XP_972434.2] | #N/A | 0.5248545 |
|  | PREDICTED: sentrin-specific protease-like - [512904964_XP_004925826.1] | 1.28630792 | 0.524774 |
|  | hypothetical protein CRE_21687 - [308450787_XP_003088427.1] | 1.1421378 | 0.5247281 |
|  | AGAP005467-PA - [158294225_XP_001237663.2] | 1.10240435 | 0.5247155 |
|  | PREDICTED: serine hydrolase-like protein 2-like - [512907689_XP_004926505.1] | #N/A | 0.5246309 |
|  | ribosomal protein S8 - [112984034_NP_001037263.1] | #N/A | 0.524399 |
|  | PREDICTED: esterase FE4-like - [512934081_XP_004932947.1] | 1.10813229 | 0.5242195 |
|  | PREDICTED: calnexin-like - [350411618_XP_003489404.1] | 1.17375 | 0.5242002 |
|  | signal recognition particle receptor alpha subunit - [170580372_XP_001895235.1] | 1.49106756 | 0.5241811 |
|  | PREDICTED: similar to thioredoxin reductase isoform 2 - [91079422_XP_975772.1] | 1.15239862 | 0.5241751 |
|  | eukaryotic initiation factor 4E-2 - [148298734_NP_001091833.1] | 1.08183925 | 0.524106 |
|  | PREDICTED: trafficking protein particle complex subunit 13-like - [512930782_XP_004932145.1] | #N/A | 0.5239312 |
|  | PREDICTED: muscle M-line assembly protein unc-89-like isoform X1 - [512895998_XP_004923808.1] | 1.09952599 | 0.5238395 |
|  | PREDICTED: mitochondrial ribonuclease P protein 1 homolog - [512922406_XP_004930112.1] | 1.11909893 | 0.5237657 |
|  | PREDICTED: FGFR1 oncogene partner 2 homolog - [512912971_XP_004927785.1] | 1.17382133 | 0.5237242 |
|  | PREDICTED: chitobiosyldiphosphodolichol beta-mannosyltransferase-like isoform X1 - [512897528_XP_004924175.1] | #N/A | 0.5236767 |
|  | AGAP004362-PA - [347971758_XP_313646.4] | 1.09519243 | 0.5235348 |
|  | PREDICTED: NADH dehydrogenase [ubiquinone] 1 subunit C2-like - [512907903_XP_004926558.1] | 1.1148328 | 0.5234683 |
|  | PREDICTED: MATH and LRR domain-containing protein PFE0570w-like - [512890299_XP_004922494.1] | 1.24757915 | 0.5232745 |
|  | PREDICTED: uncharacterized protein LOC101742365 - [512926603_XP_004931127.1] | 1.15860958 | 0.5231249 |
|  | PREDICTED: similar to esophageal cancer associated protein - [189238854_XP_971868.2] | 1.20361786 | 0.5229708 |
|  | PREDICTED: tropomodulin-like isoform X1 - [512926814_XP_004931179.1] | 1.14465588 | 0.5228245 |
|  | PREDICTED: ankyrin repeat and KH domain-containing protein 1-like - [390338417_XP_001199736.2] | 1.06166602 | 0.5226705 |
|  | PREDICTED: similar to GA11945-PA - [91079957_XP_969398.1] | 1.11606848 | 0.5226175 |
|  | pleiohomeotic - [319803027_NP_001188362.1] | #N/A | 0.5226077 |
|  | PREDICTED: LOW QUALITY PROTEIN: transcriptional regulator ATRX homolog - [512915227_XP_004928338.1] | 1.21553603 | 0.5224028 |
|  | PREDICTED: uncharacterized protein LOC101739222 - [512906600_XP_004926237.1] | 1.1227457 | 0.5223477 |
|  | survivin-1 - [379698877_NP_001243911.1] | 1.04710405 | 0.522341 |
|  | PREDICTED: zinc finger protein 830-like - [512901771_XP_004925217.1] | 1.11145515 | 0.5223085 |
|  | PREDICTED: mRNA cap guanine-N7 methyltransferase-like isoform X1 - [512892799_XP_004923021.1] | 1.16901012 | 0.5222369 |
|  | 6-phosphogluconolactonase - [148298750_NP_001091839.1] | 1.11085521 | 0.5222303 |
|  | interleukin enhancer binding factor - [114051277_NP_001040383.1] | 1.12625113 | 0.5221944 |
|  | snap-scaffold423_size138491-abinit-gene-0.12-mRNA-1 | #N/A | 0.5221662 |
|  | PREDICTED: retinol dehydrogenase 14-like - [512916411_XP_004928634.1] | 1.15649801 | 0.5221493 |
|  | GM25739 - [195328573_XP_002030989.1] | 1.10702452 | 0.522125 |
|  | GK12316 - [195442481_XP_002068983.1] | 1.17577866 | 0.5220766 |
|  | PREDICTED: periodic tryptophan protein 1 homolog isoform X1 - [512892863_XP_004923037.1] | 1.14038267 | 0.5218855 |
|  | Fanconi anemia, complementation group I - [350536799_NP_001233148.1] | 1.16200348 | 0.5218722 |
|  | ribosomal protein L32 - [148298719_NP_001091752.1] | 1.2098043 | 0.5217783 |
|  | PREDICTED: probable methylmalonate-semialdehyde dehydrogenase [acylating], mitochondrial-like - [512914963_XP_004928272.1] | 1.09980087 | 0.5215936 |
|  | PREDICTED: dual specificity mitogen-activated protein kinase kinase 4-like, partial - [512909974_XP_004927053.1] | 1.21084314 | 0.5214906 |
|  | PREDICTED: serine/threonine-protein kinase SRPK3-like - [350406251_XP_003487708.1] | #N/A | 0.5214469 |
|  | PREDICTED: similar to cleft lip and palate transmembrane protein 1 - [91081299_XP_968887.1] | 1.21642672 | 0.5212819 |
|  | PREDICTED: Fanconi anemia group J protein homolog, partial - [380021224_XP_003694471.1] | #N/A | 0.5212604 |
|  | PREDICTED: hypothetical protein - [291227595_XP_002733772.1] | 1.24036735 | 0.5212505 |
|  | PREDICTED: ATP-dependent RNA helicase SUPV3L1, mitochondrial-like - [115938145_XP_786336.2] | 1.09787607 | 0.5211468 |
|  | PREDICTED: glutathione S-transferase C-terminal domain-containing protein homolog - [512906978_XP_004926329.1] | 1.06141151 | 0.5210409 |
|  | PREDICTED: similar to thymus-specific serine protease - [91078858_XP_972061.1] | 1.08898624 | 0.5209833 |
|  | coatomer protein complex subunit epsilon - [289629222_NP_001166195.1] | 1.13005682 | 0.5209246 |
|  | PREDICTED: FAM203 family protein CG6073-like - [512889069_XP_004922191.1] | 1.1394255 | 0.520719 |
|  | PREDICTED: similar to CG5913 CG5913-PA - [91094385_XP_971123.1] | 1.20305439 | 0.5207058 |
|  | PREDICTED: tRNA (cytosine(34)-C(5))-methyltransferase-like - [512890543_XP_004922531.1] | #N/A | 0.5206474 |
|  | GH20077 - [195028752_XP_001987240.1] | 1.17826366 | 0.5206343 |
|  | PREDICTED: condensin complex subunit 1-like - [512931391_XP_004932291.1] | 1.13202424 | 0.5204676 |
|  | PREDICTED: uncharacterized protein LOC101746538 - [512927070_XP_004931242.1] | 1.06498334 | 0.5203295 |
|  | PREDICTED: UBX domain-containing protein 6-like - [512936666_XP_004933573.1] | 1.24200949 | 0.5202972 |
|  | PREDICTED: LOW QUALITY PROTEIN: solute carrier family 12 member 9-like - [512894781_XP_004923512.1] | 1.05092888 | 0.5202456 |
|  | beta-tubulin - [112983503_NP_001036887.1] | 1.13301552 | 0.5201284 |
|  | PREDICTED: serine/threonine-protein kinase minibrain-like isoform 1 - [193697462_XP_001944220.1] | 1.29362436 | 0.5201236 |
|  | PREDICTED: mRNA-decapping enzyme 1B-like isoform X1 - [512901573_XP_004925168.1] | 1.13210996 | 0.5200947 |
|  | GK13754 - [195441137_XP_002068383.1] | 1.13918587 | 0.5199067 |
|  | PREDICTED: uncharacterized protein LOC100882243 - [383850285_XP_003700726.1] | #N/A | 0.5197938 |
|  | PREDICTED: DEP domain-containing protein 5-like isoform 2 - [345481876_XP_003424475.1] | 1.26892512 | 0.5197812 |
|  | PREDICTED: PIH1 domain-containing protein 1-like - [512907998_XP_004926574.1] | 1.15034142 | 0.5197351 |
|  | PREDICTED: LIM and SH3 domain protein Lasp-like - [383859135_XP_003705052.1] | 1.16952957 | 0.5197255 |
|  | hydroxysteroid dehydrogenase - [114051868_NP_001040436.1] | 1.11021103 | 0.5195643 |
|  | PREDICTED: similar to AGAP011207-PA - [91086301_XP_973766.1] | #N/A | 0.51954 |
|  | PREDICTED: DNA-directed RNA polymerase II 16 kDa polypeptide-like isoform X1 - [512930834_XP_004932158.1] | 1.06214372 | 0.5195184 |
|  | PREDICTED: phosphoglycolate phosphatase 2-like - [512912080_XP_004927568.1] | 1.08413951 | 0.5194913 |
|  | PREDICTED: RNA polymerase II transcriptional coactivator-like isoform X1 - [512907838_XP_004926542.1] | 1.19661025 | 0.5194259 |
|  | PREDICTED: methylcrotonoyl-CoA carboxylase subunit alpha, mitochondrial-like - [512899580_XP_004924681.1] | 1.19773417 | 0.5194171 |
|  | PREDICTED: voltage-dependent anion-selective channel-like isoform X4 - [512928976_XP_004931712.1] | 1.13483846 | 0.5192503 |
|  | PREDICTED: chromodomain-helicase-DNA-binding protein 1-like - [328722605_XP_001946846.2] | 1.14359893 | 0.5192257 |
|  | PREDICTED: uncharacterized protein CG42248-like - [512915428_XP_004928387.1] | 1.11222434 | 0.5191684 |
|  | predicted protein - [156402507_XP_001639632.1] | 1.14681845 | 0.5190941 |
|  | PREDICTED: LOW QUALITY PROTEIN: vacuolar protein sorting-associated protein 33A-like - [340727968_XP_003402305.1] | #N/A | 0.5189908 |
|  | PREDICTED: multidrug resistance-associated protein 7-like - [390350570_XP_003727446.1] | #N/A | 0.5189858 |
|  | protein translation factor SUI1 homolog - [112983000_NP_001037082.1] | 1.08729424 | 0.5189752 |
|  | PREDICTED: guanylate cyclase 32E-like - [512900415_XP_004924886.1] | #N/A | 0.5189482 |
|  | PREDICTED: spindle assembly abnormal protein 6 homolog - [512930625_XP_004932105.1] | 1.09939317 | 0.5188161 |
|  | peroxisome assembly factor-2 (peroxisomal-type atpase 1), partial - [157103169_XP_001647852.1] | 1.15433049 | 0.5188043 |
|  | polyadenylate binding protein 2 - [112983360_NP_001037644.1] | 1.11181478 | 0.5187738 |
|  | PREDICTED: nuclear RNA export factor 1-like - [512927210_XP_004931278.1] | #N/A | 0.5184918 |
|  | PREDICTED: evolutionarily conserved signaling intermediate in Toll pathway, mitochondrial-like - [512932121_XP_004932467.1] | 1.17642737 | 0.5184741 |
|  | PREDICTED: cleavage stimulation factor subunit 2 tau variant isoform X1 - [512924318_XP_004930566.1] | 1.18151064 | 0.5184535 |
|  | PREDICTED: cAMP-dependent protein kinase catalytic subunit alpha-like - [512913151_XP_004927827.1] | #N/A | 0.5183565 |
|  | PREDICTED: vacuole membrane protein 1-like - [512917355_XP_004928866.1] | 1.12341794 | 0.5181423 |
|  | PREDICTED: probable medium-chain specific acyl-CoA dehydrogenase, mitochondrial-like - [350406349_XP_003487742.1] | 1.15280168 | 0.5181131 |
|  | PREDICTED: HSPC279-like - [291234228_XP_002737053.1] | 1.10435651 | 0.5180596 |
|  | PREDICTED: probable ATP-dependent RNA helicase YTHDC2-like - [340715187_XP_003396100.1] | 1.14001031 | 0.5178368 |
|  | PREDICTED: Golgi SNAP receptor complex member 1-like - [512909292_XP_004926885.1] | 1.14989081 | 0.5178071 |
|  | eukaryotic translation initiation factor 3 subunit F - [114053215_NP_001040528.1] | 1.1784773 | 0.5177866 |
|  | PREDICTED: F-box-like/WD repeat-containing protein TBL1XR1-like - [512899548_XP_004924673.1] | 1.08145959 | 0.5177637 |
|  | PREDICTED: uncharacterized protein LOC101739643 - [512913245_XP_004927849.1] | 1.21867526 | 0.5175459 |
|  | PREDICTED: alpha-methylacyl-CoA racemase-like isoform X1 - [512925885_XP_004930947.1] | 1.09556256 | 0.5174213 |
|  | PREDICTED: mitochondrial import receptor subunit TOM70-like - [340715323_XP_003396165.1] | 1.13879408 | 0.5172992 |
|  | PREDICTED: congested-like trachea protein-like - [512898561_XP_004924428.1] | 1.13898733 | 0.5170791 |
|  | PREDICTED: dihydrolipoyllysine-residue succinyltransferase component of 2-oxoglutarate dehydrogenase complex, mitochondrial-like - [328714865_XP_001944020.2] | 1.1517504 | 0.5169245 |
|  | PREDICTED: ER membrane protein complex subunit 3-like - [512932551_XP_004932573.1] | 1.15103149 | 0.5167788 |
|  | PREDICTED: alpha-endosulfine-like isoform X1 - [512906620_XP_004926242.1] | 1.07031268 | 0.516776 |
|  | PREDICTED: transmembrane protein 62-like - [350397503_XP_003484897.1] | 1.17455281 | 0.5166354 |
|  | PREDICTED: uncharacterized protein LOC100883544 - [383851927_XP_003701482.1] | 1.21929656 | 0.5166344 |
|  | PREDICTED: multidrug resistance-associated protein 4-like - [328783654_XP_397395.4] | 1.14174919 | 0.5166009 |
|  | PREDICTED: cytochrome b5-like isoform X2 - [512916749_XP_004928716.1] | 1.17711082 | 0.5165745 |
|  | GD18485 - [195572752_XP_002104359.1] | 1.16276042 | 0.516549 |
|  | hypothetical protein CRE_02939 - [308499086_XP_003111729.1] | 1.14749072 | 0.5165423 |
|  | PREDICTED: aryl-hydrocarbon-interacting protein-like 1-like - [512931240_XP_004932253.1] | #N/A | 0.5164926 |
|  | PREDICTED: probable Ufm1-specific protease 2-like - [512913206_XP_004927840.1] | 1.12039089 | 0.516454 |
|  | PREDICTED: thioredoxin domain-containing protein 15-like - [512898542_XP_004924424.1] | 1.20683392 | 0.5162146 |
|  | PREDICTED: glutaredoxin-related protein 5, mitochondrial-like isoform X1 - [512915142_XP_004928317.1] | #N/A | 0.5160761 |
|  | PREDICTED: CCR4-NOT transcription complex subunit 1-like - [328701756_XP_001948108.2] | 1.20436927 | 0.5160658 |
|  | PREDICTED: ankyrin repeat domain-containing protein 17-like - [512895085_XP_004923588.1] | 1.27925388 | 0.5159382 |
|  | GL26600 - [195161500_XP_002021606.1] | 1.18268959 | 0.5158574 |
|  | PREDICTED: similar to CG17838 CG17838-PB - [189234965_XP_973687.2] | 1.12275268 | 0.5157708 |
|  | PREDICTED: protein tyrosine phosphatase type IVA 1-like isoform X3 - [498972281_XP_004527273.1] | 1.091728 | 0.5157322 |
|  | PREDICTED: similar to AGAP008379-PA - [189236457_XP_973878.2] | 1.09356806 | 0.5155818 |
|  | PREDICTED: protein unc-13 homolog A-like - [512921020_XP_004929779.1] | #N/A | 0.5155474 |
|  | juvenile hormone epoxide hydrolase-like protein 3 - [261245099_NP_001159619.1] | 1.12802614 | 0.5154288 |
|  | PREDICTED: vacuolar protein sorting-associated protein 52 homolog - [328793258_XP_003251853.1] | #N/A | 0.5152968 |
|  | PREDICTED: chromatin assembly factor 1 subunit A-like - [499004479_XP_004535342.1] | 1.54112684 | 0.5151341 |
|  | PREDICTED: uncharacterized protein LOC101737582, partial - [512908034_XP_004926583.1] | #N/A | 0.5150922 |
|  | PREDICTED: zinc finger protein 598-like - [512887500_XP_004921935.1] | 1.17756076 | 0.5150537 |
|  | PREDICTED: uncharacterized protein LOC101735752 - [512918382_XP_004929120.1] | #N/A | 0.5149813 |
|  | AGAP007258-PA - [158285967_XP_308544.4] | 1.15001619 | 0.514915 |
|  | PREDICTED: DDB1- and CUL4-associated factor-like 1-like - [383849533_XP_003700399.1] | 1.26492086 | 0.5148355 |
|  | chitin synthase domain protein - [339239721_XP_003378777.1] | 1.16423701 | 0.5147992 |
|  | PREDICTED: eukaryotic translation initiation factor 3 subunit A-like isoform X2 - [512917662_XP_004928942.1] | 1.16868664 | 0.5147616 |
|  | PREDICTED: fasciclin-3-like - [512905684_XP_004926006.1] | 1.18562006 | 0.5147216 |
|  | PREDICTED: lariat debranching enzyme-like isoform X1 - [512928216_XP_004931526.1] | 1.08984115 | 0.5145514 |
|  | progesterone receptor membrane component 2 - [114052687_NP_001040267.1] | 1.11214603 | 0.5144329 |
|  | lethal(3)neo18 - [195963331_NP_001124377.1] | 1.20991215 | 0.5143502 |
|  | PREDICTED: Down syndrome cell adhesion molecule-like protein Dscam2-like - [512937522_XP_004933786.1] | #N/A | 0.514223 |
|  | signal peptidase complex subunit 3 - [148298671_NP_001091763.1] | 1.12602835 | 0.5141986 |
|  | PREDICTED: dynamin-like isoform X2 - [512916247_XP_004928591.1] | 1.09383095 | 0.5140554 |
|  | interleukin enhancer binding factor - [114051277_NP_001040383.1] | 1.12780552 | 0.5140358 |
|  | retinoblastoma-binding protein 5 - [170049502_XP_001856893.1] | 1.31992765 | 0.5139315 |
|  | PREDICTED: peroxisomal leader peptide-processing protease-like - [512897776_XP_004924236.1] | #N/A | 0.5139205 |
|  | GK10679 - [195425411_XP_002061002.1] | 1.13946973 | 0.513851 |
|  | PREDICTED: THO complex subunit 7 homolog isoform X1 - [512886197_XP_004921720.1] | 1.23352928 | 0.5136605 |
|  | PREDICTED: FACT complex subunit spt16-like - [512885677_XP_004921634.1] | 1.16973979 | 0.5136257 |
|  | PREDICTED: von Willebrand factor A domain-containing protein 8-like - [512910290_XP_004927135.1] | 1.13693427 | 0.5135643 |
|  | PREDICTED: LOW QUALITY PROTEIN: fumarate hydratase, mitochondrial-like - [512909069_XP_004926832.1] | 1.1368313 | 0.51355 |
|  | ATP synthase F1, beta subunit - [339242327_XP_003377089.1] | 1.13247337 | 0.5134937 |
|  | PREDICTED: uncharacterized protein LOC101738484 - [512913891_XP_004928004.1] | #N/A | 0.5134577 |
|  | peroxisomal biogenesis factor 3 - [114052659_NP_001040264.1] | 1.17741633 | 0.5133852 |
|  | glucosamine-6-phosphate N-acetyltransferase - [114052422_NP_001040128.1] | 1.12796663 | 0.5133516 |
|  | PREDICTED: ER lumen protein retaining receptor-like - [350404577_XP_003487151.1] | #N/A | 0.5132865 |
|  | PREDICTED: ras-related protein Rab-39A-like - [512924899_XP_004930706.1] | 1.14119884 | 0.5132069 |
|  | PREDICTED: dynein heavy chain 3, axonemal-like - [512912120_XP_004927578.1] | #N/A | 0.5132035 |
|  | GK11031 - [195445677_XP_002070435.1] | #N/A | 0.513079 |
|  | maker-scaffold2076_size93416-snap-gene-0.21-mRNA-1 | #N/A | 0.5129338 |
|  | PREDICTED: uncharacterized protein LOC101745663 isoform X1 - [512904839_XP_004925796.1] | #N/A | 0.5128319 |
|  | PREDICTED: ubiquitin carboxyl-terminal hydrolase 14-like - [512910665_XP_004927226.1] | 1.09536031 | 0.5127313 |
|  | PREDICTED: transmembrane 9 superfamily member 2-like - [512926299_XP_004931051.1] | 1.15055435 | 0.5126084 |
|  | PREDICTED: nuclear pore complex protein Nup155-like, partial - [512900447_XP_004924894.1] | 1.12707346 | 0.512535 |
|  | GH12185 - [195041231_XP_001991213.1] | 1.26067794 | 0.512243 |
|  | ras-related GTP-binding protein Rab3 - [112983326_NP_001037620.1] | 1.13762878 | 0.5121815 |
|  | PREDICTED: lysine-specific demethylase lid-like - [512894817_XP_004923521.1] | 1.17417863 | 0.5121294 |
|  | ribosomal protein P0 - [112982735_NP_001037123.1] | 1.12567117 | 0.5121115 |
|  | cullin - [157136359_XP_001663720.1] | 1.16943932 | 0.5119944 |
|  | glutathione S-transferase theta 1 - [169234684_NP_001108463.1] | 1.15849834 | 0.5119008 |
|  | cathepsin B precursor - [112983908_NP_001036850.1] | 1.07807352 | 0.5118555 |
|  | PREDICTED: uncharacterized protein C20orf24 homolog - [383853337_XP_003702179.1] | #N/A | 0.5118258 |
|  | PREDICTED: vacuolar protein sorting-associated protein 51 homolog - [512892352_XP_004922910.1] | #N/A | 0.5116328 |
|  | PREDICTED: mitotic spindle assembly checkpoint protein MAD2A-like - [512905315_XP_004925913.1] | 1.08952973 | 0.5115262 |
|  | mothers against dpp - [170028413_XP_001842090.1] | 1.11794066 | 0.5114741 |
|  | PREDICTED: translation initiation factor IF-3, mitochondrial-like - [512913257_XP_004927852.1] | 1.04438521 | 0.5114422 |
|  | PREDICTED: glutaryl-CoA dehydrogenase, mitochondrial-like - [512930664_XP_004932115.1] | 1.12072081 | 0.5113923 |
|  | PREDICTED: exocyst complex component 4-like - [512899992_XP_004924783.1] | 1.25595267 | 0.51132 |
|  | PREDICTED: protein TRC8 homolog - [512888562_XP_004922106.1] | 1.14346019 | 0.5112743 |
|  | PREDICTED: glyoxylate reductase/hydroxypyruvate reductase-like isoform X2 - [512898607_XP_004924438.1] | #N/A | 0.5112 |
|  | PREDICTED: AMP deaminase 2-like isoform 1 - [193610715_XP_001951813.1] | #N/A | 0.5110704 |
|  | fructose 1,6-bisphosphate aldolase - [148298685_NP_001091766.1] | 1.10385571 | 0.5110698 |
|  | PREDICTED: uncharacterized protein LOC100881023 - [383858413_XP_003704696.1] | 0.95923253 | 0.5110524 |
|  | PREDICTED: receptor-mediated endocytosis protein 6 homolog - [512917109_XP_004928806.1] | #N/A | 0.511027 |
|  | GH20583 - [195026770_XP_001986331.1] | 1.10760911 | 0.5109781 |
|  | PREDICTED: similar to acyl-coa dehydrogenase isoform 1 - [91076006_XP_966406.1] | 1.14715991 | 0.5109573 |
|  | PREDICTED: heterogeneous nuclear ribonucleoprotein U-like protein 1-like - [512893414_XP_004923175.1] | 1.11857323 | 0.5109112 |
|  | PREDICTED: dnaJ homolog subfamily C member 10-like - [512908026_XP_004926581.1] | 1.05788643 | 0.5106755 |
|  | AGAP002094-PA - [347967171_XP_320945.5] | 1.12249606 | 0.5106273 |
|  | PREDICTED: NFX1-type zinc finger-containing protein 1-like isoform 2 - [345492871_XP_003426945.1] | 1.32189092 | 0.5106243 |
|  | PREDICTED: splicing factor U2af 38 kDa subunit-like - [512907824_XP_004926538.1] | 1.09989884 | 0.5106087 |
|  | PREDICTED: serine hydroxymethyltransferase, cytosolic-like isoform X1 - [498972812_XP_004527404.1] | 1.13829902 | 0.5105498 |
|  | PREDICTED: traB domain-containing protein-like - [512929969_XP_004931954.1] | 1.20446838 | 0.5104914 |
|  | PREDICTED: pre-mRNA-splicing factor CWC25 homolog - [512925920_XP_004930955.1] | #N/A | 0.5104435 |
|  | PREDICTED: proliferation-associated protein 2G4-like - [380011026_XP_003689614.1] | 1.1285729 | 0.5104309 |
|  | PREDICTED: thioredoxin, mitochondrial-like isoform X1 - [512914421_XP_004928138.1] | 1.04683273 | 0.5103966 |
|  | PREDICTED: uncharacterized protein LOC101743677 - [512905616_XP_004925989.1] | #N/A | 0.5103005 |
|  | NADH dehydrogenase 1 alpha subcomplex subunit 5 - [153792038_NP_001093287.1] | 1.15840874 | 0.5102817 |
|  | AGAP007985-PA, partial - [158297213_XP_317481.4] | #N/A | 0.5102595 |
|  | nuclear factor NF-kappa-B p110 subunit isoform 1 - [156255214_NP_001095935.1] | 1.08509147 | 0.5099603 |
|  | PREDICTED: pseudouridine-metabolizing bifunctional protein C1861.05-like - [512916817_XP_004928733.1] | 1.18661072 | 0.5099484 |
|  | aubergine protein - [166706856_NP_001098066.2] | 1.10353298 | 0.5099145 |
|  | PREDICTED: similar to methionine aminopeptidase - [91092262_XP_967283.1] | 1.13312322 | 0.5098666 |
|  | PREDICTED: eukaryotic translation initiation factor 3 subunit M-like - [156542482_XP_001600090.1] | 1.15239603 | 0.5098482 |
|  | peroxisomal membrane anchor protein - [114052665_NP_001040266.1] | 1.21579509 | 0.5098305 |
|  | PREDICTED: neutral alpha-glucosidase AB-like isoform X3 - [512893254_XP_004923136.1] | 1.19261483 | 0.5095871 |
|  | PREDICTED: succinate dehydrogenase [ubiquinone] flavoprotein subunit, mitochondrial-like - [328704221_XP_001950339.2] | 1.15594883 | 0.5095496 |
|  | transcription elongation factor S-II - [114050769_NP_001040406.1] | 1.16993859 | 0.5094107 |
|  | PREDICTED: protein FAM114A2-like - [512891240_XP_004922649.1] | 1.10470635 | 0.5094017 |
|  | PREDICTED: LOW QUALITY PROTEIN: serrate RNA effector molecule homolog - [512922734_XP_004930189.1] | 1.09386022 | 0.5091 |
|  | PREDICTED: solute carrier family 12 member 6-like - [340372225_XP_003384645.1] | #N/A | 0.5090616 |
|  | PREDICTED: dehydrogenase/reductase SDR family member 4-like isoform X1 - [512928059_XP_004931489.1] | 1.09944045 | 0.509034 |
|  | PREDICTED: 6-phosphofructo-2-kinase/fructose-2,6-bisphosphatase-like isoform X1 - [512895397_XP_004923662.1] | 1.07167757 | 0.5088816 |
|  | Bphl-like protein - [356991242_NP_001239350.1] | 1.14585692 | 0.5088479 |
|  | PREDICTED: uncharacterized protein LOC101746985 - [512910230_XP_004927117.1] | #N/A | 0.5088208 |
|  | PREDICTED: mitochondrial 2-oxodicarboxylate carrier-like - [512914433_XP_004928141.1] | 1.08732361 | 0.5087904 |
|  | PREDICTED: adenosine deaminase-like protein-like - [512913756_XP_004927971.1] | 1.08563873 | 0.5087713 |
|  | PREDICTED: protein arginine N-methyltransferase 3-like - [512925614_XP_004930882.1] | 1.12528606 | 0.5087166 |
|  | PREDICTED: eukaryotic translation initiation factor 2D-like isoform X1 - [512896078_XP_004923825.1] | #N/A | 0.508708 |
|  | hydroxymethylglutaryl-CoA lyase - [114051864_NP_001040133.1] | 1.14617012 | 0.5085758 |
|  | PREDICTED: WD repeat-containing protein 91-like isoform X1 - [512890099_XP_004922454.1] | #N/A | 0.5084982 |
|  | PREDICTED: uncharacterized protein LOC101743552 - [512919145_XP_004929303.1] | 1.16160041 | 0.5084312 |
|  | CRE-OGT-1 protein - [308501583_XP_003112976.1] | 1.18609016 | 0.5084291 |
|  | PREDICTED: similar to cxpwmw03 - [91075966_XP_969306.1] | 1.12154954 | 0.5083931 |
|  | PREDICTED: tricarboxylate transport protein, mitochondrial-like - [328720505_XP_001951190.2] | 1.17195435 | 0.5081419 |
|  | transcription-associated protein 1 - [170055080_XP_001863421.1] | #N/A | 0.5081263 |
|  | PREDICTED: serine/threonine-protein kinase PAK 3-like - [512902932_XP_004925495.1] | 1.264828 | 0.5080958 |
|  | farnesyl diphosphate synthase 3 - [153791944_NP_001093302.1] | 1.18902714 | 0.5080923 |
|  | PREDICTED: eukaryotic translation initiation factor 3 subunit B-like isoform 2 - [345478795_XP_003423811.1] | 1.16324634 | 0.5080816 |
|  | snap-scaffold3510_size31006-abinit-gene-0.7-mRNA-1 | #N/A | 0.5080116 |
|  | PREDICTED: PDZ and LIM domain protein Zasp-like - [512907528_XP_004926465.1] | #N/A | 0.5079476 |
|  | PREDICTED: DNA polymerase alpha catalytic subunit-like - [512892731_XP_004923004.1] | #N/A | 0.507846 |
|  | PREDICTED: enoyl-CoA hydratase domain-containing protein 3, mitochondrial-like isoform X1 - [512929179_XP_004931760.1] | 1.10963939 | 0.5077066 |
|  | conserved hypothetical protein - [170043701_XP_001849514.1] | #N/A | 0.5076261 |
|  | PREDICTED: lon protease homolog, mitochondrial-like - [345485119_XP_001603638.2] | 1.14534318 | 0.5075444 |
|  | R2D2 protein - [304307735_NP_001182007.1] | 1.73085159 | 0.5074792 |
|  | PREDICTED: uncharacterized protein LOC101743028 - [512909033_XP_004926823.1] | #N/A | 0.5074332 |
|  | PREDICTED: proton-coupled amino acid transporter 4-like isoform 2 - [345494960_XP_003427405.1] | 1.02206628 | 0.5074177 |
|  | PREDICTED: histone-lysine N-methyltransferase SETD1B-like - [512918175_XP_004929068.1] | 1.19472474 | 0.5073707 |
|  | PREDICTED: serine/threonine-protein phosphatase 4 regulatory subunit 2-like - [512915131_XP_004928314.1] | 1.13577823 | 0.5072944 |
|  | PREDICTED: zinc finger protein 62-like - [512893108_XP_004923099.1] | 1.1806007 | 0.5071668 |
|  | PREDICTED: glycosylphosphatidylinositol anchor attachment 1 protein-like - [512911307_XP_004927379.1] | 1.12527121 | 0.5071622 |
|  | PREDICTED: abhydrolase domain-containing protein 3-like - [512912362_XP_004927636.1] | 1.11023962 | 0.5071226 |
|  | PREDICTED: transcription factor BTF3 homolog 4-like - [512934370_XP_004933017.1] | 1.09358741 | 0.5070407 |
|  | PREDICTED: arrestin domain-containing protein 3-like - [512920552_XP_004929660.1] | #N/A | 0.5070067 |
|  | surfeit 1 isoform 1 - [114050943_NP_001040328.1] | 1.14975439 | 0.5069798 |
|  | PREDICTED: 5'-3' exoribonuclease 1-like - [512930429_XP_004932066.1] | #N/A | 0.5068647 |
|  | PREDICTED: NAD kinase domain-containing protein 1, mitochondrial-like - [512935791_XP_004933360.1] | #N/A | 0.5067999 |
|  | PREDICTED: NADH dehydrogenase [ubiquinone] iron-sulfur protein 4, mitochondrial-like - [512889996_XP_004922426.1] | 1.09984856 | 0.5067292 |
|  | PREDICTED: cytoplasmic FMR1-interacting protein-like - [512889173_XP_004922208.1] | #N/A | 0.506723 |
|  | PREDICTED: 116 kDa U5 small nuclear ribonucleoprotein component-like isoform 1 - [340369206_XP_003383139.1] | 1.14443114 | 0.5066529 |
|  | PREDICTED: anaphase-promoting complex subunit 1-like - [512905529_XP_004925967.1] | 0.5066361 |  |
|  | PREDICTED: protein SMG8-like - [512902072_XP_004925285.1] | 1.30829775 | 0.5066337 |
|  | chloride intracellular channel isoform 1 - [114053119_NP_001040533.1] | 1.15684288 | 0.5066299 |
|  | PREDICTED: thioredoxin domain-containing protein 5-like - [512895097_XP_004923591.1] | 1.17978151 | 0.5066167 |
|  | proteasome 26S non-ATPase subunit 4 - [148298816_NP_001091810.1] | 1.14373294 | 0.5065996 |
|  | PREDICTED: enoyl-CoA delta isomerase 2, mitochondrial-like - [512928119_XP_004931504.1] | 1.0868092 | 0.5065343 |
|  | PREDICTED: structural maintenance of chromosomes protein 6-like - [512906755_XP_004926273.1] | 1.29721666 | 0.5063155 |
|  | PREDICTED: esterase FE4-like - [512899307_XP_004924611.1] | 1.03421842 | 0.506264 |
|  | PREDICTED: MAP kinase-activated protein kinase 2-like - [512925510_XP_004930857.1] | 1.13913872 | 0.5061705 |
|  | PREDICTED: dolichyl-diphosphooligosaccharide--protein glycosyltransferase subunit 1-like - [512931880_XP_004932409.1] | 1.12476296 | 0.5058417 |
|  | PREDICTED: uncharacterized protein LOC101745073 - [512898882_XP_004924506.1] | #N/A | 0.5058282 |
|  | PREDICTED: probable splicing factor, arginine/serine-rich 7-like - [512936098_XP_004933436.1] | 1.14636725 | 0.5058271 |
|  | serine protease inhibitor 10 precursor - [226342882_NP_001139703.1] | #N/A | 0.5057477 |
|  | PREDICTED: chromosome transmission fidelity protein 18 homolog - [512906829_XP_004926291.1] | #N/A | 0.5057045 |
|  | importin alpha 34 - [256080295_XP_002576417.1] | 1.15126338 | 0.5056701 |
|  | PREDICTED: transmembrane protein 214-like isoform X1 - [512936231_XP_004933467.1] | 1.14124576 | 0.5056562 |
|  | ribosomal protein S20 - [148298732_NP_001091809.1] | 1.12169714 | 0.5055805 |
|  | cdk10/11-like protein - [282165750_NP_001164116.1] | #N/A | 0.5054672 |
|  | PREDICTED: LOW QUALITY PROTEIN: probable trans-2-enoyl-CoA reductase, mitochondrial-like - [512929583_XP_004931857.1] | 1.12993276 | 0.5053905 |
|  | coatomer protein complex subunit alpha - [289629216_NP_001166192.1] | 1.09563487 | 0.5053469 |
|  | PREDICTED: short spindle protein 4-like - [512925578_XP_004930873.1] | 1.10475999 | 0.5052703 |
|  | PREDICTED: ras-like GTP-binding protein Rho1-like isoform X2 - [512936173_XP_004933454.1] | 1.16118258 | 0.5051246 |
|  | uncharacterized protein LOC100216501 - [218505761_NP_001136226.1] | 1.11093353 | 0.5051108 |
|  | PREDICTED: MIP18 family protein CG7949-like - [512888956_XP_004922172.1] | #N/A | 0.5051029 |
|  | PREDICTED: hypothetical protein LOC409093 - [328791009_XP_392619.4] | 1.17437722 | 0.5049959 |
|  | PREDICTED: ribosome-binding protein 1-like isoform X3 - [512898977_XP_004924530.1] | 1.16565426 | 0.5049497 |
|  | PREDICTED: uncharacterized protein C31H12.03c-like - [512927404_XP_004931326.1] | 1.16330142 | 0.5049489 |
|  | snap-scaffold7576_size38005-abinit-gene-0.1-mRNA-1 | #N/A | 0.5049302 |
|  | PREDICTED: uncharacterized oxidoreductase C24B10.20-like - [512885385_XP_004921586.1] | 1.08692706 | 0.5047261 |
|  | PREDICTED: LOW QUALITY PROTEIN: dynein heavy chain 8, axonemal-like - [512923223_XP_004930301.1] | #N/A | 0.5047185 |
|  | PREDICTED: serine-rich adhesin for platelets-like isoform X7 - [499002359_XP_004534818.1] | 1.13765753 | 0.5045974 |
|  | ribosomal protein S17 - [112984008_NP_001037267.1] | 1.1504745 | 0.5045904 |
|  | PREDICTED: double-strand-break repair protein rad21 homolog - [512924626_XP_004930640.1] | 1.15617476 | 0.5045868 |
|  | PREDICTED: vesicle transport protein SEC20-like isoform X1 - [512885732_XP_004921644.1] | 1.07896988 | 0.5045748 |
|  | PREDICTED: succinyl-CoA:3-ketoacid-coenzyme A transferase 1, mitochondrial-like - [340726810_XP_003401746.1] | 1.11997537 | 0.5045157 |
|  | PREDICTED: ATP-dependent RNA helicase WM6-like - [156545225_XP_001606735.1] | #N/A | 0.5045083 |
|  | yellow-e precursor - [261245091_NP_001159615.1] | 1.4807995 | 0.5045004 |
|  | PREDICTED: 4-aminobutyrate aminotransferase, mitochondrial-like - [350420895_XP_003492665.1] | 1.09533384 | 0.5044467 |
|  | PREDICTED: delta-1-pyrroline-5-carboxylate dehydrogenase, mitochondrial-like - [512916781_XP_004928724.1] | 1.14466812 | 0.5043454 |
|  | PREDICTED: probable RNA-binding protein 46-like - [512898155_XP_004924330.1] | 1.04969536 | 0.5043172 |
|  | PREDICTED: transmembrane protein 256 homolog isoform X1 - [512899358_XP_004924624.1] | 1.16379017 | 0.5041631 |
|  | PREDICTED: uncharacterized protein C9orf114 homolog isoform X1 - [512926567_XP_004931118.1] | 1.21234049 | 0.5041449 |
|  | Calcium-transporting ATPase sarcoplasmic/endoplasmic reticulum type - [170582704_XP_001896249.1] | 1.17914603 | 0.5041301 |
|  | electron-transfer-flavoprotein beta polypeptide - [114053151_NP_001040123.1] | 1.10848229 | 0.5041181 |
|  | PREDICTED: probable enoyl-CoA hydratase, mitochondrial-like - [512890854_XP_004922585.1] | 1.15681825 | 0.5040593 |
|  | PREDICTED: diphosphomevalonate decarboxylase-like - [340716987_XP_003396971.1] | 1.08566488 | 0.5038938 |
|  | dihydrolipoamide acetyltransferase component of pyruvate dehydrogenase - [157105359_XP_001648832.1] | 1.12586855 | 0.5038875 |
|  | ribosomal protein L8 - [112983487_NP_001037141.1] | 1.12011109 | 0.503881 |
|  | PREDICTED: LOW QUALITY PROTEIN: axin-like - [512932571_XP_004932578.1] | #N/A | 0.5037949 |
|  | PREDICTED: exocyst complex component 2-like - [512893013_XP_004923076.1] | 1.19375022 | 0.5037189 |
|  | PREDICTED: vitamin K epoxide reductase complex subunit 1-like protein 1-like - [512930652_XP_004932112.1] | 1.1147474 | 0.5036636 |
|  | PREDICTED: perilipin-4-like isoform X1 - [512910256_XP_004927124.1] | 1.16177688 | 0.5036635 |
|  | PREDICTED: U5 small nuclear ribonucleoprotein 40 kDa protein-like - [512896582_XP_004923945.1] | 1.1531419 | 0.5034085 |
|  | PREDICTED: vesicle-associated membrane protein/synaptobrevin-binding protein-like isoform X1 - [512921529_XP_004929900.1] | 1.1426924 | 0.503185 |
|  | snap-scaffold4060_size27624-abinit-gene-0.3-mRNA-1 | #N/A | 0.5031805 |
|  | PREDICTED: seipin-like isoform X2 - [512893274_XP_004923141.1] | 1.12435515 | 0.5030658 |
|  | PREDICTED: nucleoporin-like protein amo1-like - [512896578_XP_004923944.1] | 1.00915679 | 0.5030177 |
|  | PREDICTED: MAP/microtubule affinity-regulating kinase 3-like - [345481101_XP_003424287.1] | 1.13274746 | 0.5029861 |
|  | PREDICTED: hsc70-interacting protein-like isoform X1 - [512910645_XP_004927221.1] | 1.12297122 | 0.5027846 |
|  | snap-scaffold761_size62764-abinit-gene-0.5-mRNA-1 | 1.17995863 | 0.5027833 |
|  | PREDICTED: similar to conserved hypothetical protein - [91086043_XP_973506.1] | 1.21519429 | 0.5027639 |
|  | PREDICTED: LOW QUALITY PROTEIN: ER membrane protein complex subunit 4-like - [512926008_XP_004930977.1] | 1.20541083 | 0.502604 |
|  | kakapo - [170033490_XP_001844610.1] | 1.05382801 | 0.5024332 |
|  | hypothetical protein AaeL_AAEL004355 - [157105718_XP_001648995.1] | #N/A | 0.5024252 |
|  | PREDICTED: DNA mismatch repair protein Msh2-like - [449663119_XP_002154213.2] | 1.12889959 | 0.5023855 |
|  | snap-scaffold2830_size54092-abinit-gene-0.0-mRNA-1 | #N/A | 0.5023534 |
|  | PREDICTED: CG12264-like - [291238258_XP_002739045.1] | 1.10567888 | 0.502345 |
|  | PREDICTED: transmembrane protein 214-like isoform X1 - [512936231_XP_004933467.1] | 1.16289633 | 0.502329 |
|  | PREDICTED: hyccin-like - [512900248_XP_004924845.1] | 1.22190539 | 0.5023157 |
|  | PREDICTED: uncharacterized protein LOC101744580 - [512930586_XP_004932096.1] | 1.18256252 | 0.5021632 |
|  | PREDICTED: double-strand-break repair protein rad21 homolog - [512924626_XP_004930640.1] | 1.12604056 | 0.5021121 |
|  | heterogeneous nuclear ribonucleoprotein A1 - [153792009_NP_001093319.1] | 1.03335613 | 0.5020826 |
|  | PREDICTED: phosphatidylinositol 4-kinase type 2-beta-like - [512910218_XP_004927114.1] | #N/A | 0.5018576 |
|  | PREDICTED: prostatic acid phosphatase-like - [383865693_XP_003708307.1] | 1.1427334 | 0.5018061 |
|  | PREDICTED: serine/threonine-protein kinase svkA-like - [512919359_XP_004929356.1] | #N/A | 0.501799 |
|  | ribosomal protein S3 - [112984112_NP_001037253.1] | 1.13123366 | 0.5017263 |
|  | ribosomal protein L21 - [112984298_NP_001037223.1] | 1.14995482 | 0.5017057 |
|  | PREDICTED: alpha-1,2-mannosyltransferase ALG9-like - [340718452_XP_003397681.1] | #N/A | 0.5015409 |
|  | PREDICTED: LOW QUALITY PROTEIN: polycomb protein Sfmbt - [345488218_XP_003425859.1] | #N/A | 0.501531 |
|  | PREDICTED: motile sperm domain-containing protein 2-like - [512897615_XP_004924196.1] | 1.11979865 | 0.5014612 |
|  | PREDICTED: cytoplasmic aconitate hydratase-like - [512888904_XP_004922163.1] | 1.08171675 | 0.5014404 |
|  | PREDICTED: choline/ethanolaminephosphotransferase 1-like - [383847999_XP_003699640.1] | #N/A | 0.5013927 |
|  | PREDICTED: Golgi phosphoprotein 3-like isoform X1 - [512922639_XP_004930165.1] | 1.26027589 | 0.5011629 |
|  | clathrin heavy chain - [219362829_NP_001136443.1] | 1.14146766 | 0.5010764 |
|  | Protein ALG-1, isoform a - [392927851_NP_001257238.1] | 1.14324204 | 0.5010246 |
|  | PREDICTED: ferrochelatase, mitochondrial-like - [512889656_XP_004922337.1] | 1.14413913 | 0.5008055 |
|  | PREDICTED: endoplasmic reticulum lectin 1-like - [512919691_XP_004929447.1] | 1.2144377 | 0.5007619 |
|  | AGAP003405-PA - [347969836_XP_311692.5] | 1.02053047 | 0.5007177 |
|  | PREDICTED: protein kinase C and casein kinase substrate in neurons protein 2-like isoform 1 - [345493388_XP_001605519.2] | #N/A | 0.500675 |
|  | PREDICTED: nuclear pore complex protein Nup214-like - [512932125_XP_004932468.1] | 1.13020586 | 0.5006309 |
|  | PREDICTED: LOW QUALITY PROTEIN: phosphatidylinositol-binding clathrin assembly protein LAP-like - [512893989_XP_004923321.1] | 1.06467594 | 0.50014 |
|  | PREDICTED: immunoglobulin superfamily DCC subclass member 4-like - [512908208_XP_004926625.1] | #N/A | 0.5001303 |
|  | CRE-EMB-8 protein - [308461935_XP_003093255.1] | 1.10455828 | 0.500097 |
|  | PREDICTED: uncharacterized protein LOC101738134 - [512921346_XP_004929854.1] | 1.17459613 | 0.5000845 |
|  | inosine-5'-monophosphate dehydrogenase family protein - [170585846_XP_001897693.1] | 1.17421851 | 0.5000812 |
|  | PREDICTED: MOXD1 homolog 1-like, partial - [512937654_XP_004933817.1] | 1.43478364 | 0.4999172 |
|  | PREDICTED: vesicle-trafficking protein SEC22b-B-like - [512916697_XP_004928703.1] | 1.17861155 | 0.4998725 |
|  | PREDICTED: CAD protein-like isoform X3 - [524885963_XP_005099582.1] | 1.13466092 | 0.4998403 |
|  | PREDICTED: charged multivesicular body protein 5-like - [512926615_XP_004931130.1] | 1.10129782 | 0.4997279 |
|  | acetylglucosaminyltransferase - [350536319_NP_001233218.1] | 1.18607415 | 0.4997253 |
|  | PREDICTED: p21-activated protein kinase-interacting protein 1-like - [512934109_XP_004932954.1] | 1.49325842 | 0.4996448 |
|  | PREDICTED: elongator complex protein 5-like - [512917602_XP_004928927.1] | 1.1618828 | 0.4996264 |
|  | PREDICTED: glucose-fructose oxidoreductase domain-containing protein 1-like - [512934073_XP_004932945.1] | 1.31201284 | 0.4996156 |
|  | eukaryotic translation initiation factor 3 subunit G - [112982970_NP_001037586.1] | 1.12745848 | 0.4995468 |
|  | PREDICTED: BUD13 homolog - [512893156_XP_004923111.1] | 1.10962417 | 0.499485 |
|  | mitochondrial matrix protein p33 - [114052587_NP_001040258.1] | 1.1122811 | 0.4993135 |
|  | PREDICTED: metaxin-1-like - [512937369_XP_004933751.1] | 1.13081473 | 0.4993124 |
|  | Apaf-1 protein - [317108135_NP_001186937.1] | #N/A | 0.4991463 |
|  | AGAP002809-PA - [347968597_XP_312104.5] | 1.09435585 | 0.4991399 |
|  | PREDICTED: melanotransferrin-like - [512912319_XP_004927626.1] | 0.4991036 |  |
|  | PREDICTED: conserved oligomeric Golgi complex subunit 2-like - [512886722_XP_004921806.1] | 1.05426898 | 0.4987527 |
|  | AGAP004440-PC - [347971897_XP_003436812.1] | 1.20797173 | 0.4987367 |
|  | PREDICTED: CDP-diacylglycerol--inositol 3-phosphatidyltransferase-like - [383860604_XP_003705779.1] | #N/A | 0.498612 |
|  | PREDICTED: CDGSH iron-sulfur domain-containing protein 2 homolog isoform X1 - [512926682_XP_004931147.1] | 1.10202341 | 0.4984442 |
|  | PREDICTED: uridine phosphorylase 1-like isoform X2 - [512894464_XP_004923437.1] | 1.05112172 | 0.4984345 |
|  | PREDICTED: cadherin-89D-like - [498951849_XP_004523242.1] | #N/A | 0.4983201 |
|  | GA19445 - [198472302_XP_001355895.2] | 1.22661926 | 0.4979426 |
|  | dihydrolipoamide dehydrogenase - [112983096_NP_001037054.1] | 1.17896272 | 0.4978223 |
|  | PREDICTED: similar to acetyl-CoA acetyltransferase, mitochondrial - [189234785_XP_975008.2] | 1.12975315 | 0.4977326 |
|  | PREDICTED: long-chain fatty acid transport protein 4-like - [383847649_XP_003699465.1] | 1.1093721 | 0.4976843 |
|  | CRE-TAG-321 protein - [308492786_XP_003108583.1] | #N/A | 0.4976329 |
|  | eukaryotic translation initiation factor 3 subunit H - [112983906_NP_001036848.1] | 1.18694407 | 0.4976245 |
|  | GI15496 - [195114314_XP_002001712.1] | 1.14979245 | 0.4976155 |
|  | PREDICTED: AP-3 complex subunit beta-2-like - [512901242_XP_004925087.1] | 1.1544383 | 0.4976071 |
|  | PREDICTED: ATPase family AAA domain-containing protein 1-B-like isoform X2 - [512898465_XP_004924407.1] | 1.11769866 | 0.4974781 |
|  | autophagy related protein Atg3-like protein - [215820604_NP_001135961.1] | 1.14531229 | 0.4973373 |
|  | snap-scaffold8440_size11166-abinit-gene-0.2-mRNA-1 | #N/A | 0.4973067 |
|  | PREDICTED: CAAX prenyl protease 1 homolog - [512886363_XP_004921744.1] | 1.08478089 | 0.497185 |
|  | PREDICTED: translocating chain-associated membrane protein 1-like isoform X1 - [512922710_XP_004930183.1] | 1.12959704 | 0.4971314 |
|  | heat shock 70 kD protein cognate precursor - [112984012_NP_001036837.1] | 1.2294182 | 0.4971127 |
|  | ATP synthase - [114052262_NP_001040232.1] | 1.12494529 | 0.4971068 |
|  | maker-scaffold7349_size12930-snap-gene-0.7-mRNA-1 | 1.38517157 | 0.4970681 |
|  | PREDICTED: uncharacterized protein LOC101738379 - [512894621_XP_004923474.1] | 0.99095623 | 0.4970418 |
|  | C. briggsae CBR-PAT-3 protein - [268564043_XP_002647075.1] | 1.19649795 | 0.4969721 |
|  | snap-scaffold9804_size7052-abinit-gene-0.0-mRNA-1 | 1.39398 | 0.4968977 |
|  | PREDICTED: UPF0568 protein C14orf166 homolog - [512937960_XP_004933891.1] | 1.22455405 | 0.4968712 |
|  | diptheria toxin resistance protein - [114051592_NP_001040357.1] | 1.18395175 | 0.4966601 |
|  | maker-scaffold159_size209907-snap-gene-1.30-mRNA-1 | 1.12950017 | 0.4965876 |
|  | PREDICTED: TBC1 domain family member 5-like - [512892546_XP_004922958.1] | #N/A | 0.4964039 |
|  | PREDICTED: LOW QUALITY PROTEIN: protein 4.1 homolog - [512905888_XP_004926058.1] | 1.07083526 | 0.4962902 |
|  | PREDICTED: protein farnesyltransferase/geranylgeranyltransferase type-1 subunit alpha - [512932797_XP_004932633.1] | 1.09383667 | 0.4962008 |
|  | PREDICTED: cdc42 homolog - [512934827_XP_004933126.1] | 1.13103894 | 0.4961487 |
|  | protein tyrosine phosphatase - [350536615_NP_001232932.1] | 1.10198093 | 0.496076 |
|  | PREDICTED: uncharacterized protein LOC101738779 - [512889248_XP_004922228.1] | 1.18341624 | 0.4960331 |
|  | PREDICTED: ER membrane protein complex subunit 7-like - [512897788_XP_004924239.1] | 1.28495958 | 0.4960088 |
|  | maker-scaffold2466_size62115-snap-gene-0.11-mRNA-1 | 1.04697587 | 0.4959522 |
|  | PREDICTED: cullin-1-like - [340724050_XP_003400398.1] | #N/A | 0.4958858 |
|  | PREDICTED: BTB/POZ and MATH domain-containing protein 2-like isoform X1 - [512916518_XP_004928660.1] | 1.13058251 | 0.4958791 |
|  | PREDICTED: protein ERGIC-53-like - [512908888_XP_004926789.1] | 1.1523689 | 0.4958673 |
|  | PREDICTED: probable 4-coumarate--CoA ligase 3-like - [512915254_XP_004928344.1] | 1.17056793 | 0.4958642 |
|  | PREDICTED: myosin heavy chain, non-muscle isoform 2 - [328710646_XP_003244321.1] | 1.09426123 | 0.4955798 |
|  | PREDICTED: centrosomin-like isoform X1 - [512918968_XP_004929261.1] | 0.92694764 | 0.4954918 |
|  | PREDICTED: shootin-1-like - [512934336_XP_004933010.1] | #N/A | 0.4954624 |
|  | PREDICTED: frataxin homolog, mitochondrial-like - [512892245_XP_004922884.1] | #N/A | 0.4953189 |
|  | PREDICTED: SH3 domain-containing kinase-binding protein 1-like - [512926546_XP_004931112.1] | 1.27582637 | 0.4952214 |
|  | PREDICTED: ATP-binding cassette sub-family B member 6, mitochondrial-like - [340378098_XP_003387565.1] | 1.1365581 | 0.4951822 |
|  | glutathione S-transferase epsilon 4 - [169234678_NP_001108460.1] | #N/A | 0.4951154 |
|  | PREDICTED: splicing factor 3A subunit 1-like isoform 3 - [340712930_XP_003395005.1] | 1.09608182 | 0.4949754 |
|  | PREDICTED: ubiquinone biosynthesis monooxygenase COQ6-like - [512887444_XP_004921926.1] | 1.11922456 | 0.4948762 |
|  | GI19399 - [195120560_XP_002004792.1] | 1.07508629 | 0.4947358 |
|  | PREDICTED: similar to CG7028 CG7028-PA - [189241893_XP_969149.2] | 1.11877763 | 0.49472 |
|  | PREDICTED: LOW QUALITY PROTEIN: protein 4.1 homolog - [512905888_XP_004926058.1] | 1.08243292 | 0.4945582 |
|  | PREDICTED: putative leucine-rich repeat-containing protein DDB_G0290503-like - [512919463_XP_004929392.1] | #N/A | 0.4944596 |
|  | maker-scaffold435_size155218-snap-gene-0.39-mRNA-1 | 1.24663355 | 0.4943544 |
|  | PREDICTED: laminin subunit alpha-like - [498959881_XP_004524546.1] | 0.97861312 | 0.4942606 |
|  | PREDICTED: ras-related protein Rab-35-like - [512917246_XP_004928841.1] | 1.16269071 | 0.4942518 |
|  | PREDICTED: serine/threonine-protein kinase polo-like - [512893001_XP_004923073.1] | #N/A | 0.4942482 |
|  | splicing factor 3a - [256070991_XP_002571825.1] | 1.05920105 | 0.4942313 |
|  | PREDICTED: rab5 GDP/GTP exchange factor-like - [512885323_XP_004921576.1] | 1.12366312 | 0.4942267 |
|  | CRE-ABCF-3 protein - [308498808_XP_003111590.1] | 1.16692178 | 0.4940208 |
|  | PREDICTED: diphthine--ammonia ligase-like isoform X1 - [512914725_XP_004928213.1] | #N/A | 0.4940142 |
|  | GK12344 - [195442583_XP_002069032.1] | 1.21696021 | 0.4939422 |
|  | PREDICTED: nucleoporin NUP53-like isoform X1 - [512900068_XP_004924802.1] | 1.08808358 | 0.4939371 |
|  | ribosomal protein S2 - [112982669_NP_001037564.1] | 1.05937835 | 0.4938984 |
|  | PREDICTED: LOW QUALITY PROTEIN: neuroblastoma-amplified sequence-like - [512906758_XP_004926274.1] | #N/A | 0.4937242 |
|  | isochorismatase domain containing protein - [114052855_NP_001040541.1] | 1.06501948 | 0.4934581 |
|  | PREDICTED: hypothetical protein LOC100123605 - [345495352_XP_001607269.2] | 1.03593921 | 0.4934567 |
|  | PREDICTED: protein RER1-like isoform X1 - [512900169_XP_004924826.1] | 1.21744376 | 0.4933703 |
|  | ubiquinol-cytochrome c reductase - [164448652_NP_001106738.1] | 1.14641609 | 0.4932707 |
|  | PREDICTED: probable arginine--tRNA ligase, mitochondrial-like - [512913783_XP_004927977.1] | 1.18657965 | 0.4932262 |
|  | PREDICTED: uncharacterized protein LOC101741884, partial - [512931248_XP_004932255.1] | 1.21812413 | 0.4932033 |
|  | PREDICTED: nucleolin-like isoform X1 - [512916669_XP_004928696.1] | 1.16428669 | 0.4931762 |
|  | PREDICTED: Actin, cytoplasmic-like isoform 1 - [291244281_XP_002742029.1] | 1.11875905 | 0.4931102 |
|  | AGAP007388-PA - [158285754_XP_308444.4] | 1.17454877 | 0.4930223 |
|  | PREDICTED: enhancer of rudimentary homolog - [512925027_XP_004930738.1] | 1.14400058 | 0.4928122 |
|  | PREDICTED: similar to TH1 CG9984-PA - [91078898_XP_973390.1] | #N/A | 0.492808 |
|  | peptidylprolyl isomerase - [114052358_NP_001040520.1] | 0.97308264 | 0.4926899 |
|  | PREDICTED: tumor suppressor candidate 3-like - [512914377_XP_004928127.1] | 1.15773846 | 0.4924702 |
|  | PREDICTED: nitrilase and fragile histidine triad fusion protein NitFhit-like - [512925202_XP_004930781.1] | 1.15062877 | 0.492362 |
|  | maker-scaffold4479_size25298-snap-gene-0.15-mRNA-1 | 1.1761797 | 0.4921734 |
|  | heterochromatin protein 1beta-like protein - [261245093_NP_001159616.1] | 1.13220532 | 0.4921423 |
|  | cyclin dependent kinase 5 - [274318357_NP_001162053.1] | 0.9738305 | 0.4921147 |
|  | GJ15299 - [195400663_XP_002058935.1] | #N/A | 0.4918564 |
|  | PREDICTED: ftsJ methyltransferase domain-containing protein 1 homolog isoform X1 - [512916643_XP_004928689.1] | 1.13862271 | 0.4918381 |
|  | PREDICTED: similar to GA14517-PA - [91091934_XP_967645.1] | 1.07533498 | 0.4913971 |
|  | small nuclear ribonucleoprotein sm d2 - [134948671_NP_001077096.1] | 1.11956327 | 0.4913718 |
|  | PREDICTED: NADH dehydrogenase [ubiquinone] 1 alpha subcomplex subunit 8-like - [512935361_XP_004933256.1] | 1.09272629 | 0.491363 |
|  | PREDICTED: helicase domino-like isoform X2 - [524900276_XP_005106559.1] | #N/A | 0.4913521 |
|  | PREDICTED: peptidyl-prolyl cis-trans isomerase E-like isoform X1 - [512892819_XP_004923026.1] | 0.91213048 | 0.4912531 |
|  | PREDICTED: pyruvate dehydrogenase E1 component subunit beta, mitochondrial-like - [328714666_XP_001948556.2] | 1.16241526 | 0.4910336 |
|  | vacuolar ATP synthase 21 kDa proteolipid subunit - [114051648_NP_001040169.1] | 1.17887735 | 0.4910012 |
|  | small nuclear ribonucleoprotein polypeptide - [148298654_NP_001091757.1] | 1.09620818 | 0.4909148 |
|  | ribosomal protein S19 - [112983980_NP_001037271.1] | 1.09484705 | 0.4904676 |
|  | CRAG protein - [170061053_XP_001866071.1] | 1.14928445 | 0.4903391 |
|  | isocitrate dehydrogenase - [157134807_XP_001656452.1] | 1.15911742 | 0.4901767 |
|  | transmembrane protein precursor - [114052128_NP_001040219.1] | 1.17463304 | 0.4901522 |
|  | PREDICTED: thioredoxin-related transmembrane protein 1-like - [512895915_XP_004923787.1] | 1.16004527 | 0.4900389 |
|  | UDP-glycosyltransferase UGT33N1 precursor - [379698974_NP_001243959.1] | 1.12877679 | 0.489866 |
|  | PREDICTED: cytochrome c oxidase assembly protein COX15 homolog isoform X1 - [512910321_XP_004927143.1] | 1.15106409 | 0.4898545 |
|  | PREDICTED: DNA topoisomerase 2-binding protein 1-like - [512893144_XP_004923108.1] | #N/A | 0.4897939 |
|  | maker-scaffold569_size109293-snap-gene-0.21-mRNA-1 | #N/A | 0.4897034 |
|  | PREDICTED: histone chaperone asf1-like - [512889351_XP_004922255.1] | 1.27618139 | 0.4894479 |
|  | PREDICTED: ribosomal protein L4-like - [291222349_XP_002731171.1] | 1.13869595 | 0.4893852 |
|  | PREDICTED: LOW QUALITY PROTEIN: hepatocyte growth factor-regulated tyrosine kinase substrate-like - [512932173_XP_004932480.1] | 1.23556855 | 0.4893646 |
|  | glucosamine-6-phosphate N-acetyltransferase - [114052422_NP_001040128.1] | 1.09941163 | 0.48934 |
|  | myosin vi, partial - [157105157_XP_001648742.1] | #N/A | 0.4893032 |
|  | presenilin-like signal peptide peptidase - [114051566_NP_001040306.1] | 1.20285282 | 0.489302 |
|  | H+ transporting ATP synthase O subunit isoform 1 - [114053277_NP_001040526.1] | 1.09180033 | 0.4892866 |
|  | maker-scaffold2552_size56143-snap-gene-0.19-mRNA-1 | #N/A | 0.489201 |
|  | PREDICTED: uncharacterized protein LOC101738950 - [512929885_XP_004931933.1] | 1.3261112 | 0.4891968 |
|  | PREDICTED: digestive organ expansion factor homolog - [340714280_XP_003395658.1] | 1.19101581 | 0.4891794 |
|  | PREDICTED: long-chain-fatty-acid--CoA ligase 5-like - [512929388_XP_004931808.1] | #N/A | 0.4890548 |
|  | eukaryotic translation initiation factor 5 - [112983206_NP_001037662.1] | 1.12016064 | 0.4889765 |
|  | predicted protein - [156364893_XP_001626578.1] | #N/A | 0.4889696 |
|  | PREDICTED: LOW QUALITY PROTEIN: thymidylate kinase-like - [512887735_XP_004921972.1] | 1.0891285 | 0.4888385 |
|  | PREDICTED: alpha-aminoadipic semialdehyde dehydrogenase-like isoform 1 - [391329060_XP_003738995.1] | 1.15499592 | 0.4888208 |
|  | AP-3 complex subunit delta-1 - [339258344_XP_003369358.1] | 1.08937945 | 0.4888148 |
|  | PREDICTED: extended synaptotagmin-2-A-like - [512899682_XP_004924706.1] | 1.14526061 | 0.4887673 |
|  | Pre-mRNA-splicing factor 8-like protein - [339242957_XP_003377404.1] | 1.10059357 | 0.4887296 |
|  | PREDICTED: calcium-binding mitochondrial carrier protein Aralar1-like - [383851350_XP_003701196.1] | 1.14568334 | 0.4886555 |
|  | PREDICTED: heterogeneous nuclear ribonucleoprotein H-like - [512908216_XP_004926627.1] | 1.01211838 | 0.4885971 |
|  | PREDICTED: ruvB-like 1-like - [72014808_XP_782589.1] | 1.07832687 | 0.488545 |
|  | PREDICTED: dolichyl-diphosphooligosaccharide--protein glycosyltransferase subunit 2-like - [512906640_XP_004926247.1] | 1.15611814 | 0.4884106 |
|  | PREDICTED: programmed cell death protein 4-like - [512901559_XP_004925164.1] | 1.08001639 | 0.4883728 |
|  | GL11419 - [195150333_XP_002016109.1] | 2.26318261 | 0.488312 |
|  | PREDICTED: dimethyladenosine transferase 2, mitochondrial-like - [512898989_XP_004924533.1] | 1.07972722 | 0.4882464 |
|  | PREDICTED: lysM and putative peptidoglycan-binding domain-containing protein 2-like - [512894091_XP_004923347.1] | #N/A | 0.4881048 |
|  | argonaute 3 - [166706858_NP_001098067.2] | 1.07637431 | 0.4880166 |
|  | PREDICTED: prostaglandin E synthase 2-like - [512921613_XP_004929919.1] | 1.13525419 | 0.4879758 |
|  | PREDICTED: transmembrane emp24 domain-containing protein 5-like - [512901298_XP_004925101.1] | #N/A | 0.4879126 |
|  | ribosomal protein S6 - [112982661_NP_001037566.1] | 1.09226119 | 0.4878752 |
|  | PREDICTED: mitochondrial 2-oxoglutarate/malate carrier protein-like - [512898511_XP_004924417.1] | 1.14412214 | 0.4878745 |
|  | PREDICTED: u4/U6 small nuclear ribonucleoprotein Prp31-like - [340729136_XP_003402864.1] | 1.09588447 | 0.4878734 |
|  | PREDICTED: zinc finger CCHC domain-containing protein 4-like isoform X1 - [512895417_XP_004923667.1] | 1.11653325 | 0.4877722 |
|  | PREDICTED: similar to UDP-glucose glycoprotein:glucosyltransferase - [189237348_XP_969332.2] | 1.17817818 | 0.4877674 |
|  | PREDICTED: glypican-1-like - [512893132_XP_004923105.1] | #N/A | 0.4876969 |
|  | PREDICTED: cytosolic Fe-S cluster assembly factor NUBP2 homolog - [512932603_XP_004932586.1] | #N/A | 0.4874251 |
|  | PREDICTED: acyl-CoA dehydrogenase family member 9, mitochondrial-like isoform X1 - [512906994_XP_004926333.1] | 1.16882981 | 0.4873801 |
|  | PREDICTED: LOW QUALITY PROTEIN: symplekin-like - [512915384_XP_004928376.1] | 1.22146855 | 0.4872145 |
|  | PREDICTED: chaperone activity of bc1 complex-like, mitochondrial-like isoform X1 - [498984706_XP_004530431.1] | 1.1642459 | 0.4871942 |
|  | GK23617 - [195442898_XP_002069183.1] | 0.4871604 |  |
|  | PREDICTED: inhibitor of growth protein 1-like - [512927327_XP_004931306.1] | 1.16281967 | 0.4868626 |
|  | PREDICTED: elongator complex protein 1-like isoform X1 - [524870783_XP_005092172.1] | 1.16109651 | 0.4868489 |
|  | PREDICTED: pyruvate carboxylase, mitochondrial - [390364224_XP_780258.2] | 1.13184776 | 0.4868428 |
|  | PREDICTED: alpha-1,3/1,6-mannosyltransferase ALG2-like - [512927218_XP_004931280.1] | 1.12300506 | 0.4867548 |
|  | PREDICTED: uncharacterized protein LOC101743404 - [512914008_XP_004928034.1] | #N/A | 0.486714 |
|  | PREDICTED: neurotactin-like isoform X1 - [512913538_XP_004927921.1] | 1.08064785 | 0.4866769 |
|  | PREDICTED: NHL repeat-containing protein 2-like - [512918827_XP_004929227.1] | 1.26066322 | 0.4866072 |
|  | maker-scaffold9910_size3163-snap-gene-0.2-mRNA-1 | 1.33880563 | 0.4865147 |
|  | PREDICTED: acetyl-CoA acetyltransferase, mitochondrial-like - [383864963_XP_003707947.1] | #N/A | 0.4865053 |
|  | PREDICTED: selT-like protein-like, partial - [512885094_XP_004921538.1] | 1.22335495 | 0.4864553 |
|  | mortality factor 4-like - [225703088_NP_001139536.1] | 1.21805205 | 0.4864406 |
|  | PREDICTED: equilibrative nucleoside transporter 2-like - [512934567_XP_004933064.1] | #N/A | 0.4862612 |
|  | maker-scaffold226_size255107-snap-gene-0.23-mRNA-1 | 1.09378857 | 0.4860756 |
|  | PREDICTED: phosphatidylinositol 4-kinase alpha-like - [512918227_XP_004929081.1] | #N/A | 0.4860168 |
|  | GF16847 - [194743506_XP_001954241.1] | 1.21146737 | 0.4860148 |
|  | PREDICTED: iron-sulfur cluster assembly enzyme ISCU, mitochondrial-like - [512933054_XP_004932696.1] | 1.08372252 | 0.4859871 |
|  | PREDICTED: uncharacterized protein DDB_G0274915-like - [512894562_XP_004923460.1] | 1.17568613 | 0.4859384 |
|  | PREDICTED: l-2-hydroxyglutarate dehydrogenase, mitochondrial-like - [512919941_XP_004929507.1] | 1.14732549 | 0.4858566 |
|  | PREDICTED: translocation protein SEC62-like isoform X1 - [512897304_XP_004924120.1] | 1.36264452 | 0.4857962 |
|  | snap-scaffold8149_size6505-abinit-gene-0.1-mRNA-1 | #N/A | 0.4857562 |
|  | PREDICTED: protein KIAA0664 homolog - [391326275_XP_003737643.1] | 1.0822441 | 0.4856131 |
|  | snap-scaffold226_size255107-abinit-gene-0.15-mRNA-1 | #N/A | 0.4855007 |
|  | serine-threonine kinase-like protein - [182509214_NP_001116818.1] | 1.12783251 | 0.4853821 |
|  | snap-scaffold5098_size22355-abinit-gene-0.4-mRNA-1 | #N/A | 0.4853544 |
|  | PREDICTED: zinc finger protein 484-like isoform X1 - [512890692_XP_004922556.1] | 1.02720743 | 0.485317 |
|  | pre-mRNA-processing-splicing factor 8 - [170062750_XP_001866804.1] | 1.10129486 | 0.4853046 |
|  | PREDICTED: dynamin-like 120 kDa protein, mitochondrial-like - [383853990_XP_003702505.1] | 1.16614647 | 0.4851316 |
|  | PREDICTED: pleckstrin homology domain-containing family A member 6-like, partial - [512940013_XP_004934377.1] | #N/A | 0.4850956 |
|  | PREDICTED: sphingomyelin synthase-related 1-like - [512907737_XP_004926517.1] | 1.10572358 | 0.4849836 |
|  | PREDICTED: protein peanut-like - [512922897_XP_004930228.1] | #N/A | 0.4847878 |
|  | snap-scaffold10399_size6901-abinit-gene-0.0-mRNA-1 | #N/A | 0.4846971 |
|  | PREDICTED: BTB/POZ domain-containing protein At1g21780-like - [512916537_XP_004928664.1] | 1.18465457 | 0.4843682 |
|  | PREDICTED: serine/threonine-protein phosphatase 5-like - [512894203_XP_004923376.1] | 1.12565786 | 0.4843618 |
|  | PREDICTED: glutaminyl-peptide cyclotransferase-like - [512887846_XP_004921989.1] | 1.16189884 | 0.4843066 |
|  | PREDICTED: mitochondrial Rho GTPase-like isoform 2 - [340721856_XP_003399330.1] | 1.15724264 | 0.4842887 |
|  | PREDICTED: neurochondrin homolog - [512887085_XP_004921864.1] | #N/A | 0.4841644 |
|  | 40S ribosomal protein SA - [162952033_NP_001106143.1] | 1.15571356 | 0.484116 |
|  | PREDICTED: uncharacterized protein LOC101743552 - [512919145_XP_004929303.1] | 1.08173234 | 0.4840371 |
|  | CRE-HSP-60 protein - [308480043_XP_003102229.1] | 1.13401394 | 0.4839739 |
|  | PREDICTED: acyl-CoA:lysophosphatidylglycerol acyltransferase 1-like - [512911760_XP_004927491.1] | 1.18557503 | 0.4839183 |
|  | PREDICTED: pre-mRNA-splicing factor SPF27-like - [512919497_XP_004929398.1] | 1.17870443 | 0.4839024 |
|  | PREDICTED: sialic acid synthase-like - [512931483_XP_004932314.1] | #N/A | 0.4837173 |
|  | PREDICTED: uncharacterized protein LOC101739704 - [512931711_XP_004932370.1] | 1.15909315 | 0.4836961 |
|  | PREDICTED: anamorsin homolog - [512928304_XP_004931546.1] | 1.05729812 | 0.4836911 |
|  | PREDICTED: MATH and LRR domain-containing protein PFE0570w-like - [512914568_XP_004928174.1] | #N/A | 0.4835381 |
|  | epimerase family protein SDR39U1 - [114052837_NP_001040495.1] | 1.14758499 | 0.4834764 |
|  | PREDICTED: uncharacterized protein LOC101736979 - [512913518_XP_004927916.1] | 1.14941531 | 0.4834692 |
|  | elongation factor Ts - [114051515_NP_001040359.1] | 1.16936006 | 0.4834683 |
|  | PREDICTED: hypothetical protein LOC100575841 - [328701240_XP_003241537.1] | #N/A | 0.4834317 |
|  | PREDICTED: tRNA-specific adenosine deaminase 2-like - [512887020_XP_004921853.1] | 1.06946501 | 0.4834158 |
|  | PREDICTED: C-terminal-binding protein-like - [391338504_XP_003743598.1] | 1.07951967 | 0.4833804 |
|  | 3-hydroxyacyl-CoA dehydrogenase - [114051676_NP_001040424.1] | 1.10087761 | 0.4833004 |
|  | GH21538 - [195027758_XP_001986749.1] | #N/A | 0.483241 |
|  | PREDICTED: tyrosine-protein phosphatase non-receptor type 9-like - [512920396_XP_004929622.1] | #N/A | 0.4830987 |
|  | CRE-CTS-1 protein - [308502315_XP_003113342.1] | 1.11072149 | 0.483067 |
|  | PREDICTED: nuclear pore complex protein Nup214-like - [512932125_XP_004932468.1] | 1.14426249 | 0.4830007 |
|  | PREDICTED: clathrin heavy chain 1-like isoform X1 - [524869547_XP_005091566.1] | 1.13420475 | 0.4826874 |
|  | dihydrolipoamide dehydrogenase - [112983096_NP_001037054.1] | 1.17820851 | 0.4823415 |
|  | PREDICTED: uncharacterized protein LOC101743157 - [512891384_XP_004922674.1] | 1.08792099 | 0.4822649 |
|  | PREDICTED: ankyrin repeat and zinc finger domain-containing protein 1-like - [512910246_XP_004927121.1] | 1.23469506 | 0.4822631 |
|  | PREDICTED: protein disulfide-isomerase TMX3-like isoform X1 - [512928190_XP_004931521.1] | 1.199594 | 0.4822267 |
|  | ribosomal protein L34 - [151301158_NP_001093076.1] | 1.12711908 | 0.4822141 |
|  | PREDICTED: mediator of RNA polymerase II transcription subunit 20-like - [512936525_XP_004933539.1] | #N/A | 0.4821674 |
|  | PREDICTED: beta-catenin-like protein 1-like - [512899535_XP_004924669.1] | #N/A | 0.4820712 |
|  | PREDICTED: 39S ribosomal protein L41, mitochondrial-like - [498949838_XP_004522918.1] | 1.11712492 | 0.4819677 |
|  | ribosomal protein L10 - [160333861_NP_001037048.1] | 1.12572478 | 0.4819623 |
|  | signal recognition particle receptor beta subunit - [148298679_NP_001091817.1] | 1.16867513 | 0.4817193 |
|  | PREDICTED: nucleolar complex protein 3 homolog - [512904879_XP_004925806.1] | 1.23609974 | 0.4816955 |
|  | PREDICTED: exonuclease 3'-5' domain-containing protein 2-like - [512892918_XP_004923051.1] | 0.9896112 | 0.4816911 |
|  | ribosomal protein L9 - [112983495_NP_001037144.1] | 1.10823799 | 0.4816754 |
|  | PREDICTED: LOW QUALITY PROTEIN: mitotic checkpoint serine/threonine-protein kinase BUB1 beta-like - [512901785_XP_004925221.1] | 1.10802527 | 0.4816554 |
|  | hypothetical protein CRE_02939 - [308499086_XP_003111729.1] | 1.17373276 | 0.4814753 |
|  | PREDICTED: uncharacterized protein LOC101740623 - [512889587_XP_004922319.1] | 1.24545973 | 0.4813445 |
|  | ribosomal protein L23 - [112984274_NP_001037227.1] | 1.13505226 | 0.481334 |
|  | hypothetical protein CRE_18959 - [308506629_XP_003115497.1] | 1.1075131 | 0.4812569 |
|  | peptidyl-prolyl cis-trans isomerase-like - [256074558_XP_002573591.1] | 1.09492108 | 0.4811868 |
|  | snap-scaffold12554_size9432-abinit-gene-0.2-mRNA-1 | #N/A | 0.481154 |
|  | AGAP005375-PA - [158294075_XP_315383.4] | 1.05036607 | 0.4811358 |
|  | 71 kDa protein - [350536745_NP_001233145.1] | 1.21654001 | 0.4811141 |
|  | signal peptidase complex subunit 2 - [114052092_NP_001040214.1] | 1.16776556 | 0.4810817 |
|  | PREDICTED: similar to purine biosynthesis protein 6, pur6 isoform 1 - [91092272_XP_966432.1] | 1.09129013 | 0.4809411 |
|  | PREDICTED: NADH dehydrogenase [ubiquinone] 1 beta subcomplex subunit 11, mitochondrial-like - [512900232_XP_004924841.1] | 1.12123126 | 0.4809244 |
|  | PREDICTED: putative neutral sphingomyelinase-like - [512913002_XP_004927793.1] | 1.21332575 | 0.480692 |
|  | PREDICTED: N-sulphoglucosamine sulphohydrolase-like - [512887989_XP_004922014.1] | #N/A | 0.4806279 |
|  | PREDICTED: DDB1- and CUL4-associated factor-like 1-like - [512907663_XP_004926499.1] | 1.12897977 | 0.4805714 |
|  | phospholipase C beta 1 - [284813573_NP_001165392.1] | #N/A | 0.4805302 |
|  | GL11185 - [195149405_XP_002015648.1] | #N/A | 0.4804971 |
|  | PREDICTED: cGMP-dependent protein kinase egl-4-like isoform 2 - [390357547_XP_003729032.1] | 0.99425494 | 0.4803949 |
|  | PREDICTED: tyrosine kinase receptor Cad96Ca-like - [512892230_XP_004922880.1] | 1.02749816 | 0.4803828 |
|  | PREDICTED: spectrin beta chain - [328777761_XP_396777.4] | 1.19939951 | 0.4803525 |
|  | PREDICTED: mitochondrial ubiquitin ligase activator of nfkb 1-A-like - [512885470_XP_004921600.1] | 1.14152425 | 0.480155 |
|  | cleavage and polyadenylation specificity factor subunit 3 - [170052069_XP_001862054.1] | 1.12952037 | 0.4801075 |
|  | PREDICTED: RNA-binding protein 39-like isoform X1 - [498998794_XP_004533936.1] | 1.07093637 | 0.4800626 |
|  | PREDICTED: U3 small nucleolar RNA-associated protein 15 homolog - [512920438_XP_004929632.1] | 1.15031355 | 0.4800156 |
|  | ras-related GTP-binding protein 4b - [114052070_NP_001040209.1] | #N/A | 0.4799467 |
|  | PREDICTED: probable ATP-dependent RNA helicase DDX47-like - [512895366_XP_004923655.1] | 1.1844645 | 0.4799062 |
|  | maker-scaffold1255_size70687-snap-gene-0.18-mRNA-1 | 1.13801189 | 0.4797676 |
|  | PREDICTED: condensin complex subunit 3-like - [512917412_XP_004928880.1] | 1.17715433 | 0.4797314 |
|  | peptidyl-prolyl cis-trans isomerase - [114051666_NP_001040172.1] | 1.15481779 | 0.4797214 |
|  | PREDICTED: oxysterol-binding protein-related protein 9-like - [345494831_XP_001603798.2] | #N/A | 0.4792252 |
|  | PREDICTED: protein lin-7 homolog C-like - [512885614_XP_004921623.1] | #N/A | 0.4791625 |
|  | 40S ribosomal protein S4 - [112984078_NP_001037257.1] | 1.10471952 | 0.479138 |
|  | metalloprotease - [157131944_XP_001662373.1] | 1.13218649 | 0.4790953 |
|  | ubiquitin-specific protease, partial - [157132977_XP_001662730.1] | #N/A | 0.4789495 |
|  | PREDICTED: probable enoyl-CoA hydratase, mitochondrial-like isoform X1 - [498982817_XP_004529957.1] | 1.10672659 | 0.4787075 |
|  | ADP-ribosylation factor-like 6 interacting protein - [114051297_NP_001040353.1] | 1.1872219 | 0.4786314 |
|  | PREDICTED: CBP80/20-dependent translation initiation factor-like isoform X1 - [512895334_XP_004923647.1] | 1.19023835 | 0.4785613 |
|  | PREDICTED: histone deacetylase 6-like - [512896373_XP_004923895.1] | 1.14117066 | 0.4784569 |
|  | ribosomal protein S13 - [148298648_NP_001091754.1] | 1.14699978 | 0.4783936 |
|  | PREDICTED: protein-S-isoprenylcysteine O-methyltransferase-like - [512895448_XP_004923674.1] | #N/A | 0.4783146 |
|  | BET1-like protein - [114051141_NP_001040395.1] | 1.14236593 | 0.4782312 |
|  | ribosomal protein L28 - [112984200_NP_001037237.1] | 1.14130223 | 0.4782263 |
|  | PREDICTED: translocation protein SEC63 homolog isoform 1 - [328776350_XP_395961.4] | #N/A | 0.4782024 |
|  | PREDICTED: UBX domain-containing protein 1-like - [512919643_XP_004929435.1] | 1.18209979 | 0.4781489 |
|  | PREDICTED: cyclin-T-like - [512935815_XP_004933366.1] | 1.25991967 | 0.4780412 |
|  | PREDICTED: protein F29A7.6-like - [512938468_XP_004934012.1] | 1.18564468 | 0.4779763 |
|  | PREDICTED: atlastin-like - [512923057_XP_004930260.1] | 1.36688207 | 0.4778869 |
|  | RNA binding motif protein Y14 - [112982916_NP_001037578.1] | 1.11111092 | 0.4778409 |
|  | PREDICTED: tubulin gamma-1 chain-like isoform 2 - [340722873_XP_003399825.1] | 1.09711697 | 0.4778289 |
|  | PREDICTED: RNA-binding protein 25-like - [499010076_XP_004536712.1] | 1.11770031 | 0.4775303 |
|  | mitochondrial carrier homolog - [112983868_NP_001036860.1] | 1.11461293 | 0.4775031 |
|  | PREDICTED: uncharacterized protein LOC101736992 - [512931431_XP_004932301.1] | 1.18175475 | 0.477495 |
|  | PREDICTED: aurora kinase B-like - [512937305_XP_004933735.1] | 1.04642334 | 0.4774175 |
|  | PREDICTED: pre-mRNA-processing factor 17-like isoform X1 - [512892375_XP_004922916.1] | #N/A | 0.4772844 |
|  | PREDICTED: uncharacterized protein LOC101736905 - [512934184_XP_004932972.1] | #N/A | 0.4771087 |
|  | snap-scaffold11501_size11360-processed-gene-0.3-mRNA-1 | #N/A | 0.4768135 |
|  | PREDICTED: LOW QUALITY PROTEIN: protein lava lamp-like - [512914193_XP_004928081.1] | #N/A | 0.476754 |
|  | PREDICTED: uncharacterized protein LOC101738916 - [512887177_XP_004921879.1] | 0.99470748 | 0.4766881 |
|  | PREDICTED: NADH dehydrogenase [ubiquinone] 1 alpha subcomplex subunit 9, mitochondrial-like - [512913550_XP_004927924.1] | 1.14645429 | 0.4765879 |
|  | PREDICTED: short-chain specific acyl-CoA dehydrogenase, mitochondrial-like isoform X1 - [512932587_XP_004932582.1] | 1.09396429 | 0.4763208 |
|  | PREDICTED: protein SERAC1-like - [512935154_XP_004933205.1] | #N/A | 0.4763131 |
|  | defective in cullin neddylation protein - [114051233_NP_001040114.1] | 1.21079661 | 0.4763126 |
|  | proline synthetase co-transcribed bacterial-like protein - [114051511_NP_001040304.1] | 1.21293563 | 0.4763085 |
|  | PREDICTED: similar to osa CG7467-PA - [189233762_XP_001814255.1] | 1.14370014 | 0.4762811 |
|  | CRE-ANC-1 protein - [308485166_XP_003104782.1] | #N/A | 0.4762538 |
|  | PREDICTED: zinc finger MYM-type protein 4-like - [512927657_XP_004931389.1] | 1.13194893 | 0.4761952 |
|  | fructose 1,6-bisphosphate aldolase - [148298685_NP_001091766.1] | 1.09784015 | 0.4761897 |
|  | GK10955 - [195445992_XP_002070577.1] | 1.24437617 | 0.4761123 |
|  | PREDICTED: mediator of RNA polymerase II transcription subunit 12-like - [512899614_XP_004924689.1] | #N/A | 0.4758976 |
|  | PREDICTED: uncharacterized protein LOC101744231 - [512915830_XP_004928484.1] | #N/A | 0.4758764 |
|  | PREDICTED: dnaJ homolog subfamily C member 3-like - [512898714_XP_004924464.1] | 1.16613354 | 0.4758085 |
|  | PREDICTED: cleavage and polyadenylation specificity factor subunit CG7185-like isoform X2 - [512902577_XP_004925410.1] | 1.21339634 | 0.4758 |
|  | PREDICTED: ribonuclease H1-like - [512892585_XP_004922968.1] | 0.9251446 | 0.4757097 |
|  | PREDICTED: serine/threonine-protein phosphatase Pgam5, mitochondrial-like isoform X1 - [512939951_XP_004934358.1] | #N/A | 0.4756663 |
|  | PREDICTED: PRKR-interacting protein 1 homolog - [512931022_XP_004932205.1] | 1.14714968 | 0.4755827 |
|  | PREDICTED: protein disulfide-isomerase A3-like - [156553206_XP_001599732.1] | 1.17621387 | 0.4755161 |
|  | PREDICTED: pre-rRNA-processing protein TSR1 homolog - [390334118_XP_003723853.1] | 1.11046278 | 0.4751797 |
|  | splicing factor 3B subunit 3 - [339259094_XP_003369733.1] | 1.05223037 | 0.475136 |
|  | carotenoid-binding protein isoform 2 - [160333446_NP_001103832.1] | 0.9195981 | 0.4750528 |
|  | AGAP009672-PA, partial - [158298546_XP_001689143.1] | 1.12889409 | 0.4749873 |
|  | snap-scaffold14025_size3834-abinit-gene-0.0-mRNA-1 | #N/A | 0.4747844 |
|  | PREDICTED: casein kinase I isoform delta-like isoform X1 - [524884103_XP_005098675.1] | #N/A | 0.474649 |
|  | GM24457 - [195327903_XP_002030656.1] | 1.12617457 | 0.4740649 |
|  | GL15642 - [195176355_XP_002028747.1] | #N/A | 0.4740573 |
|  | PREDICTED: delta(3,5)-Delta(2,4)-dienoyl-CoA isomerase, mitochondrial-like - [512895733_XP_004923742.1] | 1.13197158 | 0.4739225 |
|  | PREDICTED: LOW QUALITY PROTEIN: microtubule-actin cross-linking factor 1-like - [391348043_XP_003748261.1] | #N/A | 0.47377 |
|  | PREDICTED: transmembrane protein 222-like - [512895476_XP_004923681.1] | #N/A | 0.4737597 |
|  | PREDICTED: similar to peroxisomal membrane protein PEX16 - [91080479_XP_970655.1] | 1.17012544 | 0.4737368 |
|  | PREDICTED: mpv17-like protein 2-like - [512929905_XP_004931938.1] | #N/A | 0.4736725 |
|  | PREDICTED: similar to AGAP008327-PA - [91082963_XP_973727.1] | 1.15823426 | 0.47334 |
|  | ATP-binding cassette sub-family B member 7 mitochondrial precursor (abc7) - [256087346_XP_002579832.1] | #N/A | 0.472974 |
|  | PREDICTED: similar to GH06117p - [189239726_XP_001807608.1] | 1.2016719 | 0.4729305 |
|  | PREDICTED: protein phosphatase 1 regulatory subunit 7-like - [512896721_XP_004923978.1] | 1.11365263 | 0.4728681 |
|  | maker-scaffold1569_size233047-snap-gene-1.35-mRNA-1 | 1.05338862 | 0.4727694 |
|  | PREDICTED: uncharacterized protein LOC101737762 isoform X1 - [512892922_XP_004923052.1] | 1.17466191 | 0.4727426 |
|  | geranylgeranyltransferase type I beta subunit - [300068969_NP_001177770.1] | 1.04269033 | 0.472706 |
|  | GA26930, isoform A - [198449530_XP_002136920.1] | 1.12155255 | 0.4725305 |
|  | PREDICTED: LOW QUALITY PROTEIN: sterile alpha and TIR motif-containing protein 1-like - [380030235_XP_003698758.1] | 1.19026863 | 0.4724418 |
|  | PREDICTED: venom carboxylesterase-6-like - [380016494_XP_003692218.1] | 1.10664165 | 0.4721703 |
|  | PREDICTED: gametogenetin-binding protein 2-like - [512923660_XP_004930406.1] | #N/A | 0.4720436 |
|  | PREDICTED: metal transporter CNNM2-like - [512911157_XP_004927345.1] | 1.10716669 | 0.4719876 |
|  | PREDICTED: succinate dehydrogenase [ubiquinone] flavoprotein subunit, mitochondrial-like - [512929202_XP_004931765.1] | 1.18934094 | 0.4717745 |
|  | ociad protein isoform 1 - [153791696_NP_001093266.1] | 1.17183626 | 0.4717531 |
|  | PREDICTED: amidophosphoribosyltransferase-like - [512916510_XP_004928658.1] | #N/A | 0.4717375 |
|  | PREDICTED: regulator of microtubule dynamics protein 1-like isoform X3 - [512922893_XP_004930227.1] | #N/A | 0.4715915 |
|  | PREDICTED: ectonucleoside triphosphate diphosphohydrolase 5-like - [512891871_XP_004922790.1] | #N/A | 0.4714291 |
|  | PREDICTED: low affinity cationic amino acid transporter 2-like - [512902463_XP_004925382.1] | #N/A | 0.4713737 |
|  | PREDICTED: alpha-(1,3)-fucosyltransferase 10-like isoform X1 - [512932484_XP_004932556.1] | 1.1260457 | 0.4713513 |
|  | ribosomal protein L12 - [112983546_NP_001037150.1] | 1.11198735 | 0.4712205 |
|  | PREDICTED: molybdopterin synthase catalytic subunit-like isoform X1 - [512894290_XP_004923393.1] | 1.03019419 | 0.4711848 |
|  | transgelin - [114051357_NP_001040372.1] | 1.08836521 | 0.4711727 |
|  | PREDICTED: arf-GAP with dual PH domain-containing protein 1-like - [512934065_XP_004932943.1] | #N/A | 0.4710877 |
|  | GK23735 - [195433206_XP_002064606.1] | 1.14355148 | 0.4708782 |
|  | GF19216 - [194769430_XP_001966807.1] | 1.12581819 | 0.4707849 |
|  | predicted protein - [156406520_XP_001641093.1] | 1.08648888 | 0.4707837 |
|  | PREDICTED: uncharacterized protein LOC101742189 isoform X1 - [512890084_XP_004922451.1] | #N/A | 0.4705282 |
|  | PREDICTED: acetyl-CoA hydrolase-like - [512936895_XP_004933631.1] | #N/A | 0.4704332 |
|  | H+ transporting ATP synthase delta subunit - [151301059_NP_001093091.1] | 1.14390598 | 0.4703918 |
|  | exuperantia - [114053141_NP_001040124.1] | 1.1182119 | 0.4701411 |
|  | PREDICTED: uncharacterized protein LOC101744356, partial - [512896449_XP_004923914.1] | 1.1987884 | 0.4699449 |
|  | PREDICTED: cytochrome P450 6j1 - [512923906_XP_004930467.1] | #N/A | 0.4699268 |
|  | PREDICTED: adipocyte plasma membrane-associated protein-like - [512899100_XP_004924560.1] | 1.20679276 | 0.4698206 |
|  | PREDICTED: general transcription factor IIF subunit 2-like - [512925936_XP_004930959.1] | 1.14852024 | 0.4697871 |
|  | PREDICTED: uncharacterized protein LOC101736142 - [512913490_XP_004927909.1] | 1.14661304 | 0.4697427 |
|  | PREDICTED: uncharacterized protein LOC101738379 - [512894621_XP_004923474.1] | #N/A | 0.4696785 |
|  | PREDICTED: general transcription factor IIE subunit 2-like - [512899785_XP_004924732.1] | 1.06759554 | 0.4696213 |
|  | PREDICTED: carnitine O-acetyltransferase-like - [512919117_XP_004929296.1] | 1.13602375 | 0.4694298 |
|  | PREDICTED: ran GTPase-activating protein 1-like - [512888893_XP_004922162.1] | 1.13935659 | 0.4692946 |
|  | PREDICTED: LOW QUALITY PROTEIN: d-2-hydroxyglutarate dehydrogenase, mitochondrial-like - [380028530_XP_003697951.1] | 1.11327471 | 0.4692153 |
|  | PREDICTED: FAST kinase domain-containing protein 5-like - [512913397_XP_004927885.1] | 1.13244359 | 0.4690631 |
|  | PREDICTED: mannosyl-oligosaccharide glucosidase-like - [512901532_XP_004925158.1] | 1.20435195 | 0.4690258 |
|  | PREDICTED: succinate dehydrogenase [ubiquinone] iron-sulfur subunit, mitochondrial-like - [512934347_XP_004933012.1] | 1.1505867 | 0.468706 |
|  | protein phosphatase 1 catalytic subunit - [114052575_NP_001040480.1] | 1.16454274 | 0.4685901 |
|  | ribosomal protein L24 - [112984248_NP_001037231.1] | #N/A | 0.4681255 |
|  | ribosomal protein L27A - [112983527_NP_001037522.1] | 1.11388801 | 0.4680445 |
|  | PREDICTED: similar to kismet CG3696-PA - [189237363_XP_970443.2] | 1.14538492 | 0.4680361 |
|  | PREDICTED: bis(5'-nucleosyl)-tetraphosphatase [asymmetrical]-like - [512890078_XP_004922449.1] | 1.13922854 | 0.4678847 |
|  | AGAP002280-PA - [347967542_XP_307898.5] | #N/A | 0.4678839 |
|  | PREDICTED: cytochrome c oxidase assembly protein COX11, mitochondrial-like - [512937062_XP_004933673.1] | 1.13207196 | 0.4678608 |
|  | PREDICTED: integrator complex subunit 2-like - [328698586_XP_001944802.2] | 1.13760331 | 0.4678018 |
|  | PREDICTED: protein YIPF5-like - [512924465_XP_004930602.1] | 1.20676694 | 0.4676831 |
|  | PREDICTED: aminomethyltransferase, mitochondrial-like - [512927436_XP_004931334.1] | 1.11810238 | 0.4676636 |
|  | PREDICTED: muscle M-line assembly protein unc-89-like - [512890305_XP_004922495.1] | #N/A | 0.4674406 |
|  | PREDICTED: LOW QUALITY PROTEIN: chromobox protein homolog 1-like - [512894868_XP_004923534.1] | #N/A | 0.467406 |
|  | splicing factor 3B subunit 2 - [170048906_XP_001870832.1] | 1.11538326 | 0.4673061 |
|  | sodium/potassium-transporting ATPase subunit alpha - [339258464_XP_003369418.1] | 1.10041579 | 0.4672595 |
|  | polymerase delta interacting protein 3 - [112982657_NP_001037640.1] | 1.19490353 | 0.4672317 |
|  | predicted protein, partial - [156365823_XP_001626842.1] | 1.23713505 | 0.4672186 |
|  | PREDICTED: putative mitochondrial inner membrane protein-like - [512906225_XP_004926143.1] | 1.15096729 | 0.4671969 |
|  | PREDICTED: mitochondrial import receptor subunit TOM40 homolog 1-like - [512908256_XP_004926637.1] | 1.03096461 | 0.467016 |
|  | PREDICTED: ubiquitin domain-containing protein UBFD1-like - [512928796_XP_004931667.1] | 1.11043045 | 0.4668682 |
|  | PREDICTED: protein tweety-like - [512932737_XP_004932620.1] | 1.11434191 | 0.4666971 |
|  | PREDICTED: elongation factor Tu, mitochondrial-like - [156549512_XP_001604878.1] | 1.149073 | 0.4662665 |
|  | PREDICTED: uncharacterized protein LOC101738573 - [512909195_XP_004926862.1] | 1.17378037 | 0.4662204 |
|  | PREDICTED: tetratricopeptide repeat protein 19 homolog, mitochondrial-like - [512889780_XP_004922370.1] | 1.14715105 | 0.4661856 |
|  | PREDICTED: tRNA pseudouridine synthase A, mitochondrial-like isoform X3 - [512904739_XP_004925771.1] | #N/A | 0.4661477 |
|  | PREDICTED: long-chain-fatty-acid--CoA ligase ACSBG2 isoform X1 - [512922647_XP_004930167.1] | 1.14881684 | 0.4658099 |
|  | transport protein Sec61 alpha subunit - [112983370_NP_001037628.1] | #N/A | 0.4657622 |
|  | PREDICTED: box C/D snoRNA protein 1-like - [340375394_XP_003386220.1] | #N/A | 0.4655796 |
|  | PREDICTED: uncharacterized protein LOC101745859 - [512898347_XP_004924377.1] | #N/A | 0.4654983 |
|  | PREDICTED: putative RNA polymerase II subunit B1 CTD phosphatase RPAP2-like - [512916601_XP_004928680.1] | 1.29061048 | 0.4654972 |
|  | PREDICTED: 28S ribosomal protein S29, mitochondrial-like - [512906644_XP_004926248.1] | 1.12387906 | 0.4652953 |
|  | PREDICTED: ATP-binding cassette sub-family D member 3-like, partial - [512900091_XP_004924807.1] | 1.17741895 | 0.4652902 |
|  | PREDICTED: SWI/SNF-related matrix-associated actin-dependent regulator of chromatin subfamily E member 1-like - [512891328_XP_004922664.1] | 1.14519748 | 0.4652746 |
|  | PREDICTED: malectin-like - [512923918_XP_004930470.1] | 1.1666338 | 0.465264 |
|  | PREDICTED: 6-phosphofructokinase-like isoform X2 - [512922706_XP_004930182.1] | 1.14370178 | 0.4651936 |
|  | PREDICTED: cell growth-regulating nucleolar protein-like isoform X1 - [512887118_XP_004921870.1] | 1.15889853 | 0.465158 |
|  | PREDICTED: similar to AGAP002348-PA - [91080613_XP_974147.1] | #N/A | 0.4650985 |
|  | PREDICTED: heterogeneous nuclear ribonucleoprotein U-like protein 1-like - [512893414_XP_004923175.1] | 1.13351841 | 0.4650286 |
|  | maker-scaffold5499_size28385-snap-gene-0.8-mRNA-1 | 1.11928374 | 0.4649734 |
|  | PREDICTED: leucine-rich PPR motif-containing protein, mitochondrial-like - [512922079_XP_004930031.1] | 1.13714811 | 0.4649579 |
|  | PREDICTED: DNA-directed RNA polymerase II subunit RPB11-like - [512923730_XP_004930423.1] | #N/A | 0.4648919 |
|  | maker-scaffold322_size93182-exonerate_est2genome-gene-0.0-mRNA-1 | #N/A | 0.4648161 |
|  | PREDICTED: zinc finger protein 391-like isoform X1 - [512934543_XP_004933058.1] | 1.20534779 | 0.4647999 |
|  | PREDICTED: fatty acid synthase-like - [512912462_XP_004927661.1] | #N/A | 0.4647316 |
|  | ATP synthase beta subunit - [170040305_XP_001847944.1] | 1.12784537 | 0.4647083 |
|  | PREDICTED: Y+L amino acid transporter 2-like - [512900708_XP_004924956.1] | #N/A | 0.4646804 |
|  | PREDICTED: leucine-rich repeat protein soc-2 homolog - [512924326_XP_004930568.1] | 1.15947961 | 0.46462 |
|  | PREDICTED: probable 28S ribosomal protein S6, mitochondrial-like - [512913265_XP_004927854.1] | 1.1327139 | 0.4646195 |
|  | PREDICTED: zinc transporter ZIP11-like - [512928224_XP_004931528.1] | 1.10300739 | 0.4645934 |
|  | ribosomal protein L36 - [112984158_NP_001037245.1] | 1.10255981 | 0.4644999 |
|  | PREDICTED: GPN-loop GTPase 3-like - [512891592_XP_004922723.1] | 1.17159894 | 0.4644848 |
|  | eukaryotic initiation factor 4A-III - [163838674_NP_001106217.1] | 1.04694305 | 0.4644127 |
|  | PREDICTED: electron transfer flavoprotein-ubiquinone oxidoreductase, mitochondrial-like - [512902642_XP_004925425.1] | 1.16352272 | 0.4642392 |
|  | PIWI - [157116679_XP_001652831.1] | 1.12168752 | 0.4641206 |
|  | ribosomal protein L7A - [112983462_NP_001037138.1] | 1.12794257 | 0.4640492 |
|  | fumarylacetoacetase - [160333413_NP_001103763.1] | 1.12436219 | 0.4639949 |
|  | PREDICTED: glutaminyl-peptide cyclotransferase-like - [512887846_XP_004921989.1] | 1.16709654 | 0.4639886 |
|  | argonaute 2 - [166706854_NP_001036995.2] | 1.03382926 | 0.4638983 |
|  | PREDICTED: uncharacterized protein LOC101743192 - [512928848_XP_004931680.1] | 1.1294523 | 0.4637999 |
|  | GI15296 - [195129790_XP_002009337.1] | 1.21408432 | 0.4636119 |
|  | PREDICTED: carnitine O-palmitoyltransferase 1, liver isoform-like - [512910525_XP_004927194.1] | #N/A | 0.4634833 |
|  | PREDICTED: transcription elongation factor SPT4-like - [380030199_XP_003698741.1] | 1.07915427 | 0.4634449 |
|  | PREDICTED: testis-expressed sequence 10 protein-like - [512932987_XP_004932679.1] | #N/A | 0.4632307 |
|  | PREDICTED: similar to mitochondrial processing peptidase beta subunit - [91085025_XP_973732.1] | 1.15895304 | 0.463118 |
|  | juvenile hormone esterase binding protein - [114052406_NP_001040474.1] | 1.18602604 | 0.4631042 |
|  | PREDICTED: uncharacterized protein LOC101746757 - [512904867_XP_004925803.1] | 0.96680666 | 0.462948 |
|  | PREDICTED: u2 small nuclear ribonucleoprotein B''-like - [340726396_XP_003401545.1] | 1.07373976 | 0.4629196 |
|  | PREDICTED: threonylcarbamoyladenosine tRNA methylthiotransferase-like - [524874253_XP_005093865.1] | 0.9940176 | 0.4629006 |
|  | PREDICTED: uncharacterized protein LOC101747157 - [512889146_XP_004922204.1] | 0.96134762 | 0.462864 |
|  | mitochondrial translational release factor 1 - [114052118_NP_001040455.1] | 1.18807657 | 0.4628004 |
|  | PREDICTED: NADPH:adrenodoxin oxidoreductase, mitochondrial-like, partial - [512912740_XP_004927730.1] | 1.20128742 | 0.4623396 |
|  | GK24416 - [195437566_XP_002066711.1] | 1.08355008 | 0.4622858 |
|  | PREDICTED: probable ATP-dependent RNA helicase CG8611-like - [512916973_XP_004928772.1] | 1.19484089 | 0.4621492 |
|  | PREDICTED: similar to cell division control protein - [91091782_XP_969684.1] | 1.12266693 | 0.4618532 |
|  | adenylate kinase 2 - [284813563_NP_001165387.1] | 1.11854704 | 0.4618065 |
|  | PREDICTED: GPI ethanolamine phosphate transferase 3-like - [512936055_XP_004933425.1] | 1.15602712 | 0.4616826 |
|  | ribosomal protein S5 - [112984070_NP_001037259.1] | 1.16266642 | 0.4616309 |
|  | PREDICTED: transcription initiation factor TFIID subunit 7-like isoform X1 - [512921218_XP_004929824.1] | 1.28871194 | 0.4613109 |
|  | PREDICTED: RWD domain-containing protein 1-like - [512926702_XP_004931152.1] | 1.12357832 | 0.4611376 |
|  | PREDICTED: fatty-acid amide hydrolase 2-like - [512896796_XP_004923996.1] | 1.16022749 | 0.4611248 |
|  | PREDICTED: DNA-directed RNA polymerases I, II, and III subunit RPABC3-like - [512907576_XP_004926477.1] | 1.10408204 | 0.4610208 |
|  | PREDICTED: succinyl-CoA ligase [ADP/GDP-forming] subunit alpha, mitochondrial-like - [512917873_XP_004928994.1] | 1.15208029 | 0.460906 |
|  | PREDICTED: sister chromatid cohesion protein PDS5 homolog B-A-like - [328778597_XP_623860.2] | 1.12423882 | 0.4608685 |
|  | PREDICTED: microtubule-associated protein futsch-like - [512922402_XP_004930111.1] | 1.25409317 | 0.4607717 |
|  | PREDICTED: RNA polymerase-associated protein CTR9 homolog - [156543124_XP_001605583.1] | 1.01882321 | 0.4607062 |
|  | 40S ribosomal protein S3a - [112984098_NP_001037255.1] | 1.14255125 | 0.4606635 |
|  | 60S ribosomal protein L3 - [256079531_XP_002576040.1] | 1.20432315 | 0.4606255 |
|  | PREDICTED: hemicentin-1-like - [512895729_XP_004923741.1] | #N/A | 0.460336 |
|  | ribosomal protein L13 - [112983562_NP_001037153.1] | 1.1083582 | 0.4602652 |
|  | surfeit 4-like protein - [114050789_NP_001040156.1] | 1.13043669 | 0.4602383 |
|  | GTP-binding nuclear protein Ran - [114052751_NP_001040274.1] | 1.08675976 | 0.4598842 |
|  | PREDICTED: uncharacterized protein LOC101736419 - [512927563_XP_004931366.1] | 1.11309793 | 0.4598591 |
|  | PREDICTED: mitochondrial import inner membrane translocase subunit TIM50-C-like - [512911979_XP_004927544.1] | 1.13381361 | 0.4596906 |
|  | PREDICTED: integrator complex subunit 10-like - [512937286_XP_004933730.1] | 1.28023872 | 0.4596461 |
|  | GH24170 - [195048248_XP_001992496.1] | #N/A | 0.4595581 |
|  | PREDICTED: similar to CG2972 CG2972-PA - [91083257_XP_974124.1] | 1.15436995 | 0.4595465 |
|  | PHD-finger 5A - [148298705_NP_001091825.1] | 1.11828128 | 0.4595451 |
|  | PREDICTED: WAS/WASL-interacting protein family member 1-like - [512901633_XP_004925183.1] | 1.12580115 | 0.4594829 |
|  | PREDICTED: peptide deformylase, mitochondrial-like - [512926933_XP_004931209.1] | 1.06826973 | 0.4594051 |
|  | prolactin regulatory binding-element protein - [114051449_NP_001040367.1] | #N/A | 0.4593973 |
|  | receptor expression enhancing protein isoform 2 - [114050941_NP_001040329.1] | 1.1187941 | 0.4593462 |
|  | PREDICTED: alpha-tocopherol transfer protein-like - [512925719_XP_004930907.1] | 1.03309853 | 0.4593422 |
|  | AGAP011340-PA - [158287218_XP_309311.4] | 1.21166477 | 0.4591266 |
|  | GJ22920 - [195391142_XP_002054222.1] | 1.08785235 | 0.4587887 |
|  | will die slowly - [112982984_NP_001037087.1] | 1.0645426 | 0.4583698 |
|  | PREDICTED: WD repeat-containing protein 3-like - [524885813_XP_005099510.1] | 1.2072396 | 0.4583319 |
|  | PREDICTED: uncharacterized protein LOC101738799 - [512904005_XP_004925756.1] | 1.21514607 | 0.4581724 |
|  | 3-hydroxyisobutyrate dehydrogenase - [195963353_NP_001124349.1] | 1.10401498 | 0.4581648 |
|  | PREDICTED: tetratricopeptide repeat protein 37-like - [512923902_XP_004930466.1] | 1.12509214 | 0.4580642 |
|  | cytochrome c oxidase polypeptide IV - [118918433_NP_001073120.1] | 1.15844626 | 0.4580516 |
|  | PREDICTED: xaa-Pro dipeptidase-like isoform X3 - [512915107_XP_004928308.1] | #N/A | 0.4577687 |
|  | PREDICTED: 3-hydroxyacyl-CoA dehydrogenase type-2-like - [512910163_XP_004927100.1] | 1.11266667 | 0.4576786 |
|  | PREDICTED: synaptobrevin homolog YKT6-like - [512914955_XP_004928270.1] | 1.19062528 | 0.4576544 |
|  | PREDICTED: geranylgeranyl transferase type-2 subunit beta-like - [512887909_XP_004922000.1] | 1.13227446 | 0.4575407 |
|  | PREDICTED: venom carboxylesterase-6-like - [512936246_XP_004933471.1] | 1.21099514 | 0.4575028 |
|  | PREDICTED: ubiquitin carboxyl-terminal hydrolase 30-like - [512895599_XP_004923711.1] | 1.22398122 | 0.4573137 |
|  | GH24281 - [195047250_XP_001992302.1] | 1.18529049 | 0.4570881 |
|  | PREDICTED: epsin-1-like - [512910596_XP_004927211.1] | 1.05056118 | 0.4569439 |
|  | PREDICTED: eukaryotic translation initiation factor 3 subunit A-like isoform X1 - [512917658_XP_004928941.1] | 1.16074495 | 0.4568853 |
|  | H+ transporting ATP synthase subunit d - [153791739_NP_001093279.1] | 1.13764093 | 0.4567134 |
|  | PREDICTED: ATP-dependent RNA helicase abstrakt-like isoform X1 - [512889324_XP_004922248.1] | #N/A | 0.45644 |
|  | snap-scaffold311_size215076-abinit-gene-1.6-mRNA-1 | #N/A | 0.4563753 |
|  | PREDICTED: interferon-related developmental regulator 2-like - [499005139_XP_004535505.1] | 1.41234695 | 0.4563151 |
|  | PREDICTED: histone H1-like, partial - [512939227_XP_004934189.1] | 1.07664938 | 0.4558621 |
|  | PREDICTED: presqualene diphosphate phosphatase-like - [512939536_XP_004934264.1] | 1.15127517 | 0.45583 |
|  | carboxyl/cholinesterase 7 precursor - [306518664_NP_001182393.1] | 1.07635479 | 0.4557972 |
|  | PREDICTED: malate dehydrogenase, mitochondrial-like - [512917790_XP_004928974.1] | 1.11694871 | 0.4555439 |
|  | ribosomal protein S16 - [112984394_NP_001037508.1] | 1.08571797 | 0.4553558 |
|  | PREDICTED: cytochrome c oxidase subunit 6A1, mitochondrial-like - [512931126_XP_004932231.1] | 1.18126681 | 0.455155 |
|  | PREDICTED: probable 39S ribosomal protein L49, mitochondrial-like - [512914405_XP_004928134.1] | 1.11170369 | 0.4551502 |
|  | Rab7 - [114051368_NP_001040368.1] | 1.12285854 | 0.4551431 |
|  | mRNA transport regulator 3 - [153791339_NP_001093283.1] | #N/A | 0.4550101 |
|  | AGAP006949-PA - [158286555_XP_308807.4] | 1.14627481 | 0.4550021 |
|  | GA21639 - [198476820_XP_001357493.2] | 1.12298949 | 0.4544692 |
|  | ATP-dependent RNA helicase p62 - [282158103_NP_001164095.1] | 1.07600855 | 0.4542024 |
|  | ribosomal protein L35A - [112984156_NP_001037243.1] | 1.15570667 | 0.4540565 |
|  | hypothetical protein AaeL_AAEL011289 - [157128966_XP_001661571.1] | 1.13378952 | 0.4538114 |
|  | PREDICTED: LOW QUALITY PROTEIN: collagen alpha-1(II) chain-like - [499002463_XP_004534844.1] | #N/A | 0.4538022 |
|  | rRNA processing protein Ebp2 - [114052360_NP_001040242.1] | 1.2031383 | 0.4537791 |
|  | extracellular regulated MAP kinase - [112982894_NP_001036921.1] | 1.12543826 | 0.4537066 |
|  | PREDICTED: endoplasmic reticulum-Golgi intermediate compartment protein 3-like isoform X1 - [512919071_XP_004929286.1] | #N/A | 0.4536996 |
|  | PREDICTED: nesprin-2-like - [512927195_XP_004931274.1] | 1.13279313 | 0.4536154 |
|  | PREDICTED: probable hydroxyacid-oxoacid transhydrogenase, mitochondrial-like - [383850997_XP_003701050.1] | 1.05256137 | 0.4534901 |
|  | GL19422 - [195147882_XP_002014903.1] | 1.15304743 | 0.453362 |
|  | PREDICTED: similar to heparan-alpha-glucosaminide N-acetyltransferase - [91078976_XP_974454.1] | 1.11279372 | 0.4530539 |
|  | PREDICTED: LOW QUALITY PROTEIN: PAX-interacting protein 1-like - [512918027_XP_004929030.1] | 1.10927357 | 0.4530038 |
|  | PREDICTED: abl interactor 2-like - [512896934_XP_004924030.1] | 1.14131063 | 0.4529585 |
|  | PREDICTED: soluble NSF attachment protein 29-like - [512917490_XP_004928899.1] | 1.21883581 | 0.4529109 |
|  | PREDICTED: zinc finger protein 34-like - [512901668_XP_004925192.1] | #N/A | 0.4527057 |
|  | glycosyl-phosphatidyl-inositol-anchored protein - [114051483_NP_001040301.1] | 1.11649537 | 0.4526885 |
|  | PREDICTED: mitochondrial elongation factor G2-like - [291240555_XP_002740187.1] | 1.13360903 | 0.4526223 |
|  | PREDICTED: uncharacterized protein LOC101737240 isoform X2 - [512892910_XP_004923049.1] | 1.0838474 | 0.4525197 |
|  | PREDICTED: enoyl-CoA hydratase domain-containing protein 2, mitochondrial-like - [512887979_XP_004922012.1] | 1.08659399 | 0.4524824 |
|  | PREDICTED: fatty-acid amide hydrolase 2-B-like - [512929292_XP_004931785.1] | 1.13382217 | 0.4523427 |
|  | PREDICTED: transcription factor A, mitochondrial-like - [512906934_XP_004926318.1] | 1.07799682 | 0.45234 |
|  | PREDICTED: pre-mRNA-processing factor 39-like - [512898902_XP_004924511.1] | 1.04801793 | 0.4522707 |
|  | olfactory receptor 3 - [112982950_NP_001036925.1] | #N/A | 0.4522537 |
|  | PREDICTED: LOW QUALITY PROTEIN: glucosidase 2 subunit beta-like - [512924768_XP_004930676.1] | 1.18066002 | 0.4522504 |
|  | PREDICTED: uncharacterized protein C3orf33-like isoform X1 - [512894103_XP_004923350.1] | 1.1447491 | 0.452148 |
|  | PREDICTED: RNA-binding protein 45-like - [512916655_XP_004928692.1] | 1.26766667 | 0.4517938 |
|  | PREDICTED: Niemann-Pick disease, type C1-like - [291221448_XP_002730736.1] | 1.05810366 | 0.4517593 |
|  | PREDICTED: atlastin-like isoform X1 - [512903844_XP_004925717.1] | 1.15313814 | 0.4515815 |
|  | PREDICTED: CCR4-NOT transcription complex subunit 6-like - [345486629_XP_001605640.2] | #N/A | 0.4511643 |
|  | PREDICTED: slit homolog 1 protein-like - [512925575_XP_004930872.1] | #N/A | 0.4511106 |
|  | PREDICTED: uncharacterized protein LOC101740268 isoform X1 - [512896586_XP_004923946.1] | 1.09614606 | 0.4510718 |
|  | PREDICTED: 28S ribosomal protein S9, mitochondrial-like - [512889844_XP_004922386.1] | 1.14703021 | 0.4504515 |
|  | Der1-like domain family member 1 - [114051465_NP_001040297.1] | #N/A | 0.4503161 |
|  | PREDICTED: kinesin-like protein KIF23-like - [340725041_XP_003400883.1] | 1.07534597 | 0.4501997 |
|  | GJ18233 - [195398015_XP_002057620.1] | 1.11030896 | 0.4501854 |
|  | PREDICTED: uncharacterized protein LOC101738225 - [512925594_XP_004930877.1] | #N/A | 0.4501851 |
|  | ras-like protein 1 - [112983398_NP_001036973.1] | 1.02173488 | 0.4499352 |
|  | PREDICTED: ankyrin repeat, SAM and basic leucine zipper domain-containing protein 1-like - [512913293_XP_004927861.1] | 1.023756 | 0.449903 |
|  | NADH dehydrogenase-ubiquinone Fe-S protein 2 - [114051447_NP_001040366.1] | 1.18860935 | 0.449726 |
|  | PREDICTED: uncharacterized protein LOC101747197 - [512888732_XP_004922135.1] | 1.19203889 | 0.4497142 |
|  | PREDICTED: poly(A)-specific ribonuclease PARN-like domain-containing protein 1-like - [512924082_XP_004930511.1] | 1.07729675 | 0.4496397 |
|  | PREDICTED: mismatch repair endonuclease PMS2-like - [449662703_XP_002165510.2] | #N/A | 0.4494469 |
|  | PREDICTED: transmembrane emp24 domain-containing protein 2-like - [512917444_XP_004928888.1] | 1.13205133 | 0.4493937 |
|  | PREDICTED: aminomethyltransferase, mitochondrial-like - [512927436_XP_004931334.1] | 1.11423029 | 0.4493612 |
|  | F-actin capping protein beta subunit - [114051904_NP_001040434.1] | 1.18661008 | 0.4491317 |
|  | NAD-dependent malic enzyme, mitochondrial precursor - [170594758_XP_001902120.1] | 1.1268973 | 0.4490997 |
|  | PREDICTED: solute carrier family 25 member 35-like - [512924014_XP_004930494.1] | 1.01930837 | 0.4490468 |
|  | PREDICTED: cirhin-like - [512928155_XP_004931513.1] | 1.16199932 | 0.4489459 |
|  | PREDICTED: vacuolar protein sorting-associated protein 26-like - [512893176_XP_004923116.1] | 1.17155465 | 0.4488783 |
|  | PREDICTED: activating signal cointegrator 1 complex subunit 1-like - [512926037_XP_004930985.1] | #N/A | 0.4487488 |
|  | AGAP001879-PA - [347966738_XP_321188.4] | 1.06865022 | 0.4487198 |
|  | 40S ribosomal protein S14 - [112982701_NP_001037114.1] | 1.0950402 | 0.4486789 |
|  | ribosomal protein L7Ae - [114052793_NP_001040276.1] | 1.109317 | 0.4482707 |
|  | PREDICTED: cleft lip and palate transmembrane protein 1-like protein-like isoform X1 - [512931691_XP_004932365.1] | 1.10683748 | 0.448238 |
|  | PREDICTED: 39S ribosomal protein L14, mitochondrial-like - [512915273_XP_004928349.1] | 1.13757589 | 0.4481488 |
|  | PREDICTED: 28S ribosomal protein S29, mitochondrial-like - [512906644_XP_004926248.1] | 1.13067538 | 0.4481367 |
|  | PREDICTED: large subunit GTPase 1 homolog isoform X1 - [512934011_XP_004932931.1] | #N/A | 0.4478858 |
|  | 60S ribosomal protein L17 - [112984422_NP_001037165.1] | 1.1047431 | 0.4478682 |
|  | putative signal recognition particle 54 kDa protein - [148298873_NP_001091776.1] | 1.11518004 | 0.4477271 |
|  | PREDICTED: tRNA-dihydrouridine(47) synthase [NAD(P)(+)]-like - [512937099_XP_004933683.1] | 1.13413328 | 0.4476455 |
|  | ribosomal protein L27 - [112984216_NP_001037235.1] | 1.17171162 | 0.4474351 |
|  | PREDICTED: ATP synthase mitochondrial F1 complex assembly factor 2-like isoform X1 - [512925901_XP_004930951.1] | 1.16170093 | 0.4473126 |
|  | PREDICTED: serine/threonine-protein kinase PLK1-like isoform X1 - [524875083_XP_005094272.1] | 1.09829689 | 0.4473094 |
|  | wolfram syndrome 1 - [170055009_XP_001863389.1] | #N/A | 0.4472996 |
|  | PREDICTED: striatin-3-like isoform X1 - [512919848_XP_004929485.1] | 1.12955673 | 0.4472098 |
|  | snap-scaffold1495_size74011-abinit-gene-0.7-mRNA-1 | #N/A | 0.4471977 |
|  | ras GTPase-activating protein 1 - [170033038_XP_001844386.1] | #N/A | 0.4469897 |
|  | PREDICTED: succinate dehydrogenase assembly factor 2-B, mitochondrial-like isoform X2 - [512913506_XP_004927913.1] | 1.10973743 | 0.4466444 |
|  | PREDICTED: peptidyl-prolyl cis-trans isomerase FKBP8-like isoform X1 - [512889921_XP_004922407.1] | 1.17211652 | 0.4464388 |
|  | PREDICTED: WD repeat-containing protein 75-like - [512930699_XP_004932124.1] | 1.17833021 | 0.4463458 |
|  | tRNA pseudouridine synthase D - [170027628_XP_001841699.1] | 1.17345173 | 0.4461702 |
|  | PREDICTED: transmembrane protein nessy-like - [512938134_XP_004933932.1] | 1.17638424 | 0.4460674 |
|  | maker-scaffold7533_size7858-snap-gene-0.6-mRNA-1 | #N/A | 0.4459604 |
|  | PREDICTED: GDP-Man:Man(3)GlcNAc(2)-PP-Dol alpha-1,2-mannosyltransferase-like, partial - [512937866_XP_004933869.1] | 1.1197861 | 0.4458804 |
|  | PREDICTED: DNA-directed RNA polymerase III subunit RPC4-like - [512904785_XP_004925782.1] | 1.14288468 | 0.4457003 |
|  | heat shock protein - [170050377_XP_001861262.1] | 1.15375065 | 0.4456705 |
|  | PREDICTED: ankyrin repeat and LEM domain-containing protein 2-like - [512902684_XP_004925434.1] | 1.24589787 | 0.4452126 |
|  | PREDICTED: phosphatidylserine decarboxylase proenzyme-like - [512889180_XP_004922210.1] | 1.04778559 | 0.4451244 |
|  | PREDICTED: jerky protein homolog-like - [328716033_XP_003245816.1] | #N/A | 0.44491 |
|  | vacuolar protein sorting 29 - [114053067_NP_001040505.1] | 1.09320407 | 0.4445947 |
|  | PREDICTED: DNA-directed RNA polymerases I and III subunit RPAC1-like - [512899076_XP_004924554.1] | 1.08978245 | 0.4445234 |
|  | beta-actin, partial - [312106932_XP_003150813.1] | 1.11898501 | 0.4444048 |
|  | PREDICTED: protein misato-like isoform X1 - [512930039_XP_004931970.1] | 1.10388378 | 0.4442615 |
|  | PREDICTED: uncharacterized protein LOC101736108 - [512928324_XP_004931551.1] | 1.15592415 | 0.4442271 |
|  | PREDICTED: putative ATP synthase subunit f, mitochondrial-like - [512887786_XP_004921980.1] | 1.13844322 | 0.4437449 |
|  | PREDICTED: nesprin-1-like - [512920041_XP_004929533.1] | 1.08456977 | 0.4436631 |
|  | GK16828 - [195428092_XP_002062108.1] | 1.06523916 | 0.4436437 |
|  | PREDICTED: DNA-directed RNA polymerases I, II, and III subunit RPABC1-like - [512935950_XP_004933401.1] | 1.14010249 | 0.4435894 |
|  | PREDICTED: syntaxin-18-like - [512901098_XP_004925051.1] | 1.16123613 | 0.4435429 |
|  | PREDICTED: protein SCO1 homolog, mitochondrial-like - [512907862_XP_004926548.1] | 1.09930745 | 0.4433308 |
|  | PREDICTED: probable Xaa-Pro aminopeptidase 3-like - [512916506_XP_004928657.1] | 1.0742642 | 0.4432786 |
|  | DEAD box ATP-dependent RNA helicase - [157126319_XP_001654592.1] | 1.11926167 | 0.4432328 |
|  | uncharacterized protein LOC778510 - [148298880_NP_001091805.1] | 1.16257726 | 0.4430907 |
|  | PREDICTED: uncharacterized protein C17orf59 homolog - [512928904_XP_004931694.1] | #N/A | 0.442709 |
|  | PREDICTED: host cell factor 1-like - [512892015_XP_004922826.1] | 1.16550188 | 0.4425577 |
|  | PREDICTED: probable complex I intermediate-associated protein 30, mitochondrial-like isoform X1 - [512887056_XP_004921859.1] | #N/A | 0.4425555 |
|  | PREDICTED: uncharacterized protein LOC101739229 - [512926311_XP_004931054.1] | 0.97357075 | 0.4423776 |
|  | hypothetical protein AaeL_AAEL001898 - [157125003_XP_001654205.1] | #N/A | 0.4422991 |
|  | ebna2 binding protein P100 - [170041583_XP_001848537.1] | 1.09233882 | 0.4422624 |
|  | PREDICTED: 60S ribosomal protein L4-like - [340725537_XP_003401125.1] | 1.14999352 | 0.4421785 |
|  | PREDICTED: nucleolar protein 11-like - [512897524_XP_004924174.1] | 1.2066573 | 0.4419522 |
|  | PREDICTED: ribosomal protein S6 kinase alpha-5-like isoform X1 - [512893319_XP_004923151.1] | 1.11494657 | 0.4417923 |
|  | PREDICTED: GTP-binding protein 1-like - [512885488_XP_004921603.1] | 1.22268473 | 0.441709 |
|  | stathmin - [114052122_NP_001040215.1] | 0.81846215 | 0.4416618 |
|  | PREDICTED: cytochrome c-like isoform X1 - [512929276_XP_004931781.1] | 1.25845719 | 0.4416335 |
|  | PREDICTED: protein RRP5 homolog - [512903893_XP_004925728.1] | 1.18055746 | 0.441541 |
|  | PREDICTED: protein cereblon homolog isoform X2 - [512899338_XP_004924619.1] | 1.17306819 | 0.4415221 |
|  | cell division cycle 2 - [112984382_NP_001037512.1] | 1.03734446 | 0.4414939 |
|  | PREDICTED: GPI transamidase component PIG-T-like - [512931535_XP_004932327.1] | 1.14856915 | 0.4414688 |
|  | ribosomal protein L18A - [112984310_NP_001037219.1] | 1.14136194 | 0.4413192 |
|  | PREDICTED: mesencephalic astrocyte-derived neurotrophic factor homolog - [512887792_XP_004921981.1] | 1.15512108 | 0.4412832 |
|  | PREDICTED: myelin expression factor 2-like isoform X6 - [512920586_XP_004929669.1] | 1.11138664 | 0.4411069 |
|  | PREDICTED: integrator complex subunit 4-like - [512892624_XP_004922977.1] | #N/A | 0.4405663 |
|  | PREDICTED: protein dpy-30 homolog - [512908977_XP_004926809.1] | 1.15608926 | 0.4405037 |
|  | PREDICTED: laminin subunit alpha-1-like - [512919221_XP_004929322.1] | #N/A | 0.4403879 |
|  | heat shock protein hsp23.7 precursor - [112983144_NP_001036942.1] | 1.27016159 | 0.4401562 |
|  | pyruvate dehydrogenase kinase - [167860162_NP_001108115.1] | 1.13110312 | 0.4401439 |
|  | GK14651 - [195434953_XP_002065466.1] | #N/A | 0.4401387 |
|  | PREDICTED: LOW QUALITY PROTEIN: uncharacterized protein KIAA0195-like - [340725210_XP_003400966.1] | 1.25450617 | 0.4399372 |
|  | acyl-CoA desaturase - [112983214_NP_001037018.1] | 1.15741863 | 0.4396688 |
|  | Protein UNC-43, isoform r - [392900436_NP_001255480.1] | 1.06029469 | 0.4395979 |
|  | GF20366 - [194762514_XP_001963379.1] | 1.07474864 | 0.4395037 |
|  | PREDICTED: rRNA 2'-O-methyltransferase fibrillarin-like - [512893346_XP_004923158.1] | 1.14316352 | 0.439492 |
|  | PREDICTED: separin-like - [512891836_XP_004922781.1] | 0.90208054 | 0.4394786 |
|  | snap-scaffold1012_size151494-abinit-gene-0.21-mRNA-1 | 1.04217597 | 0.4394747 |
|  | PREDICTED: dnaJ homolog subfamily B member 13-like - [512909282_XP_004926883.1] | #N/A | 0.4392999 |
|  | PREDICTED: neutral alpha-glucosidase AB-like isoform X1 - [512893246_XP_004923134.1] | 1.11726053 | 0.4392749 |
|  | PREDICTED: ras-related C3 botulinum toxin substrate 1-like - [512932473_XP_004932553.1] | 0.99489404 | 0.4391645 |
|  | PREDICTED: wolframin-like - [350403939_XP_003486957.1] | 1.18397926 | 0.439123 |
|  | PREDICTED: uncharacterized protein LOC100877796 - [383848791_XP_003700031.1] | #N/A | 0.4389855 |
|  | PREDICTED: thioredoxin domain-containing protein-like isoform X1 - [512896970_XP_004924038.1] | #N/A | 0.4388629 |
|  | PREDICTED: HEAT repeat containing 1-like - [291235035_XP_002737451.1] | 1.19478842 | 0.4386802 |
|  | PREDICTED: peptidyl-tRNA hydrolase 2, mitochondrial-like - [512891629_XP_004922730.1] | 1.1840415 | 0.4386695 |
|  | PREDICTED: similar to groucho CG8384-PA - [189234983_XP_968115.2] | 1.12350976 | 0.4386297 |
|  | ribosomal protein S30 - [112983904_NP_001037281.1] | 1.11770151 | 0.4386041 |
|  | ubiquinone biosynthesis protein COQ7-like protein - [151301088_NP_001093078.1] | 1.1353107 | 0.4384325 |
|  | PREDICTED: probable dolichyl pyrophosphate Glc1Man9GlcNAc2 alpha-1,3-glucosyltransferase-like - [512913313_XP_004927866.1] | 1.1947797 | 0.438424 |
|  | AGAP008864-PA, partial - [158299488_XP_552730.3] | #N/A | 0.4381628 |
|  | PREDICTED: putative helicase mov-10-B.1-like - [512886919_XP_004921836.1] | 0.9980948 | 0.4381334 |
|  | PREDICTED: vacuolar protein sorting-associated protein 33A-like - [512892510_XP_004922949.1] | #N/A | 0.4380916 |
|  | PREDICTED: mucin-17-like - [512920879_XP_004929743.1] | 1.21110476 | 0.4380889 |
|  | PREDICTED: 27 kDa hemolymph protein-like - [328713066_XP_001947666.2] | 1.09329696 | 0.4376758 |
|  | PREDICTED: protein PTCD3 homolog, mitochondrial-like - [512913033_XP_004927801.1] | 1.11533621 | 0.437573 |
|  | PREDICTED: nuclear RNA export factor 1-like - [512914662_XP_004928198.1] | 1.11098126 | 0.4373576 |
|  | PREDICTED: cap-specific mRNA (nucleoside-2'-O-)-methyltransferase 1-like - [512930954_XP_004932188.1] | 1.05109745 | 0.4373441 |
|  | PREDICTED: tetratricopeptide repeat protein 4-like - [512890872_XP_004922588.1] | 0.941077 | 0.4372516 |
|  | mobility group protein 1B - [151301198_NP_001093087.1] | 1.17501116 | 0.4369622 |
|  | PREDICTED: parafibromin-like - [512916036_XP_004928534.1] | 1.14879015 | 0.4368886 |
|  | PREDICTED: 1-acyl-sn-glycerol-3-phosphate acyltransferase gamma-like isoform X1 - [512929977_XP_004931956.1] | 1.11320187 | 0.4368525 |
|  | CRE-RPB-2 protein - [308487676_XP_003106033.1] | 1.21462622 | 0.4367732 |
|  | G protein alpha subunit Go isoform 2 - [290563180_NP_001166852.1] | 1.18447259 | 0.4367454 |
|  | PREDICTED: CCAAT/enhancer-binding protein zeta-like isoform X2 - [512893315_XP_004923150.1] | 1.19794915 | 0.4363893 |
|  | cdc2-related kinase - [112983598_NP_001037345.1] | 1.21617284 | 0.4363485 |
|  | NADH dehydrogenase isoform 1 - [114052452_NP_001040477.1] | 1.08142501 | 0.4361526 |
|  | PREDICTED: LOW QUALITY PROTEIN: unconventional myosin-Va-like - [512935838_XP_004933372.1] | #N/A | 0.4361126 |
|  | PREDICTED: dnaJ homolog subfamily C member 3-like - [512898714_XP_004924464.1] | 1.19217444 | 0.4360114 |
|  | PREDICTED: N-acetylgalactosaminyltransferase 6-like - [512913871_XP_004927999.1] | 1.09341997 | 0.4359207 |
|  | PREDICTED: insulin-like receptor isoform 1 - [345487749_XP_001606180.2] | #N/A | 0.4356044 |
|  | PREDICTED: probable peptide chain release factor C12orf65 homolog, mitochondrial-like - [512923594_XP_004930390.1] | 1.12224937 | 0.4355976 |
|  | PREDICTED: sterol regulatory element-binding protein 1-like isoform X1 - [512913344_XP_004927874.1] | 1.14528782 | 0.4354845 |
|  | PREDICTED: dihydropyrimidine dehydrogenase [NADP+]-like - [328706442_XP_001944849.2] | 1.07499505 | 0.4352481 |
|  | PREDICTED: DNA polymerase V-like - [512909378_XP_004926906.1] | #N/A | 0.4351917 |
|  | PREDICTED: aspartate--tRNA ligase, mitochondrial-like - [383858762_XP_003704868.1] | 1.17608133 | 0.4351869 |
|  | PREDICTED: NAD(P) transhydrogenase, mitochondrial-like isoform X1 - [512899568_XP_004924678.1] | #N/A | 0.4351863 |
|  | PREDICTED: zinc finger protein 62-like - [512893453_XP_004923185.1] | #N/A | 0.4350878 |
|  | PREDICTED: renin receptor-like isoform X1 - [512924395_XP_004930584.1] | 1.18221242 | 0.4350314 |
|  | PREDICTED: monocarboxylate transporter 5-like - [512925775_XP_004930921.1] | 0.99633891 | 0.4346728 |
|  | PREDICTED: uncharacterized protein LOC101738225 - [512925594_XP_004930877.1] | #N/A | 0.4345759 |
|  | PREDICTED: tRNA (cytosine(34)-C(5))-methyltransferase-like - [512890543_XP_004922531.1] | #N/A | 0.4344977 |
|  | PREDICTED: erythroid differentiation-related factor 1-like - [383851762_XP_003701400.1] | #N/A | 0.4344315 |
|  | PREDICTED: 3-hydroxyisobutyryl-CoA hydrolase, mitochondrial-like - [512926929_XP_004931208.1] | 1.0997496 | 0.4344307 |
|  | snap-scaffold27_size75870-abinit-gene-0.8-mRNA-1 | #N/A | 0.4343039 |
|  | snap-scaffold10894_size5178-abinit-gene-0.1-mRNA-1 | #N/A | 0.4342673 |
|  | PREDICTED: serine--tRNA ligase, mitochondrial-like isoform X1 - [512917258_XP_004928844.1] | 1.10980435 | 0.4341817 |
|  | PREDICTED: scaffold attachment factor B2-like - [512927388_XP_004931322.1] | 1.23856093 | 0.4340077 |
|  | PREDICTED: PRKCA-binding protein-like - [512894605_XP_004923470.1] | #N/A | 0.4334373 |
|  | uncharacterized protein LOC100862770 - [379698910_NP_001243926.1] | 1.04101686 | 0.4332318 |
|  | AGAP012366-PA, partial - [158300198_XP_551819.3] | 1.15898322 | 0.4331504 |
|  | beta-arrestin-1-like - [525343482_NP_001266347.1] | #N/A | 0.4329808 |
|  | PREDICTED: scaffold attachment factor B2-like - [512927388_XP_004931322.1] | 1.21888847 | 0.4329605 |
|  | protein-O-fucosyltransferase 1 precursor - [118918374_NP_001037061.2] | 1.12822335 | 0.432831 |
|  | DNA topoisomerase III beta-1 - [312079442_XP_003142175.1] | 1.19739855 | 0.4328207 |
|  | PREDICTED: probable ATP-dependent RNA helicase DDX23-like - [512892914_XP_004923050.1] | 1.16829358 | 0.4324385 |
|  | ribosomal protein L26 - [112984230_NP_001037233.1] | 1.12301542 | 0.4323288 |
|  | PREDICTED: uncharacterized protein LOC101738363 - [512931998_XP_004932437.1] | 1.15573624 | 0.4322243 |
|  | PREDICTED: mitochondrial import inner membrane translocase subunit Tim21-like - [512926403_XP_004931077.1] | #N/A | 0.4322112 |
|  | small GTP binding protein RAB5 - [112983262_NP_001037614.1] | 1.17630172 | 0.4321432 |
|  | mitochondrial ribosomal protein S25 - [114051479_NP_001040363.1] | 1.10333268 | 0.4321316 |
|  | PREDICTED: aminoacylase-1-like isoform X2 - [512902901_XP_004925488.1] | #N/A | 0.4319643 |
|  | multiprotein bridging factor 1 - [112984062_NP_001036824.1] | 1.11506513 | 0.4318402 |
|  | ATP-dependent RNA helicase - [157106032_XP_001649136.1] | 1.09465883 | 0.4315266 |
|  | PREDICTED: predicted protein-like - [291233668_XP_002736774.1] | 1.12103665 | 0.431323 |
|  | PREDICTED: transmembrane protein 177-like isoform X1 - [512903053_XP_004925524.1] | 1.22648201 | 0.4313229 |
|  | ribosomal protein S26 - [112983535_NP_001037520.1] | 1.10414183 | 0.4312984 |
|  | PREDICTED: 5-methylcytosine rRNA methyltransferase NSUN4-like - [512906849_XP_004926296.1] | 1.1376961 | 0.4306542 |
|  | PREDICTED: LOW QUALITY PROTEIN: midasin-like - [512890270_XP_004922489.1] | 1.15981509 | 0.4303673 |
|  | PREDICTED: uncharacterized protein LOC101450796 - [498988395_XP_004531342.1] | #N/A | 0.4303392 |
|  | PREDICTED: NADH dehydrogenase [ubiquinone] 1 beta subcomplex subunit 2, mitochondrial-like - [512926542_XP_004931111.1] | 1.16614518 | 0.4303085 |
|  | PREDICTED: retinol dehydrogenase 13-like isoform X1 - [512903874_XP_004925724.1] | 1.10827238 | 0.4302247 |
|  | PREDICTED: KDEL motif-containing protein 1-like isoform X1 - [512925865_XP_004930942.1] | #N/A | 0.4300593 |
|  | transport protein Sec61 beta subunit - [112983408_NP_001037632.1] | 1.10757713 | 0.4299974 |
|  | PREDICTED: xylosyltransferase oxt-like - [193603480_XP_001949441.1] | 1.17540237 | 0.4299334 |
|  | PREDICTED: stress-70 protein, mitochondrial isoform 1 - [72014569_XP_781277.1] | 1.13238945 | 0.4299043 |
|  | PREDICTED: nuclear pore complex protein Nup98-Nup96-like - [512889717_XP_004922353.1] | 1.19554278 | 0.4296859 |
|  | ribosomal protein S25 - [112983942_NP_001037275.1] | 1.15818754 | 0.4295676 |
|  | PREDICTED: plasma membrane calcium-transporting ATPase 3-like isoform X1 - [512905918_XP_004926066.1] | #N/A | 0.4295163 |
|  | PREDICTED: pentatricopeptide repeat-containing protein 1, mitochondrial-like - [512934289_XP_004932999.1] | 1.09658736 | 0.4293671 |
|  | PREDICTED: 28S ribosomal protein S30, mitochondrial-like - [512920532_XP_004929655.1] | 1.1740301 | 0.4293416 |
|  | PREDICTED: ubiquinol-cytochrome c reductase complex chaperone CBP3 homolog - [512901853_XP_004925234.1] | 1.06240263 | 0.4293395 |
|  | PREDICTED: segment polarity protein dishevelled homolog DVL-3 - [328782547_XP_392577.4] | 1.22374974 | 0.4291422 |
|  | PREDICTED: 39S ribosomal protein L3, mitochondrial-like isoform X1 - [512925873_XP_004930944.1] | 1.1385 | 0.4291365 |
|  | ribosomal protein L30 - [112984182_NP_001037239.1] | 1.14548597 | 0.4290717 |
|  | PREDICTED: uncharacterized protein LOC101745204 - [512935977_XP_004933407.1] | 1.20638461 | 0.4290058 |
|  | PREDICTED: DNA-directed RNA polymerase I subunit RPA2-like - [512934423_XP_004933029.1] | 1.16852045 | 0.4289882 |
|  | PREDICTED: uncharacterized protein LOC101740749 - [512932599_XP_004932585.1] | 1.13084318 | 0.428927 |
|  | PREDICTED: coiled-coil domain-containing protein 174-like isoform X1 - [499012445_XP_004537294.1] | #N/A | 0.4287194 |
|  | PREDICTED: mitochondrial import inner membrane translocase subunit TIM44-like - [512909037_XP_004926824.1] | 1.15986606 | 0.4285882 |
|  | AGAP003121-PA - [347969294_XP_562889.4] | 1.02427665 | 0.428513 |
|  | PREDICTED: aconitate hydratase, mitochondrial-like - [512932519_XP_004932565.1] | 1.10610867 | 0.4282175 |
|  | PREDICTED: G-protein-signaling modulator 2-like isoform 1 - [193606283_XP_001943489.1] | #N/A | 0.4281897 |
|  | PREDICTED: GTP-binding protein 10 homolog - [512885255_XP_004921564.1] | 1.14571714 | 0.4281241 |
|  | PREDICTED: flotillin-1-like - [512921692_XP_004929936.1] | #N/A | 0.427863 |
|  | PREDICTED: U3 small nucleolar ribonucleoprotein protein IMP3-like isoform X1 - [512916658_XP_004928693.1] | #N/A | 0.4275567 |
|  | ribosomal protein L36A - [148298804_NP_001091753.1] | 1.1326489 | 0.4274675 |
|  | PREDICTED: uncharacterized protein LOC101747067 - [512896780_XP_004923992.1] | #N/A | 0.4274077 |
|  | PREDICTED: transcription initiation factor TFIID subunit 10-like - [512907544_XP_004926469.1] | 1.6570149 | 0.4273265 |
|  | PREDICTED: protein turtle-like - [498996335_XP_004533334.1] | #N/A | 0.4270983 |
|  | PREDICTED: GPI mannosyltransferase 1-like - [512893179_XP_004923117.1] | 1.20282026 | 0.4268583 |
|  | PREDICTED: ATP-binding cassette sub-family B member 7, mitochondrial-like isoform X2 - [512905323_XP_004925915.1] | #N/A | 0.4265999 |
|  | dimethyladenosine transferase - [157103251_XP_001647892.1] | 1.11694457 | 0.426579 |
|  | PREDICTED: uncharacterized protein LOC101743259 - [512905604_XP_004925986.1] | #N/A | 0.4265583 |
|  | lola - [157121102_XP_001659826.1] | 1.19466199 | 0.4265112 |
|  | clathrin heavy chain - [219362829_NP_001136443.1] | 1.12986862 | 0.4264589 |
|  | GI24040 - [195117176_XP_002003125.1] | 1.12215148 | 0.4264134 |
|  | PREDICTED: transmembrane protein 223-like - [512928551_XP_004931605.1] | 1.17661676 | 0.4263789 |
|  | PREDICTED: proline-, glutamic acid- and leucine-rich protein 1-like - [512934057_XP_004932941.1] | 1.16262691 | 0.426348 |
|  | PREDICTED: actin-related protein 2-like isoform 3 - [383856508_XP_003703750.1] | 1.16903579 | 0.4263109 |
|  | PREDICTED: 2-oxoglutarate and iron-dependent oxygenase domain-containing protein 3-like - [512920859_XP_004929738.1] | 1.10953282 | 0.4260195 |
|  | scavenger receptor class B member 3 - [283945476_NP_001164650.1] | 1.08212527 | 0.4256229 |
|  | PREDICTED: coiled-coil domain-containing protein 58-like - [156550520_XP_001602291.1] | 1.13809266 | 0.4255336 |
|  | PREDICTED: transcription elongation factor SPT5-like - [156553711_XP_001600437.1] | 1.061632 | 0.4252681 |
|  | PREDICTED: LETM1 and EF-hand domain-containing protein anon-60Da, mitochondrial-like - [383856455_XP_003703724.1] | 1.12423057 | 0.4251262 |
|  | PREDICTED: DNA ligase 1-like isoform X1 - [512920369_XP_004929616.1] | #N/A | 0.4250498 |
|  | PREDICTED: nuclear RNA export factor 1-like - [512914662_XP_004928198.1] | 1.31611804 | 0.4249605 |
|  | PREDICTED: nucleolar GTP-binding protein 2-like - [512928274_XP_004931539.1] | 1.0353373 | 0.4248309 |
|  | PREDICTED: programmed cell death protein 4-like - [512901559_XP_004925164.1] | 1.11062016 | 0.4244551 |
|  | PREDICTED: protein CLP1 homolog - [328708204_XP_001946108.2] | 1.17733601 | 0.4242189 |
|  | snap-scaffold7072_size30312-abinit-gene-0.1-mRNA-1 | 1.0796605 | 0.4238118 |
|  | PREDICTED: phospholipase D3-like - [512886224_XP_004921723.1] | 1.16291191 | 0.4237257 |
|  | PREDICTED: 28S ribosomal protein S33, mitochondrial-like isoform X1 - [512887965_XP_004922010.1] | 1.26682541 | 0.423596 |
|  | PREDICTED: 28S ribosomal protein S15, mitochondrial-like - [512917359_XP_004928867.1] | #N/A | 0.4235145 |
|  | histone H2A-like protein 2 - [237648978_NP_001153666.1] | 1.14718616 | 0.4231737 |
|  | PREDICTED: E3 ubiquitin-protein ligase RBBP6-like isoform X2 - [512903068_XP_004925528.1] | #N/A | 0.4230979 |
|  | PREDICTED: eukaryotic translation initiation factor 3 subunit D-1-like - [498939730_XP_004521270.1] | 1.17318317 | 0.4229222 |
|  | ubiquinol-cytochrome c reductase core protein II - [163838684_NP_001106225.1] | 1.15442793 | 0.422896 |
|  | PREDICTED: ADP-ribosylation factor-related protein 1-like isoform X1 - [512885560_XP_004921614.1] | #N/A | 0.4228704 |
|  | uncharacterized protein LOC767623 - [148298701_NP_001091746.1] | 1.03846268 | 0.4228338 |
|  | PREDICTED: similar to CG6621 CG6621-PA - [91082317_XP_974328.1] | 1.15904664 | 0.4228089 |
|  | PREDICTED: lysosomal protein NCU-G1-B-like - [512901106_XP_004925053.1] | 1.01020758 | 0.4224973 |
|  | nuclear factor NF-kappa-B p110 subunit isoform 1 - [156255214_NP_001095935.1] | 1.0791911 | 0.4224778 |
|  | PREDICTED: zinc finger MYM-type protein 4-like - [512927657_XP_004931389.1] | 1.28578555 | 0.4221761 |
|  | AGAP004070-PA - [347971177_XP_309625.4] | 1.19793097 | 0.4218915 |
|  | mitochondrial import inner membrane translocase - [158635981_NP_001091841.1] | 1.21757563 | 0.4217885 |
|  | PREDICTED: la-related protein 1-like - [512889664_XP_004922339.1] | 1.16574847 | 0.4217478 |
|  | PREDICTED: putative helicase mov-10-B.1-like - [512886919_XP_004921836.1] | 0.9074068 | 0.4214257 |
|  | PREDICTED: putative ATP-dependent RNA helicase DHX30-like isoform 2 - [345489731_XP_003426215.1] | #N/A | 0.4212744 |
|  | PREDICTED: lamin-B receptor-like - [512933328_XP_004932764.1] | 1.17417662 | 0.4212466 |
|  | PREDICTED: similar to succinyl-coa synthetase beta chain - [91095067_XP_972655.1] | 1.1058733 | 0.4212017 |
|  | PREDICTED: uncharacterized protein LOC101745112 - [512934653_XP_004933084.1] | 1.16542877 | 0.4208805 |
|  | PREDICTED: sialin-like - [512906853_XP_004926297.1] | #N/A | 0.4208734 |
|  | PREDICTED: uncharacterized protein LOC101742876 - [512900331_XP_004924865.1] | 1.18678874 | 0.4208195 |
|  | PREDICTED: THO complex subunit 1-like - [512934007_XP_004932930.1] | #N/A | 0.4200085 |
|  | PREDICTED: organic cation transporter protein-like - [512920828_XP_004929730.1] | #N/A | 0.4199307 |
|  | PREDICTED: electron transfer flavoprotein-ubiquinone oxidoreductase, mitochondrial-like - [512902642_XP_004925425.1] | 1.10504412 | 0.4197223 |
|  | tetraspanin E118 - [114051159_NP_001040391.1] | #N/A | 0.4195807 |
|  | PREDICTED: uncharacterized protein LOC101745283 - [512903836_XP_004925715.1] | #N/A | 0.4194545 |
|  | PREDICTED: uncharacterized protein LOC101741928 - [512925502_XP_004930855.1] | 1.25328455 | 0.4186343 |
|  | coproporphirynogen oxidase - [114052330_NP_001040239.1] | 1.24543255 | 0.4184024 |
|  | PREDICTED: IQ and AAA domain-containing protein 1-like - [512935257_XP_004933230.1] | #N/A | 0.4180709 |
|  | mitochondrial ribosomal protein, L10, putative - [157123189_XP_001660051.1] | 1.18087123 | 0.417766 |
|  | PREDICTED: myoneurin-like - [512898227_XP_004924348.1] | 1.11937944 | 0.4176835 |
|  | PREDICTED: squamous cell carcinoma antigen recognized by T-cells 3-like - [383858361_XP_003704670.1] | 1.09563229 | 0.4174765 |
|  | putative mitochondrial ribosomal protein - [148298715_NP_001091803.1] | 1.16808374 | 0.4166277 |
|  | ribosomal protein L22 - [112984292_NP_001037225.1] | 1.15482342 | 0.4165295 |
|  | CRE-MOG-4 protein - [308503220_XP_003113794.1] | 1.01959855 | 0.4164815 |
|  | PREDICTED: cleavage stimulation factor subunit 1-like - [512907006_XP_004926336.1] | 1.24737661 | 0.4163928 |
|  | ALY - [112983092_NP_001037596.1] | 1.14663164 | 0.4158918 |
|  | PREDICTED: probable malonyl-CoA-acyl carrier protein transacylase, mitochondrial-like - [498965667_XP_004525631.1] | 1.15638509 | 0.4158635 |
|  | PREDICTED: uncharacterized protein LOC101744608 - [512903251_XP_004925574.1] | 1.18947695 | 0.415804 |
|  | ubiquitin-protein ligase - [157111695_XP_001651687.1] | 1.16665911 | 0.4155951 |
|  | PREDICTED: MAP/microtubule affinity-regulating kinase 3-like - [512893299_XP_004923146.1] | #N/A | 0.4154746 |
|  | PREDICTED: DNA topoisomerase 1-like - [350404638_XP_003487171.1] | 1.23843671 | 0.4153712 |
|  | PREDICTED: transmembrane protein 186-like - [498982617_XP_004529908.1] | 1.00892038 | 0.4153646 |
|  | ribosomal protein L10A - [112983523_NP_001037147.1] | 1.14890951 | 0.4148234 |
|  | PREDICTED: alpha-ketoglutarate-dependent dioxygenase alkB homolog 4-like isoform X1 - [512937824_XP_004933859.1] | 1.21617977 | 0.4147882 |
|  | PREDICTED: similar to G protein alpha 73B CG12232-PA - [91086719_XP_970742.1] | #N/A | 0.4146257 |
|  | PREDICTED: uncharacterized protein LOC101744707 - [512916857_XP_004928743.1] | 1.31320608 | 0.4145079 |
|  | PREDICTED: protein sel-1 homolog 1-like - [512894301_XP_004923396.1] | 1.16812419 | 0.413698 |
|  | PREDICTED: paraplegin-like - [512935106_XP_004933193.1] | 1.13141184 | 0.4136536 |
|  | ATP-dependent RNA Helicase - [256083123_XP_002577799.1] | 0.82322809 | 0.4136351 |
|  | PREDICTED: X-ray repair cross-complementing protein 5-like - [512889909_XP_004922404.1] | 1.11861954 | 0.4135894 |
|  | snap-scaffold2307_size83225-abinit-gene-0.22-mRNA-1 | #N/A | 0.4135011 |
|  | PREDICTED: nucleolar protein 9-like - [512905824_XP_004926041.1] | 1.10585794 | 0.4133285 |
|  | PREDICTED: mediator of RNA polymerase II transcription subunit 17-like - [499012483_XP_004537303.1] | 1.29658005 | 0.4132134 |
|  | PREDICTED: ras guanine nucleotide exchange factor L-like - [512930978_XP_004932194.1] | #N/A | 0.4131699 |
|  | PREDICTED: NADH dehydrogenase [ubiquinone] 1 alpha subcomplex subunit 7-like - [512937293_XP_004933732.1] | 1.1875339 | 0.4130966 |
|  | PREDICTED: cyclin-dependent kinase 12-like - [498946095_XP_004522314.1] | 1.12533608 | 0.4130447 |
|  | PREDICTED: uncharacterized protein LOC101745979 isoform X2 - [512929155_XP_004931754.1] | #N/A | 0.4128196 |
|  | PREDICTED: cysteine-rich with EGF-like domain protein 2-like - [512917105_XP_004928805.1] | 1.21669136 | 0.412742 |
|  | PREDICTED: protein canopy homolog 3-like - [512892652_XP_004922984.1] | 1.12702289 | 0.4123793 |
|  | snap-scaffold1347_size89861-abinit-gene-0.19-mRNA-1 | #N/A | 0.4117477 |
|  | PREDICTED: NADH dehydrogenase [ubiquinone] 1 alpha subcomplex subunit 4-like isoform X1 - [512908093_XP_004926598.1] | 1.17255506 | 0.411736 |
|  | PREDICTED: methyltransferase-like protein 17, mitochondrial-like - [512901421_XP_004925131.1] | 1.09681881 | 0.4116778 |
|  | glutamyl-tRNA amidotransferase subunit B - [114051415_NP_001040315.1] | 1.18377432 | 0.411319 |
|  | PREDICTED: ADP-ribosylation factor 6-like - [340721293_XP_003399058.1] | 1.2404807 | 0.4111951 |
|  | maker-scaffold3048_size45284-snap-gene-0.10-mRNA-1 | 1.00115835 | 0.4111254 |
|  | PREDICTED: E3 ubiquitin-protein ligase hyd-like - [512899654_XP_004924699.1] | #N/A | 0.4111172 |
|  | PREDICTED: probable proline--tRNA ligase, mitochondrial-like - [512907123_XP_004926365.1] | 1.08336821 | 0.410955 |
|  | PREDICTED: nicastrin-like - [512916853_XP_004928742.1] | 1.22150558 | 0.4107714 |
|  | snap-scaffold10161_size15440-abinit-gene-0.1-mRNA-1 | 0.99122277 | 0.4102095 |
|  | PREDICTED: palmitoyltransferase ZDHHC6-like - [512927155_XP_004931264.1] | #N/A | 0.410075 |
|  | GK17364 - [195428116_XP_002062120.1] | 1.07554722 | 0.4098607 |
|  | ecdysteroid-regulated 16 kDa protein precursor - [151301100_NP_001093080.1] | 0.97176741 | 0.4095868 |
|  | mitochondrial single-stranded DNA-binding protein - [114051211_NP_001040384.1] | 1.07449971 | 0.4092304 |
|  | PREDICTED: 28S ribosomal protein S28, mitochondrial-like - [512922525_XP_004930138.1] | 1.10863826 | 0.4087487 |
|  | PREDICTED: LOW QUALITY PROTEIN: nuclear factor related to kappa-B-binding protein-like - [512915558_XP_004928418.1] | 1.08799768 | 0.4084832 |
|  | PREDICTED: mitochondrial genome maintenance exonuclease 1-like isoform X1 - [512915246_XP_004928342.1] | 1.14875403 | 0.4083165 |
|  | snap-scaffold10074_size5310-abinit-gene-0.0-mRNA-1 | 1.12008304 | 0.4080866 |
|  | PREDICTED: titin-like - [512896112_XP_004923832.1] | #N/A | 0.4080644 |
|  | maker-scaffold2259_size89868-snap-gene-0.6-mRNA-1 | 1.16117643 | 0.4078145 |
|  | PREDICTED: rabenosyn-5-like - [512923516_XP_004930371.1] | #N/A | 0.4077024 |
|  | PREDICTED: ribosome biogenesis regulatory protein homolog - [512887748_XP_004921974.1] | 1.11049551 | 0.4073582 |
|  | DnaJ (Hsp40) homolog 7 - [255652887_NP_001157384.1] | 1.23766216 | 0.4072619 |
|  | PREDICTED: uncharacterized protein LOC101745112 - [512934653_XP_004933084.1] | 1.18819736 | 0.4072247 |
|  | serine protease inhibitor 8 precursor - [226342880_NP_001139702.1] | #N/A | 0.4071834 |
|  | PREDICTED: 28S ribosomal protein S31, mitochondrial-like - [512922048_XP_004930024.1] | 1.19958929 | 0.4070792 |
|  | PREDICTED: programmed cell death protein 4-like, partial - [512939843_XP_004934340.1] | 1.16758061 | 0.4066818 |
|  | PREDICTED: translation factor GUF1 homolog, mitochondrial-like - [512905174_XP_004925878.1] | 1.10613731 | 0.4066089 |
|  | snap-scaffold2259_size89868-abinit-gene-0.3-mRNA-1 | 1.1803777 | 0.406526 |
|  | PREDICTED: DNA topoisomerase 3-beta-1-like - [156551898_XP_001606777.1] | 1.27456903 | 0.4061935 |
|  | snap-scaffold4629_size18405-processed-gene-0.1-mRNA-1 | 1.10463548 | 0.4059644 |
|  | RNA-binding protein lark - [112983834_NP_001037293.1] | 1.04345953 | 0.4057708 |
|  | NADH-ubiquinone oxidoreductase Fe-S protein 7 - [114052144_NP_001040456.1] | 1.16599662 | 0.4057261 |
|  | PREDICTED: sortilin-related receptor-like - [328721377_XP_003247287.1] | 1.19588815 | 0.4053708 |
|  | ribosomal protein L11 - [112984334_NP_001037215.1] | 1.09970987 | 0.4050625 |
|  | PREDICTED: protoporphyrinogen oxidase-like - [512926905_XP_004931202.1] | 1.09989961 | 0.4050266 |
|  | PREDICTED: protein hook-like - [512923171_XP_004930288.1] | 1.20552341 | 0.404991 |
|  | NADH dehydrogenase ubiquinone Fe-S 8 - [114051372_NP_001040316.1] | #N/A | 0.4048039 |
|  | maker-scaffold863_size315357-snap-gene-1.4-mRNA-1 | #N/A | 0.4047485 |
|  | PREDICTED: NFU1 iron-sulfur cluster scaffold homolog, mitochondrial-like - [512929420_XP_004931816.1] | 1.12317547 | 0.4047088 |
|  | PREDICTED: uncharacterized protein LOC101738909 - [512933042_XP_004932693.1] | #N/A | 0.4045579 |
|  | GE17236 - [195479052_XP_002100747.1] |  | 0.4045452 |
|  | PREDICTED: probable 39S ribosomal protein L24, mitochondrial-like - [512936499_XP_004933534.1] | 1.09110024 | 0.4042818 |
|  | PREDICTED: ribosomal L1 domain-containing protein CG13096-like - [512905182_XP_004925880.1] | 1.18036041 | 0.4039946 |
|  | PREDICTED: DNA repair protein RAD50-like - [512931320_XP_004932273.1] | 1.07623226 | 0.4036453 |
|  | PREDICTED: WD repeat-containing protein 43-like - [512893342_XP_004923157.1] | 1.17280398 | 0.4034463 |
|  | PREDICTED: muscle M-line assembly protein unc-89-like - [512887224_XP_004921887.1] | 0.85334456 | 0.4031757 |
|  | snap-scaffold484_size156588-abinit-gene-0.14-mRNA-1 | #N/A | 0.402813 |
|  | PREDICTED: hypothetical protein LOC100648310 - [340709092_XP_003393148.1] | 1.09423035 | 0.4027988 |
|  | AGAP000252-PA - [347963460_XP_310873.5] | 1.17759299 | 0.4027077 |
|  | AGAP001783-PA - [347966573_XP_321284.5] | #N/A | 0.4026003 |
|  | PREDICTED: titin-like - [512892550_XP_004922959.1] | 1.2285051 | 0.4023139 |
|  | GG19021 - [194890874_XP_001977399.1] | 1.11101924 | 0.4018623 |
|  | PREDICTED: protein Spindly-like - [512899908_XP_004924762.1] | 1.06906351 | 0.4016137 |
|  | PREDICTED: zinc finger CCCH domain-containing protein 14-like - [512915626_XP_004928435.1] | 0.99003491 | 0.4012535 |
|  | PREDICTED: CCAAT/enhancer-binding protein zeta-like isoform X2 - [512893315_XP_004923150.1] | 1.2508606 | 0.4008621 |
|  | PREDICTED: THO complex subunit 5 homolog isoform X1 - [512930990_XP_004932197.1] | 1.13384022 | 0.4008163 |
|  | PREDICTED: disks large-associated protein 5-like isoform X1 - [512897496_XP_004924167.1] | 1.06940963 | 0.4003977 |
|  | PREDICTED: probable serine hydrolase-like - [512900339_XP_004924867.1] | 1.22288503 | 0.4003902 |
|  | PREDICTED: uncharacterized protein LOC101740589 - [512906938_XP_004926319.1] | 1.2241256 | 0.3999178 |
|  | PREDICTED: transcription initiation factor TFIID subunit 9-like - [512896610_XP_004923952.1] | 1.32487525 | 0.3997954 |
|  | PREDICTED: IQ motif containing GTPase activating protein 1-like - [291232521_XP_002736212.1] | 1.04543566 | 0.3996568 |
|  | PREDICTED: general transcription factor 3C polypeptide 5-like - [512925711_XP_004930905.1] | #N/A | 0.3993972 |
|  | PREDICTED: MICAL-like protein 1-like - [512924181_XP_004930536.1] | 1.18216457 | 0.3993145 |
|  | snap-scaffold1864_size83256-processed-gene-0.5-mRNA-1 | 1.24642276 | 0.3989839 |
|  | PREDICTED: LOW QUALITY PROTEIN: 39S ribosomal protein L37, mitochondrial-like - [512899200_XP_004924584.1] | 1.09576536 | 0.3988109 |
|  | PREDICTED: tRNA-dihydrouridine(20) synthase [NAD(P)+]-like - [512912545_XP_004927682.1] | 1.0208202 | 0.398451 |
|  | PREDICTED: E3 ubiquitin-protein ligase hyd-like - [512899654_XP_004924699.1] | 1.2466063 | 0.3981284 |
|  | PREDICTED: thioredoxin domain-containing protein-like isoform X1 - [512896970_XP_004924038.1] | 1.23515408 | 0.3979866 |
|  | PREDICTED: U3 small nucleolar RNA-interacting protein 2-like - [512887719_XP_004921969.1] | 1.09323616 | 0.3976078 |
|  | PREDICTED: SCY1-like protein 2 - [380027911_XP_003697658.1] | 1.23614128 | 0.397566 |
|  | PREDICTED: differentially expressed in FDCP 6-like - [512911292_XP_004927376.1] | 1.06162063 | 0.3974203 |
|  | PREDICTED: protein disulfide-isomerase A5-like isoform X2 - [512921265_XP_004929835.1] | 1.18808189 | 0.3969949 |
|  | PREDICTED: uncharacterized protein LOC101739573 - [512892975_XP_004923066.1] | 1.15429194 | 0.3968028 |
|  | PREDICTED: FAD-dependent oxidoreductase domain-containing protein 1-like - [512918992_XP_004929267.1] | 1.10766637 | 0.3967767 |
|  | GA29310 - [198466814_XP_002134706.1] | #N/A | 0.3967031 |
|  | anillin/rhotekin (rtkn) - [157119629_XP_001653425.1] | 1.05466852 | 0.396628 |
|  | hypothetical protein AaeL_AAEL005968 - [157111837_XP_001651749.1] | 1.14390625 | 0.3964696 |
|  | PREDICTED: mucin-5AC-like - [512918306_XP_004929101.1] | #N/A | 0.3961053 |
|  | PREDICTED: peptidyl-prolyl cis-trans isomerase FKBP8-like isoform X1 - [512889921_XP_004922407.1] | #N/A | 0.3960822 |
|  | maker-scaffold7230_size12627-snap-gene-0.5-mRNA-1 | 1.13649686 | 0.3960161 |
|  | PREDICTED: luciferin 4-monooxygenase-like - [512899232_XP_004924592.1] | #N/A | 0.3958361 |
|  | PREDICTED: choline-phosphate cytidylyltransferase B-like isoform X5 - [512892672_XP_004922989.1] | 0.87854436 | 0.3958078 |
|  | PREDICTED: uncharacterized protein LOC101740250 - [512927669_XP_004931392.1] | #N/A | 0.3955012 |
|  | PREDICTED: protein AAR2 homolog - [512937132_XP_004933692.1] | 1.17906171 | 0.3954862 |
|  | PREDICTED: peroxisomal membrane protein 11B-like - [512889947_XP_004922413.1] | 1.08714601 | 0.3953204 |
|  | PREDICTED: NADH dehydrogenase [ubiquinone] complex I, assembly factor 7 homolog isoform X1 - [512891359_XP_004922669.1] | 1.112153 | 0.3952813 |
|  | maker-scaffold7118_size11600-snap-gene-0.2-mRNA-1 | 1.1174778 | 0.3950598 |
|  | ribosomal protein L20 - [114050757_NP_001040151.1] | 1.11131526 | 0.3950361 |
|  | GA21641 - [125987465_XP_001357495.1] | 1.23009925 | 0.3949619 |
|  | PREDICTED: carboxy-terminal domain RNA polymerase II polypeptide A small phosphatase 1-like isoform X1 - [512918705_XP_004929197.1] | 1.47901489 | 0.3949504 |
|  | PREDICTED: uncharacterized protein LOC100881490 - [383853556_XP_003702288.1] | #N/A | 0.3947907 |
|  | PREDICTED: protein disulfide-isomerase A5-like isoform X2 - [512921265_XP_004929835.1] | 1.13389639 | 0.3947361 |
|  | PREDICTED: E3 SUMO-protein ligase PIAS3-like isoform X1 - [512890045_XP_004922440.1] | 1.22220041 | 0.3946873 |
|  | high temperature requirement protein A2 - [325197114_NP_001191410.1] | 1.09702315 | 0.3946369 |
|  | RNA polymerase Rpb1, domain 5 superfamily - [339237703_XP_003380406.1] | 1.16087702 | 0.3942087 |
|  | PREDICTED: histone deacetylase Rpd3 isoform 1 - [328788017_XP_394976.4] | 1.20531615 | 0.3938663 |
|  | PREDICTED: retinol dehydrogenase 14-like - [512901802_XP_004925225.1] | 1.15567569 | 0.3938624 |
|  | PREDICTED: 39S ribosomal protein L23, mitochondrial-like - [512920392_XP_004929621.1] | 1.14972446 | 0.3938142 |
|  | PREDICTED: dolichyl-diphosphooligosaccharide--protein glycosyltransferase subunit DAD1-like - [512911355_XP_004927391.1] | 1.08464398 | 0.3937589 |
|  | PREDICTED: similar to zinc knuckle domain protein - [189240756_XP_968999.2] | 1.18862607 | 0.393142 |
|  | PREDICTED: splicing factor, arginine/serine-rich 15-like - [512902964_XP_004925503.1] | 1.12602421 | 0.3930555 |
|  | tRNA selenocysteine 1-associated protein 1 - [112983244_NP_001037608.1] | 1.12024263 | 0.3928658 |
|  | autophagy 5-like - [215820600_NP_001135959.1] | #N/A | 0.3925882 |
|  | H2A histone family member V - [114053181_NP_001040529.1] | 1.12682887 | 0.3924081 |
|  | PREDICTED: RNA-binding protein 4.1-like isoform X1 - [512894313_XP_004923399.1] | 1.08308648 | 0.3922523 |
|  | maker-scaffold8069_size14790-snap-gene-0.6-mRNA-1 | #N/A | 0.3917005 |
|  | PREDICTED: transformation/transcription domain-associated protein-like - [390343732_XP_001181502.2] | 1.19093134 | 0.3912942 |
|  | PREDICTED: nucleolar protein 58-like - [340372413_XP_003384738.1] | 1.15941658 | 0.391021 |
|  | poly(A)-binding protein, putative - [157126845_XP_001660974.1] | #N/A | 0.3905204 |
|  | PREDICTED: zinc transporter foi-like, partial - [512939621_XP_004934286.1] | 1.07181346 | 0.390463 |
|  | PREDICTED: protein EMSY-like - [512926307_XP_004931053.1] | #N/A | 0.3904452 |
|  | PREDICTED: nucleolar protein 5A (56kDa with KKE/D repeat)-like - [291243323_XP_002741552.1] | 1.15442389 | 0.3904358 |
|  | PREDICTED: titin-like - [512920045_XP_004929534.1] | 1.09376678 | 0.390097 |
|  | glutathione S-transferase delta 3 - [112982796_NP_001037546.1] | 1.16673745 | 0.3899006 |
|  | snap-scaffold2373_size37043-abinit-gene-0.7-mRNA-1 | #N/A | 0.3896984 |
|  | PREDICTED: uncharacterized protein LOC101745951 - [512897822_XP_004924247.1] | 1.09759456 | 0.3895627 |
|  | ribosomal protein L6 - [112982812_NP_001037132.1] | 1.2114209 | 0.3891588 |
|  | PREDICTED: transducin beta-like protein 3-like - [193635377_XP_001943093.1] | 1.15777852 | 0.3889182 |
|  | snap-scaffold1292_size67328-abinit-gene-0.1-mRNA-1 | #N/A | 0.3889151 |
|  | peptidylprolyl isomerase B precursor - [114052472_NP_001040479.1] | 0.88525565 | 0.3887427 |
|  | translocon-associated protein gamma isoform 1 - [290563285_NP_001040330.1] | 1.15040521 | 0.3879705 |
|  | snap-scaffold361_size93452-abinit-gene-0.2-mRNA-1 | #N/A | 0.3877377 |
|  | PREDICTED: protein windbeutel-like - [512917230_XP_004928837.1] | 1.13244567 | 0.3872943 |
|  | GM25167 - [195326391_XP_002029912.1] | 1.25752643 | 0.3872874 |
|  | PREDICTED: zinc finger protein 91-like - [512901460_XP_004925140.1] | #N/A | 0.3871893 |
|  | PREDICTED: 39S ribosomal protein L46, mitochondrial-like - [512919884_XP_004929494.1] | 1.09809329 | 0.3869063 |
|  | PREDICTED: atrial natriuretic peptide-converting enzyme - [345495958_XP_001605459.2] | 1.21086349 | 0.3869006 |
|  | PREDICTED: similar to S23e ribosomal protein - [91076514_XP_973351.1] | 1.10354292 | 0.3868687 |
|  | GL11478 - [195150583_XP_002016230.1] | 1.12608854 | 0.3860877 |
|  | PREDICTED: similar to fetal alzheimer antigen, falz - [189240808_XP_001811424.1] | 1.17142987 | 0.3859638 |
|  | ubiquitin-conjugating enzyme E2 J1-like - [358031576_NP_001239599.1] | 1.14889401 | 0.3858506 |
|  | PREDICTED: uncharacterized protein LOC101742045 - [512890082_XP_004922450.1] | 1.09181395 | 0.3857213 |
|  | PREDICTED: alpha-2-macroglobulin receptor-associated protein-like isoform X1 - [512894952_XP_004923555.1] | 1.17275892 | 0.3857122 |
|  | PREDICTED: nitric oxide-associated protein 1-like - [512916725_XP_004928710.1] | 1.19296324 | 0.3856772 |
|  | hypothetical protein AaeL_AAEL013256 - [157135180_XP_001656560.1] | 1.17862007 | 0.3856044 |
|  | PREDICTED: uncharacterized protein LOC101739729 - [512902273_XP_004925335.1] | 1.11413885 | 0.3854957 |
|  | PREDICTED: LOW QUALITY PROTEIN: solute carrier family 25 member 40-like - [512903725_XP_004925688.1] | 1.13008668 | 0.3850991 |
|  | PREDICTED: NFX1-type zinc finger-containing protein 1-like isoform 2 - [328716874_XP_003246062.1] | 1.11270347 | 0.3849145 |
|  | PREDICTED: 39S ribosomal protein L38, mitochondrial-like - [512910657_XP_004927224.1] | 1.10522023 | 0.3848759 |
|  | PREDICTED: ribosome production factor 2 homolog - [512886386_XP_004921747.1] | 1.20441135 | 0.3846178 |
|  | PREDICTED: uncharacterized protein LOC101743790 - [512915818_XP_004928481.1] | 1.08476623 | 0.3843626 |
|  | PREDICTED: heat shock 70 kDa protein cognate 5-like, partial - [512912053_XP_004927562.1] | 1.09755637 | 0.3838507 |
|  | PREDICTED: something about silencing protein 10-like - [512931046_XP_004932211.1] | 1.19196472 | 0.3834169 |
|  | PREDICTED: protein TBRG4-like - [512902019_XP_004925272.1] | 1.15802186 | 0.3831228 |
|  | snap-scaffold198_size101588-abinit-gene-0.3-mRNA-1 | 1.14678552 | 0.3822854 |
|  | PREDICTED: NEDD8 ultimate buster 1-like - [512892023_XP_004922828.1] | #N/A | 0.3821444 |
|  | PREDICTED: interaptin-like - [512913423_XP_004927892.1] | 1.13050287 | 0.3820863 |
|  | PREDICTED: mitochondrial inner membrane protein OXA1L-like - [512920434_XP_004929631.1] | 1.09123944 | 0.3818036 |
|  | PREDICTED: selenoprotein M-like - [512885227_XP_004921560.1] | 1.07659506 | 0.3814097 |
|  | mitochondrial ribosomal protein L12 - [114053275_NP_001040523.1] | 1.06049777 | 0.3811887 |
|  | PREDICTED: CDGSH iron-sulfur domain-containing protein 3, mitochondrial-like isoform X1 - [512916765_XP_004928720.1] | 1.1358875 | 0.3810616 |
|  | PREDICTED: thiosulfate sulfurtransferase/rhodanese-like domain-containing protein 3-like - [512926752_XP_004931164.1] | 1.06557896 | 0.3808646 |
|  | G protein alpha subunit 73B-like protein - [261245109_NP_001159624.1] | #N/A | 0.3806365 |
|  | hypothetical protein Bm1_50075 - [170593461_XP_001901483.1] | 1.11540538 | 0.3803973 |
|  | PREDICTED: heterogeneous nuclear ribonucleoprotein 87F-like - [512924292_XP_004930561.1] | 1.05972781 | 0.3802003 |
|  | PREDICTED: elongation factor Tu, mitochondrial-like - [512898195_XP_004924340.1] | 1.17952726 | 0.3798767 |
|  | maker-scaffold3386_size23279-snap-gene-0.7-mRNA-1 | 1.09893547 | 0.3794255 |
|  | AGAP003882-PB - [347970832_XP_003436646.1] | #N/A | 0.3793068 |
|  | conserved hypothetical protein - [170062648_XP_001866761.1] | #N/A | 0.3792098 |
|  | PREDICTED: uncharacterized protein LOC101738671 - [512923409_XP_004930347.1] | 1.13583066 | 0.3788969 |
|  | PREDICTED: uncharacterized protein LOC101742398 - [512913980_XP_004928027.1] | 1.14683387 | 0.3788405 |
|  | PREDICTED: mitochondrial GTPase 1-like - [512904712_XP_004925764.1] | 1.10476636 | 0.3787136 |
|  | PREDICTED: uncharacterized protein LOC101738759 isoform X1 - [512922877_XP_004930223.1] | 0.87992825 | 0.3786953 |
|  | PREDICTED: C-myc promoter-binding protein-like - [512920906_XP_004929750.1] | #N/A | 0.378488 |
|  | PREDICTED: probable isocitrate dehydrogenase [NAD] subunit alpha, mitochondrial-like - [512901143_XP_004925063.1] | 1.09634645 | 0.3783368 |
|  | maker-scaffold6900_size13032-snap-gene-0.3-mRNA-1 | 1.27098201 | 0.3780559 |
|  | PREDICTED: transcription initiation factor TFIID subunit 3-like - [512893949_XP_004923310.1] | 1.17805289 | 0.3778514 |
|  | PREDICTED: 39S ribosomal protein L53, mitochondrial-like - [512922414_XP_004930114.1] | 1.08568798 | 0.3777686 |
|  | PREDICTED: golgi-specific brefeldin A-resistance guanine nucleotide exchange factor 1-like - [512896744_XP_004923983.1] | #N/A | 0.376819 |
|  | GE21775 - [195475256_XP_002089900.1] | 1.12629271 | 0.3755844 |
|  | PREDICTED: 15 kDa selenoprotein-like - [512913281_XP_004927858.1] | 1.04708469 | 0.3752523 |
|  | cyclin B homolog - [112983608_NP_001037343.1] | 0.99273427 | 0.3747025 |
|  | snap-scaffold6178_size11058-abinit-gene-0.0-mRNA-1 | 1.10584606 | 0.3746872 |
|  | PREDICTED: hypothetical protein LOC100643024 isoform 1 - [340728715_XP_003402663.1] | 1.1257327 | 0.3746301 |
|  | PREDICTED: chromosome-associated kinesin KIF4A-like - [499004406_XP_004535324.1] | 0.84806864 | 0.3742352 |
|  | snap-scaffold9297_size5576-abinit-gene-0.2-mRNA-1 | #N/A | 0.3741213 |
|  | histone H2B-like protein - [237648982_NP_001153668.1] | 1.14456501 | 0.3737034 |
|  | N-acetyltransferase 10 - [339238465_XP_003380787.1] | 1.19126233 | 0.3736738 |
|  | muscular protein 20 - [114052470_NP_001040476.1] | #N/A | 0.3735301 |
|  | PREDICTED: mitochondrial ribonuclease P protein 3-like - [512914736_XP_004928216.1] | 1.20891273 | 0.373229 |
|  | PREDICTED: ESF1 homolog isoform X1 - [512930522_XP_004932080.1] | 1.17615161 | 0.3731363 |
|  | PREDICTED: probable pseudouridine-5'-monophosphatase-like - [512892952_XP_004923060.1] | 1.18529293 | 0.3729559 |
|  | PREDICTED: probable uridine-cytidine kinase-like isoform 2 - [345493729_XP_003427142.1] | 1.22158783 | 0.3725424 |
|  | PREDICTED: DNA ligase 1-like - [512928727_XP_004931649.1] | 0.88175026 | 0.3718696 |
|  | PREDICTED: WD repeat-containing protein 74-like - [512901476_XP_004925144.1] | 1.1360975 | 0.3714099 |
|  | snap-scaffold9043_size7708-abinit-gene-0.1-mRNA-1 | #N/A | 0.3708137 |
|  | PREDICTED: sec1 family domain-containing protein 2-like - [512931447_XP_004932305.1] | 1.18979335 | 0.3704889 |
|  | PREDICTED: nucleosomal histone kinase 1-like - [512911689_XP_004927474.1] | 1.15021739 | 0.3702444 |
|  | PREDICTED: hypothetical protein LOC100740403 - [350399801_XP_003485643.1] | 1.20126599 | 0.3701392 |
|  | U2 small nuclear ribonucleoprotein A' - [114052002_NP_001040446.1] | 1.07993696 | 0.3700886 |
|  | maker-scaffold6903_size14286-snap-gene-0.6-mRNA-1 | 1.14907176 | 0.3698898 |
|  | PREDICTED: 60S ribosome subunit biogenesis protein NIP7 homolog - [512893238_XP_004923132.1] | 1.09507895 | 0.3697706 |
|  | ferritin precursor - [112982932_NP_001037580.1] | 1.15505247 | 0.3697124 |
|  | PREDICTED: ubiquinone biosynthesis monooxygenase COQ6-like - [512887166_XP_004921877.1] | 1.09679372 | 0.3695677 |
|  | PREDICTED: integrator complex subunit 6-B-like - [340719415_XP_003398149.1] | 1.20375524 | 0.3691721 |
|  | AGAP010767-PA, partial - [158298375_XP_318541.4] | 1.14221035 | 0.3682319 |
|  | ubiquinone biosynthesis protein COQ4 homolog, mitochondrial - [114051918_NP_001040440.1] | 1.13204173 | 0.3679035 |
|  | GK17392 - [195428208_XP_002062166.1] | #N/A | 0.3676364 |
|  | PREDICTED: uncharacterized protein LOC101746868 - [512927927_XP_004931457.1] | 1.07399004 | 0.3671862 |
|  | PREDICTED: probable ATP-dependent RNA helicase Dbp73D-like - [512931491_XP_004932316.1] | 1.18367612 | 0.3666899 |
|  | PREDICTED: peregrin-like - [340368978_XP_003383026.1] | #N/A | 0.3663599 |
|  | PREDICTED: 39S ribosomal protein L48, mitochondrial-like - [512903092_XP_004925534.1] | 1.20493287 | 0.3656898 |
|  | GH23993 - [195064226_XP_001996523.1] | 0.99098746 | 0.3655068 |
|  | PREDICTED: BMP-binding endothelial regulator protein-like - [512893761_XP_004923259.1] | #N/A | 0.364342 |
|  | PREDICTED: WD repeat-containing protein 18-like - [512902634_XP_004925423.1] | 1.16961694 | 0.3633101 |
|  | ribosomal protein L15 - [112984404_NP_001037162.1] | 1.177439 | 0.3632682 |
|  | PREDICTED: protein penguin-like - [512887729_XP_004921971.1] | 1.20716508 | 0.3629673 |
|  | PREDICTED: putative DNA helicase Ino80-like isoform 1 - [345491056_XP_001602268.2] | #N/A | 0.3629531 |
|  | PREDICTED: lysine-specific histone demethylase 1A-like - [512886356_XP_004921743.1] | #N/A | 0.3628978 |
|  | ribosomal protein L7 - [112982844_NP_001037135.1] | #N/A | 0.3619577 |
|  | PREDICTED: similar to CG5800 CG5800-PA - [91077478_XP_968425.1] | 1.1639592 | 0.3617403 |
|  | snap-scaffold7118_size11600-abinit-gene-0.1-mRNA-1 | 1.13701824 | 0.3616228 |
|  | fibroinase precursor - [164420679_NP_001037464.2] | 0.97671843 | 0.3616153 |
|  | snap-scaffold7311_size5067-abinit-gene-0.0-mRNA-1 | 1.23041521 | 0.3612879 |
|  | PREDICTED: protein Dr1-like isoform X1 - [512918940_XP_004929254.1] | 1.09189454 | 0.3608557 |
|  | PREDICTED: similar to RNA methyltransferase like 1 - [189241058_XP_968858.2] | #N/A | 0.3605449 |
|  | GI12813 - [195115772_XP_002002430.1] | 0.87608345 | 0.3598935 |
|  | PREDICTED: inhibitor of Bruton tyrosine kinase-like - [512890003_XP_004922428.1] | 1.16387317 | 0.3597819 |
|  | GH22027 - [195029563_XP_001987641.1] | 1.21362465 | 0.3595968 |
|  | PREDICTED: muscle M-line assembly protein unc-89-like - [512902858_XP_004925478.1] | 1.18337793 | 0.359595 |
|  | PREDICTED: tubulin-specific chaperone D-like - [512897110_XP_004924073.1] | 1.33170035 | 0.359464 |
|  | PREDICTED: probable ATP-dependent RNA helicase DDX27-like - [512906378_XP_004926181.1] | 1.18222647 | 0.3593449 |
|  | PREDICTED: 28S ribosomal protein S22, mitochondrial-like - [512923586_XP_004930388.1] | 1.09752086 | 0.3592622 |
|  | PREDICTED: peptidyl-tRNA hydrolase ICT1, mitochondrial-like - [512939861_XP_004934344.1] | 1.13025106 | 0.358755 |
|  | PREDICTED: uncharacterized protein LOC101740008 - [512911138_XP_004927341.1] | #N/A | 0.3587035 |
|  | PREDICTED: growth arrest and DNA damage-inducible proteins-interacting protein 1-like - [512913883_XP_004928002.1] | #N/A | 0.3579799 |
|  | GH12382 - [195039555_XP_001990902.1] | 1.012306 | 0.3568395 |
|  | PREDICTED: MAP kinase-activating death domain protein-like - [512914091_XP_004928055.1] | 1.03036866 | 0.3564415 |
|  | PREDICTED: 39S ribosomal protein L22, mitochondrial-like - [512913415_XP_004927890.1] | 1.15217364 | 0.3557896 |
|  | PREDICTED: probable H/ACA ribonucleoprotein complex subunit 1-like - [498985687_XP_004530665.1] | 1.10707806 | 0.3549575 |
|  | PREDICTED: protein 60A-like - [512919015_XP_004929272.1] | 1.06844313 | 0.3548721 |
|  | PREDICTED: 39S ribosomal protein L11, mitochondrial-like isoform X1 - [512887074_XP_004921862.1] | 1.1752532 | 0.3547539 |
|  | PREDICTED: ribosome-recycling factor, mitochondrial-like - [512915329_XP_004928363.1] | 0.94355537 | 0.3543847 |
|  | PREDICTED: probable tRNA threonylcarbamoyladenosine biosynthesis protein Osgepl1-like isoform X1 - [512929671_XP_004931880.1] | #N/A | 0.3543507 |
|  | PREDICTED: uncharacterized protein LOC101744136 - [512920377_XP_004929618.1] | 1.11063089 | 0.3538104 |
|  | maker-scaffold624_size153965-snap-gene-0.39-mRNA-1 | #N/A | 0.3536241 |
|  | maker-scaffold212_size100679-snap-gene-0.21-mRNA-1 | #N/A | 0.3528163 |
|  | PREDICTED: AP-2 complex subunit sigma-like - [340727423_XP_003402043.1] | 1.19609108 | 0.3526032 |
|  | PREDICTED: uncharacterized protein LOC101735752 - [512918382_XP_004929120.1] | 1.24890978 | 0.3524941 |
|  | PREDICTED: zinc finger protein 26-like - [512895664_XP_004923726.1] | #N/A | 0.3522278 |
|  | PREDICTED: LOW QUALITY PROTEIN: ELAV-like protein 2-like - [512905483_XP_004925955.1] | 1.09285686 | 0.3512245 |
|  | PREDICTED: periodic tryptophan protein 2 homolog - [512914373_XP_004928126.1] | 1.24467723 | 0.351135 |
|  | PREDICTED: organic cation transporter protein-like - [512910365_XP_004927154.1] | 0.80766063 | 0.3509638 |
|  | scavenger receptor type C precursor - [201023283_NP_001128387.1] | 1.00305671 | 0.3508821 |
|  | PREDICTED: tubulin-specific chaperone cofactor E-like protein-like - [512915219_XP_004928336.1] | #N/A | 0.3504808 |
|  | PREDICTED: protein Peter pan-like - [512901975_XP_004925261.1] | 1.3113336 | 0.3503545 |
|  | PREDICTED: 39S ribosomal protein L47, mitochondrial-like - [512930008_XP_004931963.1] | 1.11506387 | 0.3503386 |
|  | PREDICTED: mitogen-activated protein kinase kinase kinase kinase 4-like isoform X1 - [512915911_XP_004928503.1] | #N/A | 0.3503211 |
|  | PREDICTED: muscle M-line assembly protein unc-89-like - [512902858_XP_004925478.1] | 1.20885334 | 0.3500586 |
|  | PREDICTED: similar to Probable nucleolar GTP-binding protein 1 - [91087227_XP_975491.1] | 1.15516694 | 0.3498452 |
|  | PREDICTED: nucleolar complex protein 4 homolog B-like - [512916609_XP_004928682.1] | 1.17807235 | 0.3497878 |
|  | snap-scaffold13678_size1693-abinit-gene-0.0-mRNA-1 | #N/A | 0.3492964 |
|  | PREDICTED: 39S ribosomal protein L19, mitochondrial-like - [512927199_XP_004931275.1] | 1.12933116 | 0.3488534 |
|  | PREDICTED: probable ATP-dependent RNA helicase kurz-like - [512917309_XP_004928856.1] | 1.16994825 | 0.3487974 |
|  | GD22602 - [195576920_XP_002078321.1] | 1.13528499 | 0.3487438 |
|  | mitochondrial ribosomal protein S12 - [114052733_NP_001040492.1] | #N/A | 0.3485205 |
|  | PREDICTED: cytochrome P450 CYP12A2-like isoform X1 - [512913741_XP_004927968.1] | #N/A | 0.3483653 |
|  | GL13384 - [195168862_XP_002025249.1] | 1.18110542 | 0.3482511 |
|  | PREDICTED: protein penguin-like - [512887729_XP_004921971.1] | 1.15660585 | 0.3477197 |
|  | PREDICTED: exosome complex exonuclease RRP44-like - [391337831_XP_003743268.1] | #N/A | 0.347393 |
|  | GK13906 - [195451637_XP_002073011.1] | 1.08103901 | 0.3471823 |
|  | AGAP003972-PA - [347971000_XP_318424.4] | 1.19129936 | 0.3471778 |
|  | DnaJ domain-containing protein - [114052917_NP_001040116.1] | 1.12677304 | 0.3465625 |
|  | PREDICTED: plasma membrane calcium-transporting ATPase 3-like isoform X5 - [512905934_XP_004926070.1] | #N/A | 0.3465574 |
|  | PREDICTED: uncharacterized protein LOC101746488 - [512920442_XP_004929633.1] | 1.11370433 | 0.3465373 |
|  | PREDICTED: 28S ribosomal protein S18b, mitochondrial-like - [512918976_XP_004929263.1] | 1.10296961 | 0.3464246 |
|  | GK19958 - [195432070_XP_002064049.1] | 1.07269067 | 0.3462329 |
|  | snap-scaffold13490_size6904-abinit-gene-0.0-mRNA-1 | 1.10212405 | 0.3460638 |
|  | transcriptional adaptor 3 - [114050821_NP_001040349.1] | 1.14879349 | 0.3458236 |
|  | PREDICTED: 39S ribosomal protein L9, mitochondrial-like - [512898914_XP_004924514.1] | 1.14757462 | 0.345673 |
|  | AGAP004557-PA - [118784646_XP_313855.3] | 1.28624437 | 0.3455174 |
|  | translation elongation factor g - [157127164_XP_001661064.1] | 1.1867284 | 0.3453961 |
|  | PREDICTED: LOW QUALITY PROTEIN: multiple PDZ domain protein-like - [512936747_XP_004933594.1] | #N/A | 0.3452455 |
|  | PREDICTED: putative ribosomal RNA methyltransferase NOP2-like - [512923828_XP_004930447.1] | 1.21927336 | 0.3452215 |
|  | ribosomal protein L31 - [148298875_NP_001091751.1] | 1.11483002 | 0.3450127 |
|  | PREDICTED: protein CWC15 homolog - [512919019_XP_004929273.1] | 1.15032042 | 0.3447244 |
|  | PREDICTED: von Willebrand factor A domain-containing protein 9-like - [512915753_XP_004928466.1] | 1.3240535 | 0.3445684 |
|  | PREDICTED: uncharacterized protein LOC101737848 isoform X2 - [512892360_XP_004922912.1] | #N/A | 0.3444517 |
|  | PREDICTED: dual specificity protein kinase TTK-like - [512917137_XP_004928813.1] | 1.17487231 | 0.3437731 |
|  | PREDICTED: E3 ubiquitin-protein ligase MARCH5-like - [512910765_XP_004927251.1] | 1.12936301 | 0.3436517 |
|  | PREDICTED: tail-anchored protein insertion receptor WRB-like - [512906900_XP_004926309.1] | 1.20683133 | 0.3431715 |
|  | syndecan binding protein - [114052500_NP_001040345.1] | 1.2760824 | 0.342741 |
|  | PREDICTED: headcase protein-like - [512919761_XP_004929464.1] | 0.97113552 | 0.3426612 |
|  | PREDICTED: probable ATP-dependent RNA helicase pitchoune-like - [512924540_XP_004930620.1] | 1.21508502 | 0.3425254 |
|  | PTB-associated splicing factor - [114326261_NP_001037408.2] | 1.14286936 | 0.3421852 |
|  | PREDICTED: neuropathy target esterase sws-like - [345494016_XP_001605101.2] | 0.9783694 | 0.3421506 |
|  | coiled-coil-helix-coiled-coil-helix domain-containing protein 2, mitochondrial-like - [153791332_NP_001093285.1] | 1.16522422 | 0.3417553 |
|  | PREDICTED: microspherule protein 1-like isoform X1 - [512931680_XP_004932363.1] | 1.25963553 | 0.3416751 |
|  | maker-scaffold1587_size64702-snap-gene-0.10-mRNA-1 | #N/A | 0.341476 |
|  | PREDICTED: mitochondrial folate transporter/carrier-like isoform X1 - [512931813_XP_004932393.1] | 1.13742485 | 0.3414229 |
|  | PREDICTED: mediator of RNA polymerase II transcription subunit 8-like isoform X1 - [512903156_XP_004925550.1] | 1.19483494 | 0.3412758 |
|  | PREDICTED: zinc finger matrin-type protein CG9776-like - [512907249_XP_004926395.1] | 1.16721252 | 0.3409769 |
|  | PREDICTED: nucleolar protein 12-like - [512934665_XP_004933087.1] | 1.17967974 | 0.3405157 |
|  | PREDICTED: 39S ribosomal protein L15, mitochondrial-like - [512926069_XP_004930993.1] | 1.06579633 | 0.3398674 |
|  | ribosomal protein S18 - [112984000_NP_001037269.1] | 1.0731235 | 0.3398484 |
|  | PREDICTED: carbonyl reductase [NADPH] 3-like - [512909199_XP_004926863.1] | 1.16047177 | 0.3394616 |
|  | PREDICTED: bromodomain-containing protein 7-like - [512906743_XP_004926270.1] | 1.0516121 | 0.3393273 |
|  | PREDICTED: uncharacterized protein PFB0145c-like - [512887399_XP_004921918.1] | #N/A | 0.3393073 |
|  | PREDICTED: nucleolar protein 12-like - [512932555_XP_004932574.1] | 1.25906284 | 0.3387237 |
|  | PREDICTED: chaoptin-like, partial - [512912473_XP_004927664.1] | 0.94903889 | 0.3384018 |
|  | PREDICTED: putative polypeptide N-acetylgalactosaminyltransferase 9-like isoform X1 - [498924609_XP_004517478.1] | 1.15859218 | 0.3381603 |
|  | PREDICTED: nucleolin 2-like - [512907524_XP_004926464.1] | 1.17206035 | 0.3380867 |
|  | PREDICTED: iron-sulfur protein NUBPL-like isoform X1 - [512916552_XP_004928667.1] | 1.22467208 | 0.3377498 |
|  | PREDICTED: 39S ribosomal protein L51, mitochondrial-like - [512916125_XP_004928556.1] | 1.12901342 | 0.3377297 |
|  | PREDICTED: RNA binding protein fox-1 homolog 2-like - [512900455_XP_004924896.1] | 1.1423838 | 0.3367702 |
|  | AGAP003051-PA - [118781711_XP_311819.3] | 1.34903576 | 0.3366103 |
|  | PREDICTED: probable 39S ribosomal protein L45, mitochondrial-like - [512904773_XP_004925779.1] | 1.19401322 | 0.3365834 |
|  | PREDICTED: uncharacterized protein LOC100214037 - [449669939_XP_002160793.2] | #N/A | 0.3363949 |
|  | KDM4 - [379698892_NP_001243917.1] | 1.37569945 | 0.3355818 |
|  | GI14709 - [195131917_XP_002010390.1] | 1.14629511 | 0.3352543 |
|  | gp150 protein - [170038512_XP_001847093.1] | 0.73002118 | 0.334985 |
|  | NADH dehydrogenase (ubiquinone) 1 alpha subcomplex, 13 - [114051728_NP_001040176.1] | 1.21834824 | 0.3346255 |
|  | PREDICTED: actin-binding protein IPP-like - [512900268_XP_004924850.1] | #N/A | 0.3345327 |
|  | transmembrane BAX inhibitor motif-containing protein 5 - [112982814_NP_001037544.1] | 1.24964996 | 0.3341011 |
|  | PREDICTED: uncharacterized protein LOC101741203 - [512901199_XP_004925077.1] | 1.09018808 | 0.3340985 |
|  | PREDICTED: H/ACA ribonucleoprotein complex subunit 4-like - [512907580_XP_004926478.1] | 1.1506322 | 0.3339079 |
|  | TGF beta-inducible nuclear protein 1 - [112984142_NP_001037438.1] | 1.10993935 | 0.3336188 |
|  | PREDICTED: Down syndrome cell adhesion molecule-like protein Dscam2-like - [512894664_XP_004923484.1] | #N/A | 0.3327437 |
|  | PREDICTED: GTPase Era, mitochondrial-like - [512929901_XP_004931937.1] | 1.1140176 | 0.3324442 |
|  | GJ12492 - [195375331_XP_002046455.1] | #N/A | 0.3323712 |
|  | PREDICTED: putative mediator of RNA polymerase II transcription subunit 26-like - [512914123_XP_004928063.1] | 0.94747063 | 0.3322664 |
|  | PREDICTED: ATP-dependent Clp protease ATP-binding subunit clpX-like, mitochondrial-like - [340729247_XP_003402917.1] | 1.09174943 | 0.3317737 |
|  | PREDICTED: protein MAK16 homolog - [512914301_XP_004928108.1] | 1.15121046 | 0.3317256 |
|  | PREDICTED: ubiquitin carboxyl-terminal hydrolase 36-like - [512928201_XP_004931523.1] | 1.23702095 | 0.3310618 |
|  | monocarboxylate transporter - [170033020_XP_001844377.1] | 0.98345859 | 0.3297179 |
|  | PREDICTED: dimethyladenosine transferase 1, mitochondrial-like - [512910485_XP_004927183.1] | #N/A | 0.3294964 |
|  | PREDICTED: uncharacterized protein LOC101738149 - [512934772_XP_004933113.1] | 1.14164665 | 0.3293447 |
|  | PREDICTED: small subunit processome component 20 homolog - [499001224_XP_004534539.1] | 1.17684758 | 0.3290526 |
|  | PREDICTED: connectin-like - [512915242_XP_004928341.1] | 0.71274788 | 0.3278021 |
|  | PREDICTED: ubiquitin-like modifier-activating enzyme 5-like - [391342102_XP_003745362.1] | 1.17376204 | 0.3270553 |
|  | PREDICTED: 39S ribosomal protein L18, mitochondrial-like - [512917674_XP_004928945.1] | 1.08089876 | 0.3259712 |
|  | snap-scaffold7186_size6755-abinit-gene-0.0-mRNA-1 | #N/A | 0.3255839 |
|  | PREDICTED: eukaryotic translation initiation factor 4A-like - [291244806_XP_002742284.1] | #N/A | 0.3255489 |
|  | PREDICTED: probable 28S ribosomal protein S26, mitochondrial-like - [512904789_XP_004925783.1] | 1.12831943 | 0.3252694 |
|  | PREDICTED: alanine--tRNA ligase, mitochondrial-like - [512894448_XP_004923433.1] | 1.13469613 | 0.3252477 |
|  | conserved hypothetical protein - [170056267_XP_001863953.1] | 1.14577563 | 0.3250896 |
|  | PREDICTED: MATH and LRR domain-containing protein PFE0570w-like - [512897957_XP_004924280.1] | 1.19569646 | 0.3250033 |
|  | PREDICTED: putative methyltransferase NSUN5-like - [512886032_XP_004921694.1] | #N/A | 0.3240506 |
|  | PREDICTED: nuclear pore complex protein Nup98-Nup96-like - [499014827_XP_004537879.1] | 1.14036897 | 0.3240368 |
|  | mitochondrial ribosomal protein S5 - [114052779_NP_001040341.1] | 1.1152643 | 0.3235479 |
|  | PREDICTED: uncharacterized protein LOC101739435 - [512890007_XP_004922429.1] | 1.27360739 | 0.3226303 |
|  | PREDICTED: 28S ribosomal protein S11, mitochondrial-like - [512906656_XP_004926251.1] | 1.21343215 | 0.3223702 |
|  | PREDICTED: similar to 18 wheeler - [91076478_XP_972409.1] | #N/A | 0.3219406 |
|  | mitochondrial ribosomal protein L2 - [157117802_XP_001658945.1] | 1.13900537 | 0.3216878 |
|  | PREDICTED: YTH domain-containing protein 1-like - [512928087_XP_004931496.1] | 1.17038413 | 0.3216298 |
|  | PREDICTED: 28S ribosomal protein S35, mitochondrial-like - [512889360_XP_004922258.1] | 1.09967034 | 0.3215917 |
|  | PREDICTED: uncharacterized protein LOC100880872 - [383858908_XP_003704941.1] | 1.19676084 | 0.3215554 |
|  | PREDICTED: transcription initiation factor TFIID subunit 2-like isoform X2 - [498986370_XP_004530838.1] | 1.21963105 | 0.320527 |
|  | PREDICTED: ras-related protein Ral-a-like - [512895491_XP_004923684.1] | 1.23573547 | 0.3195152 |
|  | mitochondrial ribosomal protein S10 - [114051477_NP_001040362.1] | 1.10918827 | 0.3191433 |
|  | PREDICTED: failed axon connections-like isoform X2 - [512921315_XP_004929847.1] | #N/A | 0.318798 |
|  | PREDICTED: RNA-binding protein 28-like - [512891531_XP_004922707.1] | 1.15299921 | 0.3180723 |
|  | PREDICTED: uncharacterized protein LOC101747137 - [512925329_XP_004930812.1] | #N/A | 0.3178268 |
|  | PREDICTED: uncharacterized protein LOC101741349 - [512907241_XP_004926393.1] | #N/A | 0.3177858 |
|  | PREDICTED: uncharacterized protein K02A2.6-like - [390348191_XP_003726960.1] | #N/A | 0.3176787 |
|  | cyclin 3 - [158508578_NP_001103478.1] | 0.98607526 | 0.3173981 |
|  | AGAP011285-PA, partial - [158287301_XP_309361.4] | 1.16387156 | 0.3170943 |
|  | PREDICTED: transcriptional regulator ATRX homolog isoform X1 - [512935453_XP_004933279.1] | 1.07503064 | 0.316945 |
|  | PREDICTED: CCAAT/enhancer-binding protein zeta-like isoform X2 - [512893315_XP_004923150.1] | 1.24159901 | 0.316787 |
|  | ras-related protein 2 - [114051712_NP_001040327.1] | 1.06958537 | 0.3167389 |
|  | snap-scaffold6487_size8639-abinit-gene-0.1-mRNA-1 | 0.9915972 | 0.3159271 |
|  | PREDICTED: RRP15-like protein-like isoform X1 - [512894047_XP_004923336.1] | 1.17790429 | 0.3153496 |
|  | PREDICTED: 39S ribosomal protein L28, mitochondrial-like - [512913578_XP_004927931.1] | 1.16272431 | 0.3152209 |
|  | PREDICTED: 39S ribosomal protein L16, mitochondrial-like - [512893569_XP_004923214.1] | 1.14047579 | 0.314594 |
|  | PREDICTED: 39S ribosomal protein L30, mitochondrial-like - [512916346_XP_004928617.1] | #N/A | 0.3144946 |
|  | PREDICTED: vacuolar protein sorting-associated protein 4B-like isoform X1 - [524886022_XP_005099611.1] | 1.23973456 | 0.3144362 |
|  | GH19898 - [195029395_XP_001987558.1] | 1.08499977 | 0.3139233 |
|  | PREDICTED: retinol dehydrogenase 11-like isoform X1 - [512933873_XP_004932899.1] | 1.25289141 | 0.313854 |
|  | PREDICTED: uncharacterized protein LOC101746705 isoform X1 - [512907635_XP_004926492.1] | #N/A | 0.3135739 |
|  | PREDICTED: NADH dehydrogenase [ubiquinone] 1 alpha subcomplex subunit 6-like - [512930751_XP_004932137.1] | 1.11759164 | 0.3124744 |
|  | PREDICTED: LOW QUALITY PROTEIN: bifunctional lysine-specific demethylase and histidyl-hydroxylase NO66-like - [512937631_XP_004933811.1] | 1.20143938 | 0.3122799 |
|  | PREDICTED: homeobox protein prospero - [512886631_XP_004921790.1] | 0.76519906 | 0.3120143 |
|  | PREDICTED: dystroglycan-like isoform X1 - [512936001_XP_004933413.1] | 0.67723644 | 0.3114486 |
|  | PREDICTED: integrator complex subunit 1-like - [512904009_XP_004925757.1] | 1.18828608 | 0.3111269 |
|  | PREDICTED: WD repeat-containing protein 81-like isoform X1 - [498952059_XP_004523277.1] | #N/A | 0.3109611 |
|  | cytidine deaminase - [114051311_NP_001040110.1] | 1.0963325 | 0.3102525 |
|  | PREDICTED: histone-lysine N-methyltransferase SETD1B-like - [512918175_XP_004929068.1] |  | 0.3101581 |
|  | PREDICTED: probable RNA methyltransferase bin3-like - [512923910_XP_004930468.1] | 1.06754959 | 0.3086945 |
|  | PREDICTED: targeting protein for Xklp2-like - [512932169_XP_004932479.1] | 1.00837892 | 0.3083095 |
|  | PREDICTED: G-protein coupled receptor Mth2-like - [383856679_XP_003703835.1] | #N/A | 0.3082972 |
|  | ATP-dependent RNA helicase DDX54-like protein - [379699004_NP_001243975.1] | 1.19674284 | 0.3061247 |
|  | mitochondrial ribosomal protein L32 - [148298659_NP_001091811.1] | 1.07157399 | 0.3060161 |
|  | PREDICTED: putative ATP-dependent RNA helicase DHX30-like - [512898335_XP_004924374.1] | 1.16220309 | 0.3053685 |
|  | PREDICTED: uncharacterized protein LOC101735385 isoform X1 - [512912176_XP_004927592.1] | 1.14737895 | 0.3046821 |
|  | PREDICTED: uncharacterized protein LOC101741379 - [512896365_XP_004923893.1] | #N/A | 0.3038939 |
|  | AGAP007112-PA - [158286258_XP_308647.4] | 1.18925756 | 0.3034137 |
|  | PREDICTED: surfeit locus protein 6 homolog - [512916121_XP_004928555.1] | 1.19032909 | 0.3026121 |
|  | snap-scaffold229_size256595-processed-gene-1.8-mRNA-1 | #N/A | 0.3025075 |
|  | PREDICTED: probable ATP-dependent RNA helicase kurz-like isoform X1 - [498987649_XP_004531156.1] | 1.19169492 | 0.3012006 |
|  | snap-scaffold23071_size642-abinit-gene-0.0-mRNA-1 | 1.15286707 | 0.3006266 |
|  | meiotic recombination 11 - [112983948_NP_001036845.1] | 1.08028586 | 0.2999728 |
|  | PREDICTED: KRR1 small subunit processome component homolog - [512930542_XP_004932085.1] | 1.14741822 | 0.2999268 |
|  | chromatin modifying protein 1b - [114052338_NP_001040238.1] | 1.16498066 | 0.2998714 |
|  | PREDICTED: chaoptin-like, partial - [512912473_XP_004927664.1] | 0.96586698 | 0.2990264 |
|  | insulin receptor precursor - [112983268_NP_001037011.1] | 1.06601241 | 0.2983419 |
|  | PREDICTED: 39S ribosomal protein L52, mitochondrial-like - [512928563_XP_004931608.1] | 1.06151618 | 0.2977715 |
|  | PREDICTED: F-box/WD repeat-containing protein 7-like - [512935090_XP_004933189.1] | #N/A | 0.297533 |
|  | PREDICTED: nucleolar protein 6-like - [512901131_XP_004925060.1] | #N/A | 0.297483 |
|  | PREDICTED: NFX1-type zinc finger-containing protein 1-like - [390355660_XP_003728602.1] | 1.12293671 | 0.2958571 |
|  | GA26554 - [198452112_XP_002137422.1] | #N/A | 0.2958267 |
|  | PREDICTED: mitotic spindle assembly checkpoint protein MAD2B-like - [512896421_XP_004923907.1] | 1.04510483 | 0.2955281 |
|  | PREDICTED: innexin inx3-like isoform 1 - [345496464_XP_003427733.1] | 0.98398067 | 0.2939864 |
|  | AGAP003002-PA - [347969042_XP_311882.5] | 1.25186501 | 0.2938564 |
|  | Noki protein - [112983870_NP_001037283.1] | #N/A | 0.2934485 |
|  | PREDICTED: probable ATP-dependent RNA helicase DDX17-like isoform X1 - [512927903_XP_004931451.1] | 1.14815994 | 0.292737 |
|  | GE11101 - [195472959_XP_002088765.1] | 0.89600093 | 0.2924837 |
|  | PREDICTED: solute carrier family 12 member 1-like - [340367746_XP_003382414.1] | #N/A | 0.2916938 |
|  | repressor splicing factor 1 - [112983080_NP_001037670.1] | 1.14079187 | 0.2914399 |
|  | PREDICTED: 28S ribosomal protein S7, mitochondrial-like - [512911717_XP_004927481.1] | 1.15827831 | 0.291366 |
|  | PREDICTED: uncharacterized protein C1orf131 homolog - [512916462_XP_004928646.1] | 1.21679199 | 0.2913184 |
|  | PREDICTED: 39S ribosomal protein L17, mitochondrial-like - [512914839_XP_004928241.1] | 1.07020091 | 0.2907858 |
|  | PREDICTED: PCK2 protein-like - [291243463_XP_002741620.1] | 1.10319055 | 0.2902557 |
|  | PREDICTED: probable RNA 3'-terminal phosphate cyclase-like protein-like - [512895386_XP_004923660.1] | 1.06535498 | 0.2894806 |
|  | PREDICTED: lipase 3-like - [512907743_XP_004926519.1] | 1.01952121 | 0.2894211 |
|  | PREDICTED: 39S ribosomal protein L36, mitochondrial-like - [512928039_XP_004931484.1] | #N/A | 0.2888996 |
|  | PREDICTED: ATP-binding cassette sub-family G member 1 - [512920232_XP_004929581.1] | 0.94919221 | 0.2877359 |
|  | mitochondria-associated granulocyte macrophage CSF signaling molecule - [114052549_NP_001040256.1] | 1.14021011 | 0.2876262 |
|  | acyl-CoA desaturase 1 - [170057527_XP_001864523.1] | 1.11057546 | 0.2872483 |
|  | PREDICTED: pinin-like isoform X1 - [512903780_XP_004925701.1] | 1.13718626 | 0.2872156 |
|  | PREDICTED: protein FAM49B-like - [512928429_XP_004931576.1] | 0.83836838 | 0.2871293 |
|  | PREDICTED: nucleolar protein 14 homolog - [512934366_XP_004933016.1] | 1.18809402 | 0.2867014 |
|  | GH20712 - [195025710_XP_001986110.1] |  | 0.2861267 |
|  | AGAP008770-PA, partial - [158293558_XP_557938.3] | 1.13599643 | 0.2861167 |
|  | PREDICTED: 39S ribosomal protein L43, mitochondrial-like - [512901409_XP_004925128.1] | 1.10540421 | 0.2828946 |
|  | AGAP011084-PA, partial - [158287566_XP_309566.4] | 1.19319082 | 0.2824642 |
|  | PREDICTED: tyrosine-protein kinase transmembrane receptor ROR1-like - [380016444_XP_003692195.1] | 0.85518351 | 0.2823575 |
|  | GI13618 - [195128419_XP_002008661.1] | 1.16535053 | 0.2807448 |
|  | GH24866 - [195049074_XP_001992648.1] | 1.2449411 | 0.2805322 |
|  | PREDICTED: ATP-dependent RNA helicase p62-like - [512934172_XP_004932969.1] | 1.13666323 | 0.277883 |
|  | PREDICTED: uncharacterized protein LOC101738500 isoform X1 - [512929869_XP_004931929.1] | 1.24653388 | 0.2776573 |
|  | PREDICTED: transcription factor Dp-1-like isoform X1 - [512912539_XP_004927679.1] | #N/A | 0.2768508 |
|  | PREDICTED: dual specificity protein phosphatase CDC14A-like - [512909243_XP_004926874.1] | 1.03367178 | 0.2748039 |
|  | nucleolar GTP-binding protein - [157124441_XP_001654061.1] | 1.15326615 | 0.273929 |
|  | PREDICTED: forkhead box protein N3-like - [512915662_XP_004928444.1] | #N/A | 0.2738957 |
|  | PREDICTED: similar to Ab1-133 - [91079004_XP_974762.1] | #N/A | 0.2737518 |
|  | PREDICTED: 39S ribosomal protein L1, mitochondrial-like - [512909140_XP_004926849.1] | 1.15939101 | 0.2732169 |
|  | PREDICTED: transmembrane protein 70 homolog, mitochondrial-like - [512910993_XP_004927307.1] | 1.13889592 | 0.2702611 |
|  | isopentenyl-diphosphate delta isomerase - [291045214_NP_001040323.2] | 1.20234366 | 0.2700388 |
|  | PREDICTED: nucleolar protein 10-like - [512911884_XP_004927521.1] | 1.09663297 | 0.268919 |
|  | PREDICTED: actin-binding protein anillin-like - [512902405_XP_004925368.1] | #N/A | 0.2689039 |
|  | PREDICTED: LOW QUALITY PROTEIN: histone acetyltransferase p300-like - [512914330_XP_004928115.1] | #N/A | 0.2644745 |
|  | AGAP000461-PB - [347963867_XP_003437001.1] | 0.91716469 | 0.2626764 |
|  | bystin - [223890170_NP_001138800.1] | 1.13422073 | 0.2608625 |
|  | GH14499 - [195021668_XP_001985437.1] | 1.19980592 | 0.2595536 |
|  | PREDICTED: glucose dehydrogenase [acceptor]-like, partial - [512938329_XP_004933980.1] | #N/A | 0.2575899 |
|  | PREDICTED: C3 and PZP-like alpha-2-macroglobulin domain-containing protein 8-like - [512909835_XP_004927018.1] | #N/A | 0.2563877 |
|  | PREDICTED: nucleolar GTP-binding protein 2-like - [156552454_XP_001601194.1] | 1.20577836 | 0.2559943 |
|  | predicted protein - [170053867_XP_001862871.1] | 2.39922705 | 0.2541449 |
|  | PREDICTED: CCAAT/enhancer-binding protein zeta-like isoform X1 - [512893311_XP_004923149.1] | 1.23891431 | 0.2533268 |
|  | PREDICTED: LOW QUALITY PROTEIN: disks large homolog 5-like - [512898635_XP_004924445.1] | #N/A | 0.2527198 |
|  | PREDICTED: uncharacterized protein LOC101741971 - [512919415_XP_004929372.1] | 1.14100953 | 0.2500333 |
|  | PREDICTED: putative oxidoreductase GLYR1 homolog - [512896168_XP_004923846.1] | 1.21090855 | 0.2496831 |
|  | PREDICTED: U3 small nucleolar RNA-associated protein 6 homolog - [512933926_XP_004932911.1] | 1.14243172 | 0.2458506 |
|  | PREDICTED: probable ATP-dependent RNA helicase pitchoune-like - [345489122_XP_001604385.2] | 1.22087437 | 0.2449118 |
|  | PREDICTED: zinc finger protein on ecdysone puffs-like - [512934411_XP_004933026.1] | 1.09316881 | 0.2435271 |
|  | PREDICTED: uncharacterized protein LOC101736974 - [512905467_XP_004925951.1] | 1.16254238 | 0.2433738 |
|  | PREDICTED: ribosomal RNA-processing protein 8-like - [512907175_XP_004926378.1] | 1.12751423 | 0.2429743 |
|  | PREDICTED: chaoptin-like - [512897873_XP_004924259.1] | 0.83785083 | 0.2426419 |
|  | PREDICTED: RNA-binding protein 25-like - [512907155_XP_004926373.1] | 1.04627847 | 0.2421445 |
|  | PREDICTED: 39S ribosomal protein L21, mitochondrial-like - [512885313_XP_004921574.1] | 1.12897978 | 0.2414729 |
|  | PREDICTED: similar to AGAP011455-PA - [91084091_XP_968609.1] | #N/A | 0.2410407 |
|  | PREDICTED: transcription elongation factor SPT5-like - [383859710_XP_003705335.1] | #N/A | 0.240938 |
|  | snap-scaffold2117_size56248-abinit-gene-0.2-mRNA-1 | #N/A | 0.2379574 |
|  | jumonji/arid domain-containing protein - [256074107_XP_002573368.1] | 1.12449058 | 0.236851 |
|  | PREDICTED: uncharacterized protein LOC101744149 - [512933453_XP_004932795.1] | 1.10415926 | 0.2342398 |
|  | snap-scaffold2972_size79608-abinit-gene-0.21-mRNA-1 | 0.80763914 | 0.2335254 |
|  | farnesyl pyrophosphate syntase - [112983458_NP_001036889.1] | 0.89013377 | 0.2332684 |
|  | PREDICTED: RNA pseudouridylate synthase domain-containing protein 2-like - [512919307_XP_004929343.1] | #N/A | 0.2315461 |
|  | eukaryotic translation initiation factor 4E-binding protein 2 - [114050837_NP_001040409.1] | 0.94379413 | 0.2274931 |
|  | PREDICTED: LOW QUALITY PROTEIN: set1/Ash2 histone methyltransferase complex subunit ASH2-like - [512892269_XP_004922890.1] | 1.15250059 | 0.2261659 |
|  | PREDICTED: MPN domain-containing protein CG4751-like - [512926144_XP_004931012.1] | #N/A | 0.2258826 |
|  | PREDICTED: meckelin-like - [512890411_XP_004922511.1] | #N/A | 0.2208912 |
|  | maker-scaffold8131_size4958-snap-gene-0.2-mRNA-1 | #N/A | 0.2197426 |
|  | PREDICTED: uncharacterized protein LOC101736472 - [512892317_XP_004922902.1] | 1.15959187 | 0.2183726 |
|  | poly [ADP-ribose] polymerase - [256072936_XP_002572789.1] | 0.89015127 | 0.2178291 |
|  | kinesin-like protein Ncd - [187281809_NP_001119723.1] | 0.71543647 | 0.2152045 |
|  | prolyl 4-hydroxylase alpha subunit precursor - [112984520_NP_001037195.1] | 1.10514558 | 0.2150354 |
|  | uncharacterized protein LOC100141456 - [347360997_NP_001108406.1] | 1.14034643 | 0.2146947 |
|  | PREDICTED: plancitoxin-1-like - [350416059_XP_003490830.1] | #N/A | 0.2131703 |
|  | PREDICTED: innexin inx1-like - [512917157_XP_004928818.1] | #N/A | 0.2130499 |
|  | ced-6 protein - [295424202_NP_001171330.1] | 1.14593796 | 0.2095014 |
|  | PREDICTED: LOW QUALITY PROTEIN: 28S ribosomal protein S17, mitochondrial-like - [512889943_XP_004922412.1] | 1.12424131 | 0.2083501 |
|  | PREDICTED: uncharacterized protein LOC101736240 - [512923163_XP_004930286.1] | #N/A | 0.1821003 |
|  | PREDICTED: ribosomal RNA-processing protein 7 homolog A-like - [512897540_XP_004924178.1] | 1.08117298 | 0.1815608 |
|  | PREDICTED: A-kinase anchor protein 1, mitochondrial-like isoform X1 - [512900126_XP_004924816.1] | 1.02162067 | 0.1807433 |
|  | PREDICTED: DDB1- and CUL4-associated factor 13-like - [512919876_XP_004929492.1] | 1.20771922 | 0.1698032 |
|  | PREDICTED: RNA polymerase II elongation factor ELL-like - [512920940_XP_004929759.1] | #N/A | 0.1688389 |
|  | PREDICTED: deoxynucleotidyltransferase terminal-interacting protein 2-like - [512916434_XP_004928639.1] | 1.13455267 | 0.1662054 |
|  | PREDICTED: protein regulator of cytokinesis 1-like - [512914760_XP_004928222.1] | 0.59361366 | 0.1638466 |
|  | PREDICTED: similar to AGAP001153-PA - [91085387_XP_966471.1] | 1.22711439 | 0.1638058 |
|  | PREDICTED: similar to mediator complex - [189234764_XP_001815546.1] | 1.14077987 | 0.16003 |
|  | PREDICTED: fidgetin-like protein 1-like - [512885458_XP_004921598.1] | #N/A | 0.1592388 |
|  | PREDICTED: protein AATF-like - [512910689_XP_004927232.1] | 1.17215066 | 0.1553749 |
|  | PREDICTED: collagen alpha-2(IV) chain-like - [383858152_XP_003704566.1] | #N/A | 0.1489118 |
|  | PREDICTED: kinesin-like protein KIF18A-like - [512899853_XP_004924749.1] | 0.81070944 | 0.1475381 |
|  | PREDICTED: similar to CG4713 CG4713-PA - [189237243_XP_971461.2] | 1.01948304 | 0.0824469 |
|  | PREDICTED: indole-3-acetaldehyde oxidase-like - [512915424_XP_004928386.1] | #N/A | 0 |
|  | PREDICTED: uncharacterized protein LOC101735740 - [512907367_XP_004926424.1] | #N/A | 0 |
|  | snap-scaffold6272_size6239-abinit-gene-0.2-mRNA-1 | 11.5702636 | #N/A |
|  | PREDICTED: transmembrane protease serine 9-like - [512930252_XP_004932020.1] | 10.2052158 | #N/A |
|  | maker-scaffold379_size50259-snap-gene-0.22-mRNA-1 | 9.06734183 | #N/A |
|  | PREDICTED: coiled-coil domain-containing protein 40-like - [512888130_XP_004922038.1] | 6.81200757 | #N/A |
|  | sex-specific storage-protein 2 precursor - [124430725_NP_001037590.1] | 5.23208448 | #N/A |
|  | maker-scaffold595_size58831-snap-gene-0.5-mRNA-1 | 5.21580143 | #N/A |
|  | PREDICTED: hemocyte protein-glutamine gamma-glutamyltransferase-like - [512936208_XP_004933462.1] | 3.5989023 | #N/A |
|  | J domain-containing protein - [112983220_NP_001037016.1] | 3.43872827 | #N/A |
|  | antimicrobial protein 6Tox precursor - [112983312_NP_001037004.1] | 3.0761451 | #N/A |
|  | PREDICTED: RNA-binding protein 40-like - [512897520_XP_004924173.1] | 3.00022769 | #N/A |
|  | PREDICTED: polyserase-2-like - [512911221_XP_004927359.1] | 2.88853338 | #N/A |
|  | maker-scaffold10548_size2836-snap-gene-0.2-mRNA-1 | 2.41052086 | #N/A |
|  | PREDICTED: maternal protein tudor-like - [512928832_XP_004931676.1] | 2.37392982 | #N/A |
|  | GJ20366 - [195382811_XP_002050122.1] | 2.21752224 | #N/A |
|  | PREDICTED: similar to hyperpolarization-activated ion channel isoform 1 - [91087557_XP_966775.1] | 2.17127597 | #N/A |
|  | PREDICTED: regulator of microtubule dynamics protein 1-like isoform X3 - [512922893_XP_004930227.1] | 2.05542822 | #N/A |
|  | PREDICTED: early growth response protein 3-like - [512933177_XP_004932726.1] | 1.98729178 | #N/A |
|  | PREDICTED: solute carrier family 35 member F5-like, partial - [512890248_XP_004922485.1] | 1.95491905 | #N/A |
|  | PREDICTED: protein spinster-like isoform X2 - [512930633_XP_004932107.1] | 1.95486948 | #N/A |
|  | PREDICTED: acetylcholine receptor subunit beta-like 2-like - [498934165_XP_004519820.1] | 1.88468166 | #N/A |
|  | PREDICTED: protein lines-like - [512895796_XP_004923757.1] | 1.84127992 | #N/A |
|  | PREDICTED: inositol polyphosphate 5-phosphatase K-like - [512930439_XP_004932068.1] | 1.83642858 | #N/A |
|  | PREDICTED: inositol polyphosphate 5-phosphatase K-like - [512930439_XP_004932068.1] | 1.80880632 | #N/A |
|  | GD25020 - [195586140_XP_002082836.1] | 1.79450246 | #N/A |
|  | PREDICTED: UBA-like domain-containing protein 1-like isoform X2 - [512928020_XP_004931479.1] | 1.7834398 | #N/A |
|  | ecdysone 20-hydroxylase - [163838670_NP_001106219.1] | 1.76922437 | #N/A |
|  | PREDICTED: DDB1- and CUL4-associated factor 7-like - [512920026_XP_004929529.1] | 1.76000136 | #N/A |
|  | lysosomal-associated transmembrane protein - [112983148_NP_001037039.1] | 1.74712399 | #N/A |
|  | chitinase-like protein EN03 precursor - [112983920_NP_001036847.1] | 1.73662622 | #N/A |
|  | snap-scaffold769_size84986-abinit-gene-0.7-mRNA-1 | 1.72785726 | #N/A |
|  | PREDICTED: uncharacterized protein LOC101744418 - [512920753_XP_004929711.1] | 1.69551868 | #N/A |
|  | PREDICTED: single-stranded DNA-binding protein 3-like - [512912247_XP_004927608.1] | 1.69355666 | #N/A |
|  | snap-scaffold1153_size97057-abinit-gene-0.4-mRNA-1 | 1.69043662 | #N/A |
|  | PREDICTED: protein PIEZO2-like - [345483322_XP_001602864.2] | 1.65608498 | #N/A |
|  | snap-scaffold8015_size4440-abinit-gene-0.3-mRNA-1 | 1.64599093 | #N/A |
|  | PREDICTED: mannosyl-oligosaccharide alpha-1,2-mannosidase isoform A-like - [512926658_XP_004931141.1] | 1.64583592 | #N/A |
|  | PREDICTED: sodium channel protein Nach-like - [350414973_XP_003490491.1] | 1.64463504 | #N/A |
|  | PREDICTED: ER degradation-enhancing alpha-mannosidase-like 2-like - [383864037_XP_003707486.1] | 1.63744452 | #N/A |
|  | tweek, isoform G - [386769723_NP_001188824.2] | 1.63440943 | #N/A |
|  | PREDICTED: fizzy-related protein homolog - [512916466_XP_004928647.1] | 1.60790668 | #N/A |
|  | PREDICTED: inositol polyphosphate 5-phosphatase K-like - [512930439_XP_004932068.1] | 1.57720644 | #N/A |
|  | chitinase-like protein EN03 precursor - [112983920_NP_001036847.1] | 1.55565092 | #N/A |
|  | PREDICTED: mitochondrial sodium/hydrogen exchanger 9B2-like isoform X1 - [512919007_XP_004929270.1] | 1.52902913 | #N/A |
|  | PREDICTED: LOW QUALITY PROTEIN: transcription initiation factor TFIID subunit 4-like - [512900696_XP_004924953.1] | 1.52747765 | #N/A |
|  | snap-scaffold406_size68945-abinit-gene-0.11-mRNA-1 | 1.51068631 | #N/A |
|  | PREDICTED: mitogen-activated protein kinase kinase kinase 4-like - [512908185_XP_004926619.1] | 1.51064621 | #N/A |
|  | snap-scaffold695_size197564-abinit-gene-0.37-mRNA-1 | 1.51048564 | #N/A |
|  | PREDICTED: hypothetical protein LOC100571634 - [328698807_XP_003240738.1] | 1.50890572 | #N/A |
|  | PREDICTED: TBC1 domain family member 23-like isoform X1 - [498969631_XP_004526608.1] | 1.50516066 | #N/A |
|  | PREDICTED: RNA-binding protein 26-like - [512925443_XP_004930841.1] | 1.49321119 | #N/A |
|  | PREDICTED: thioredoxin domain-containing protein 11-like - [512909931_XP_004927042.1] | 1.48611753 | #N/A |
|  | PREDICTED: DTW domain-containing protein 2-like - [512920404_XP_004929624.1] | 1.48509435 | #N/A |
|  | PREDICTED: similar to scavenger receptor cysteine-rich protein isoform 1 - [91080607_XP_967476.1] | 1.4832944 | #N/A |
|  | PREDICTED: transducin-like enhancer protein 4 - [345497371_XP_001601479.2] | 1.48188627 | #N/A |
|  | snap-scaffold2608_size54790-abinit-gene-0.1-mRNA-1 | 1.46776973 | #N/A |
|  | PREDICTED: palmitoyltransferase ZDHHC2-like - [512927555_XP_004931364.1] | #REF! | #N/A |
|  | PREDICTED: nicotinamidase-like isoform X1 - [512904833_XP_004925794.1] | 1.46673925 | #N/A |
|  | snap-scaffold125_size151734-processed-gene-0.1-mRNA-1 | 1.46364098 | #N/A |
|  | snap-scaffold1112_size102494-abinit-gene-0.0-mRNA-1 | 1.46341538 | #N/A |
|  | PREDICTED: mitogen-activated protein kinase kinase kinase kinase 4-like isoform X5 - [512915926_XP_004928507.1] | 1.44900982 | #N/A |
|  | snap-scaffold5786_size34229-abinit-gene-0.0-mRNA-1 | 1.44722995 | #N/A |
|  | mitogen activated protein kinase kinase 2 - [170061049_XP_001866069.1] | 1.44701847 | #N/A |
|  | PREDICTED: E3 ubiquitin-protein ligase UBR3-like - [512902554_XP_004925404.1] | 1.4402174 | #N/A |
|  | snap-scaffold6714_size19185-abinit-gene-0.3-mRNA-1 | 1.43804081 | #N/A |
|  | YAP65-like protein - [182510216_NP_001116819.1] | 1.43559253 | #N/A |
|  | PREDICTED: similar to AGAP004892-PA - [91092856_XP_969290.1] | 1.43171093 | #N/A |
|  | PREDICTED: E3 ubiquitin-protein ligase Nedd-4-like - [512909803_XP_004927010.1] | 1.43063859 | #N/A |
|  | PREDICTED: putative gamma-glutamylcyclotransferase CG2811-like isoform X2 - [512900843_XP_004924990.1] | 1.42497048 | #N/A |
|  | PREDICTED: partitioning defective 3 homolog - [512924150_XP_004930528.1] | 1.42123722 | #N/A |
|  | PREDICTED: catenin delta-2-like - [512909191_XP_004926861.1] | 1.41661357 | #N/A |
|  | snap-scaffold4689_size36696-abinit-gene-0.1-mRNA-1 | 1.40490678 | #N/A |
|  | snap-scaffold2001_size44329-abinit-gene-0.6-mRNA-1 | 1.40361031 | #N/A |
|  | PREDICTED: chromatin accessibility complex protein 1-like - [512896353_XP_004923890.1] | 1.39606391 | #N/A |
|  | PREDICTED: putative inorganic phosphate cotransporter-like - [512902513_XP_004925393.1] | 1.39597889 | #N/A |
|  | snap-scaffold1017_size87795-abinit-gene-0.10-mRNA-1 | 1.39479011 | #N/A |
|  | snap-scaffold1211_size24441-abinit-gene-0.7-mRNA-1 | 1.39094381 | #N/A |
|  | snap-scaffold1536_size103565-abinit-gene-0.19-mRNA-1 | 1.39004257 | #N/A |
|  | eIF2B-beta protein - [112983292_NP_001037652.1] | 1.38012978 | #N/A |
|  | AGAP011292-PA - [158287296_XP_309356.4] | 1.3795671 | #N/A |
|  | PREDICTED: phosducin-like protein-like - [512934041_XP_004932937.1] | 1.37828536 | #N/A |
|  | snap-scaffold4053_size49547-abinit-gene-0.3-mRNA-1 | 1.37666784 | #N/A |
|  | PREDICTED: microfibrillar-associated protein 1-like - [512920528_XP_004929654.1] | 1.37612999 | #N/A |
|  | cyclin dependent kinase 4 - [274318355_NP_001162052.1] | 1.37266924 | #N/A |
|  | transcription initiation factor TFIID subunit 12 - [114051678_NP_001040422.1] | 1.37249279 | #N/A |
|  | cuticular protein RR-1 motif 26 precursor - [290563245_NP_001166724.1] | 1.37072774 | #N/A |
|  | PREDICTED: diphosphoinositol polyphosphate phosphohydrolase 2-like - [512897512_XP_004924171.1] | 1.37024331 | #N/A |
|  | PREDICTED: biorientation of chromosomes in cell division protein 1-like 1-like - [512934673_XP_004933089.1] | 1.36819227 | #N/A |
|  | snap-scaffold3574_size61875-processed-gene-0.9-mRNA-1 | 1.3678355 | #N/A |
|  | hypothetical protein CRE_31104 - [308500844_XP_003112607.1] | 1.36659338 | #N/A |
|  | myosin xv - [157129369_XP_001661658.1] | 1.36602354 | #N/A |
|  | PREDICTED: uncharacterized protein LOC101735334 - [512916241_XP_004928588.1] | 1.36178827 | #N/A |
|  | GG10523 - [194863057_XP_001970255.1] | 1.36085195 | #N/A |
|  | PREDICTED: protein wings apart-like - [512924376_XP_004930580.1] | 1.36056568 | #N/A |
|  | PREDICTED: catenin delta-2-like - [512909191_XP_004926861.1] | 1.3581443 | #N/A |
|  | PREDICTED: uncharacterized protein LOC101736321 - [512905740_XP_004926020.1] | 1.3571967 | #N/A |
|  | snap-scaffold4707_size24836-abinit-gene-0.2-mRNA-1 | 1.35703844 | #N/A |
|  | acid phosphatase isoenzyme - [114051630_NP_001040167.1] | 1.35600126 | #N/A |
|  | PREDICTED: ephrin-B2a-like - [512935518_XP_004933293.1] | 1.3539304 | #N/A |
|  | GH10500 - [195032868_XP_001988576.1] | 1.35355261 | #N/A |
|  | PREDICTED: similar to CG5639 CG5639-PA - [91087471_XP_967522.1] | 1.35172969 | #N/A |
|  | PREDICTED: PH and SEC7 domain-containing protein 4-like - [512926698_XP_004931151.1] | #REF! | #N/A |
|  | PREDICTED: uncharacterized protein LOC101743063 - [512893892_XP_004923293.1] | 1.34893013 | #N/A |
|  | snap-scaffold1810_size32065-abinit-gene-0.4-mRNA-1 | 1.34865141 | #N/A |
|  | PREDICTED: similar to exosome complex exonuclease RRP44 - [91088453_XP_969131.1] | 1.34591378 | #N/A |
|  | snap-scaffold907_size51334-abinit-gene-0.9-mRNA-1 | 1.34571509 | #N/A |
|  | PREDICTED: tyrosine-protein phosphatase non-receptor type 23-like - [512926237_XP_004931035.1] | 1.34156054 | #N/A |
|  | PREDICTED: LOW QUALITY PROTEIN: liprin-alpha-1-like - [512925321_XP_004930810.1] | 1.34130025 | #N/A |
|  | PREDICTED: LOW QUALITY PROTEIN: ubiquitin-conjugating enzyme E2 O-like - [512888094_XP_004922031.1] | 1.34096297 | #N/A |
|  | PREDICTED: uncharacterized protein LOC101744741, partial - [512895591_XP_004923709.1] | 1.34046114 | #N/A |
|  | snap-scaffold1126_size107384-abinit-gene-0.10-mRNA-1 | 1.34018005 | #N/A |
|  | PREDICTED: transcriptional adapter 1-like - [512913648_XP_004927947.1] | 1.33916345 | #N/A |
|  | prolactin regulatory binding-element protein - [114051449_NP_001040367.1] | 1.33872014 | #N/A |
|  | maker-scaffold344_size104086-snap-gene-0.23-mRNA-1 | 1.33790159 | #N/A |
|  | maker-scaffold6276_size19400-snap-gene-0.5-mRNA-1 | 1.33622031 | #N/A |
|  | PREDICTED: zinc finger protein Xfin-like - [512889725_XP_004922355.1] | 1.33575209 | #N/A |
|  | PREDICTED: THAP domain-containing protein 1-like - [512885533_XP_004921610.1] | 1.33395084 | #N/A |
|  | PREDICTED: zinc finger MYM-type protein 4-like - [512927657_XP_004931389.1] | 1.33373654 | #N/A |
|  | AGAP000222-PB - [347963397_XP_003436939.1] | 1.33288758 | #N/A |
|  | maker-scaffold4833_size29984-snap-gene-0.6-mRNA-1 | 1.33256088 | #N/A |
|  | 1-phosphatidylinositol-4,5-bisphosphate phosphodiesterase beta-4 - [284813575_NP_001165393.1] | 1.33225188 | #N/A |
|  | PREDICTED: ribosomal RNA processing protein 1 homolog - [512905688_XP_004926007.1] | 1.33204647 | #N/A |
|  | snap-scaffold9771_size8448-abinit-gene-0.1-mRNA-1 | 1.33027171 | #N/A |
|  | PREDICTED: 5'-3' exoribonuclease 1-like - [512930429_XP_004932066.1] | 1.33010138 | #N/A |
|  | snap-scaffold1606_size136675-abinit-gene-0.4-mRNA-1 | 1.32905498 | #DIV/0! |
|  | predicted protein - [156401135_XP_001639147.1] | 1.32718448 | #N/A |
|  | PREDICTED: uncharacterized protein LOC101737689 - [512913879_XP_004928001.1] | 1.3261112 | #N/A |
|  | PREDICTED: microtubule-associated protein futsch-like - [512901270_XP_004925094.1] | 1.32549787 | #N/A |
|  | PREDICTED: uncharacterized protein PFB0145c-like - [512894037_XP_004923331.1] | 1.32525736 | #N/A |
|  | PREDICTED: ashwin-like - [512924847_XP_004930693.1] | 1.32475434 | #N/A |
|  | PREDICTED: laminin subunit gamma-1-like - [512933533_XP_004932814.1] | 1.3246855 | #N/A |
|  | PREDICTED: luciferin 4-monooxygenase-like - [512912344_XP_004927632.1] | 1.32403668 | #N/A |
|  | snap-scaffold13089_size3622-abinit-gene-0.0-mRNA-1 | 1.32383361 | #N/A |
|  | PREDICTED: origin recognition complex subunit 2-like - [512896914_XP_004924025.1] | 1.32306337 | #N/A |
|  | snap-scaffold4758_size19411-abinit-gene-0.3-mRNA-1 | 1.32292207 | #N/A |
|  | PREDICTED: nuclear pore complex protein Nup98-Nup96-like - [512889721_XP_004922354.1] | 1.32161779 | #N/A |
|  | PREDICTED: similar to synaptotagmin X - [91080815_XP_970240.1] | 1.32053809 | #N/A |
|  | PREDICTED: G-protein coupled receptor 64-like - [498953459_XP_004523504.1] | 1.31990458 | #N/A |
|  | snap-scaffold1638_size20423-processed-gene-0.3-mRNA-1 | 1.31780222 | #N/A |
|  | PREDICTED: dehydrogenase/reductase SDR family member 13-like - [512898081_XP_004924311.1] | 1.31684457 | #N/A |
|  | PREDICTED: UBX domain-containing protein 6-like - [512936666_XP_004933573.1] | 1.31593708 | #N/A |
|  | PREDICTED: sideroflexin-2-like - [512924827_XP_004930688.1] | 1.31557647 | #N/A |
|  |  |  |  |
|  | PREDICTED: uncharacterized protein LOC101737920 - [512921994_XP_004930011.1] | 1.31393584 | #N/A |
|  | PREDICTED: TELO2-interacting protein 1 homolog - [512895105_XP_004923593.1] | 1.31327943 | #N/A |
|  | AGAP009285-PA, partial - [158300089_XP_320082.4] | 1.31319832 | #N/A |
|  | PREDICTED: LOW QUALITY PROTEIN: PHD finger protein rhinoceros-like - [512933845_XP_004932892.1] | 1.31244704 | #N/A |
|  | PREDICTED: synapse-associated protein of 47 kDa-like - [512904919_XP_004925816.1] | 1.31230228 | #N/A |
|  | PREDICTED: OTU domain-containing protein 6B-like - [512926016_XP_004930979.1] | #REF! | #N/A |
|  | PREDICTED: oxysterol-binding protein-related protein 9-like - [512897885_XP_004924262.1] | 1.31171444 | #N/A |
|  | PREDICTED: similar to rCG37751 - [91077136_XP_971386.1] | 1.31087404 | #N/A |
|  | PREDICTED: tyrosine-protein kinase-like otk-like - [512913988_XP_004928029.1] | 1.30893729 | #N/A |
|  | PREDICTED: hypothetical protein LOC100745708 - [350421176_XP_003492760.1] | 1.30869214 | #N/A |
|  | PREDICTED: mitotic spindle assembly checkpoint protein MAD1-like - [512936023_XP_004933417.1] | 1.30860028 | #N/A |
|  | PREDICTED: inhibitor of Bruton tyrosine kinase-like - [512890003_XP_004922428.1] | 1.30851133 | #N/A |
|  | maker-scaffold116_size192256-snap-gene-0.67-mRNA-1 | 1.30767998 | #N/A |
|  | PREDICTED: protein furry-like - [350406444_XP_003487772.1] | 1.30592729 | #N/A |
|  | AGAP000810-PA - [347964550_XP_311349.5] | 1.30566131 | #N/A |
|  | PREDICTED: hypothetical protein LOC724973 - [328790073_XP_001120873.2] | 1.30407232 | #N/A |
|  | PREDICTED: vacuolar protein sorting-associated protein 72 homolog - [512894892_XP_004923540.1] | 1.30397058 | #N/A |
|  | PREDICTED: zinc finger protein 532-like - [512922669_XP_004930173.1] | 1.30364901 | #N/A |
|  | PREDICTED: F-box only protein 28-like isoform X2 - [512901737_XP_004925208.1] | 1.30340591 | #N/A |
|  | PREDICTED: peripheral plasma membrane protein CASK-like - [512895026_XP_004923573.1] | 1.30248496 | #N/A |
|  | PREDICTED: carboxy-terminal domain RNA polymerase II polypeptide A small phosphatase 1-like isoform X1 - [512918705_XP_004929197.1] | 1.30136286 | #N/A |
|  | PREDICTED: uncharacterized protein LOC101741471 - [512887646_XP_004921957.1] | 1.30098492 | #N/A |
|  | PREDICTED: protein lunapark-B-like - [383859089_XP_003705030.1] | 1.29960146 | #N/A |
|  | PREDICTED: serine/threonine-protein phosphatase 2A 56 kDa regulatory subunit delta isoform-like - [512886702_XP_004921802.1] | 1.29958371 | #N/A |
|  | PREDICTED: protein TANC2-like - [345485907_XP_003425367.1] | 1.29709547 | #N/A |
|  | PREDICTED: multiple inositol polyphosphate phosphatase 1-like - [512923788_XP_004930437.1] | 1.29708174 | #N/A |
|  | dynactin 4 protein - [114052957_NP_001040117.1] | 1.29404156 | #N/A |
|  | snap-scaffold22565_size666-abinit-gene-0.0-mRNA-1 | 1.29334268 | #N/A |
|  | PREDICTED: serine/threonine-protein kinase tousled-like 1-like - [328723550_XP_001947282.2] | 1.29160526 | #N/A |
|  | snap-scaffold4692_size17240-abinit-gene-0.4-mRNA-1 | 1.2913538 | #N/A |
|  | PREDICTED: phosphatidylinositol N-acetylglucosaminyltransferase subunit A-like isoform X1 - [512912825_XP_004927750.1] | 1.29127771 | #N/A |
|  | snap-scaffold1883_size71588-abinit-gene-0.23-mRNA-1 | 1.29068742 | #N/A |
|  | maker-scaffold1706_size46956-snap-gene-0.8-mRNA-1 | 1.2906461 | #N/A |
|  | PREDICTED: tudor domain-containing protein 7-like - [512923292_XP_004930319.1] | 1.28967678 | #N/A |
|  | PREDICTED: cathepsin O-like - [512891322_XP_004922663.1] | 1.28964293 | #N/A |
|  | PREDICTED: myosin-9-like - [512914277_XP_004928102.1] | 1.28927098 | #N/A |
|  | PREDICTED: CDP-diacylglycerol--inositol 3-phosphatidyltransferase-like - [383860604_XP_003705779.1] | 1.28863832 | #N/A |
|  | PREDICTED: probable serine hydrolase-like - [512897385_XP_004924139.1] | 1.28822926 | #N/A |
|  | snap-scaffold3032_size27942-abinit-gene-0.1-mRNA-1 | 1.28797942 | #N/A |
|  | PREDICTED: carbohydrate-responsive element-binding protein-like - [512900351_XP_004924870.1] | 1.28744121 | #N/A |
|  | PREDICTED: putative glycogen [starch] synthase-like - [340722683_XP_003399733.1] | 1.28714744 | #N/A |
|  | PREDICTED: nuclear pore complex protein Nup88-like - [512891832_XP_004922780.1] | 1.28681755 | #N/A |
|  | PREDICTED: ran-binding protein 10-like - [512897865_XP_004924257.1] | 1.28629724 | #N/A |
|  | PREDICTED: post-GPI attachment to proteins factor 2-like - [512919944_XP_004929508.1] | 1.28620369 | #N/A |
|  | maker-scaffold7256_size37059-snap-gene-0.8-mRNA-1 | 1.28508308 | #N/A |
|  | PREDICTED: DNA ligase 4-like - [512923088_XP_004930267.1] | 1.28477591 | #N/A |
|  | PREDICTED: testin-like - [512924307_XP_004930564.1] | 1.28445761 | #N/A |
|  | maker-scaffold3363_size22755-snap-gene-0.20-mRNA-1 | 1.28388156 | #N/A |
|  | snap-scaffold3438_size22401-abinit-gene-0.1-mRNA-1 | 1.2833428 | #N/A |
|  | snap-scaffold8709_size8755-abinit-gene-0.0-mRNA-1 | 1.28314894 | #N/A |
|  | PREDICTED: BTB/POZ domain-containing protein KCTD5-like - [512932285_XP_004932508.1] | 1.28270853 | #N/A |
|  | PREDICTED: retrograde Golgi transport protein RGP1 homolog - [512894045_XP_004923335.1] | 1.28269005 | #N/A |
|  | snap-scaffold6276_size19400-abinit-gene-0.3-mRNA-1 | 1.28210167 | #N/A |
|  | PREDICTED: probable dolichyl pyrophosphate Glc1Man9GlcNAc2 alpha-1,3-glucosyltransferase-like - [512913313_XP_004927866.1] | 1.28152548 | #N/A |
|  | PREDICTED: ubiquitin carboxyl-terminal hydrolase 16-like - [512896752_XP_004923985.1] | 1.2811197 | #N/A |
|  | PREDICTED: AN1-type zinc finger protein 6-like isoform X1 - [512924211_XP_004930542.1] | 1.28086723 | #N/A |
|  | GE18601 - [195472441_XP_002088509.1] | 1.28068119 | #N/A |
|  | snap-scaffold2571_size27194-abinit-gene-0.11-mRNA-1 | 1.28065911 | #N/A |
|  | PREDICTED: elongation of very long chain fatty acids protein 6-like - [512900028_XP_004924792.1] | 1.27986798 | #N/A |
|  | PREDICTED: LOW QUALITY PROTEIN: midasin-like - [512890270_XP_004922489.1] | 1.27959632 | #N/A |
|  | maker-scaffold1911_size54947-snap-gene-0.11-mRNA-1 | 1.27939937 | #N/A |
|  | PREDICTED: HMG box-containing protein 4-like - [512925219_XP_004930786.1] | 1.27928453 | #N/A |
|  | PREDICTED: atlastin-like - [512923057_XP_004930260.1] | 1.2789415 | #N/A |
|  | PREDICTED: rho GTPase-activating protein 17-like - [350407047_XP_003487967.1] | 1.27817832 | #N/A |
|  | PREDICTED: KAT8 regulatory NSL complex subunit 1-like protein-like - [512902999_XP_004925512.1] | 1.27816643 | #N/A |
|  | PREDICTED: similar to metaxin 2 - [91085775_XP_974308.1] | 1.27806039 | #N/A |
|  | PREDICTED: serine protease easter-like - [512894586_XP_004923466.1] | 1.27782199 | #N/A |
|  | snap-scaffold5531_size7972-abinit-gene-0.2-mRNA-1 | 1.27754858 | #N/A |
|  | PREDICTED: uncharacterized protein LOC101742729 - [512902353_XP_004925355.1] | 1.27658416 | #N/A |
|  | PREDICTED: uncharacterized protein LOC101736046 - [512907107_XP_004926361.1] | 1.276353 | #N/A |
|  | snap-scaffold5149_size13376-abinit-gene-0.0-mRNA-1 | 1.27612023 | #N/A |
|  | PREDICTED: condensin-2 complex subunit D3-like - [512903061_XP_004925526.1] | 1.27529171 | #N/A |
|  | PREDICTED: dyslexia-associated protein KIAA0319-like - [512929563_XP_004931852.1] | 1.27484646 | #N/A |
|  | PREDICTED: PHD finger and CXXC domain-containing protein CG17446-like - [380018756_XP_003693289.1] | 1.27476847 | #N/A |
|  | PREDICTED: protein Vhl-like - [512905804_XP_004926036.1] | 1.27474132 | #N/A |
|  | PREDICTED: uncharacterized protein LOC101736188 - [512916605_XP_004928681.1] | 1.27419688 | #N/A |
|  | DnaJ (Hsp40) homolog 13 - [114052925_NP_001040115.1] | 1.27418825 | #N/A |
|  | snap-scaffold8650_size7288-abinit-gene-0.0-mRNA-1 | 1.27404969 | #N/A |
|  | maker-scaffold4477_size22609-snap-gene-0.8-mRNA-1 | 1.27357179 | #N/A |
|  | snap-scaffold7973_size26449-abinit-gene-0.7-mRNA-1 | 1.27356294 | #N/A |
|  | PREDICTED: cytoplasmic dynein 1 intermediate chain - [328783012_XP_396853.3] | 1.27346976 | #N/A |
|  | PREDICTED: TP53RK-binding protein-like - [512887994_XP_004922015.1] | 1.27290965 | #N/A |
|  | PREDICTED: tudor domain-containing protein 7-like - [512923292_XP_004930319.1] | 1.27275591 | #N/A |
|  | PREDICTED: scaffold protein salvador-like - [512898922_XP_004924516.1] | 1.27257189 | #N/A |
|  | PREDICTED: transmembrane protein 242-like - [512929734_XP_004931896.1] | 1.27234273 | #N/A |
|  | PREDICTED: RNA-directed DNA polymerase from mobile element jockey-like - [391336237_XP_003742488.1] | 1.27230373 | #N/A |
|  | PREDICTED: uncharacterized protein LOC101744570 - [512921040_XP_004929784.1] | 1.27219645 | #N/A |
|  | conserved hypothetical protein - [170060583_XP_001865868.1] | 1.27202411 | #N/A |
|  | PREDICTED: RNA-directed DNA polymerase from mobile element jockey-like - [328707593_XP_003243439.1] | 1.27178349 | #N/A |
|  | PREDICTED: uncharacterized protein LOC101746356 - [512932908_XP_004932660.1] | 1.27170289 | #DIV/0! |
|  | hypothetical protein - [256078665_XP_002575615.1] | 1.27092079 | #N/A |
|  | snap-scaffold2087_size64639-abinit-gene-0.2-mRNA-1 | 1.27078166 | #N/A |
|  | PREDICTED: muscle M-line assembly protein unc-89-like - [512915547_XP_004928416.1] | 1.2702014 | #N/A |
|  | PREDICTED: LIM and senescent cell antigen-like-containing domain protein 2-like isoform X1 - [512924403_XP_004930586.1] | 1.26992663 | #N/A |
|  | PREDICTED: protein UXT homolog - [512886623_XP_004921789.1] | 1.26983856 | #N/A |
|  | PREDICTED: tonsoku-like protein-like - [512934234_XP_004932985.1] | 1.26958015 | #N/A |
|  | PREDICTED: myoneurin-like isoform X2 - [512929642_XP_004931872.1] | 1.2694699 | #N/A |
|  | PREDICTED: long-chain-fatty-acid--CoA ligase 5-like - [512929388_XP_004931808.1] | 1.26915056 | #N/A |
|  | PREDICTED: putative inorganic phosphate cotransporter-like - [512902513_XP_004925393.1] | 1.26896135 | #N/A |
|  | PREDICTED: uncharacterized protein LOC101738702 - [512903076_XP_004925530.1] | #REF! | #N/A |
|  | GE19029 - [195474003_XP_002089281.1] | 1.26891222 | #N/A |
|  | PREDICTED: mediator of RNA polymerase II transcription subunit 1-like - [512931284_XP_004932264.1] | 1.2685948 | #N/A |
|  | unc-50-like protein - [114050867_NP_001040161.1] | 1.26831575 | #N/A |
|  | argonaute 3 - [166706858_NP_001098067.2] | 1.26802584 | #N/A |
|  | PREDICTED: lachesin-like - [512923335_XP_004930330.1] | 1.26771011 | #N/A |
|  | PREDICTED: uncharacterized protein LOC101743370 - [512924697_XP_004930658.1] | 1.26758704 | #N/A |
|  | PREDICTED: transcription elongation factor B polypeptide 3-like - [512924453_XP_004930599.1] | 1.26755963 | #N/A |
|  | PREDICTED: non-structural maintenance of chromosomes element 1 homolog isoform X1 - [512924550_XP_004930622.1] | 1.2673667 | #N/A |
|  | PREDICTED: LOW QUALITY PROTEIN: protein SMG5-like - [512920361_XP_004929614.1] | 1.26706781 | #N/A |
|  | replication protein A2 - [112983110_NP_001036939.1] | 1.26521568 | #N/A |
|  | PREDICTED: mediator of RNA polymerase II transcription subunit 10-like isoform X1 - [512933968_XP_004932921.1] | 1.26417445 | #N/A |
|  | PREDICTED: solute carrier family 35 member B1 homolog - [512935221_XP_004933221.1] | 1.26368186 | #N/A |
|  | PREDICTED: cadherin-23-like - [512920189_XP_004929570.1] | 1.26350433 | #N/A |
|  | PREDICTED: uncharacterized protein LOC101739600 isoform X1 - [512914678_XP_004928202.1] | 1.26344167 | #N/A |
|  | DNA polymerase eta - [157106621_XP_001649408.1] | 1.26331328 | #N/A |
|  | PREDICTED: similar to AGAP007474-PA - [189238749_XP_972434.2] | 1.2632744 | #N/A |
|  | GL25215 - [195160733_XP_002021228.1] | 1.26310567 | #N/A |
|  | conserved hypothetical protein - [170061901_XP_001866436.1] | 1.2625569 | #N/A |
|  | PREDICTED: testis-expressed sequence 10 protein-like - [512932987_XP_004932679.1] | 1.26252539 | #N/A |
|  | GJ16276 - [195401244_XP_002059224.1] | 1.26218993 | #N/A |
|  | conserved hypothetical protein - [170032546_XP_001844142.1] | 1.26204355 | #N/A |
|  | phosphomevalonate kinase - [114050987_NP_001040145.1] | 1.26201318 | #N/A |
|  | PREDICTED: FAS-associated factor 2-like - [512929299_XP_004931787.1] | 1.26170839 | #N/A |
[truncated: 212,543 more chars]
